# Photo‐Induced Homologation of Carbonyl Compounds for Iterative Syntheses

**DOI:** 10.1002/anie.202211578

**Published:** 2022-11-10

**Authors:** Hua Wang, Shun Wang, Vincent George, Galder Llorente, Burkhard König

**Affiliations:** ^1^ Faculty of Chemistry and Pharmacy University Regensburg 93040 Regensburg Germany; ^2^ Department of Chemistry, School of Pharmacy The Fourth Military Medical University Xi'an 710032 P. R. China

**Keywords:** Büchner–Curtius–Schlotterbeck Reaction, Carbonyl Homologation, Diazo Compounds, Hydrazone, Photochemistry

## Abstract

We describe a photo‐induced reaction for the in situ generation of highly reactive alkyl diazo species from carbonyl precursors via photo‐excitation of N‐tosylhydrazone anions. The diazo intermediates undergo efficient C−H insertion of aldehydes, leading to the productive synthesis of aldehydes and ketones. The method is applicable to the iterative synthesis of densely functionalized carbonyl compounds through sequential trapping of the diazo species with various aldehydes. The reaction proceeds without the need of any catalyst by light irradiation and features high functional group tolerance. More than 70 examples, some performed on a gram‐scale, demonstrate the broad applicability of this reaction sequence in synthesis.

## Graphical abstract

A photo‐induced Büchner‐Curtius‐Schlotterbeck type reaction for carbonyl homologation is described. The protocol allows the use of carbonyl compounds as safe and readily available diazo precursors through direct photoexcitation of corresponding N‐tosylhydrazone anions. Functionalized aliphatic aldehydes and ketones are prepared in a practical and iterative manner.

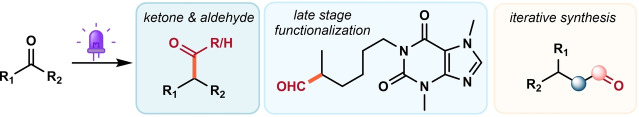

## Introduction

**Scheme 1:**
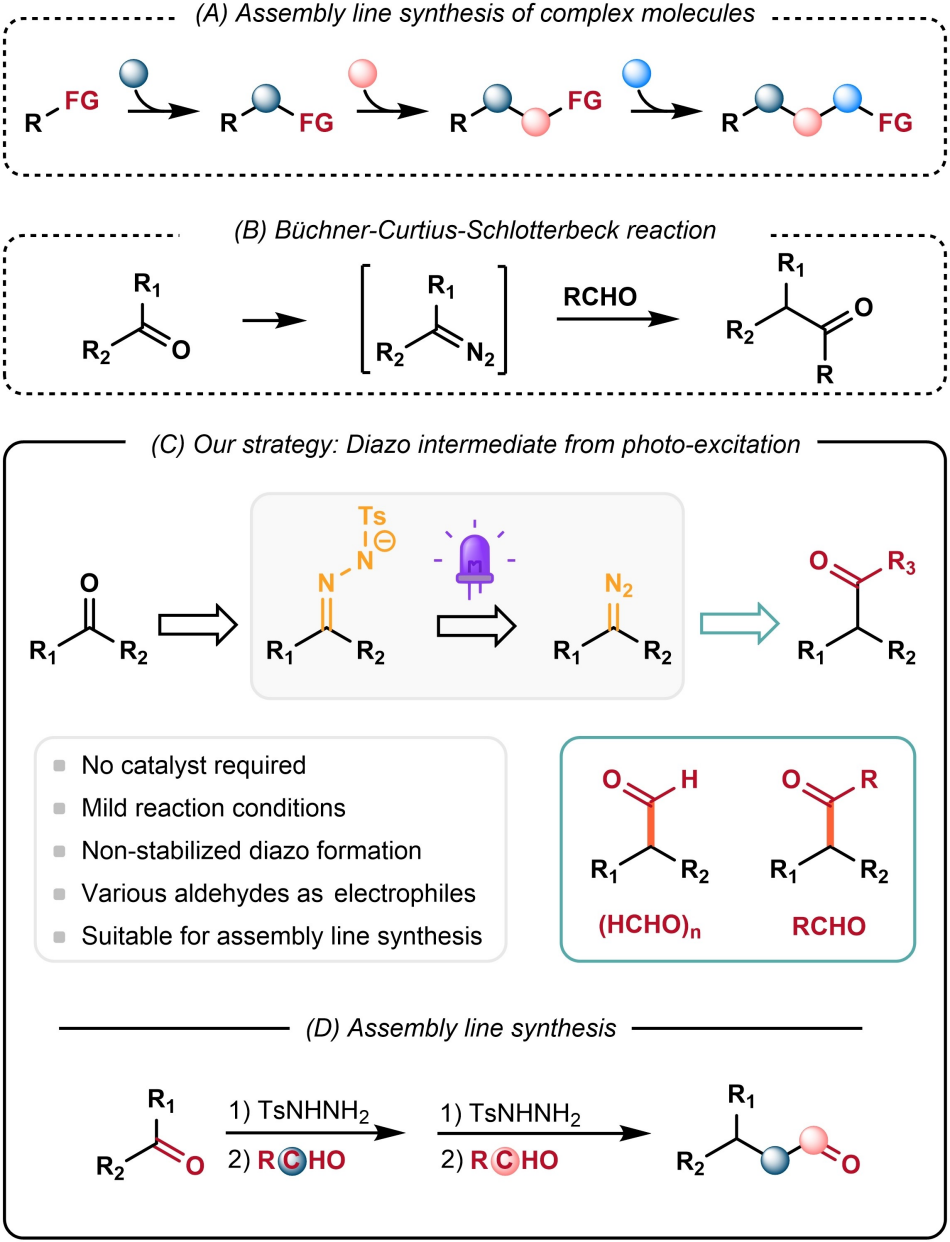
Carbonyl compound synthesis via C−H insertion of aldehydes with diazoalkane.

Iterative synthesis is a widely adopted strategy in nature for the construction of complex molecules from simple starting materials, for example, the biosynthesis of polyketides, fatty acids or peptides in living bodies.^[1]^ Iterative approaches use repeated synthesis steps with similar building blocks and reaction conditions, introducing molecular complexity in a modular manner (Scheme 1A).^[2]^ By harnessing the inherent modularity of iterative synthesis, automated synthetic platforms were developed for biopolymers such as peptides,^[3]^ oligonucleotides,^[4]^ and oligosaccharides.^[5]^ In recent years, iterative building block‐based synthesis approaches have also demonstrated their potential in the rapid assembly of complex small molecules.^[6]^ Notable examples include Crudden's preparation of polyarylated structures by iterative Suzuki–Miyaura cross‐couplings,^[7]^ Burke's synthesis of polyenes with (MIDA)boronates,[^6b^, ^8^] and Aggarwal's construction of stereo‐defined carbon chains via homologation of boronic esters^[9]^ have demonstrated the power of this concept for the construction a diverse range of complex molecules. Despite these transformative advances, reactions and strategies available for chemists to unlock a more generalized synthesis platform remained limited.

Carbonyls are arguably one of the most widespread and fundamental functional groups in organic compounds. Though numerous versatile transformations and strategies have been developed for the synthesis of carbonyl compounds, available strategies capable of being applied for an ideal iterative construction of these compounds have been scarce.^[10]^ In particular, the nucleophilic addition of diazo species to ketones or aldehydes constitutes a useful strategy to synthesize one‐carbon chain‐extended carbonyl compounds, which is known as Büchner–Curtius–Schlotterbeck (BCS) reaction (Scheme 1B).^[11]^ This transformation presents an appealing opportunity to achieve the iterative synthesis of ketones due to the fact that carbonyl compounds could serve as convenient precursors of diazo intermediates in the ensuing transformations. While the reaction of aldehydes with α‐stabilized diazo compounds has been extensively studied, corresponding reactions using non‐stabilized diazos are under‐exploited due to their problematic instability and acute toxicity, which hampered so far their broad use in synthesis.^[12]^ Alternatively, efforts have been devoted to develop carbonyl homologation reactions involving in situ generation and transformation of highly reactive non‐stabilized diazo species.^[13]^ For instance, Angle and Aggarwal[^14^, ^15^] showed that aromatic N‐tosylhydrazones or N‐tosylhydrazone salts, under thermal conditions, are effective precursors for the in situ generation of aryl‐stabilized diazo compounds, which enable facile C−H insertion reaction of aldehydes. Later, Allwood extended this reaction system to the generation of alkyl diazo compounds at elevated temperature, which was employed for the C−H insertion of aryl aldehydes.^[16]^ Ley and co‐workers recently disclosed an elegant procedure for the generation of non‐stabilized alkyl diazo intermediates via UV photolysis of oxadiazolines.^[17]^ Their reaction system was adapted to a flow photoreactor and was applicable for the generation of aliphatic ketones and aldehydes by utilizing aliphatic aldehydes and formaldehyde as electrophiles.^[18]^ Despite these achievements, we considered this valuable transformation worthy of further investigation since the existing methods commonly suffer from limited substrate scope and harsh conditions, or the use of special reagents, which hampers their broad application in the iterative synthesis of complex carbonyl compounds. We posited that the reactive alkyl diazo species could be generated and transformed in situ under mild photochemical conditions from easily accessible N‐tosylhydrazone, thus presenting a facile and automatable synthesis method for complex carbonyl compounds.

Aiming at devising an efficient and versatile way of both making and coupling a carbonyl moiety to realize iterative synthesis, we applied photochemical pathways for the in situ generation of alkyl diazo species from carbonyl derived N‐tosylhydrazone.^[19]^ Building on our continuing interest in the reactivity of photo‐excited states of anionic species,^[20]^ we envisioned that the generation of a diazo intermediate would be facilitated by the extrusion of tosylate from the excited state of a tosylhydrazone anion.^[21]^ Subsequent trapping by carbonyl electrophiles affords ketones or aldehydes as final products (Scheme 1C). The synthetic operation is easily made iterative by using the product carbonyl compounds in a subsequent reaction via simple condensation with tosylhydrazine. Notably, this synthetic platform would present an efficient hydrazone‐mediated carbonyl umpolung strategy for C−C bond formation as pioneered by Li.^[22]^ Herein, we report a light‐induced strategy for homologation of carbonyl compounds, utilizing the excited state of tosylhydrazone anion as alkyl diazo precursor. Notably, this reaction system allows the facile synthesis of aliphatic ketones and aldehydes by using paraformaldehyde and aliphatic aldehyde as electrophiles (Scheme 1D).

## Results and Discussion

We commenced our investigations of the proposed photochemical carbonyl homologation using cyclohexyl *N*‐tosylhydrazone **1** **a** and paraformaldehyde **2** as model substrates. At the outset, we measured the absorption spectra of cyclohexanone derived *N*‐tosylhydrazone **1** **a**. The absorption of compound **1** **a** in MeCN solution shows an absorption band exclusively in the UV region (<340 nm), while the addition of Cs_2_CO_3_ (1.0 equiv) caused a clear bathochromic shift by ≈50 nm that extends into near visible light region (≈390 nm) (Figure S8). Pleasingly, irradiation of the acetonitrile solution of **1** **a**, paraformaldehyde and Cs_2_CO_3_ (1.5 equiv) with a 385 nm LED (0.5 W) at 25 °C for 20 hours afforded the desired aldehyde **3** **a** in 70 % yield (Table 1, entry 1). It is noteworthy that pre‐thermolysis of solid paraformaldehyde as reported in the work of Ley^[18a]^ and Kingsbury^[10b]^ was not required in our reaction. Next, we found that using DMF, toluene or other solvents instead of MeCN as solvent led to diminished yields (entries 2 and 3; see Supporting Information for more details). The reaction did not proceed when a 455 nm LED was applied (Table 1, entry 4), and a slightly decreased yield (65 %) was obtained when using a 365 nm LED (Table 1, entry 5). We then found that increasing the light intensity of 385 nm LED to 3 W did not provide better results (Table 1, entry 6). The optimal conditions were identified after careful optimization of the amount of reagents: Decreasing the amount of **2** to 1.2 equivalent dramatically improved the efficiency (Table 1, entry 7). It is worth mentioning that the formation of doubly homologated ketone product was not observed under this conditions.^[10b]^ Rigorous control experiments were carried out, revealing that both base and light were crucial for the reaction to occur (Table 1, entries 8, 9). Finally, the reaction was evaluated under thermal conditions, no product was observed even increasing the temperature to 110 °C, thus highlighting the unique activation by irradiation in our protocol.^[16]^

**Table 1:** Optimization of reaction conditions for cyclic substrates.

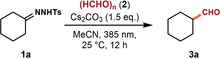		
		
Entry	Changes from standard conditions	Yield [%]^[a]^
1	None	70
2	DMF instead of MeCN	20
3	PhMe instead of MeCN	50
4	455 nm LED (0.5 W)	Trace
5	365 nm LED (0.5 W)	65
6	385 nm LED (3 W)	68
7	1.2 equiv of **2** was used	90
8	In the dark	n.d.^[b]^
9	Without Cs_2_CO_3_	n.d.
10	In the dark and heating to 110 °C	n.d.^[c]^

[a] Reaction conditions: **1** **a** (0.2 mmol, 1.0 equiv), **2** (0.3 mmol, 1.5 equiv), and Cs_2_CO_3_ (0.3 mmol, 1.5 equiv) in MeCN (2 mL), irradiation with a 385 nm LED (0.5 W) at 25 °C under N_2_. Yields were determined by GC analysis of the crude reaction mixtures. [b] Conversion of **1** **a** <5 %. [c] Cyclohexene was detected as the major product.

With the optimized conditions in hand, we first explored the scope with respect to *N*‐tosylhydrazones derived from aldehydes and ketones as shown in Table 2.A variety of six‐ to fifteen‐membered ring systems, including cyclohexyl, adamantyl, cyclododecanyl and cyclopentadecanyl substituted *N*‐tosylhydrazones reacted smoothly with paraformaldehyde to produce the one‐carbon extended aldehydes **3** **a**–**3** **f** with moderate to excellent yields. Boc‐protected piperdinone moieties are well tolerated, affording product **3** **g** in moderate yield. Subsequently, we found that the reactivity could be switched into a double homologation of tosylhydrazone by using paraformaldehyde as the limiting reagent, and this permits a straightforward access to symmetrical ketones, such as compound **3** **h**. Moreover, with slightly modified conditions, *N*‐tosylhydrazones derived from acetophenones could smoothly undergo the related transformation to afford the desired aldehydes **3** **j** and **3** **k**. More bulkier alkyl substitution at the α‐position of the carbonyl was compatible with the conditions (**3** **l**–**3** **n**, ^i^Pr, Cy, and ^t^Bu), providing the aldehydes between 42 and 56 % yield. It is to be noted that the N_2_ elimination product is the major byproduct for substrates with an α‐proton. We were delighted to find that both aromatic and aliphatic aldehyde‐derived tosylhydrazones readily participated in the coupling reaction to give the one‐carbon extended aldehyde **3** **o**–**3** **q** in reasonable yields.

**Table 2:**
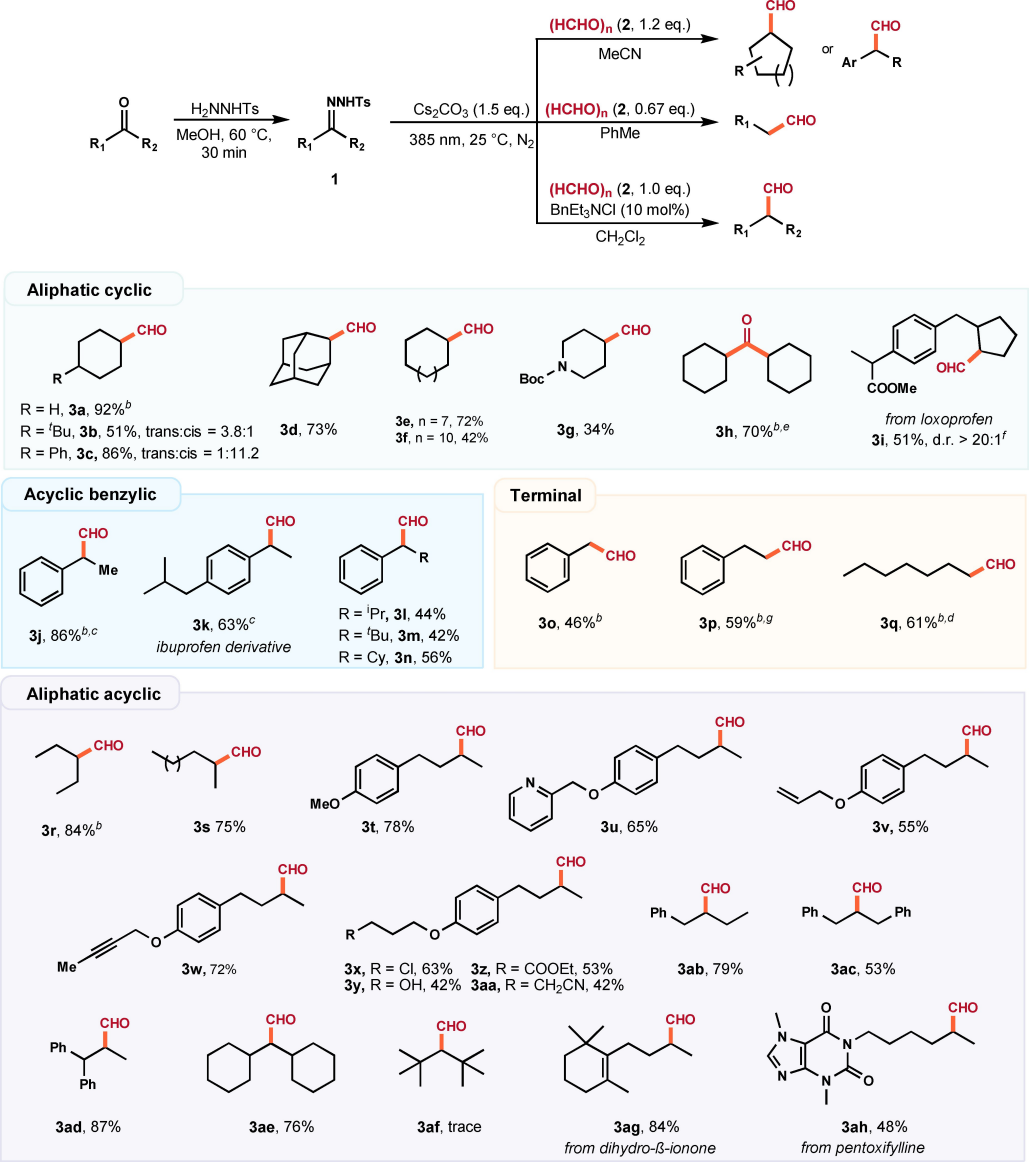
Scope of carbonyl homologation to access aliphatic aldehydes.

[a] Reaction conditions: **1**, **2**, and Cs_2_CO_3_ (0.3 mmol, 1.5 equiv) in dry solvent (2 mL), irradiation with a 385 nm LED (0.5 W) at 25 °C under N_2_. Yields of isolated products are given. [b] GC yield by using mesitylene or anisole as an internal standard. [c] **1** (0.4 mmol, 2.0 equiv), **2** (0.2 mmol, 1.0 equiv). [d] **1** (0.3 mmol, 1.5 equiv), **2** (0.20 mmol, 1.0 equiv), toluene (2 mL). [e] **1** (0.5 mmol, 2.5 equiv), **2** (0.20 mmol, 1.0 equiv). [f] Configuration of this product was not assigned due to NMR signal overlap. [g] Reaction was conducted in DCM (2 mL).

These conditions can therefore be widely applied to hydrazones from cyclic ketones. However, aliphatic acyclic hydrazones gave generally lower yields than cyclic ones under these conditions. We therefore reoptimized the conditions for this class of substrates as shown in Table S8 (see Supporting Information). Switching from MeCN to CH_2_Cl_2_ gave a 66 % yield. Furthermore, slight improvements were made using equimolar instead of excess amounts of paraformaldehyde. Finally, adding 10 mol % of BnEt_3_NCl that was previously used by Aggarwal et al. to improve the solubility of deprotonated hydrazone salts gave us the final conditions with a 78 % yield.^[15]^ Analogous to previous reports, increasing the amount of PTC used from 10 mol % to 20 mol % reduced the yield drastically.

With these optimized conditions in hand, we explored the substrate scope of the reaction. Acyclic substrates were now successfully homologated into the corresponding aldehydes with moderate to good efficiency (**3** **r**–**3** **ae**). Notably, a broad range of synthetically valuable functional groups could be well‐tolerated, including pyridine (**3** **u**), alkene (**3** **v**), alkyne (**3** **w**), chloride (**3** **x**), hydroxyl (**3** **y**), ester (**3** **z**), and nitrile (**3** **aa**) on the carbon chain remained untouched. Sterically hindered dicyclohexyl tosylhydrazone afforded the target product **3** **ae** in good yield, while bulkier di‐tert‐butyl substituted hydrazone underwent significant decomposition and failed to provide the desired product. The synthetic applicability of this strategy was further demonstrated by the late‐stage functionalization of several structurally and functionally complex molecules such as loxoprofen (**3** **i**), dihydro‐β‐ionone (**3** **ag**), and pentoxifylline (**3** **ah**) derivatives.

Having established a viable access to aldehydes, we turned our attention into the construction of ketones through the trapping of diazo intermediates with aldehyde electrophiles. We anticipated that the success of this reaction will unlock the iterative synthesis of both ketones and aldehydes via a unified approach. With this idea in mind, we examined the use of aldehydes in the reaction with tosylhydrazones. As summarized in Table 3, aliphatic aldehydes with varied chain lengths and cyclic ring structures participated well in the transformation to yield the unsymmetrical ketones in moderate to excellent yields (**5** **a**–**5** **j**). More sterically demanding branched aldehydes were found to be suitable substrates (**5** **k**–**5** **p**). Moreover, aldehydes bearing labile functional groups such as olefinic moieties (**5** **q**–**5** **r**), alkyl chloride (**5** **s**), and hydroxyl (**5** **t**) remained intact in the reaction. When isophthalaldehyde was employed in the reaction, bifunctionalization occurred smoothly to afford the expected diketone (**5** **u**). The reaction also proceeded efficiently for acetyl‐protected 5α‐dihydrotestosterone, affording the ketone derivative **5** **v** in a 50 % yield.^[23]^ Aromatic aldehydes bearing ester (**5** **w**), methoxy (**5** **y**), alkene (**5** **aa**), alkyne (**5** **ab**), pyridine (**5** **z**, **5** **ac**), and halogen (**5** **x**, **5** **ad**–**5** **af**) groups were fully feasible, delivering the aromatic ketone products in good yields. When cyclopropylethanone‐derived tosylhydrazone was employed, the corresponding ketone product was obtained in a 52 % yield (**5** **ag**), with no formation of ring‐opening product. While the scope of aldehyde formation is heavily dependent on the conditions used, changing the solvent from MeCN to CH_2_Cl_2_ and adding BnEt_3_NCl has almost no effect on the yield for many substrates tested and can therefore be seen as viable alternative reaction conditions. However, terminal hydrazones failed to produce the desired ketone products with reasonable yields under the above reaction conditions. Changing the solvent to DMSO and increasing the concentration considerably improved this transformation (see Supporting Information, Table S9 for more details). With new conditions in hand, ketones **5** **ah**–**5** **an** were obtained in moderate to good yields with excellent functional group tolerance. Since one of the R groups is a hydrogen atom, sterically demanding *tert*‐butyl groups can be incorporated without significant impact on the yield (**5** **am**–**5** **an**), even when paired with bulky α‐substituted aldehydes.

**Table 3:**
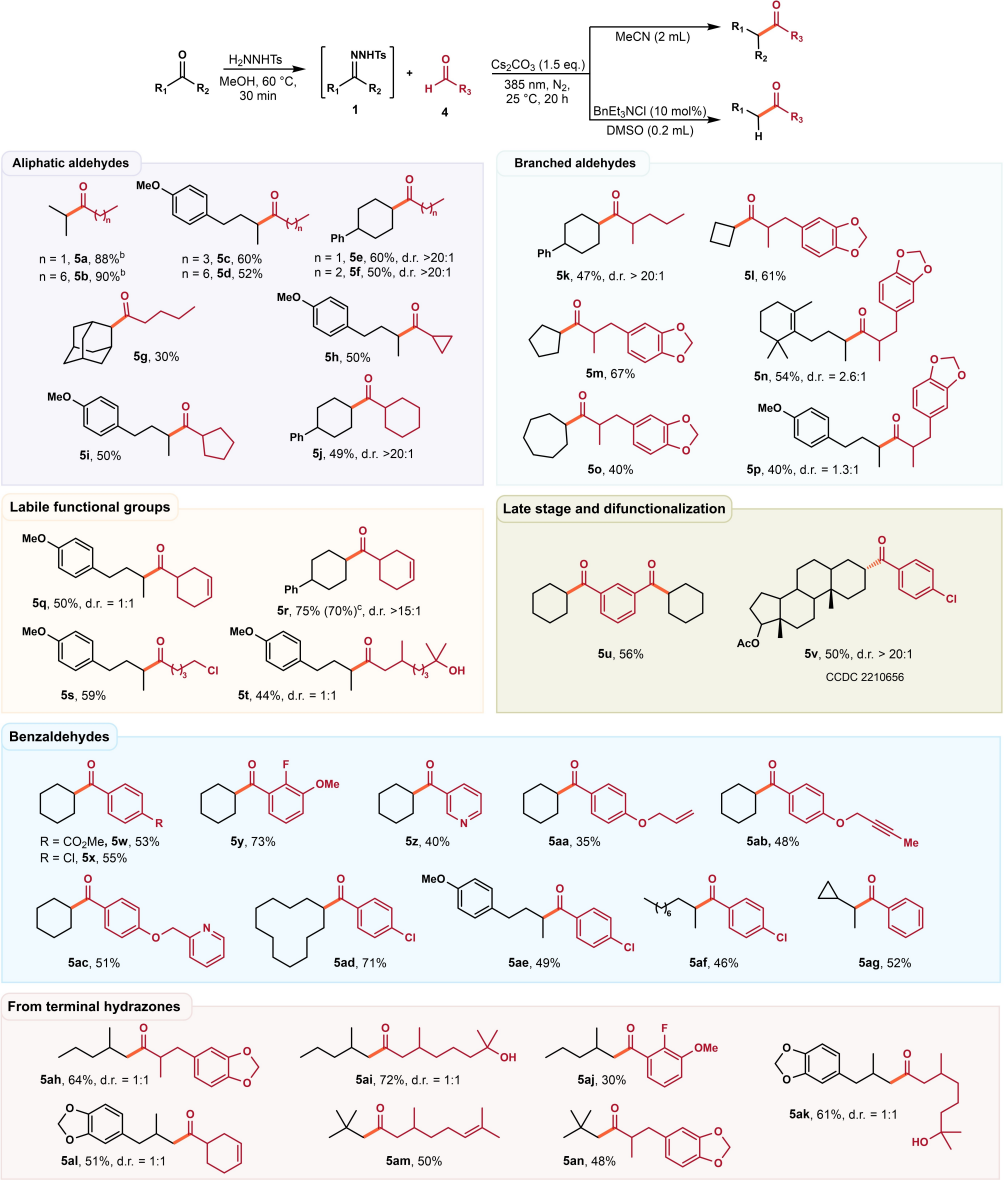
Scope of carbonyl homologation to access ketones.

[a] Reaction conditions: **1** (0.2 mmol, 1.0 equiv), **4** (0.24 mmol, 1.2 equiv), and Cs_2_CO_3_ (0.3 mmol, 1.5 equiv) in MeCN (2 mL), irradiation with a 385 nm LED (0.5 W) at 25 °C under N_2_. Yields of isolated products are given. [b] GC yield by using mesitylene as an internal standard. [c] 6 mmol scale.

**Scheme 2:**
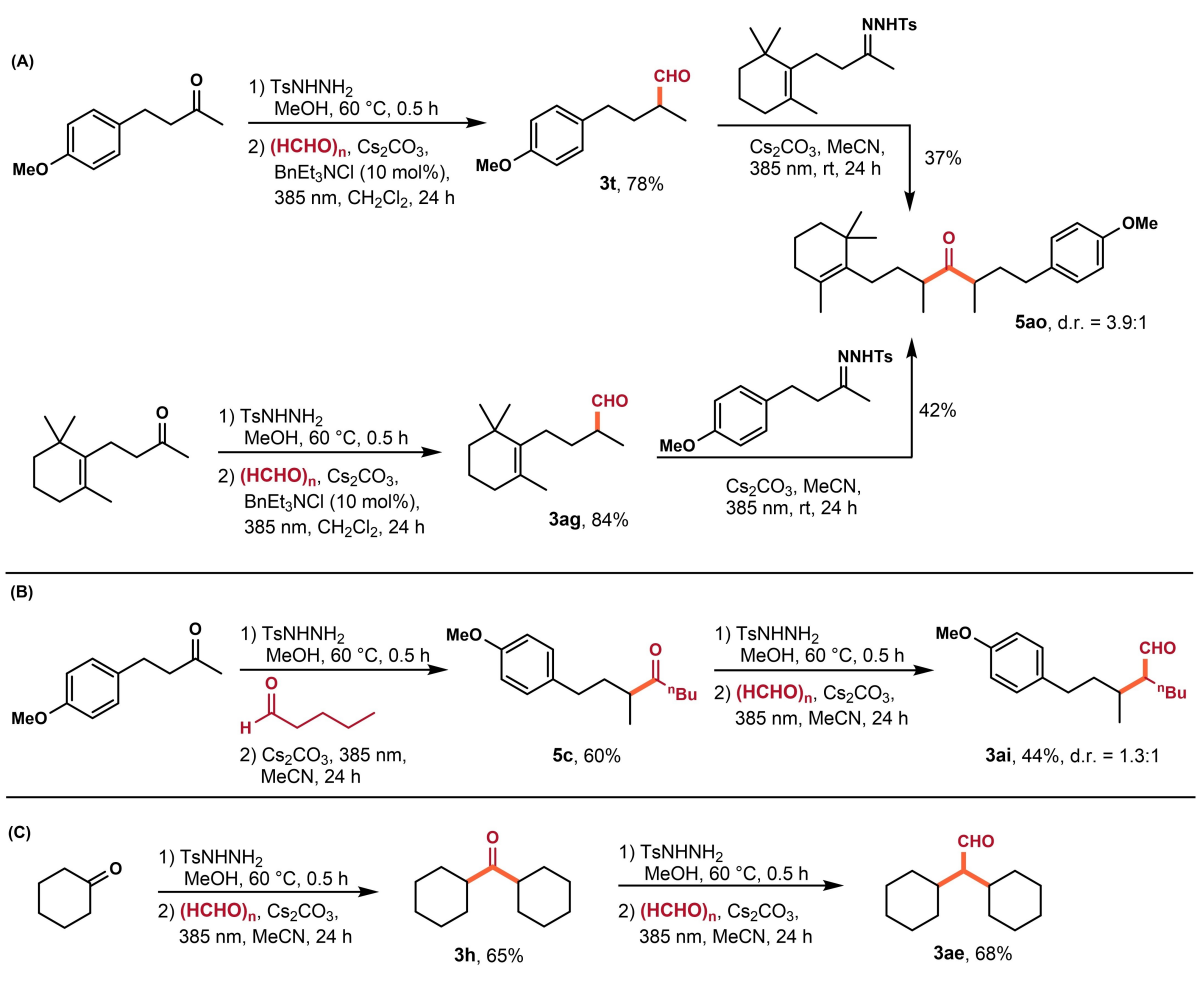
Sequential reaction process and synthetic applications.

Having established a facile access to aldehydes and ketones, we next applied our newly developed method in the sequential synthesis of carbonyl compounds (Scheme 2). The iterative synthesis process was applied for rapid assembly of a densely substituted functionalized ketone **5** **ao** from easily available starting materials by two different routes (Scheme 2A). Besides, our methods also enabled the rapid construction of aldehyde **3** **ai**, thus providing a template for the programmed construction of highly substituted aldehyde (Scheme 2B). Finally, we realized a gram‐scale synthesis of sterically hindered aldehyde **3** **ae** starting from cyclohexanone and paraformaldehyde via two consecutive iteration cycles (Scheme 2C).

**Scheme 3:**
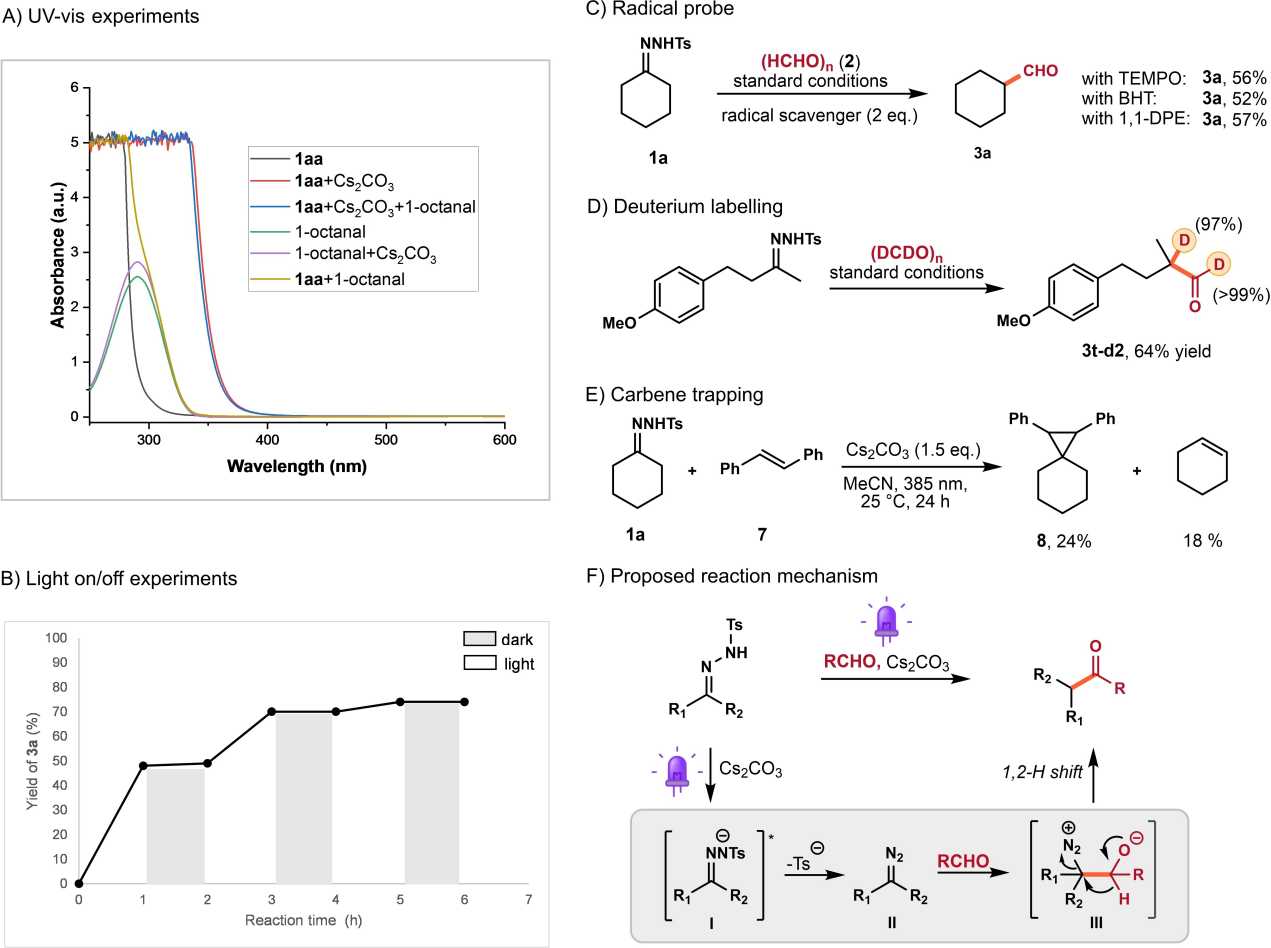
Mechanistic studies and mechanism proposal.

To gain mechanistic insight into this reaction, a series of control experiments and spectroscopic investigations were conducted. Considering the poor solubility of paraformaldehyde, we chose a reaction mixture of acetone‐derived tosylhydrazone **1** **aa**, dodecanal, and Cs_2_CO_3_ in MeCN for the UV/Vis absorption measurements (Scheme 3A). We found that tosylhydrazone **1** **aa**, dodecanal, Cs_2_CO_3_ separately, as well as the combination of **1** **aa** and dodecanal, showed the absorption exclusively in the UV (<350 nm) region. As aforementioned, we observed a clear bathochromic shift by ≈50 nm upon addition of Cs_2_CO_3_ (1.0 equiv), further addition of aldehyde in this mixture did not show any significant change in the absorption spectrum (Scheme 3A and Figure S9). These results suggest that the formation of a charge transfer aggregate between tosylhydrazone and aldehyde is unlikely in our reaction. Next, we found continuous light irradiation is essential for the reaction to proceed (Scheme 3B). Radical trapping experiments with TEMPO, BHT or, 1,1‐diphenylethylene afforded the desired aldehyde in good yields, thus rendering radicals as key intermediates of the reaction less likely (Scheme 3C). The use of deuterated paraformaldehyde delivered deuterated aldehyde **3** **t‐d2** with high levels of deuterium incorporation at both formyl and α‐carbonyl positions. Furthermore, replacing the aldehyde with 1.5 equiv of *E*‐stilbene afforded the corresponding cyclopropanation product **8** in 24 % yield and cyclohexene in 18 % yield (Scheme 3E). It is well‐known that diazo species would undergo photolysis to produce carbene intermediates under light irradiation.^[24]^ We therefore propose diazo compounds as key intermediates in our reaction, while denitrogenation and Bamford–Stevens processes occur in absence of aldehydes as trapping electrophiles. We observed that tosylhydrazone **1** **a** was with low conversion (<5 %) in the absence of light irradiation (Table 1, entry 7), thus excluding the direct base‐mediated alkyl diazo formation from the corresponding tosylhydrazone.[^16^, ^19a^] Based on the above observations and literature reports, we propose a rational reaction mechanism in Scheme 3F. The excited state of anion **I** is formed by deprotonation of *N*‐tosylhydrazone by Cs_2_CO_3_ and light irradiation, followed by extrusion of a tosylate **I** to produce the diazo intermediate **II**. Subsequently, **II** undergoes nucleophilic attack to form a diazonium alkoxide **III**, from which a 1,2‐hydride shift occurs to generate the product.

## Conclusion

In summary, we have developed a photochemical approach to access highly reactive alkyl diazo intermediates, which insert into C−H bonds of aldehydes and paraformaldehyde yielding ketones and homologated aldehydes. By taking advantage of the unique activity of photo‐excited N‐tosylhydrazone anions, highly reactive alkyl diazo intermediates were efficiently produced and transformed in situ. As showcased in Tables 2 and 3 and Scheme 2, this operationally simple protocol enables the facile construction of both aldehydes and ketones with broad substrate scope and high functional group tolerance. The synthetic utility of this method has been demonstrated in the synthesis and derivatization of biologically relevant compounds, and the iterative assembly of densely functionalized carbonyl compounds. Further studies on the trapping of photo‐generated alkyl diazo species with other agents, use of the method in natural product synthesis and transfer to an automated iterative synthesis are underway in our laboratory.

## Experimental Section

Essential Experimental Procedures/Data are included in the Supporting Information.

## Conflict of interest

The authors declare no conflict of interest.

1

## Supporting information

As a service to our authors and readers, this journal provides supporting information supplied by the authors. Such materials are peer reviewed and may be re‐organized for online delivery, but are not copy‐edited or typeset. Technical support issues arising from supporting information (other than missing files) should be addressed to the authors.

Supporting Information

## Data Availability

The data that support the findings of this study are available from the corresponding author upon reasonable request.
