# Supplementary material for: Photo‐Induced Homologation of Carbonyl Compounds for Iterative Syntheses
Source: Angew Chem Int Ed Engl. 2022 Nov 10;61(49):e202211578. doi: 10.1002/anie.202211578 (PMC10099875; doi:10.1002/anie.202211578)
Supplement: Supplementary file 1 — Supporting Information [file ANIE-61-0-s001.pdf]

Angewandte Chemie
Eine Zeitschrift der Gesellschaft Deutscher Chemiker

Supporting Information

Photo-Induced Homologation of Carbonyl Compounds for Iterative Syntheses

*H. Wang, S. Wang, V. George, G. Llorente, B. König**

Supporting Information

Photo-Induced Homologation of Carbonyl Compounds for Iterative Syntheses

Hua Wang[#a,b], Shun Wang[#a], Vincent George[#a], Gadler Llorente[a] and Burkhard König*[a]
[#]Equal contribution
[a]Institute of Organic Chemistry, Faculty of Chemistry and Pharmacy, University of Regensburg, D-93040 Regensburg, Germany
[b]Department of Chemistry, School of Pharmacy, The Fourth Military Medical University, Xi'an 710032, P.R.China

burkhard.koenig@ur.de

Contents

1. General information S2

2. Starting materials S4

3. Experimental procedures S6

4. Optimization details for the reaction conditions S7

5. Mechanistic studies S13

6. Characterization of compounds S19

7. X-ray characterization data S44

8. Copies of NMR spectra S46

9. GC spectra S135

10. References S138

1. General information

All NMR spectra were recorded at room temperature using a Bruker Avance 300 (300 MHz for ^{1}H, 75 MHz for ^{13}C), or a Bruker Avance 400 (400 MHz for ^{1}H, 101 MHz for ^{13}C) NMR spectrometer.[1] All chemical shifts are reported in δ-scale as parts per million [ppm] (multiplicity, coupling constant *J*, number of protons) relative to the solvent residual peaks as the internal standard.[2] Coupling constants *J* are given in Hertz [Hz]. Abbreviations used for signal multiplicity: ^{1}H-NMR: br = broad, s = singlet, d = doublet, t = triplet, q = quartet, dd = doublet of doublets, dt = doublet of triplets, and m = multiplet. High resolution mass spectra (HRMS) were obtained from the central analytic mass spectrometry facilities of the Faculty of Chemistry and Pharmacy, Regensburg University, and are reported according to the IUPAC recommendations 2013. All mass spectra were recorded on a Finnigan MAT 95, Thermo Quest Finnigan TSQ 7000, Finnigan MATSSQ 710 A or an Agilent Q-TOF 6540 UHD instrument. GC measurements were performed on a GC 7890 from Agilent Technologies. Data acquisition and evaluation were done with Agilent ChemStation Rev.C.01.04. [35]. Analytical TLC was performed on silica gel coated alumina plates (MN TLC sheets ALUGRAM® Xtra SIL G/UV254). Visualization was done by UV light (254 or 366 nm). If necessary, potassium permanganate was used for chemical staining. Purification by column chromatography was performed with silica gel 60 M (40-63 µm, 230-440 mesh, Merck) on a Biotage® Isolera TM Spektra One device. Photocatalytic reactions were performed with 385 nm LEDs (OSRAM Oslon SSL 80 royal-blue LEDs (λ = 385 nm (± 15 nm), 21 V, 700 mA). The sample was irradiated with a LED through the vial's plane bottom side and cooled from the side using custom-made aluminum cooling blocks connected to a thermostat (Figure S1). Gram-scale reactions were in a classic glass tube photochemical reactor setup irradiated from the outside (Figure S2). The glass tube with reaction mixture and LED cooling block were thermostated at 25 °C. UV–Vis and fluorescence measurements were performed with a Varian Cary 100 UV/Vis spectrophotometer and FluoroMax-4 spectrofluorometer, respectively. Commercially available starting materials and solvents were used without further purification.

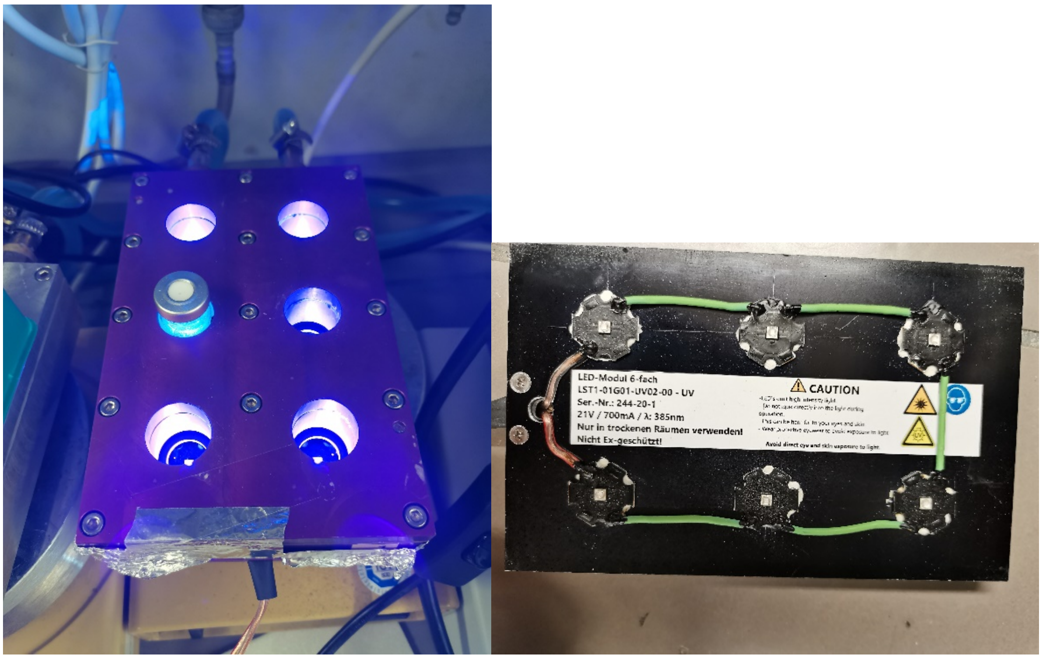

Figure S1. Photochemical set-up for regular-scale reactions

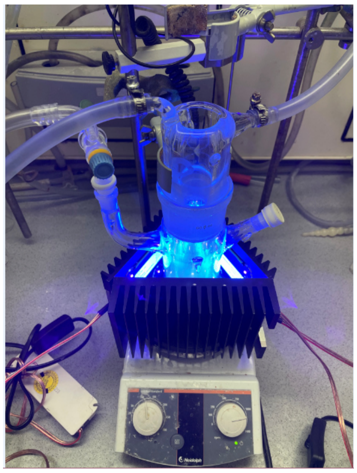

Figure S2. Photochemical set-up for large-scale reactions

2. Starting materials

2.1 Synthesis of N-tosylhydrazones from ketones[1]

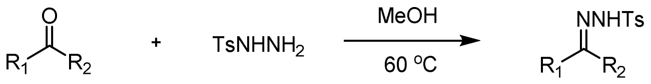

General procedure A-SM

To a stirred solution of tosylhydrazide (10 mmol) in MeOH (10 mL) at 60 °C, aldehyde or ketone (1 eq.) was added dropwise (or portionwise if solid). The reaction progress was monitored by TLC. After the completion of reaction, the reaction mixture was cooled and filtered to remove the solvent. The filtered solid was triturated with hexane/diethyl ether to get the pure N-sulfonylhydrazones.

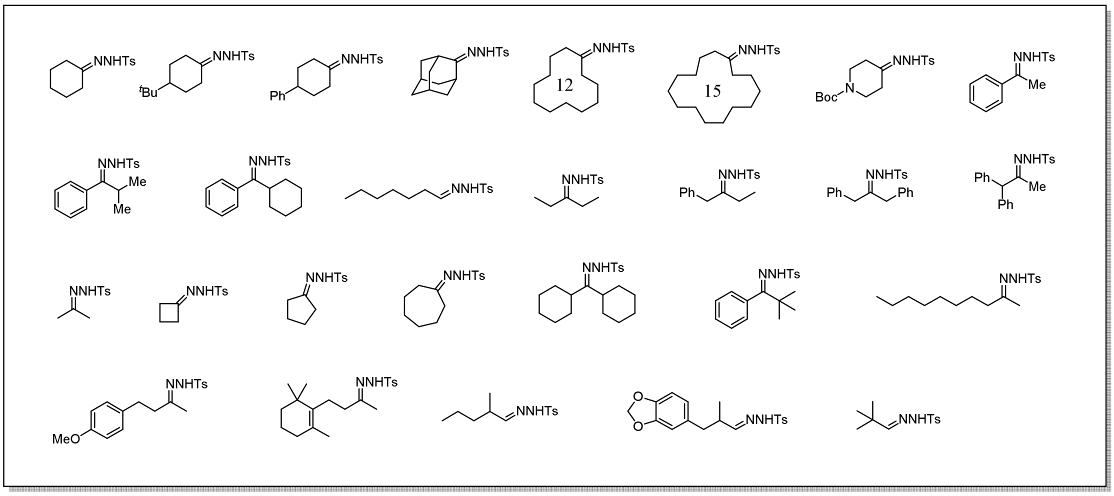

General procedure B-SM

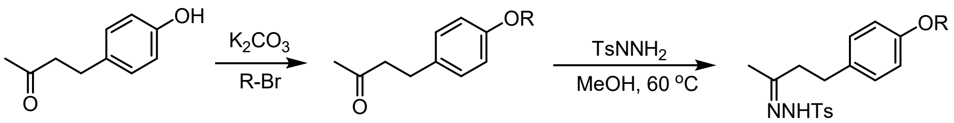

Step 1: Alkyl Bromides (3.15 mmol, 1.05 equiv.) and K_2CO_3 (4.5 mmol, 1.50 equiv.) were added to a solution of 4-(4-hydroxyphenyl)butan-2-one (3.0 mmol, 1.0 equiv.) in DMF (8.0 mL) in a 20-mL vial under air condition. After stirring at room temperature overnight, the reaction mixture was quenched with water (10 mL), before EtOAc (30 mL) was added. The organic phase was washed with water (3 × 20 mL) and brine (30 mL), and then dried over $MgSO_4$, filtered, and concentrated in vacuo. Then the crude product was used directly for next step without further purification.

Step 2: To a stirred solution of tosylhydrazide (3 mmol) in MeOH (10 mL) at 60 °C, ketone (1 eq.) from last step was added dropwise (or portionwise if solid). The solution was refluxed at 65 °C for around 30 min along with white solid crashes out, and the reaction was monitored by TLC until the complete consumption of both starting materials. The crude mixture was then purified by flash column chromatography (10% to 50%, EtOAc in petroleum ether) on silica gel.

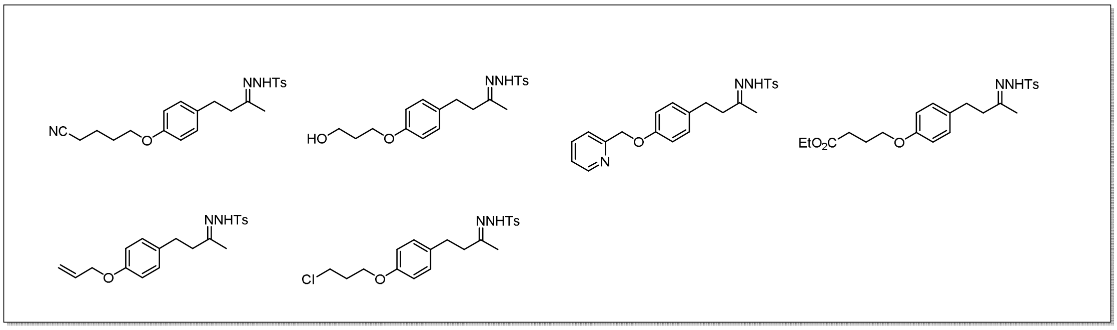

General procedure C-SM

Tosylhydrazide (0.2 mmol), aldehyde or ketone (0.2 mmol) were dissolved in MeOH (2 mL) at 60 °C. The reaction progress was monitored by TLC. After the completion of the reaction, the reaction mixture was concentrated in vacuo and directly used in the photochemical reaction.

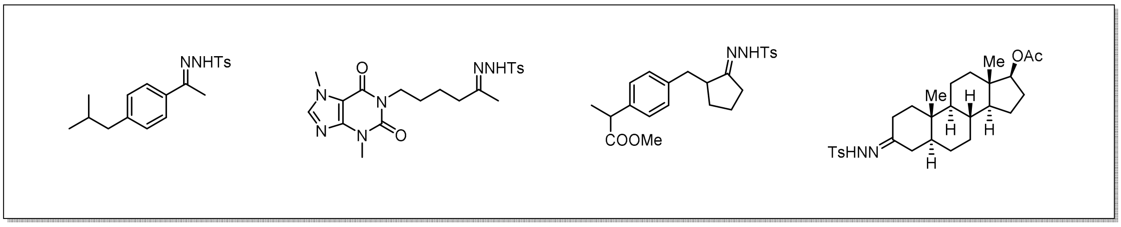

2.2 Synthesis of aldehydes

General procedure D-SM

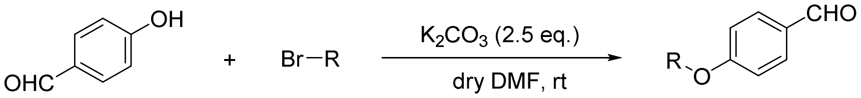

Alkyl Bromides (3.15 mmol, 1.05 equiv.) and K_2CO_3 (4.5 mmol, 1.50 equiv.) were added to a solution of 4-hydroxybenzaldehyde (0.37 g, 3.0 mmol, 1.0 equiv.) in DMF (8.0 mL) in a 20-mL vial under air condition. After stirring at room temperature overnight, the reaction mixture was quenched with water (10 mL), before EtOAc (30 mL) was added. The organic phase was washed with water (3 × 20 mL) and brine (30 mL), and then dried over $MgSO_4$, filtered, and concentrated in vacuo. Then the crude product was purified by flash column chromatography (1% to 3%, EtOAc in petroleum ether) on silica gel to afford the product.

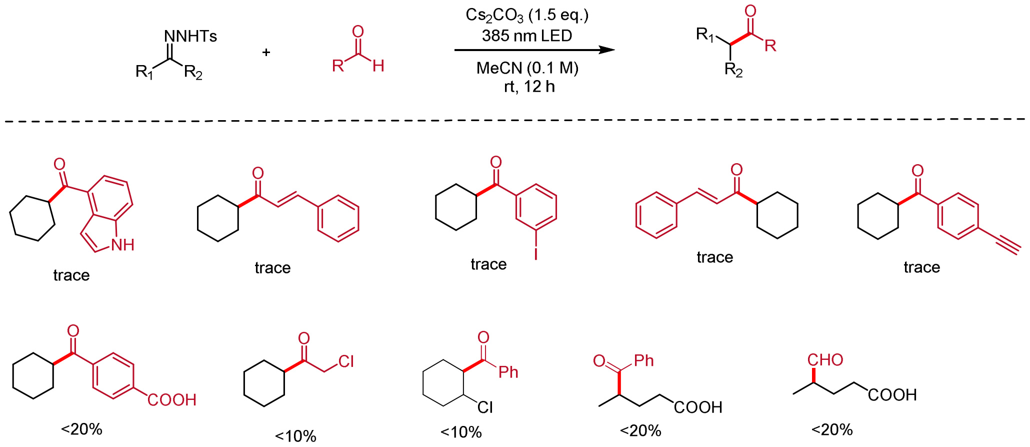

Scheme S1. List of unsuccessful substrates

3. Experimental procedures

3.1 Synthesis of aldehydes from paraformaldehyde with N-tosylhydrazones

General procedure A

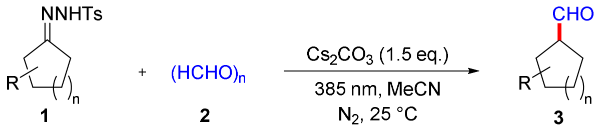

To a 5 mL snap vial with a magnetic stirring bar, tosylhydrazones (**1**, 0.2 mmol, 1.0 eq.), Cs_2CO_3 (0.3 mmol, 1.5 eq.), and paraformaldehyde (**2,** 0.24 mmol, 1.2 eq.) were added. The vial was evacuated and backfilled with N_2 three times. Then dry MeCN (2 mL) was added by syringe, and the mixture was irradiated with a 385 nm LED at 25 °C. After 20 h, the mixture was quenched with water and extracted with EtOAc or Et_2O (3 x 10 mL). The combined organic phase was then washed with H_2O (10 mL) and brine, dried over sodium sulfate, concentrated under vacuum. The residue was purified by silica gel flash chromatography (gradient eluent: petroleum ether in EtOAc =1% to 2%, or pentane in diethyl ether = 2% to 3%) to give the desired product.

General procedure B

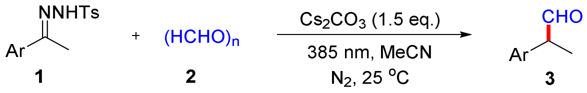

To a 5 mL snap vial with a magnetic stirring bar, tosylhydrazones (**1**, 0.4 mmol, 2.0 eq.), Cs_2CO_3 (0.3 mmol, 1.5 eq.), and paraformaldehyde (**2,** 0.2 mmol, 1.0 eq.) were added. The vial was evacuated and back filled with N_2 for three times. Then dry MeCN (2 mL) was added by syringe, and the mixture was irradiated with a 385 nm LED at 25 °C. After 20 h, the mixture was quenched with water and extracted with Et_2O (10 mL *3). The combined organic phase was then washed with H_2O (10 mL) and brine, dried over sodium sulfate, concentrated under vacuum. The residue was purified by silica gel flash chromatography (gradient eluent: pentane in diethyl ether = 2% to 3%) to give the desired product.

General procedure C

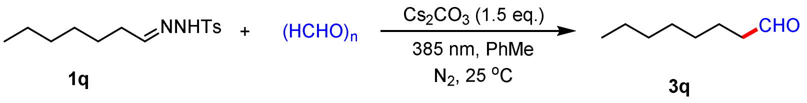

To a 5 mL snap vial with a magnetic stirring bar, tosylhydrazones (**1q**, 0.3 mmol, 1.5 eq.), Cs_2CO_3 (0.3 mmol, 1.5 eq.), and paraformaldehyde (**2,** 0.20 mmol, 1.0 eq.) were added. The vial was evacuated and back filled with N_2 for three times. Then dry toluene (2 mL) was added by syringe, and the mixture was irradiated with a 385 nm LED at 25 °C. After 20 h, the reaction mixture was diluted with EA (3 mL), dodecane was added as an internal standard, followed by water. After shaking for 1 min, the organic layer was separated, and the yield was determined by GC. *Note: we observed that tosylhydrazone **1q** decomposed considerably at ambient conditions after several days, and therefore this substrate should be freshly prepared before use.*

General procedure D

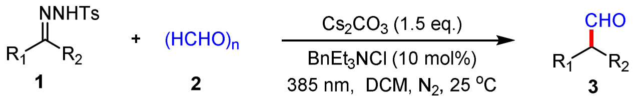

To a 5 mL snap vial with a magnetic stirring bar, tosylhydrazones (**1**, 0.2 mmol, 1.0 eq.), Cs_2CO_3 (0.3 mmol, 1.5 eq.), $BnEt_3NCl$ (20.0 µmol, 10 mol%) and paraformaldehyde (**2,** 0.2 mmol, 1.0 eq.) were added. The vial was evacuated and back filled with N_2 for three times. Then dry CH_2Cl_2 (2 mL) was added by syringe, and the mixture was irradiated with a 385 nm LED at 25 °C. After 20 h, the mixture was quenched with water and extracted with CH_2Cl_2 (10 mL *3). The combined organic phase was then washed with H_2O (10 mL) and brine, dried over sodium sulfate, concentrated under vacuum. The residue was purified by silica gel flash chromatography to give the desired product.

General procedure E

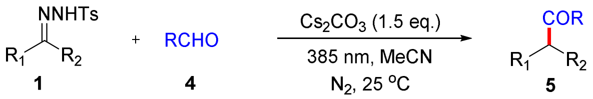

To a 5 mL snap vial with a magnetic stirring bar, tosylhydrazones (**1**, 0.2 mmol, 1.0 eq.), Cs_2CO_3 (0.3 mmol, 1.5 eq.), and aldehyde (**4,** 0.24 mmol, 1.2 eq.) were added. The vial was evacuated and back filled with N_2 for three times. Then dry MeCN (2 mL) was added by syringe, and the mixture was irradiated with a 385 nm LED at 25 °C. After 20 h, the mixture was quenched with water and extracted with Et_2O (10 mL *3). The combined organic phase was then washed with H2O (10 mL) and brine, dried over sodium sulfate, concentrated under vacuum. The residue was purified by silica gel flash chromatography (gradient eluent: pentane in diethyl ether = 2% to 3%) to give the desired product.

General procedure F

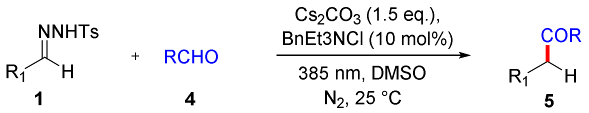

To a 5 mL snap vial with a magnetic stirring bar, tosylhydrazones (**1**, 240 µmol, 1.2 eq.), Cs_2CO_3 (300 µmol, 1.5 eq.), $BnEt_3NCl$ (20.0 µmol, 10 mol%), and aldehyde (**4,** 200 µmol, 1.0 eq., if solid) were added. The vial was evacuated and back filled with N_2 for three times. Then dry DMSO (200 µL) and aldehyde (**4,** 200 µmol, 1.0 eq., if liquid) were added by syringe, and the mixture was irradiated with a 385 nm LED at 25 °C. After 20 h, the mixture was quenched with water and extracted with EtOAc (10 mL *3). The combined organic phase was then washed with brine, dried over sodium sulfate, concentrated under vacuum. The residue was purified by silica gel column chromatography to give the desired product.

4. Optimization details for the reaction conditions

4.1 Optimization details for reaction of tosylhydrazone 1a with paraformaldehyde 2

General procedure:

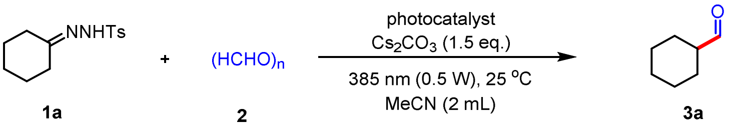

To a 5 mL snap vial with magnetic stirring bar, tosylhydrazone (**1a**, 0.2 mmol, 1.0 eq.), Cs_2CO_3 (0.3 mmol, 1.5 eq.), and paraformaldehyde (**2**, 0.3 mmol, 1.5 eq.) were added. The vial was evacuated and back filled with N_2 for three times. Then dry MeCN (2 mL) was added by syringe, and the mixture was irradiated with a 385 nm LED at 25 °C. After 20 h, the mixture was quenched with water and EtOAc. Then yields were determined by GC analysis using mesitylene as internal standard.

Table S1. Screening of photocatalysts[a]

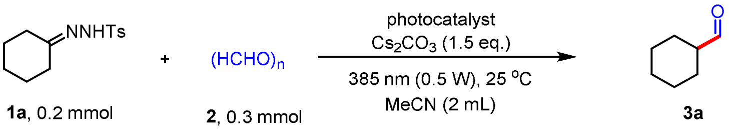

**Entry**	Photocatalyst	**Yield**
1	TBADT (0.2 mol%)	40%
2[c]	Eosin Y (5 mol%), 455 nm LED	trace
3[c]	$[Ir(dFCF_3ppy)_2dtbpy]PF_6$ (1 mol%), 455 nm LED	trace
4[c]	$Ir(ppy)_2dtbpyPF_6$ (1 mol%), 455 nm LED	trace
5[c]	$Mes\text{-}Acr^+$ PF_6 (5 mol%), 455 nm LED	trace
6[c]	*fac*-$Ir(ppy)_3$ (1 mol%), 455 nm LED	22%
**7**	**-**	**70%**

[a] Reaction conditions: Unless otherwise noted, all reactions were carried out with **1a** (0.2 mmol), **2** (0.3 mmol), Cs_2CO_3 (0.3 mmol) and photocatalyst in 2 mL MeCN, irradiation with a 385 nm LED (0.5 W) at 25 °C for 20 hours. [b] Yields were determined by GC analysis using mesitylene as an internal standard. [c] Irradiation with a 455 nm LED (0.5 W).

Table S2. Screening of bases[a]

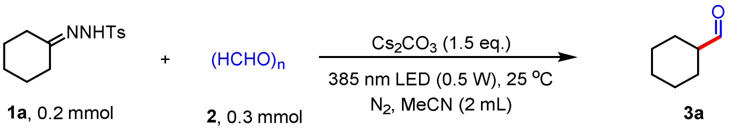

**Entry**	**Base**	**Yield**[b]
1	K_2CO_3	n.d.
2	Na_2CO_3	n.d.

3	$NaHCO_3$	n.d.
4	K_3PO_4	n.d.
5[c]	CsOAc	trace
6	DBU	trace
7	Cs_2CO_3 (1.0 eq.)	63%
8	Cs_2CO_3 (2.0 eq.)	68%
9	-	n.d.

[a] Reaction conditions: Unless otherwise noted, all reactions were carried out with **1a** (0.2 mmol), **2** (0.3 mmol), Cs_2CO_3 (0.3 mmol) and photocatalyst in 2 mL of MeCN, irradiation with a 385 nm LED at 25 °C for 20 hours, n.d. = not detected. [b] Yields were determined by GC analysis using mesitylene as an internal standard. [c] Cyclohexyl acetate was detected in a 48% yield.

Table S3. Screening of solvents[a]

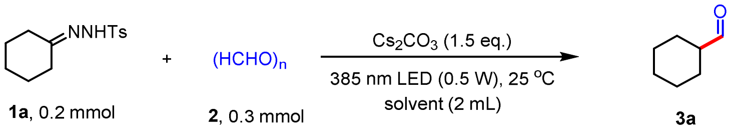

Entry	Solvent	Yield
1	DMF	20%
2	DMSO	5%
3	toluene	50%
4	dioxane	36%
5	THF	20%
6	$PhCF_3$	48%
7	PhCl	33%

[a] Reaction conditions: Unless otherwise noted, all reactions were carried out with **1a** (0.2 mmol), **2** (0.3 mmol), Cs_2CO_3 (0.3 mmol) and photocatalyst in 2 mL of solvent, irradiation with a 385 nm LED (0.5 W) at 25 °C for 20 hours. [b] Yields were determined by GC analysis using mesitylene as internal standard.

Table S4. Screening of light sources[a]

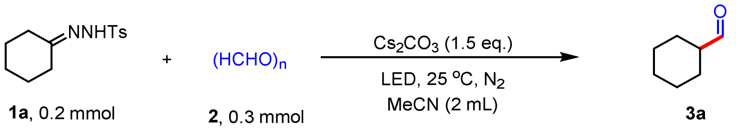

Entry	Light source	Yield
1	365 nm LED (0.5 W)	65%

2	400 nm LED (0.5 W)	trace
3	385 nm LED (3 W)	68%
4	455 nm LED (0.5 W)	n.d.
5	In the dark	n.d.
6[b]	In the dark and heating to 110 °C	n.d.

[a] Reaction conditions: Unless otherwise noted, all reactions were carried out with **1a** (0.2 mmol), **2** (0.3 mmol), Cs_2CO_3 (0.3 mmol) and photocatalyst in 2 mL MeCN, irradiation with a 385 nm LED at 25 °C for 20 hours. Yields were determined by GC analysis using mesitylene as the internal standard. [b] Cyclohexene was detected as the major product.

Table S5. Screening of ratio of **1a** with **2**[a]

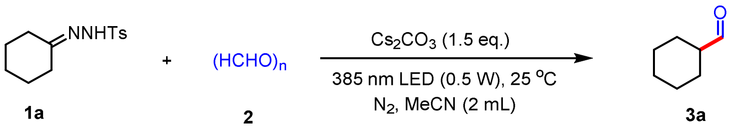

Entry	1a : 2	Yield[b]
**1**	**1:1.2**	**92%**
2	1:1	78%
3	1:2	90%
4[c]	1:1.2	68%

[a] Reaction conditions: Unless otherwise noted, all reactions were carried out with **1a** (0.2 mmol), **2** (0.3 mmol), Cs_2CO_3 (0.3 mmol) and photocatalyst in 2 mL of MeCN, irradiation with a 385 nm LED at 25 °C for 20 hours. [b]Yields were determined by GC analysis using mesitylene as internal standard. [c] Irradiation with a 385 nm LED (3 W).

4.2 Optimization details for reaction of tosylhydrazone 1j with paraformaldehyde 2

Table S6. Screening of reaction parameters[a]

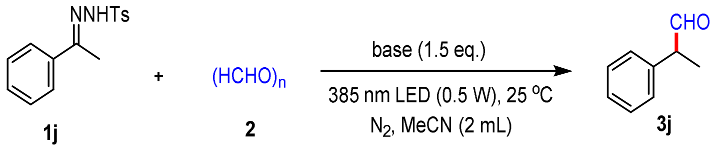

Entry	Solvent	Base	Yield
1	MeCN	Cs_2CO_3	5%
2	DMSO	Cs_2CO_3	trace
3	DMF	Cs_2CO_3	trace
4	DMAc	Cs_2CO_3	10%
5	dioxane	Cs_2CO_3	trace

6	toluene	Cs_2CO_3	3%
7	MeCN	K_2CO_3	n.d.
8	MeCN	Na_2CO_3	n.d.
9[c]	MeCN	CsOAc	trace
10[d]	MeCN	Cs_2CO_3	7%
11[e]	MeCN	Cs_2CO_3	43%
12[f]	MeCN	Cs_2CO_3	**86%**

[a] Reaction conditions: Unless otherwise noted, all reactions were carried out with **1j** (0.2 mmol), **2** (0.24 mmol), Cs_2CO_3 (0.3 mmol) and photocatalyst in 2 mL of solvent, irradiation with a 385 nm LED (0.5 W) at 25 °C for 20 hours. [b] Yields were determined by GC analysis using mesitylene as the internal standard. [c] 1-phenylethyl acetate was formed in a 65% yield. [d] **1j** (0.24 mmol, 1.2 eq.), **2** (0.20 mmol, 1.0 eq.). [e] **1j** (0.3 mmol, 1.5 eq.), **2** (0.20 mmol, 1.0 eq.). [f] **1j** (0.4 mmol, 2.0 eq.), **2** (0.20 mmol, 1.0 eq.).

4.3 Optimization details for reaction of tosylhydrazone 1q with paraformaldehyde 2

Table S7. Screening of reaction conditons[a]

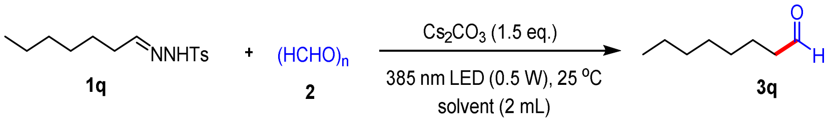

Entry	Solvent	Yield
1	DMSO	trace
2	DMAc	1%
3	MeCN	trace
4	toluene	13%
5[c]	toluene	29%
6[d]	toluene	**61%**
7[e]	toluene	43%
8[e]	toluene	47%
9[d]	$PhCF_3$	50%
10[d]	DCM	49%
11[d]	benzene	36%

[a] Reaction conditions: Unless otherwise noted, all reactions were carried out with **1q** (0.2 mmol), **2** (0.24 mmol), Cs_2CO_3 (0.3 mmol) and photocatalyst in 2 mL of solvent, irradiation with a 385 nm LED (0.5 W) at 25 °C for 20 hours. [b] Yields were determined by GC analysis using *n*-dodecane as internal standard. [c] **1q** (0.24 mmol, 1.2 eq.), **2** (0.20 mmol, 1.0 eq.). [d] **1q** (0.3 mmol, 1.5 eq.), **2** (0.20 mmol, 1.0 eq.). [e] **1q** (0.4 mmol, 2.0 eq.), **2** (0.20 mmol, 1.0 eq.).

Table S8. Screening of reaction conditions

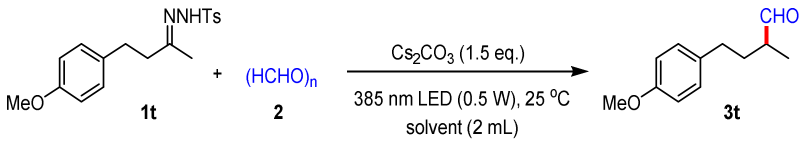

Entry	Solvent	Eq. of 2	Additives / changes	Yield
1	MeCN	1.2		50%
2	MTBE	1.2		33%
3	$(CH_2Cl)_2$	1.2		59%
4	$(CH_2Cl)_2$	1.0		67%
5	CH_2Cl_2	1.2		66%
6	CH_2Cl_2	1.0		70%
7	CH_2Cl_2	1.2	$BnEt_3NCl$ (10 mol%)	71%
**8**[b]	**CH_2Cl_2**	**1.0**	**$BnEt_3NCl$ (10 mol%)**	**78%**
9	CH_2Cl_2	1.2	$BnEt_3NCl$ (20 mol%)	66%
10	CH_2Cl_2	1.0	$BnEt_3NCl$ (20 mol%)	71%
11	$(CH_2Cl)_2$	1.0	Heating to 60 °C (dark)	n.d.

[a] Reaction conditions: Unless otherwise noted, all reactions were carried out with **1t** (0.2 mmol, 1.0 eq.), **2**, Cs_2CO_3 (0.3 mmol, 1.5 eq.) in 2 mL of solvent, irradiation with a 385 nm LED (0.5 W) at 25 °C for 20 hours. Yields of isolated products are given. [b] Epoxide **3t–1** was isolated in a 16% yield.

5. Mechanistic studies

Reaction profile and on/off Experiments

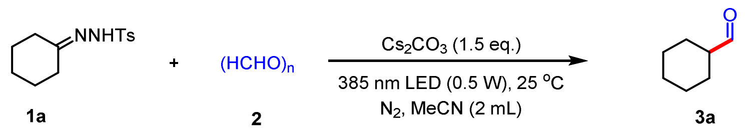

Procedure: Tosylhydrazone **1a** (0.2 mmol, 53.2 mg), Cs_2CO_3 (0.3 mmol, 98 mg), **2** (0.24 mmol, 7.2 mg), and internal standard (dodecane, 20 uL) were added into a 5 mL snap vial equipped with a stirring bar. The vial was evacuated and back filled with N_2 for three times, followed by the addition of MeCN (2 mL) *via* syringe. Then the reaction mixture was irradiated by a 385 nm LED (0.5 W) at 25 °C. An aliquot of the reaction mixture was then taken at the indicated times and analyzed by GC-FID.

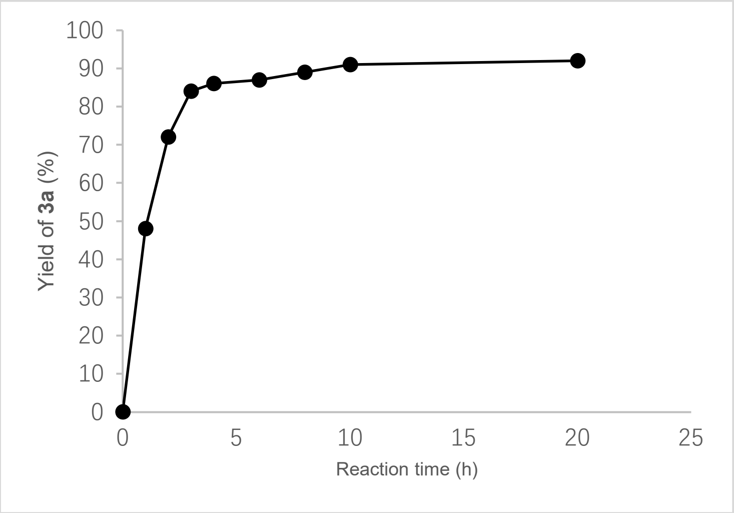

Figure S3. Reaction profile plot

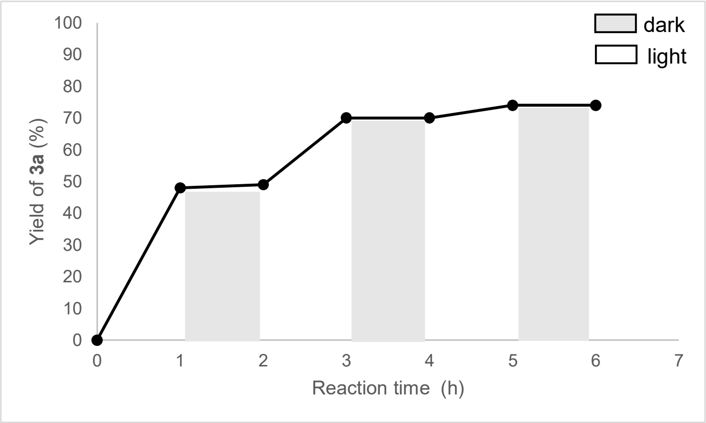

Figure S4. On/off experiments

Radical probing and deuterium labelling experiments

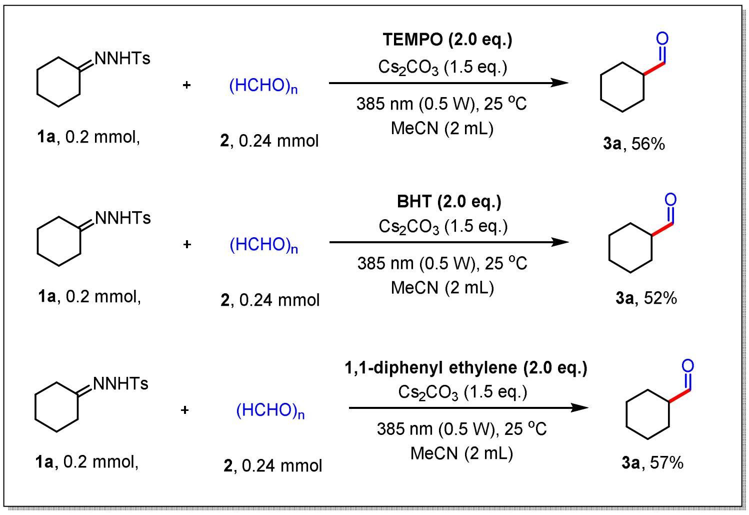

Scheme S2. Radical probing experiments

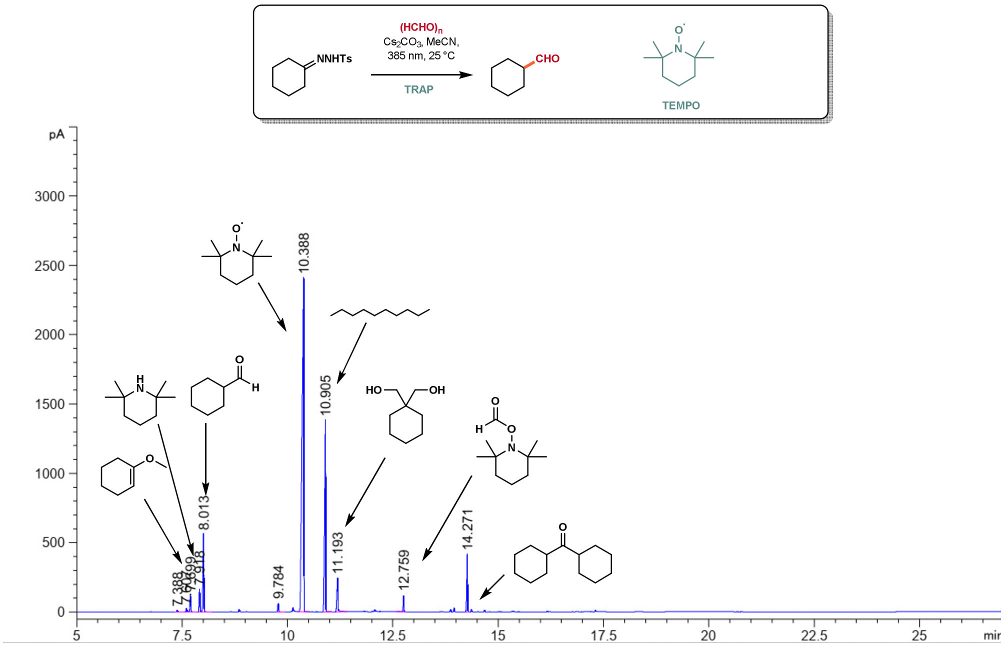

Figure S5. GCMS spectrum of the crude reaction mixture in the presence of TEMPO (2.0 eq.) after light irradiation (385 nm LED). Results: 93% of TEMPO is left unreacted. No radical trapping products can be observed, only small amounts of deoxygenated and formylated products originated from TEMPO were detected. The addition of TEMPO led to the formation of a considerable amount (~30%) of double insertion product dicyclohexyl ketone).

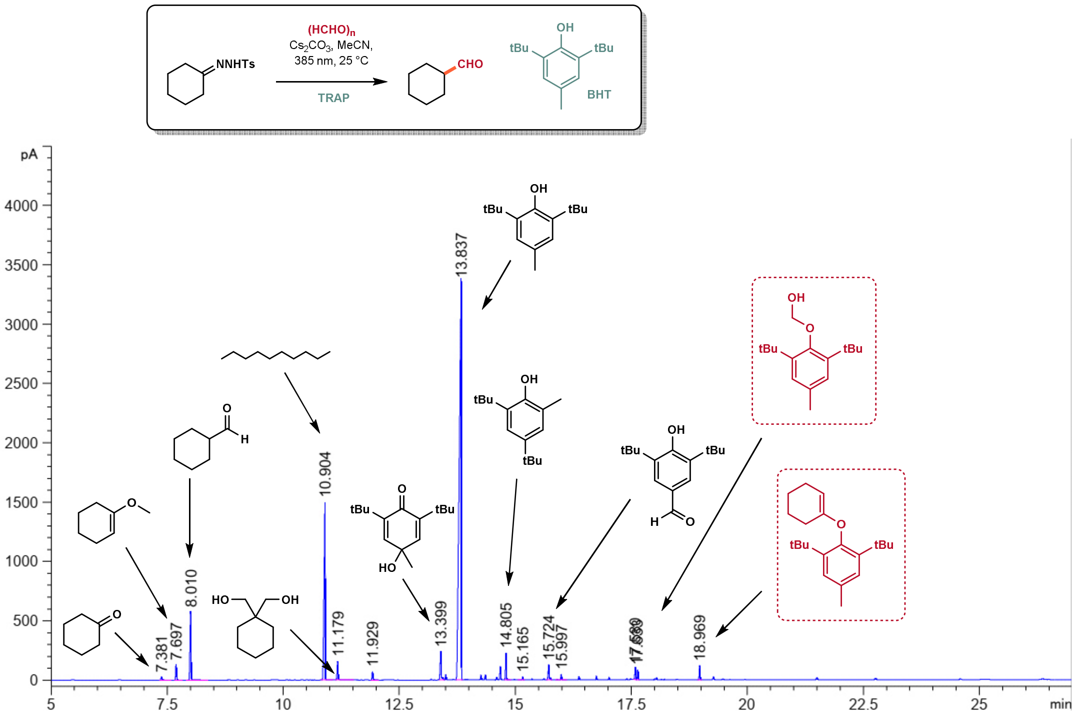

Figure S6. GCMS spectrum of the crude reaction mixture in the presence of BHT (2.0 eq.) after light irradiation (385 nm LED). Results: 75% of BHT (2.0 eq.) is left unreacted. Small amount of product originated from O-H insertion of BHT with diazo could be detected.

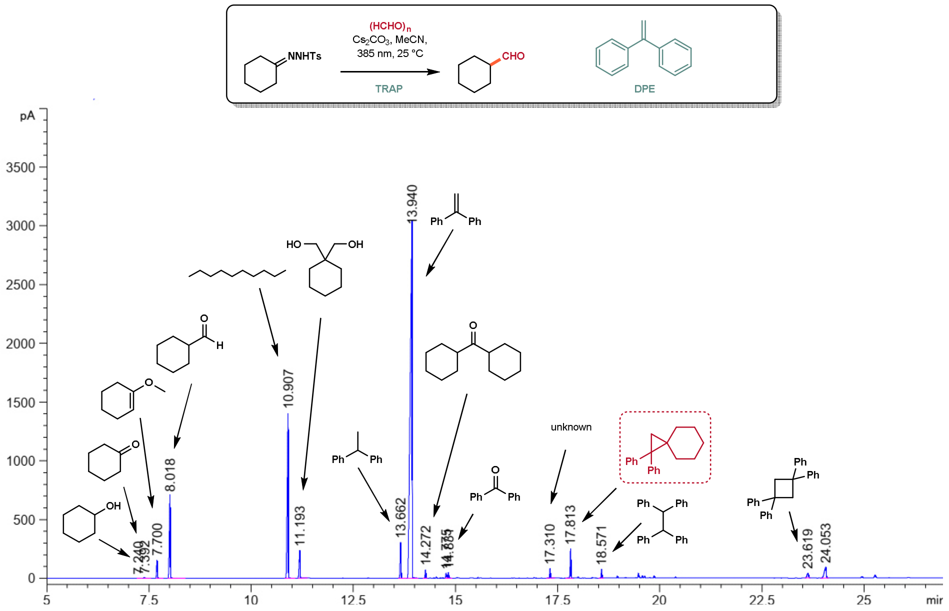

Figure S7. GCMS spectrum of the crude reaction mixture in the presence of 1,1-DPE (2.0 eq.) after light irradiation (385 nm LED). Results: 74% of 1,1-DPE (2.0 eq.) is left unreacted. A small amount of cyclopropanation product originating from carbene trapping by 1,1-DPE could be observed.

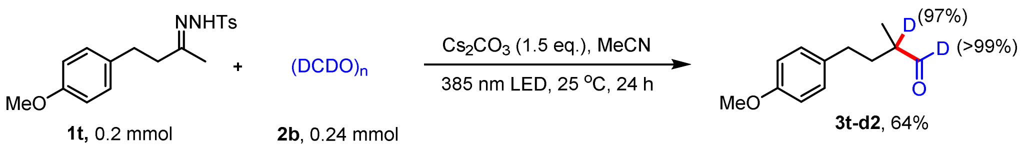

Scheme S3. Deuterium labelling experiments

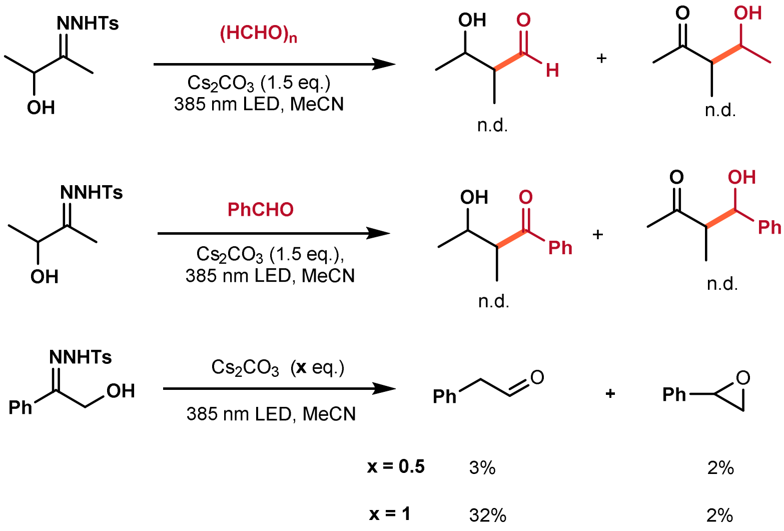

Scheme S4. Semi-pinacol rearrangement experiments. 3-hydroxybutan-2-one-derived tosylhydrazone was synthesized and subjected to the reactions with paraformaldehyde or banzaldehyde, no desired products could be detected under standard conditions. When 2-hydroxy-phenylethanone derived tosylhydrazone was employed, we detected phenylacetaldehyde and styrene oxide in 3% and 2% yield respectively in the presence of Cs_2CO_3 (0.5 eq.). Increasing the amount of Cs_2CO_3 to (1.0 eq.) led to the formation of phenylacetaldehyde in 32% yield and styrene oxide in 2% yield.

Spectroscopic investigations

Optical absorption spectra of the reaction components and their combinations were measured in MeCN. *Note:* considering the poor solubility of paraformaldehyde in MeCN, 1-octanal was selected as the substrate in the UV-vis measurements. The reaction between acetone-derived tosylhydrazone **1aa** and 1-octanal afforded the desired ketone in a 90% yield under the optimized conditions.

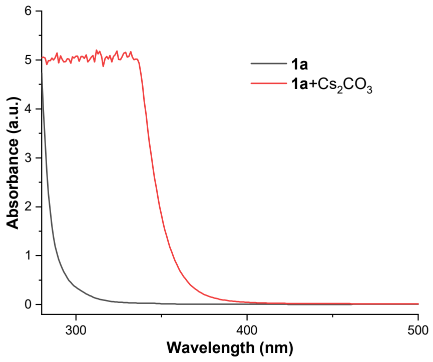

Figure S8. UV-vis spectrum of **1a** (0.1 M) in MeCN (black curve). UV-vis spectrum of **1a** (0.1 M)+Cs_2CO_3 (1.5 eq.) in MeCN (red curve).

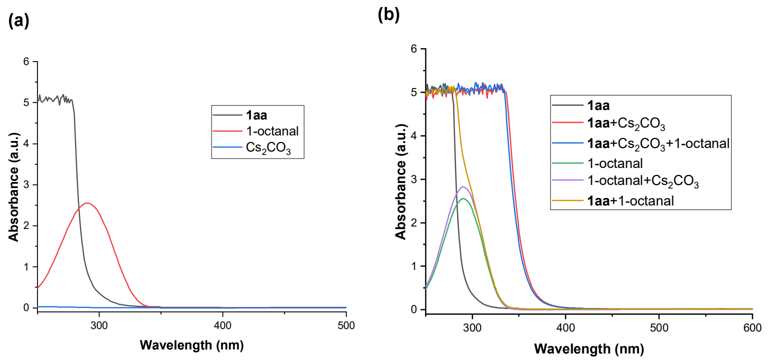

Figure S9. (a) UV-vis spectrum of acetone-derived N-tosylhydrazone **1aa** (0.1 M, black curve), 1-octanal (0.15 M, red curve), Cs_2CO_3 (sat., blue curve) in MeCN. (b) UV-vis spectrum of N-tosylhydrazone **1aa** (0.1 M, black curve), N-tosylhydrazone **1aa** (0.1 M)+Cs_2CO_3 (red curve), N-tosylhydrazone **1aa** (0.1 M)+1-octanal (0.15 M)+Cs_2CO_3 (blue curve),1-octanal (0.15 M, green curve), 1-octanal (0.15 M)+Cs_2CO_3 (purple curve), and N-tosylhydrazone **1aa** (0.1 M)+1-octanal (0.15 M) (brown curve) in MeCN.

Scheme S5. Trapping of diazo intermediates with *E*-stilbene

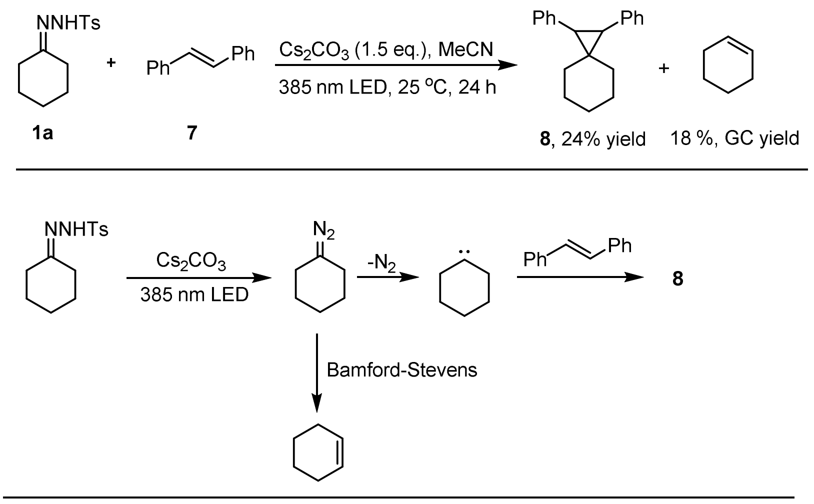

Scheme S5-1. Reaction of (diazomethylene)dibenzene with paraformaldehyde under dark conditions

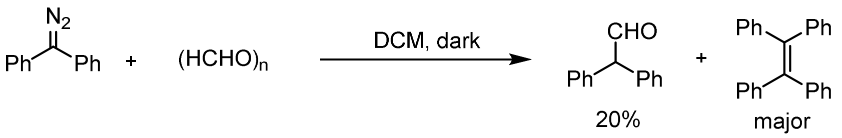

When E-stilbene was used as potential diazo trapping reagent in the reaction, the desired cyclopropanation process occurred to afford the desired product **8** in 24% yield and cyclohexene (18% yield). It is well-known that diazo species would undergo photolysis to produce carbene

intermediates under light irradiation. [2] Therefore, we propose that the diazo formed in our reaction could undergo photolysis to give carbene species and the Bamford-Stevens process to yield alkene in this case. We also showcase that the reaction between diazo and paraformaldehyde could proceed in the dark conditions (Scheme 5-1).

6. Characterization of compounds

(Z)-N'-(2,2-dimethyl-1-phenylpropylidene)-4-methylbenzenesulfonohydrazide

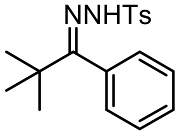

Following **General procedure A-SM** on 5 mmol scale, filtration afforded 1.57g (95%) of the title compound as a white solid. **^{1}H NMR (400 MHz, $CDCl_3$)** δ 7.77 (dd, *J* = 8.3, 1.7 Hz, 2H), 7.44 – 7.35 (m, 3H), 7.32 (d, *J* = 7.9 Hz, 2H), 6.87 (s, 1H), 6.84 – 6.76 (m, 2H), 2.44 (s, 3H), 1.05 (s, 9H). **^{13}C NMR (101 MHz, $CDCl_3$)** δ 165.46, 143.81, 135.30, 131.60, 129.28, 129.18, 127.82, 127.39,38.48, 27.96, 21.55. **HRMS (ESI):** [M+H] $^{+}$ calcd for $C_{18}H_{31}N_2O_{22}S$ 331.1475, found 331.1479.

(Z)-N'-(4-(4-methoxyphenyl)butan-2-ylidene)-4-methylbenzenesulfonohydrazide

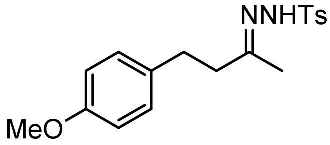

^{1}H NMR (400 MHz, Chloroform-*d*) δ 7.84 (d, *J* = 8.0 Hz, 2H), 7.43 (s, 1H), 7.30 (d, *J* = 8.0 Hz, 2H), 6.99 (d, *J* = 8.4 Hz, 2H), 6.75 (d, *J* = 8.8 Hz, 2H), 3.77 (s, 3H), 2.74 – 2.70 (m, 2H), 2.50 – 2.46 (m, 2H), 2.44 (s, 3H), 1.74 (s, 3H). **^{3}C NMR (101 MHz, Chloroform-*d*)** δ 157.84, 157.46, 157.44, 143.93, 135.41, 133.07, 129.44, 129.17, 128.07, 113.75, 55.19, 40.50, 31.11, 21.58, 16.05. **HRMS (ESI):** [M+H] $^{+}$ calcd for $C_{18}H_{23}N_2O_3S$ 347.1424, found 347.1418.

N'-(dicyclohexylmethylene)-4-methylbenzenesulfonohydrazide

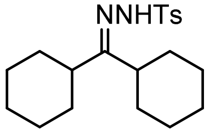

^{1}H NMR (400 MHz, Chloroform-*d*) δ 7.83 (d, *J* = 8.4 Hz, 2H), 7.48 (s, 1H), 7.29 (d, *J* = 8.0 Hz, 2H), 2.45-2.40 (m, 4H), 2.18-2.11 (m, 1H), 1.78 – 1.52 (m, 10H), 1.34 – 1.12 (m, 10H). **^{13}C NMR (101 MHz, Chloroform-*d*)** δ 143.81, 135.24, 129.28, 128.15, 41.40, 31.72, 28.41, 26.36, 25.83, 25.76, 25.71, 21.60. **HRMS (ESI):** [M+H] $^{+}$ calcd for $C_{20}H_{31}N_2O_2S$ 363.2101, found 363.2105.

(Z)-N'-(decan-2-ylidene)-4-methylbenzenesulfonohydrazide

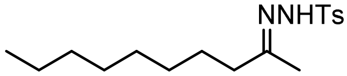

^{1}H NMR (400 MHz, Chloroform-*d*) δ 7.84 (d, *J* = 8.4 Hz, 2H), 7.30 (d, *J* = 8.0 Hz, 2H), 7.16 (s, 1H), 2.42 (s, 3H), 2.18 (t, *J* = 7.6 Hz, 2H), 1.74 (s, 3H), 1.42 (p, *J* = 7.6 Hz, 2H), 1.29 – 1.14 (m, 10H), 0.88 (t, *J* = 7.0 Hz, 3H). **^{13}C NMR (101 MHz, Chloroform-*d*)** δ 158.58, 143.85, 135.46, 129.41, 128.09, 38.68, 31.84, 29.33, 29.17, 28.95, 25.89, 22.65, 21.58, 15.44, 14.09. **HRMS (ESI):** [M+H] $^{+}$ calcd for $C_{17}H_{29}N_2O_2S$ 325.1944, found 325.1939.

(Z)-4-methyl-N'-(4-(2,6,6-trimethylcyclohex-1-en-1-yl)butan-2 ylidene)benzenesulfonohydrazide

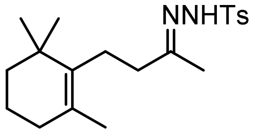

^{1}H NMR (400 MHz, Chloroform-*d*) δ 7.86 (d, *J* = 8.0 Hz, 2H), 7.29 (d, *J* = 8.4 Hz, 2H), 2.42 (s, 3H), 2.28 – 2.20 (m, 2H), 2.13-2.07 (m, 2H), 1.88 (t, *J* = 6.4 Hz, 2H), 1.78 (s, 3H), 1.57 – 1.52 (m, 5H), 1.43-1.34 (m, 2H), 0.93 (s, 6H). **^{13}C NMR (101 MHz, Chloroform-*d*)** δ 143.95, 136.05, 135.29, 129.98, 129.38, 128.23, 39.72, 39.29, 34.98, 32.72, 28.51, 28.48, 28.43, 24.98, 21.60, 19.76, 19.44, 15.84. **HRMS (ESI):** [M+H] $^+$ calcd for $C_{20}H_{31}N_2O_2S$ 363.2101, found 363.2009.

(Z)-4-methyl-N'-(2-methylpentylidene)benzenesulfonohydrazide

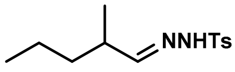

^{1}H NMR (400 MHz, Chloroform-*d*) δ 7.80 (d, *J* = 8.4 Hz, 2H), 7.55 (s, 1H), 7.30 (d, *J* = 8.0 Hz, 2H), 7.01 (d, *J* = 6.4 Hz, 1H), 2.42 (s, 3H), 2.37-2.30 (m, 1H), 1.34 – 1.20 (m, 2H), 1.16 – 1.07 (m, 2H), 0.97 (d, *J* = 7.2 Hz, 3H), 0.79 (t, *J* = 7.2 Hz, 3H). **^{13}C NMR (101 MHz, Chloroform-*d*)** δ 157.43, 144.04, 135.16, 129.50, 127.95, 36.37, 36.29, 21.56, 19.94, 17.53, 13.89. **HRMS (ESI):** [M+H] + calcd for $C_{13}H_{20}N_2O_2S$ 269.1318, found 269.1322.

(E)-N'-(3-(benzo[d][1,3]dioxol-5-yl)-2-methylpropylidene)-4-methylbenzenesulfonohydrazide

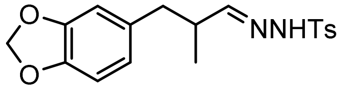

^{1}H NMR (400 MHz, Chloroform-*d*) δ 7.77 (d, *J* = 8.4 Hz, 2H), 7.69 (s, 1H), 7.29 (dd, *J* = 8.8, 0.8 Hz, 2H), 7.09 (d, *J* = 4.2 Hz, 1H), 6.63 (d, *J* = 8.0 Hz, 1H), 6.53 (d, *J* = 1.6 Hz, 1H), 6.47 (dd, *J* = 8.0, 1.6 Hz, 1H), 5.90 (dd, *J* = 2.4, 1.2 Hz, 2H), 2.70-2.65 (m, 1H), 2.63 – 2.55 (m, 1H), 2.49 – 2.45 (m, 1H), 2.43 (s, 3H), 0.98 (d, *J* = 6.8 Hz, 3H). **^{13}C NMR (101 MHz, Chloroform-*d*)** δ 155.94, 147.50, 145.86, 144.06, 135.19, 132.79, 129.52, 127.85, 121.89, 109.32, 108.05, 100.76, 40.05, 38.35, 21.57, 17.00. **HRMS (ESI):** [M+H] + calcd for $C_{18}H_{20}N_2O_4S$ 361.1217, found 361.1214.

(Z)-N'-(2,2-dimethylpropylidene)-4-methylbenzenesulfonohydrazide

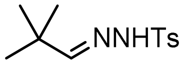

^{1}H NMR (400 MHz, Chloroform-*d*) δ 7.80 (d, *J* = 8.4 Hz, 2H), 7.31 (d, *J* = 8.0 Hz, 2H), 7.23 (s, 1H), 7.06 (s, 1H), 2.43 (s, 3H), 1.01 (s, 9H). **^{13}C NMR (101 MHz, Chloroform-*d*)** δ 160.23, 144.04, 129.41, 128.02, 35.13, 27.11, 21.59. **HRMS (ESI):** [M+H] + calcd for $C_{12}H_{18}N_2O_2S$ 255.1162, found 255.1164.

ethyl 4-(4-(3-methyl-4-oxobutyl)phenoxy)butanoate

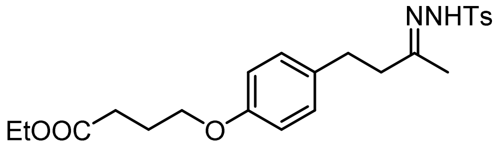

Following **General procedure B-SM** on 3.15 mmol scale, purification by flash column chromatography (30% to 50%, EtOAc in petroleum ether) on silica gel afforded 1.04g (78%) of the

title compound as a white solid, Rf = 0.3 (silica gel, petroleum ether/ethyl acetate = 3:1).

^{1}H NMR (400 MHz, CDCl$_3$) δ 7.84 (d, *J* = 8.0 Hz, 2H), 7.40 – 7.29 (m, 3H), 6.97 (d, *J* = 8.0 Hz, 2H), 6.73 (d, *J* = 8.0 Hz, 2H), 4.14 (dd, *J* = 14.0, 7.2 Hz, 2H), 4.00-3.94 (m, 2H), 2.70 (dd, *J* = 16.0, 8.0 Hz, 2H), 2.55 – 2.43 (m, 7H), 2.12 – 2.07 (m, 2H), 1.74 (s, 3H), 1.25 (t, *J* = 7.2 Hz, 3H). **^{13}C NMR (101 MHz, CDCl$_3$)** δ 173.21, 157.52, 157.09, 143.89, 135.42, 133.18, 129.43, 129.15, 128.06, 114.36, 66.69, 60.39, 40.48, 31.12, 30.79, 24.64, 21.56, 16.08, 14.18.

HRMS (ESI): [M+H]$^+$ calcd for $C_{23}H_{31}N_2O_5S$ 447.1948, found 447.1951.

(Z)-N'-(4-(4-(3-hydroxypropoxy)phenyl)butan-2-ylidene)-4-methylbenzenesulfonohydrazide

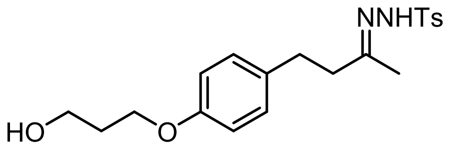

Following **General procedure B-SM** on 3.15 mmol scale, purification by flash column chromatography (30% to 50%, EtOAc in petroleum ether) on silica gel afforded 0.96g (82%) of the title compound as a white solid, Rf = 0.3 (silica gel, petroleum ether/ethyl acetate = 3:1).

^{1}H NMR (400 MHz, CDCl$_3$) δ 7.84 (d, *J* = 8.0 Hz, 2H), 7.31 (d, *J* = 8.2 Hz, 2H), 6.99 (d, *J* = 8.0 Hz, 2H), 6.76 (d, *J* = 8.0 Hz, 2H), 4.10 (t, *J* = 7.0 Hz, 2H), 3.87 (t, *J* = 7.0 Hz, 2H), 2.75-2.69 (m, 2H), 2.51-2.44 (m, 5H), 2.05 – 1.91 (m, 3H), 1.91 (s, 1H), 1.74 – 1.73 (m, 3H). **^{13}C NMR (101 MHz, CDCl$_3$)** δ 157.56 (d, *J* = 5.6 Hz), 156.99, 143.89, 135.40, 133.32, 129.42, 129.18, 128.04, 114.35, 65.78 (d, *J* = 3.1 Hz), 60.50 (d, *J* = 4.9 Hz), 40.43, 31.95, 31.10, 21.55, 16.11. **HRMS (ESI):** [M+H]$^+$ calcd for $C_{20}H_{27}N_2O_4S$ 391.1686, found 391.1690.

(Z)-N'-(4-(4-(but-2-yn-1-yloxy)phenyl)butan-2-ylidene)-4-methylbenzenesulfonohydrazide

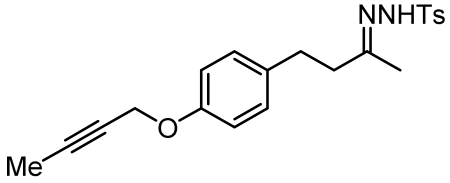

Following **General procedure B-SM** on 3.15 mmol scale, purification by flash column chromatography (15% to 20%, EtOAc in petroleum ether) on silica gel afforded 0.98g (85%) of the title compound as a white solid, Rf = 0.5 (silica gel, petroleum ether/ethyl acetate = 5:1).

^{1}H NMR (400 MHz, CDCl$_3$) δ 7.93 – 7.84 (m, 3H), 7.38 (d, *J* = 8.0 Hz, 2H), 7.07 (d, *J* = 8.0 Hz, 2H), 6.88 (d, *J* = 8.0 Hz, 2H), 4.67 (s, 2H), 2.79 (t, *J* = 8.0 Hz, 2H), 2.57-2.52 (m, 5H), 1.93 (s, 3H), 1.84 (s, 3H). **^{13}C NMR (101 MHz, CDCl$_3$)** δ 157.54, 156.01, 143.83, 135.36, 133.66, 129.37, 129.07, 127.95, 114.59, 83.50, 74.05, 56.30, 40.36, 31.03, 21.49, 16.11, 3.58.**HRMS (ESI):** [M+H]$^+$ calcd for $C_{21}H_{25}N_2O_3S$ 385.1580, found 385.1578.

(Z)-N'-(4-(4-(allyloxy)phenyl)butan-2-ylidene)-4-methylbenzenesulfonohydrazide

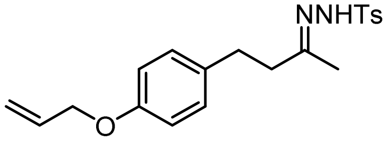

Following **General procedure B-SM** on 3.15 mmol scale, purification by flash column

chromatography (10% to 15%, EtOAc in petroleum ether) on silica gel afforded 0.93 g (83% yield) of the title compound as a white solid, Rf = 0.5 (silica gel, petroleum ether/ethyl acetate = 5:1).

^{1}H NMR (400 MHz, CDCl$_3$) δ 7.84 (d, *J* = 8.0 Hz, 2H), 7.30 (d, *J* = 8.0 Hz, 2H), 6.98 (d, *J* = 8.8 Hz, 2H), 6.77 (d, *J* = 8.4 Hz, 2H), 6.10 – 6.00 (m, 1H), 5.43-5.37 (m, 1H), 5.29 – 5.26 (m, 1H), 4.51-4.48 (m, 2H), 2.74 – 2.70 (m, 2H), 2.50-2.43 (m, 6H), 1.75 (d, *J* = 2.0 Hz, 3H). **^{13}C NMR (101 MHz, CDCl$_3$)** δ 157.54, 156.88, 143.92, 135.43, 133.39, 133.28, 129.44, 129.15, 128.07, 117.51, 114.63, 68.81, 40.45, 31.13, 21.58, 16.07.

HRMS (ESI): [M+H]$^+$ calcd for $C_{20}H_{25}N_2O_3S$ 373.1580, found 373.1577.

(E)-4-methyl-N'-(4-(4-(pyridin-2-ylmethoxy)phenyl)butan-2-ylidene)benzenesulfonohydrazide

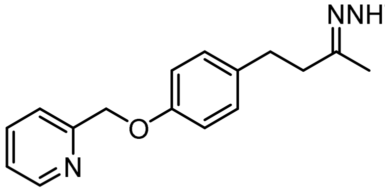

Following **General procedure B-SM** on 3.15 mmol scale, purification by flash column chromatography (25% to 50%, EtOAc in petroleum ether) on silica gel afforded 1.03 g (81% yield) of the title compound as a white solid, Rf = 0.5 (silica gel, petroleum ether/ethyl acetate = 5:1).

^{1}H NMR (400 MHz, CDCl$_3$) δ 8.61 – 8.49 (m, 2H), 7.78 – 7.65 (m, 4H), 7.26 – 7.20 (m, 3H), 6.94 (d, *J* = 8.8 Hz, 2H), 6.76-6.74 (d, *J* = 8.8 Hz, 2H), 4.96 (s, 2H), 2.68-2.62 (m, 2H), 2.42 – 2.35 (m, 5H), 1.68 (s, 3H). **^{13}C NMR (101 MHz, CDCl$_3$)** δ 157.42, 156.63, 149.29, 148.86, 143.85, 135.49, 135.26, 133.95, 132.65, 129.40, 129.33, 128.06, 123.49, 114.75, 67.57, 40.40, 31.11, 21.56, 16.16.

HRMS (ESI): [M+H]$^+$ calcd for $C_{23}H_{26}N_3O_3S$ 424.1689, found 424.1683.

(Z)-N'-(4-(4-(3-chloropropoxy)phenyl)butan-2-ylidene)-4-methylbenzenesulfonohydrazide

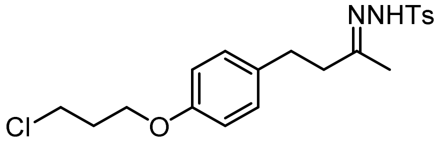

Following **General procedure B-SM** on 3.15 mmol scale, purification by flash column chromatography (25% to 50%, EtOAc in petroleum ether) on silica gel afforded 0.83 g (68% yield) of the title compound as a white solid, Rf = 0.3 (silica gel, petroleum ether/ethyl acetate = 4:1).

^{1}H NMR (400 MHz, CDCl$_3$) δ 7.85 (d, *J* =8.0 Hz, 2H), 7.71 (s, 1H), 7.31 (d, *J* = 8.0 Hz, 2H), 6.99 (d, *J* = 8.0 Hz, 2H), 6.76 (d, *J* = 8.4 Hz, 2H), 4.07 (t, *J* = 6.0 Hz, 2H), 3.74 (t, *J* = 6.4 Hz, 2H), 2.73 – 2.68 (m, 2H), 2.49-2.44 (m, 5H), 2.27 – 2.19 (m, 2H), 1.75 (s, 3H). **^{13}C NMR (101 MHz, CDCl$_3$)** δ 157.40, 157.01, 143.94, 135.46, 133.41, 129.46, 129.24, 128.11, 114.45, 64.30, 41.53, 40.47, 32.30, 31.13, 21.59, 16.01. **HRMS (ESI):** [M+H]$^+$ calcd for $C_{20}H_{26}ClN_2O_3S$ 409.1347, found 409.1356.

(Z)-N'-(4-(4-(4-cyanobutoxy)phenyl)butan-2-ylidene)-4-methylbenzenesulfonohydrazide

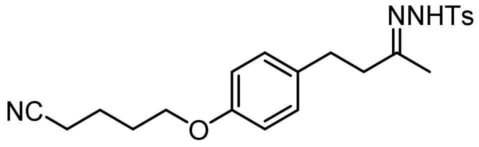

Following **General procedure B-SM** on 3.15 mmol scale, purification by flash column

chromatography (15% to 25%, EtOAc in petroleum ether) on silica gel to afford 0.95 g (77% yield) of the title compound as a white solid, Rf = 0.3 (silica gel, petroleum ether/ethyl acetate = 3:1). **^{1}H NMR (400 MHz, CDCl$_3$)** δ 7.84 (d, *J* = 8.0 Hz, 2H), 7.47 (s, 1H), 7.31 (d, *J* = 8.0 Hz, 2H), 6.99 (d, *J* = 8.0 Hz, 2H), 6.74 (d, *J* = 8.0 Hz, 2H), 4.01-3.95 (m, 2H), 2.74 – 2.70 (m, 2H), 2.49-2.44 (m, 7H), 1.96 – 1.89 (m, 4H), 1.74 (s, 3H). **^{13}C NMR (101 MHz, CDCl$_3$)** δ 157.53, 156.96, 143.91, 135.44, 133.39, 129.44, 129.25, 128.09, 119.49, 114.34, 66.63, 40.46, 31.13, 28.17, 22.44, 21.58, 16.97, 16.08.
HRMS (ESI): [M+H]$^+$ calcd for $C_{22}H_{28}N_3O_3S$ 414.1846, found 414.1854.

4-(pyridin-2-ylmethoxy)benzaldehyde

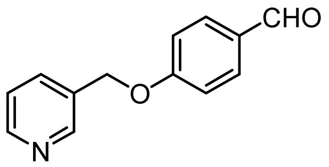

Following **General procedure D-SM** on 3 mmol scale starting from 3-Picolyl chloride hydrochlorid, purification by flash column chromatography (15% to 25%, EtOAc in petroleum ether) on silica gel afforded the title compound as a white solid 0.47 g (73% yield), Rf = 0.3 (silica gel, petroleum ether/ethyl acetate = 2:1). **^{1}H NMR (400 MHz, Chloroform-*d*)** δ 9.89 (s, 1H), 8.69-8.61 (m 2H), 7.85 (d, *J* = 8.8 Hz, 2H), 7.78 (d, *J* = 7.6 Hz, 1H), 7.36-7.33 (m, 1H), 7.08 (d, *J* = 8.8 Hz, 2H), 5.16 (s, 2H). **^{13}C NMR (101 MHz, Chloroform-*d*)** δ 190.65, 163.19, 149.76, 148.95, 135.26, 132.00, 131.53, 130.45, 123.57, 115.03, 67.75. **HRMS (ESI):** [M+H]$^+$ calcd for $C_{13}H_{12}NO_2$ 214.0863, found 214.0860.

4-(allyloxy)benzaldehyde

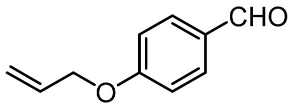

Following **General procedure D-SM** on 3 mmol scale starting from 3-bromoprop-1-ene (0.38 g, 3.15 mmol), purification by flash column chromatography (1% to 3%, EtOAc in petroleum ether) on silica gel afforded the title compound as a white solid 0.40 g (83% yield), Rf = 0.3 (silica gel, petroleum ether/ethyl acetate = 30:1).
^{1}H NMR (400 MHz, Chloroform-*d*) δ 9.88 (s, 1H), 7.83 (d, *J* = 8.8 Hz, 2H), 7.10 (d, *J* = 8.4 Hz, 2H), 6.09-6.0 (m, 1H), 5.43 (d, *J* = 17.2 Hz, 1H), 5.33 (d, *J* = 10.4 Hz, 1H), 4.62 (d, *J* = 4.8 Hz, 2H). **^{13}C NMR (101 MHz, Chloroform-*d*)** δ 190.75, 163.57, 132.25, 131.92, 130.01, 118.31, 114.97, 68.97. **HRMS (ESI):** [M+H]$^+$ calcd for $C_{10}H_{11}O_2$ 163.0754, found 163.0753.

4-(but-2-yn-1-yloxy)benzaldehyde

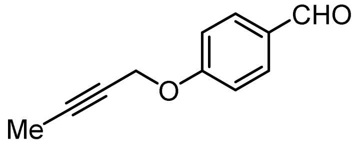

Following **General procedure D-SM** on 3 mmol scale starting from 1-Bromo-2-butyne (0.42 g, 3.15 mmol, purification by flash column chromatography (1% to 3%, EtOAc in petroleum ether) on silica gel afforded the title compound as a white solid 0.45 g (86% yield), Rf = 0.3 (silica gel, petroleum ether/ethyl acetate = 30:1). **^{1}H NMR (400 MHz, Chloroform-*d*)** δ 9.89 (s, 1H), 7.84 (d, *J*

= 8.4 Hz, 2H), 7.07 (d, *J* = 8.4 Hz, 2H), 4.73 (d, *J* = 8.4 Hz, 2H), 1.86 (t, *J* = 2.4 Hz, 3H).**^{13}C NMR (101 MHz, Chloroform-*d*)** δ 190.79, 162.74, 131.85, 130.29, 115.14, 84.67, 73.07, 56.63, 3.64. **HRMS (ESI):** [M+H] $^{+}$ calcd for $C_{11}H_{11}O_2$ 175.0754, found 175.0751.

4-(tert-Butyl)cyclohexane-1-carbaldehyde (3b)

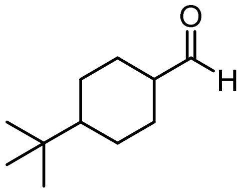

Following the general procedure A, the product was obtained as a colorless oil, 17.1 mg, 51% yield, (trans : cis = 3.8 : 1). **^{1}H NMR** (300 MHz, $CDCl_3$) δ 9.71 (s, 0.26 H), 9.60 (d, *J* = 1.8 Hz, 1H), 2.42-2.37 (m, 0.29 H), 2.31-2.24 (m, 0.59 H), 2.18-2.07 (m, 1H), 2.05-1.97 (m, 2H), 1.92-1.86 (m, 2H), 1.73-1.63 (m, 0.77H), 1.57-1.45(m, 1.6H), 1.29-1.15 (m, 2.4H), 1.09-0.90 (m, 6H), 0.85 (s, 9H), 0.79 (s, 2.5H). **^{13}C NMR** (75 MHz, $CDCl_3$) δ 205.97, 204.97, 50.51, 47.76, 47.55, 46.47, 32.46, 32.43, 31.75, 29.67, 27.45, 27.35, 26.46, 26.17, 25.44, 24.06. **HRMS** (EI), Calcd. for $C_{11}H_{20}O$ $[M]^+$ 168.1509, found, 168.1513.

4-Phenylcyclohexane-1-carbaldehyde (3c)

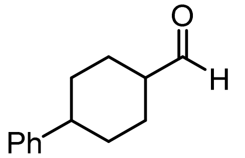

Following the general procedure A, the product was obtained as a white solid, 32.3 mg, 86% yield (trans : cis = 1:11.2). **^{1}H NMR** (300 MHz, $CDCl_3$) δ 9.79 (s, 0.7H), 9.68 (d, J = 1.8, 0.1H), 7.32-7.25 (m, 2H), 7.24-7.14 (m, 3H), 2.91-2.70 (m, 0.2H), 2.58-2.48 (m, 1.8H), 2.38-2.31 (m, 1.73 H), 2.20-1.93 (m, 0.6 H), 1.85 – 1.67 (m, 4H), 1.57-1.41 (m, 2H). **^{13}C NMR** (75 MHz, $CDCl_3$) δ 205.50, 146.80, 134.96, 128.35, 126.73, 126.04, 46.24, 43.59, 32.91, 30.61, 26.3, 24.98. **HRMS** (EI), Calcd. for $C_{13}H_{16}O$ $[M]^+$ 188.1210, found, 188.1209.

Adamantane-2-carbaldehyde (3d)

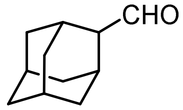

Following the general procedure A, the product was obtained as a colorless oil, 24.0 mg, 73% yield. **^{1}H NMR** (300 MHz, $CDCl_3$) δ 9.73 (s, 1H), 2.42-2.38 (m, 3H), 1.98-1.91 (m, 2H), 1.89-1.83 (m, 2H), 1.80-1.61 (m, 9H). **^{13}C NMR** (75 MHz, $CDCl_3$) δ 206.01, 56.55, 37.79, 36.99, 33.48, 28.11, 27.87, 27.44. **HRMS** (EI) Calcd. for $C_{11}H_{16}O$ $[M]^+$: 164.1201. Found: 164.1209. **HRMS** (EI) Calcd. for $C_{11}H_{16}O$ $[M]^+$: 164.1201. Found: 164.1209.

Cyclododecanecarbaldehyde (3e)

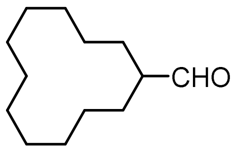

Following the general procedure A, the product was obtained as a colorless oil, 28.2 mg, 72% yield.

^{1}H NMR (300 MHz, $CDCl_3$) δ 9.63 (d, *J* = 1.5 Hz, 1H), 2.44-2.35 (m, 1H), 1.70-1.59 (m, 2H), 1.57-1.44 (m, 2H), 1.42-1.29 (m, 18H). **^{13}C NMR** (75 MHz, $CDCl_3$) δ 205.41, 47.71, 23.72, 23.58, 23.52, 23.45, 23.33, 22.30. **HRMS** (EI), Calcd. for $C_{13}H_{24}O$ $[M]^+$ 196.1827, found, 196.1819.

Cyclopentadecanecarbaldehyde (3f)

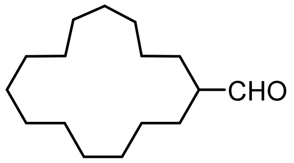

Following the general procedure A, the product was obtained as a colorless oil, 20.0mg, 42% yield. **^{1}H NMR** (300 MHz, $CDCl_3$) δ 9.61 (d, *J* = 1.8 Hz, 1H), 2.33-2.24 (m, 1H), 1.74-1.61 (m, 2H), 1.57-1.44 (m, 2H), 1.43-1.28 (m, 24H). **^{13}C NMR** (75 MHz, $CDCl_3$) δ 205.32, 50.20, 27.07, 26.70, 26.67, 26.60, 26.49, 26.38, 24.82. **HRMS** (EI) Calcd. for $C_{16}H_{30}O[M]^+$: 238.2291. Found: 238.2293.

tert-Butyl 4-formylpiperidine-1-carboxylate (3g)

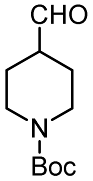

Following the general procedure A, the product was obtained as a yellowish oil, 14.5 mg, 34% yield. **^{1}H NMR** (300 MHz, $CDCl_3$) δ 9.65 (d, *J* = 1.2 Hz, 1H), 4.00-3.95 (m, 2H), 2.96-2.87 (m, 2H), 2.46 - 2.35 (m, 1H), 1.93-1.85 (m, 2H), 1.61-1.48 (m, 2H), 1.45 (s, 9H). **^{13}C NMR** (75 MHz, $CDCl_3$) δ 202.99, 154.65, 79.72, 47.99, 28.39, 25.13. **HRMS** (ESI) Calcd. for $C_{11}H_{20}NO_3^+$ $[M+H]^+$: 214.1438. Found: 214.1434.

Methyl 2-(4-((2-formylcyclopentyl)methyl)phenyl)propanoate (3i)

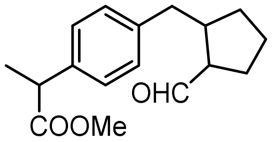

Following the general procedure A, the product was obtained as a yellowish oil, 27.9 mg, 51% yield. **^{1}H NMR** (300 MHz, $CDCl_3$) δ 9.44 (d, *J* = 2.4 Hz, 1H), 7.22-7.18 (m, 2H), 7.14-7.10 (m, 2H), 3.73-3.63 (m, 4H), 2.76-2.58 (m, 2H), 2.49-2.34 (m, 2H), 1.90-1.54 (m, 5H), 1.47 (d, *J* = 7.2 Hz, 3H), 1.39-1.32 (m, 1H). **^{13}C NMR** (75 MHz, $CDCl_3$) δ 203.59, 175.05, 139.35, 138.28, 129.14, 127.41, 57.20, 51.96, 44.93, 42.87, 40.41, 32.40, 26.19, 24.54, 18.52. **HRMS** (EI): Calcd. For $C_{17}H_{22}O_3$ $[M]^+$: 274.1563, Found: 274.1558. *Note: the stereochemistry of this product was not assigned due to NMR signal overlap.*

2-(4-Isobutylphenyl)propanal (3k)

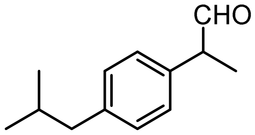

Following the general procedure B, the product was obtained as a colorless oil, 23.9 mg, 63% yield. **^{1}H NMR** (400 MHz, $CDCl_3$) δ 9.68 (d, *J* = 1.5 Hz, 1H), 7.18-7.11 (m, 4H), 3.64-3.58 (m, 1H), 2.48 (d, *J* = 7.2 Hz, 2H), 1.92-1.82 (m, 1H), 1.44 (d, *J* = 7.2 Hz, 3H), 0.93 (s, 3H), 0.91 (s, 3H). **^{13}C NMR** (101

MHz, $CDCl_3$) δ 201.16, 140.97, 134.80, 129.75, 127.97, 52.58, 44.96, 30.14, 22.31, 14.51. **HRMS** (EI): Calcd. For $C_{13}H_{18}O$ $[M]^+$: 190.1358, Found: 190.1349.

3-Methyl-2-phenylbutanal (3l)

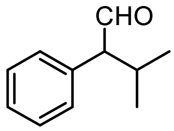

Following the general procedure A, the product was obtained as a colorless oil, 14.3 mg, 44% yield. **^{1}H NMR** (300 MHz, $CDCl_3$) δ 9.71 (d, J = 3.3 Hz, 1H), 7.40-7.27 (m, 3H), 7.21-7.17 (m, 2H), 3.18 (dd, J = 9.3, 3.3 Hz, 1H), 2.50-2.34 (m, 1H), 1.05 (d, J = 6.6 Hz, 3H), 0.77 (d, J = 6.6 Hz, 3H). **^{13}C NMR** (75 MHz, $CDCl_3$) δ 201.13, 135.45, 129.29, 128.87, 127.44, 66.82, 28.75, 21.16, 20.01. **HRMS** (EI) Calcd. for $C_{11}H_{14}O$ $[M]^+$: 162.1045. Found: 162.1046. Spectroscopic results agree with preciously reported data.[5]

3,3-Dimethyl-2-phenylbutanal (3m)

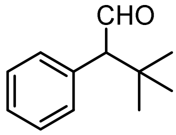

Following the general procedure A, the product was obtained as a colorless oil, 14.8 mg, 42% yield. **^{1}H NMR** (300 MHz, $CDCl_3$) δ 10.01 (d, J = 3.3 Hz, 1H), 7.39-7.27 (m, 3H), 7.24-7.20 (m, 2H), 3.29 (d, J = 3.3 Hz, 1H), 1.03 (s, 9H). **^{13}C NMR** (75 MHz, $CDCl_3$) δ 202.27, 135.20, 130.35, 128.29, 127.26, 68.34, 34.55, 28.18. **HRMS** (ESI) Calcd. for $[M+NH_4]^+$: 194.1539. Found: 194.1538. Spectroscopic results agree with previously reported data.[6]

2-Cyclohexyl-2-phenylacetaldehyde (3n)

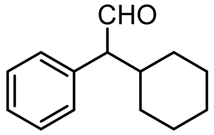

Following the general procedure A, the product was obtained as a colorless oil, 22.6 mg, 56% yield. **^{1}H NMR** (300 MHz, $CDCl_3$) δ 9.70 (d, J = 3.6 Hz, 1H), 7.39-7.26 (m, 3H), 7.20-7.16 (m, 2H), 3.25 (dd, J = 9.6, 3.6 Hz, 1H), 2.18-2.05 (m, 1H), 1.89-1.71 (m, 2H), 1.69-1.61 (m, 2H), 1.46-1.25 (m, 2H), 1.24-1.00 (m, 3H), 0.87-0.74 (m, 1H). **^{13}C NMR** (75 MHz, $CDCl_3$) δ 201.21, 135.19, 129.29, 128.85, 127.37, 65.82, 38.12, 31.77, 30.15, 26.20, 26.04, 26.00. **HRMS** (EI) Calcd. for $C_{14}H_{18}O$ $[M]^+$: 202.1352. Found: 202.1352. Spectroscopic results agree with preciously reported data.[6]

2-Methyldecanal (3s)

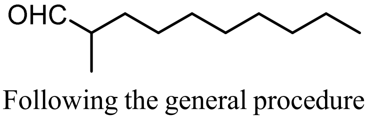

Following the general procedure D, the product was obtained as a colorless oil, 25.4 mg, 75% yield. **^{1}H NMR** (400 MHz, $CDCl_3$) δ 9.60 (d, J = 2.0 Hz, 1H), 2.36-2.27 (m, 1H), 1.74-1.63 (m, 1H), 1.37 – 1.26 (m, 13H), 1.07 (d, J = 7.2 Hz, 3H), 0.88 – 0.85 (m, 3H). **^{13}C NMR** (101 MHz, $CDCl_3$) δ 205.32, 46.29, 31.82, 30.50, 29.60, 29.38, 29.20, 26.91, 22.62, 14.05, 13.28. **HRMS** (ESI): Calcd. For $[M+NH_4]^+$: 188.2009, Found: 188.2007.

4-(4-Methoxyphenyl)-2-methylbutanal (3t)

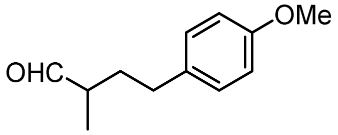

Following the general procedure D, the product was obtained as a colorless oil, 30.0 mg, 78% yield. Following the general procedure A with **1j** (8 mmol), the product was obtained as a colorless oil, 675.8 mg, 44% yield. **^{1}H NMR** (400 MHz, $CDCl_3$) δ 9.62 (d, *J* = 1.6 Hz, 1H), 7.12-7.08 (m, 2H), 6.85-6.82 (m, 2H), 3.79 (s, 3H), 2.68-2.55 (m, 2H), 2.42-2.32 (m, 1H), 1.68-1.59 (m, 1H), 1.14 (d, *J* = 6.8 Hz, 3H). **^{13}C NMR** (101 MHz, $CDCl_3$) δ 204.89, 157.95, 133.37, 129.28, 113.88, 55.24, 45.57, 32.38, 32.12, 13.32. **HRMS** (EI): Calcd. For $C_{12}H_{16}O_2$ $[M]^+$: 192.1145, Found: 192.1150.

2-(4-Methoxyphenethyl)-2-methyloxirane (3t-1)

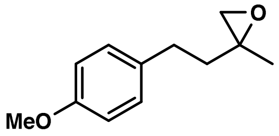

Following the general procedure E, the byproduct was obtained as a colorless oil, 6.2 mg, 16% yield. **^{1}H NMR** (400 MHz, $CDCl_3$) δ 7.10 (d, J = 8.6 Hz, 2H), 6.83 (d, J = 8.6 Hz, 2H), 3.79 (s, 3H), 2.68 – 2.62 (m, 2H), 2.62 – 2.56 (m. 2H), 1.90 (ddd, J = 13.8, 9.1, 6.9 Hz, 1H), 1.79 (ddd, J = 13.8, 9.1, 6.7 Hz, 1H), 1.37 (s, 3H). **^{13}C NMR** (101 MHz, $CDCl_3$) δ 157.9, 133.7, 129.3, 129.2, 113.9, 113.9, 56.7, 55.3, 54.0, 38.8, 30.5, 21.0.

2-methyl-4-(4-(pyridin-2-ylmethoxy)phenyl)butanal (3u)

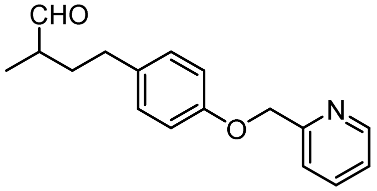

Following the general procedure D, the product was obtained as a colorless oil, 35 mg, 65% yield. **^{1}H NMR** (400 MHz, $CDCl_3$) δ 9.60 (d, *J* = 2.0 Hz, 1H), 8.67 – 8.56 (m, 2H), 7.77 (d, *J* = 8.0 Hz, 1H), 7.32 (dd, *J* = 8.0, 4.8 Hz, 1H), 7.10 (d, *J* = 8.4, 2H), 6.89 (d, *J* = 8.8 Hz, 2H), 5.05 (s, 2H), 2.65 – 2.57 (m, 2H), 2.38 – 2.32 (m, 1H), 2.07-1.97 (m, 1H), 1.67-1.58 (m, 1H), 1.13 (dd, *J* = 7.2, 2.1 Hz, 3H). **^{13}C NMR** (101 MHz, $CDCl_3$) δ 204.78, 156.71, 149.21, 148.80, 135.30, 134.16, 132.68, 129.40, 123.49, 114.80, 67.53, 45.52, 32.27, 32.09, 13.30. **HRMS (ESI):** $[M+H]^+$ calcd for $C_{17}H_{20}NO_2$ 270.1489, found 270.1489.

4-(4-(allyloxy)phenyl)-2-methylbutanal (3v)

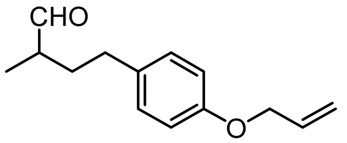

Following the general procedure D, the product was obtained as a colorless oil, 24 mg, 55% yield. **^{1}H NMR (400 MHz, Chloroform-*d*)** δ 9.61 (d, *J* = 1.6 Hz, 1H), 7.09 (d, *J* = 8.4 Hz, 2H), 6.85 (d, *J* = 8.4 Hz, 2H), 6.10 (m, 1H), 5.44 – 5.26 (m, 2H), 4.53-4.50 (m, 2H), 2.68-2.54 (m, 2H), 2.41-2.32 (m, 1H),

2.04-1.98 (m, 1H), 1.68 – 1.59 (m, 1H), 1.14 (d, *J* = 6.8 Hz, 3H). **^{13}C NMR (101 MHz, Chloroform-*d*)** δ 204.84, 156.97, 133.56, 133.41, 129.25, 117.52, 114.74, 68.84, 45.56, 32.33, 32.12, 13.31. **HRMS (ESI):** [M+H]$^{+}$ calcd for $C_{14}H_{19}O_2$ 219.1380, found 219.1383.

4-(4-(but-2-yn-1-yloxy)phenyl)-2-methylbutanal (3w)

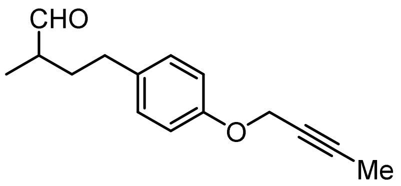

Following the general procedure D, the product was obtained as a colorless oil, 33 mg, 72% yield. **^{1}H NMR (400 MHz, Chloroform-*d*)** δ 9.61 (d, *J* = 2.0 Hz, 1H), 7.10 (d, *J* = 8.4 Hz, 2H), 6.89 (d, *J* = 8.4 Hz, 2H), 4.62 (d, *J* = 2.4 Hz, 2H), 2.68 – 2.54 (m, 2H), 2.41 – 2.32 (m, 1H), 2.07-1.98 (m, 1H), 1.86 (s, 3H), 1.68-1.58 (m, 1H), 1.14 (d, *J* = 7.2 Hz, 3H). **^{13}C NMR (101 MHz, Chloroform-*d*)** δ 204.81, 156.20, 133.96, 129.22, 114.80, 83.55, 74.11, 56.42, 45.53, 32.29, 32.11, 13.29, 3.65. **HRMS (ESI):** [M+H]$^{+}$ calcd for $C_{15}H_{19}O_2$ 231.1380, found 231.1378.

4-(4-(3-chloropropoxy)phenyl)-2-methylbutanal (3x)

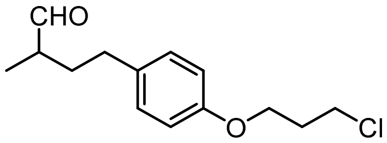

Following the general procedure D, the product was obtained as a colorless oil, 32 mg, 63% yield. **^{1}H NMR (400 MHz, Chloroform-*d*)** δ 9.61 (d, *J* = 2.0 Hz, 1H), 7.10 (d, *J* = 8.0 Hz, 2H), 6.84 (d, *J* = 8.4 Hz, 2H), 4.09 (t, *J* = 6.2 Hz, 2H), 3.74 (t, *J* = 6.4 Hz, 2H), 2.68-2.55 (m, 2H), 2.39 – 2.32 (m, 1H), 2.25-2.19 (m, 2H), 2.07-1.98 (m, 1H), 1.68-1.59 (m, 1H), 1.14 (d, *J* = 7.2 Hz, 3H). **^{13}C NMR (101 MHz, Chloroform-*d*)** δ 204.84, 157.06, 133.64, 129.31, 114.50, 64.26, 45.53, 41.53, 32.34, 32.28, 32.10, 13.30. **HRMS (ESI):** [M+H] $^{+}$ calcd for $C_{14}H_{20}ClO_2$ 255.1146, found 255.1137.

4-(4-(3-hydroxypropoxy)phenyl)-2-methylbutanal (3y)

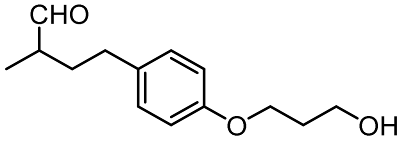

Following the general procedure D, the product was obtained as a colorless oil, 20 mg, 42% yield. **^{1}H NMR (400 MHz, Chloroform-*d*)** δ 9.60 (d, *J* = 2.0 Hz, 1H), 7.09 (d, *J* = 8.4 Hz, 2H), 6.83 (d, *J* = 8.4 Hz, 2H), 4.10 (t, *J* = 6.0 Hz, 2H), 3.85 (t, *J* = 6.0 Hz, 2H), 2.67 – 2.54 (m, 2H), 2.39-2.31 (m, 1H), 2.06 – 1.96 (m, 3H), 1.79 – 1.58 (m, 2H), 1.13 (d, *J* = 6.8 Hz, 3H). **^{13}C NMR (101 MHz, Chloroform-*d*)** δ 204.89, 157.11, 133.61, 129.30, 114.49, 65.82, 60.53, 45.54, 32.34, 32.10, 31.97, 13.30. **HRMS (ESI):** [M+H] $^{+}$ calcd for $C_{14}H_{21}O_3$ 237.1485, found 237.1493.

Ethyl 4-(4-(3-methyl-4-oxobutyl)phenoxy)butanoate (3z)

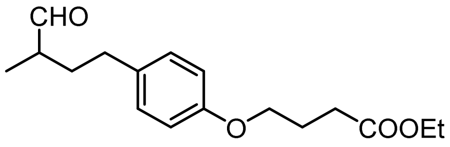

Following the general procedure D, the product was obtained as a colorless oil, 31 mg, 53% yield.

^{1}H NMR (400 MHz, Chloroform-*d*) δ 9.61 (d, *J* = 2.0 Hz, 1H), 7.08 (d, *J* = 8.4 Hz, 2H), 6.81 (d, *J* = 2.0 Hz, 2H), 4.17-4.11 (m, 2H), 3.98 (t, *J* = 6.2 Hz, 2H), 2.66 – 2.49 (m, 4H), 2.40-2.31 (m, 1H), 2.13 – 1.98 (m, 3H), 1.67 – 1.61 (m, 1H), 1.25 (t, *J* = 7.2 Hz, 3H), 1.14 (d, *J* = 7.2 Hz, 2H). **^{13}C NMR (101 MHz, Chloroform-*d*)** δ 204.86, 173.25, 157.22, 133.47, 129.28, 114.50, 66.75, 60.40, 45.57, 32.38, 32.13, 30.83, 24.68, 14.21, 13.32. **HRMS (ESI):** [M+H] $^+$ calcd for $C_{17}H_{25}O_4$ 293.1747, found 293.1752.

5-(4-(3-methyl-4-oxobutyl)phenoxy)pentanenitrile (3aa)

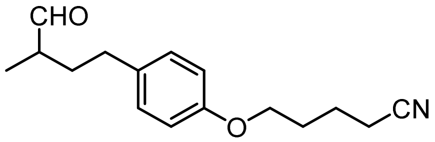

Following the general procedure D, the product was obtained as a colorless oil, 30 mg, 58% yield. **^{1}H NMR (400 MHz, Chloroform-*d*)** δ 9.61 (d, *J* = 2.0 Hz, 1H), 7.09 (d, *J* = 8.0 Hz, 2H), 6.81 (d, *J* = 8.8 Hz, 2H), 3.99-3.96 (m, 2H), 2.67 – 2.54 (m, 2H), 2.46 – 2.33 (m, 3H), 2.07 – 1.84 (m, 5H), 1.67-1.58 (m, 1H), 1.13 (d, *J* = 7.6 Hz, 3H). **^{13}C NMR (101 MHz, Chloroform-*d*)** δ 204.79, 157.03, 133.64, 129.32, 119.47, 114.40, 66.61, 45.52, 32.32, 32.08, 28.16, 22.43, 16.94, 13.30. **HRMS (ESI):** [M+H] $^+$ calcd for $C_{16}H_{22}NO_2$ 260.1465, found 260.1456.

2-Benzylbutanal (3ab)

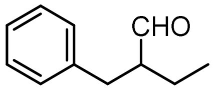

Following the general procedure D, the product was obtained as a colorless oil, 25.6 mg, 79% yield. **^{1}H NMR** (300 MHz, $CDCl_3$) δ 9.68 (d, *J* = 2.7 Hz, 1H), 7.32-7.15 (m, 5H), 3.04-2.97 (m, 1H), 2.76-2069 (m, 1H), 2.65-2.52 (m, 1H), 1.76-1.50 (m, 2H), 0.95 (t, *J* = 7.5 Hz, 3H). **^{13}C NMR** (75 MHz, $CDCl_3$) δ 204.71, 138.91, 128.92, 128.48, 126.32, 54.79, 34.53, 21.52, 11.30. **HRMS** (EI) Calcd. for $C_{11}H_{14}O$ $[M]^+$: 162.1045. Found: 162.1047. Spectroscopic results agree with previously reported data.[3]

2-Benzyl-3-phenylpropanal (3ac)

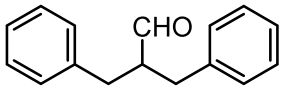

Following the general procedure D, the product was obtained as a colorless oil, 23.4 mg, 53% yield. **^{1}H NMR** (400 MHz, $CDCl_3$) δ 9.73 (d, *J* = 1.2 Hz, 1H), 7.32-7.27 (m, 4H), 7.24-7.14 (m, 6H), 3.03-2.85 (m, 3H), 2.81-2.73 (m, 2H). **^{13}C NMR** (101 MHz, $CDCl_3$) δ 204.03, 138.62, 129.83, 129.05, 128.63, 128.37, 126.55, 54.88, 34.94. **HRMS** (EI) Calcd. for $C_{16}H_{16}O$ $[M]^+$: 224.1196. Found: 224.1195. Spectroscopic results agree with previously reported data.[3]

2-Methyl-3,3-diphenylpropanal (3ad)

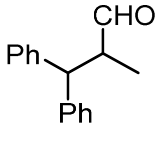

Following the general procedure D, the product was obtained as a colorless oil, 39.0 mg, 87% yield. **^{1}H NMR** (300 MHz, $CDCl_3$) δ 9.59 (d, *J* = 3.0 Hz, 1H), 7.34-7.25 (m, 8H), 7.23-7.16 (m, 2H), 4.09 (d, *J* = 11.1 Hz, 1H), 3.38-3.26 (m, 1H), 1.05 (d, *J* = 6.9 Hz, 3H). **^{13}C NMR** (75 MHz, $CDCl_3$) δ

204.20, 142.17, 142.04, 128.80, 128.73, 128.11, 127.97, 126.79, 126.71, 53.35, 50.11, 13.61. **HRMS** (EI): Calcd. For $C_{16}H_{16}O$ $[M]^+$: 224.1196, Found: 224.1199. Spectroscopic results agree with previously reported data.[4]

2,2-Dicyclohexylacetaldehyde (3ae)

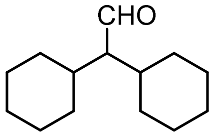

Following the general procedure D, the product was obtained as a colorless oil, 31.6 mg, 76% yield. Following the general procedure E starting from cyclohexananone (0.8 mmol), the product was obtained as a colorless oil, 36.8 mg, 41% yield over 2 steps. **^{1}H NMR** (300 MHz, $CDCl_3$) δ 9.65 (d, *J* = 4.8 Hz, 1H), 1.87-1.63 (m, 13H), 1.32-1.12 (m, 6H), 1.06-0.91 (m, 4H). **^{13}C NMR** (75 MHz, $CDCl_3$) δ 207.77, 63.06, 35.44, 31.21, 30.00, 26.54, 26.45, 26.39. **HRMS** (ESI) Calcd. for $[M+NH_4]^+$: 226.2165. Found: 226.2164.

2-Methyl-4-(2,6,6-trimethylcyclohex-1-enyl)butanal (3ag)

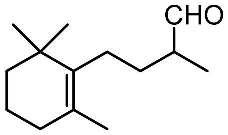

Following the general procedure D, the product was obtained as a colorless oil, 34.7 mg, 84% yield. **^{1}H NMR** (300 MHz, $CDCl_3$) δ 9.64 (d, *J* = 1.2 Hz, 1H), 2.41-2.29 (m, 0.8H), 2.07-1.95 (m, 2H), 1.92 – 1.87 (m, 2H), 1.82-1.69 (m, 1H), 1.60 - 1.52 (m, 5H), 1.49-1.37 (m, 3H), 1.13 (d, *J* = 6.9 Hz, 3H), 0.98 (s, 3H), 0.97 (s, 3H). **^{13}C NMR** (75 MHz, $CDCl_3$) δ 205.07, 136.60, 127.53, 47.16, 39.72, 34.87, 32.70, 30.99, 28.57, 26.05, 19.84, 19.44, 13.17. **HRMS** (EI): Calcd. For $C_{14}H_{24}O$ $[M]^+$: 208.1822, Found: 208.1827. Spectroscopic results agree with preciously reported data.[7]

6-(3,7-Dimethyl-2,6-dioxo-2,3,6,7-tetrahydro-1H-purin-1-yl)-2-methylhexanal (3ah)

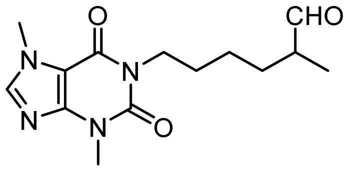

Following the general procedure D, the product was obtained as a yellowish oil, 27.6 mg, 48% yield. **^{1}H NMR** (300 MHz, $CDCl_3$) δ 9.57 (d, *J* = 2.1 Hz, 1H), 7.49 (d, *J* = 0.7 Hz, 1H), 3.98-3.93 (m, 5H), 3.52 (s, 3H), 2.37-2.25 (m, 1H), 1.79-1.58 (m, 3H), 1.46-1.32 (m, 3H), 1.05 (d, *J* = 7.2 Hz, 3H). **^{13}C NMR** (75 MHz, $CDCl_3$) δ 205.05, 155.15, 151.35, 148.64, 141.38, 107.53, 46.09, 40.90, 33.49, 29.96, 29.58, 27.86, 24.17, 13.19. **HRMS** (EI): Calcd. For $C_{14}H_{24}O$ $[M]^+$: 208.1822, Found: 208.1827. **HRMS** (ESI): Calcd. For $[M+H]^+$: 293.1608, Found: 293.1610.

1-(4-Methoxyphenyl)-3-methyloctan-4-one (5c)

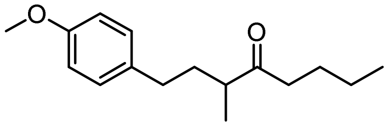

Following the general procedure E, the product was obtained as a colorless oil, 32.6 mg, 60% yield. **^{1}H NMR** (300 MHz, $CDCl_3$) δ 7.08 (d, J = 8.1 Hz, 2H), 6.82 (d, J = 8.7 Hz, 2H), 3.78 (s, 3H), 2.56-2.37 (m, 4H), 2.03-1.91 (m, 1H), 1.64-1.48 (m, 3H), 1.36-1.21 (m, 3H), 1.10 (d, J = 6.9 Hz, 3H), 0.90 (t, J = 7.2 Hz, 3H). **^{13}C NMR** (101 MHz, $CDCl_3$) δ 214.64, 157.81, 133.82, 129.21, 113.78, 55.23, 45.58, 40.89, 34.71, 32.53, 25.78, 22.40, 16.48, 13.89. **HRMS** (EI): Calcd. For $C_{16}H_{24}O_2$ $[M]^+$: 248.1776, Found: 248.1773.

1-(4-Methoxyphenyl)-3-methylundecan-4-one (5d)

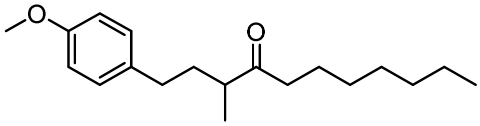

Following the general procedure E, the product was obtained as a colorless oil, 32.6 mg, 52% yield. **^{1}H NMR** (400 MHz, $CDCl_3$) δ 7.09-7.07 (m, 2H), 6.84-6.81 (m, 2H), 3.79 (s, 3H), 2.57-2.47 (m, 3H), 2.45-2.33 (m, 2H), 2.01-1.92 (m, 1H), 1.63-1.51 (m, 3H), 1.34-1.21 (m, 8H), 1.10 (d, J = 7.0 Hz, 3H), 0.92-0.83 (m, 3H). **^{13}C NMR** (101 MHz, $CDCl_3$) δ 214.69, 157.82, 133.83, 129.22, 113.79, 55.23, 45.57, 41.19, 34.71, 32.53, 31.68, 29.26, 29.10, 23.68, 22.60, 16.48, 14.05. **HRMS** (EI): Calcd. For $C_{19}H_{30}O_2$ $[M]^+$: 290.2240, Found: 290.2249.

1-(4-Phenylcyclohexyl)propan-1-one (5e)

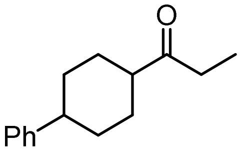

Following the general procedure E, the product was obtained as a colorless oil, 26 mg, yield = 60% (d.r.>20:1). **^{1}H NMR** (400 MHz, $CDCl_3$) δ 7.30-7.26 (m, 2H), 7.21-7.15 (m, 3H), 2.67-2.65 (m, 1H), 2.59 – 2.48 (m, 3H), 2.23 – 2.19 (m, 2H), 1.78-1.62 (m, 6H), 1.08 (t, J = 7.2 Hz, 3H). **^{13}C NMR** (101 MHz, $CDCl_3$) δ 214.03, 147.00, 128.32, 126.94, 125.93, 46.01, 43.53, 33.47, 30.39, 26.96, 8.08. **HRMS** (EI): Calcd. For $C_{15}H_{20}O$ $[M]^+$: 215.1514, Found: 216.1518.

1-(4-Phenylcyclohexyl)butan-1-one (5f)

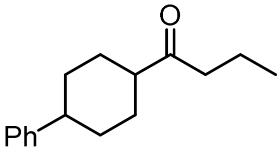

Following the general procedure E, the product was obtained as a colorless oil, 23 mg, 50% yield (d.r.>20:1). **^{1}H NMR** (300 MHz, $CDCl_3$) δ 7.30 – 7.25 (m, 2H), 7.21 – 7.14 (m, 3H), 2.64-2.62 (m, 1H), 2.59 – 2.51 (m, 1H), 2.46 (t, J = 7.2 Hz, 2H), 2.26 – 2.18 (m, 2H), 1.77 – 1.57 (m, 8H), 0.93 (t, J = 7.5 Hz, 3H). **^{13}C NMR** (75 MHz, $CDCl_3$) δ 213.56, 146.96, 128.26, 126.88, 125.87, 46.17, 43.49, 42.28, 30.32, 26.81, 17.34, 13.83. **HRMS** (EI): Calcd. For $C_{16}H_{22}O$ $[M]^+$: 230.1665, Found: 230.1669.

1-(Adamantan-2-yl)pentan-1-one (5g)

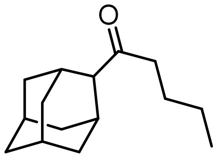

Following the general procedure E, the product was obtained as a colorless oil, 13.2 mg, 30% yield. ^{1}**H NMR** (300 MHz, $CDCl_3$) δ 2.48-2.34 (m, 4H), 2.04-1.71 (m, 11H), 1.61-1.50 (m, 4H), 1.36-1.23 (m, 2H), 0.90 (t, J = 0.6 Hz, 3H). ^{13}**C NMR** (75 MHz, $CDCl_3$) δ 213.18, 57.02, 39.43, 38.45, 37.31, 33.31, 29.21, 27.67, 27.51, 26.02, 22.47, 13.94. **HRMS** (EI): Calcd. For $C_{15}H_{24}O$ $[M]^+$: 220.1822, Found: 220.1826.

1-Cyclopropyl-4-(4-methoxyphenyl)-2-methylbutan-1-one (5h)

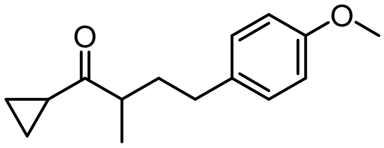

Following the general procedure E, the product was obtained as a colorless oil, 23.3 mg, 50% yield. ^{1}**H NMR** (400 MHz, $CDCl_3$) δ 7.10 (d, J = 8.4 Hz, 2H), 6.83 (d, J = 8.4 Hz, 2H), 3.79 (s, 3H), 2.73-2.64 (m, 1H), 2.55 (t, J = 8.0 Hz, 2H), 2.09-1.99 (m, 1H), 1.97-1.91 (m, 1H), 1.69-1.60 (m, 1H), 1.17 (d, J = 6.8 Hz, 3H), 1.02-0.99(m, 2H), 0.87-0.84 (m, 2H). ^{13}**C NMR** (101 MHz, $CDCl_3$) δ 214.18, 157.78, 133.92, 129.24, 113.76, 55.20, 46.44, 34.81, 32.51, 19.07, 16.28, 10.72, 10.70. **HRMS** (EI): Calcd. For $C_{15}H_{20}O_2$ $[M]^+$: 232.1458, Found: 232.1460.

1-Cyclopentyl-4-(4-methoxyphenyl)-2-methylbutan-1-one (5i)

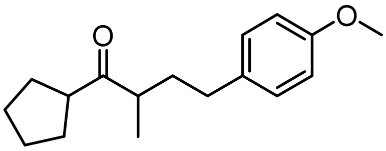

Following the general procedure E, the product was obtained as a colorless oil, 20.3 mg, 50% yield. ^{1}**H NMR** (400 MHz, $CDCl_3$) δ 7.08 (d, J = 8.4 Hz, 2H), 6.82 (d, J = 8.4 Hz, 2H), 3.79 (s, 3H), 3.00-2.92 (m, 1H), 2.69-2.60 m, 1H), 2.55-2.47 (m, 2H), 2.02-1.93 (m, 1H), 1.78-1.66 (m, 6H), 1.61-1.54 (m, 3H), 1.11 (d, J = 6.8 Hz, 3H). ^{13}**C NMR** (101 MHz, $CDCl_3$) δ 217.09, 157.78, 133.93, 129.18, 113.76, 55.23, 49.97, 45.14, 34.79, 32.62, 29.49, 29.33, 26.08, 26.03, 16.67. **HRMS** (ESI): Calcd. For $C_{17}H_{25}O_2^+$ $[M+H]^+$: 261.1849, Found: 261.1850.

Cyclohexyl(4-phenylcyclohexyl)methanone (5j)

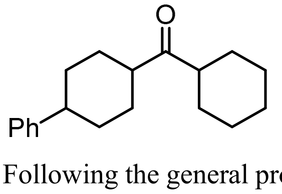

Following the general procedure E, the product was obtained as a colorless oil, 26.5 mg, 49% yield (d.r.>20:1). ^{1}**H NMR** (400 MHz, $CDCl_3$) δ 7.22-7.18 (m, 2H), 7.13-7.07 (m, 3H), 2.72-2.70 (m, 1H), 2.55-2.47 (m, 2H), 2.08-2.04 (m, 2H), 1.71-1.57 (m, 10H), 1.35-1.12 (m, 5H). ^{13}**C NMR** (101 MHz, $CDCl_3$) δ 216.75, 128.25, 126.93, 125.83, 48.21, 44.27, 43.43, 30.30, 28.98, 26.86, 25.85, 25.76. **HRMS** (ESI): Calcd. For $C_{19}H_{30}NO^+$ $[M+NH_4]^+$: 288.2322, Found: 288.2322.

2-Methyl-1-(4-phenylcyclohexyl)pentan-1-one (5k)

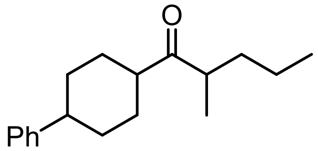

Following the general procedure E, the product was obtained as a colorless oil, 24.3 mg, 47% yield. (d.r.>20:1). **^{1}H NMR** (300 MHz, $CDCl_3$) δ 7.31 – 7.14 (m, 5H), 2.83-2.75 (m, 2H), 2.61-2.52 (m, 1H), 2.16 – 2.09 (m, 2H), 1.81 – 1.60 (m, 7H), 1.33 – 1.19 (m, 3H), 1.06 (d, J = 6.9 Hz, 3H), 0.93 – 0.88 (m, 3H). **^{13}C NMR** (75 MHz, $CDCl_3$) δ 217.77, 146.99, 128.26, 126.94, 125.85, 45.10, 43.37, 43.29, 35.74, 30.32, 30.17, 26.91, 26.57, 20.67, 17.16, 14.20. **HRMS** (EI): Calcd. For $C_{18}H_{26}O$ $[M]^+$: 258.1978, Found: 258.1980.

3-(Benzo[d][1,3]dioxol-5-yl)-1-cyclobutyl-2-methylpropan-1-one (5l)

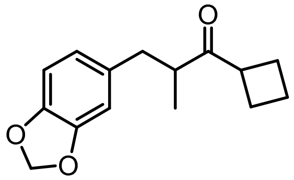

Following the general procedure E, the product was obtained as a colorless oil, 30 mg, 61% yield. **^{1}H NMR** (400 MHz, $CDCl_3$) δ 6.69 (d, J = 7.6 Hz, 1H), 6.61 (d, J = 1.6 Hz, 1H), 6.56 (dd, J = 8.0, 1.7 Hz, 1H), 5.90 (d, J = 0.8 Hz, 2H), 3.28-3.19 (m, 1H), 2.90-2.85 (m, 1H), 2.78-2.70 (m, 1H), 2.46-2.41 (m, 1H), 2.24-2.15 (m, 1H), 2.08-1.83 (m, 4H), 1.77-1.68 (m, 1H), 1.03 (d, J = 7.2 Hz, 3H). **^{13}C NMR** (101 MHz, $CDCl_3$) δ 214.57, 147.46, 145.79, 133.68, 121.82, 109.25, 108.04, 100.73, 46.30, 44.62, 38.79, 24.16, 24.00, 17.61, 16.62. **HRMS** (EI): Calcd. For $C_{15}H_{18}O_3$ $[M]^+$: 246.1251, Found: 246.1246.

3-(Benzo[d][1,3]dioxol-5-yl)-1-cyclopentyl-2-methylpropan-1-one (5m)

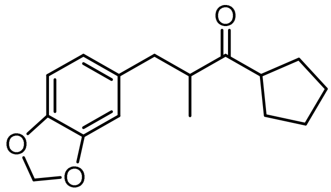

Following the general procedure E, the product was obtained as a colorless oil, 34.9 mg, 67% yield. **^{1}H NMR** (400 MHz, $CDCl_3$) δ 6.69 (d, J = 7.6 Hz, 1H), 6.62-6.56(m, 2H), 5.90 (q, J = 1.6 Hz, 2H), 2.91-2.79 (m, 3H), 2.48-2.42 (m, 1H), 1.72-1.49 (m, 8H), 1.06 (d, J = 6.8 Hz, 3H). **^{13}C NMR** (101 MHz, $CDCl_3$) δ 216.38, 147.45, 145.78, 133.75, 121.87, 109.31, 108.03, 100.73, 50.70, 47.78, 38.98, 28.77, 28.67, 25.96, 25.85, 16.85. **HRMS** (EI): Calcd. For $C_{16}H_{20}O_3$ $[M]^+$: 260.1407, Found: 260.1405.

1-(Benzo[d][1,3]dioxol-5-yl)-2,4-dimethyl-6-(2,6,6-trimethylcyclohex-1-en-1-yl)hexan-3-one (5n)

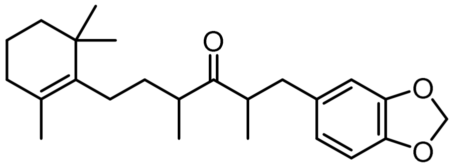

Following the general procedure E, the product was obtained as a colorless oil, 40 mg, 54% yield (d.r. = 2.6:1). **^{1}H NMR** (400 MHz, $CDCl_3$) δ 6.72-6.58 (m, 3H), 5.92-5.88 (m, 2H), 2.92-2.84 (m, 2H), 2.50 - 2.43 (2, 2H), 1.90-1.78 (m, 4H), 1.60-1.51 (m, 6H), 1.40-1.36 (m, 2H), 1.08-1.05 (m, 5H), 0.97-0.87 (m,

8H). **^{13}C NMR** (101 MHz, $CDCl_3$) δ 217.36, 217.16, 147.47, 145.84, 145.82, 136.77, 136.73, 133.80, 133.69, 127.29, 127.16, 121.98, 121.92, 109.41, 109.35, 108.10, 108.07, 100.77, 100.74, 47.64, 47.55, 46.76, 46.71, 39.76, 39.74, 39.00, 38.95, 34.87, 34.80, 33.00, 32.92, 32.68, 28.58, 28.51, 28.48, 28.44, 26.74, 26.62, 19.73, 19.70, 19.46, 17.05, 16.73, 16.06, 15.84. **HRMS** (EI): Calcd. For $C_{24}H_{34}O_3$ $[M]^+$:370.2503, Found: 370.2492.

3-(Benzo[d][1,3]dioxol-5-yl)-1-cycloheptyl-2-methylpropan-1-one (5o)

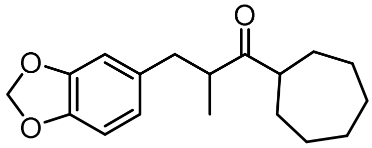

Following the general procedure E, the product was obtained as a colorless oil, 23 mg, 40% yield. **^{1}H NMR** (400 MHz, $CDCl_3$) δ 6.71-6.69 (d, J = 7.6 Hz,1H), 6.63-6.57 (m, 2H), 5.92-5.91 (m, 2H), 2.92-2.83 (m, 2H), 2.49-2.41 (m, 2H), 1.69-1.25 (m, 12H), 1.05 (d, J = 6.4 Hz, 3H). **^{13}C NMR** (101 MHz, $CDCl_3$) δ 217.24, 147.48, 145.82, 133.82, 121.93, 109.38, 108.09, 100.77, 51.79, 47.22, 39.21, 29.59, 29.21, 28.27, 26.63, 26.51, 17.07. **HRMS** (EI): Calcd. For $C_{18}H_{24}O_3$ $[M]^+$: 288.1720, Found: 288.1713.

1-(Benzo[d][1,3]dioxol-5-yl)-6-(4-methoxyphenyl)-2,4-dimethylhexan-3-one (5p)

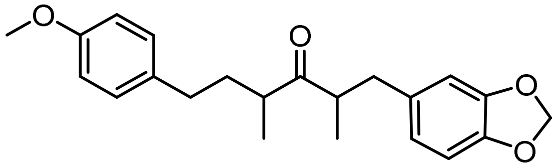

Following the general procedure E, the product was obtained as a colorless oil, 28.3 mg, 40% yield (d.r.=1.3:1). **^{1}H NMR** (300 MHz, $CDCl_3$) δ 7.07 – 6.95 (m, 2H), 6.85 – 6.78 (m, 2H), 6.71 – 6.54 (m, 3H), 5.90 (s, 1H), 5.88 (q, J = 1.5 Hz, 1H), 3.80 (s, 1.3H), 3.78 (s, 1.7H), 2.91 – 2.82 (m, 2H), 2.54 – 2.40 (m, 3H), 2.34 (t, J = 8.1 Hz, 1H), 1.97 – 1.71 (m, 1H), 1.57 – 1.36 (m, 1H), 1.07-1.03 (m, 4.5H), 0.89 (d, J = 6.9 Hz, 1.5H).**^{13}C NMR** (101 MHz, $CDCl_3$) δ 217.28 (s), 217.01, 157.80, 157.75, 147.50, 147.46, 145.88, 145.83, 133.79, 133.73, 133.70, 129.16, 129.08, 122.01, 121.92, 113.76, 113.71, 109.43,109.34, 108.10, 108.06, 100.76, 55.21, 47.52, 47.27, 45.15, 45.11, 38.98, 38.91, 34.16, 32.52, 32.24, 17.07, 16.82, 16.17, 15.81. **HRMS** (ESI): Calcd. For $C_{22}H_{27}O_4^+$ $[M+H]^+$: 355.1904, Found: 355.1906.

1-(Cyclohex-3-en-1-yl)-4-(4-methoxyphenyl)-2-methylbutan-1-one (5q)

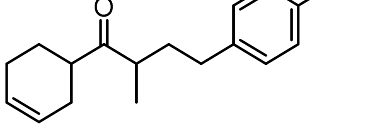

Following the general procedure E, the product was obtained as a colorless oil, 16.1 mg, 50% yield (d.r.=1:1). **^{1}H NMR** (300 MHz, $CDCl_3$) δ 7.10-7.05 (m, 2H), 6.85-6.80 (m, 2H), 5.70-5.69 (m, 2H), 3.79 (s, 3H), 2.79-2.65 (m, 2H), 2.54-2.48 (m, 2H), 2.22-1.93 (m, 5H), 1.88- 1.83 (m, 1H), 1.63-1.52 (m, 2H), 1.12 (d, 1.8 Hz, 1.5H). 1.11 (d, 1.8 Hz, 1.5H). **^{13}C NMR** (75 MHz, CDCl3) δ 217.05, 157.78, 133.86, 133.79, 129.18, 126.52, 126.47, 125.54, 125.47, 113.77, 55.24, 45.54 ,45.34, 44.13, 44.04, 34.92, 34.64, 32.70, 32.64, 27.11, 27.06, 24.85, 24.82, 24.75, 16.87, 16.60. **HRMS** (EI): Calcd. For $C_{18}H_{24}O_2$ $[M]^+$: 272.1771, Found: 272.1764.

Cyclohex-3-en-1-yl(4-phenylcyclohexyl)methanone (5r)

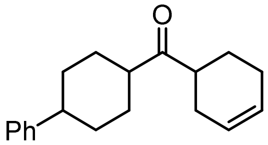

Following the general procedure E, the product was obtained as a colorless oil, 40.2 mg, 75% yield (d.r.>15:1). Following the general procedure A with **4e** (6 mmol), the product was obtained as a colorless oil, 1.126g, 70% yield (d.r.>15:1). **^{1}H NMR**. (400 MHz, $CDCl_3$) δ 7.31 – 7.26 (m, 2H), 7.22 – 7.15 (m, 3H), 5.74-5.67 (m, 2H), 2.90 – 2.83 (m, 2H), 2.61-2.53 (m, 1H), 2.22 – 2.08 (m, 6H), 2.02 – 1.84 (m, 2H), 1.77 – 1.63 (m, 6H). **^{13}C NMR** (75 MHz, $CDCl_3$) δ 216.48, 146.93, 128.26, 126.91, 126.51, 125.87, 125.64, 44.42, 43.91, 43.44, 30.31, 30.28, 27.57, 26.92, 26.82, 25.19, 24.86. **HRMS** (ESI): Calcd. For $C_{19}H_{28}NO^+$ $[M+NH_4]^+$: 286.2165, Found: 286.2171.

8-Chloro-1-(4-methoxyphenyl)-3-methyloctan-4-one (5s)

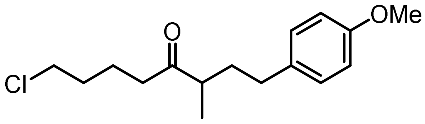

Following the general procedure E, the product was obtained as a colorless oil, 34.3 mg, 59% yield. **^{1}H NMR** (300 MHz, $CDCl_3$) δ 7.10-7.05 (m, 2H), 6.85-6.80 (m, 2H), 3.78 (s, 3H), 3.59-3.49 (m, 2H), 2.59-2.35 (m, 5H), 2.03-1.91 (m, 1H), 1.82-1.65 (m, 4H), 1.64-1.51 (m, 1H), 1.11 (d, *J* = 6.9 Hz, 3H). **^{13}C NMR** (75 MHz, $CDCl_3$) δ 213.79, 157.82, 133.62, 129.21, 113.78, 55.21, 45.53, 44.67, 40.07, 34.62, 32.47, 31.98, 20.90, 16.45. **HRMS** (EI): Calcd. For $C_{16}H_{23}O_2Cl$ $[M]^+$: 282.1381, Found: 282.1386.

10-Hydroxy-1-(4-methoxyphenyl)-3,6,10-trimethylundecan-4-one (5t)

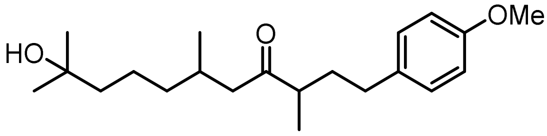

Following the general procedure E, the product was obtained as a colorless oil, 29.4 mg, 44% yield (d.r.=1:1). **^{1}H NMR** (300 MHz, $CDCl_3$) δ 7.10-7.05 (m, 2H), 6.84-6.79 (m, 2H), 3.78 (s, 3H), 2.53-2.48 (m, 2H), 2.46-2.18 (m, 2H), 2.08-1.89 (m, 2H), 1.63-1.49 (m, 2H), 1.46-1.26 (m, 6H), 1.20 (d, *J* = 0.9 Hz, 6H), 1.08 (d, *J* = 6.9 Hz, 3H), 0.88 (d, *J* = 3.0 Hz, 1.5 H), 0.86 (d, *J* = 3.0 Hz, 1.5 H). **^{13}C NMR** (75 MHz, $CDCl_3$) δ 214.34, 214.30, 157.74, 133.77, 133.75, 129.18, 113.81, 113.75, 70.92, 55.19, 48.70, 48.66, 45.88, 45.84, 43.87, 37.32, 37.29, 34.60, 34.46, 32.49, 32.45, 29.27, 29.12, 28.63, 23.82, 21.64, 19.89, 19.83, 16.32, 16.25. **HRMS** (ESI): Calcd. For $C_{21}H_{38}NO_3^+$ $[M+NH_4]^+$: 252.2846, Found: 252.2846.

1,3-Phenylenebis(cyclohexylmethanone) (5u)

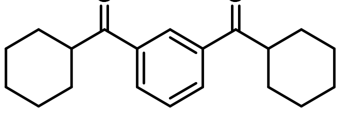

Following the general procedure E, starting from hydrazone (0.4 mmol) and isophthalaldehyde (0.2 mmol), the product was obtained as a white solid, 33.4 mg, 56% yield. **^{1}H NMR** (300 MHz, $CDCl_3$) δ 8.48 (t, *J* = 2.1 Hz, 1H), 8.11 (dd, *J* = 7.8, 1.8 Hz, 2H), 7.56 (t, *J* = 7.8 Hz, 1H), 3.35-3.25 (m, 2H), 1.92-1.82 (m, 8H), 1.77-1.71 (m, 2H), 1.57 – 1.24 (m, 10H). **^{13}C NMR** (75 MHz, $CDCl_3$) δ 203.25, 136.68,

132.20, 128.99, 127.92, 45.69, 29.33, 25.89, 25.74. **HRMS** (EI): Calcd. For $C_{20}H_{26}O_2$ $[M]^+$:298.1927, Found: 298.1920.

(5S,8R,9S,10S,13S,14S,17S)-3-(4-Chlorobenzoyl)-10,13-dimethylhexadecahydro-1H-cyclopenta[a]phenanthren-17-yl acetate (5v)

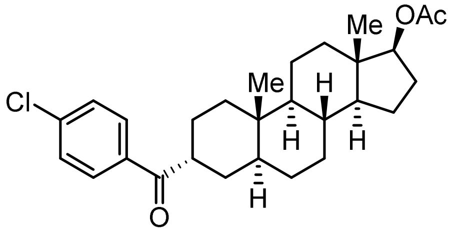

Following the general procedure E, the product was obtained as a white solid, 45.7 mg, 50% yield (d.r.>20:1). **^{1}H NMR** (300 MHz, $CDCl_3$) δ 7.90 – 7.84 (m, 2H), 7.45 – 7.39 (m, 2H), 4.62-4.56 (m, 1H), 3.29 – 3.17 (m, 1H), 2.21 – 2.08 (m, 1H), 2.03 (s, 3H), 1.84 – 1.40 (m, 12H), 1.38 – 1.23 (m, 5H), 1.14 – 0.91 (m, 4H), 0.83 (s, 3H), 0.77 (s, 3H). **^{13}C NMR** (75 MHz, $CDCl_3$) δ 202.44, 171.24, 139.11, 134.59, 129.65, 128.87, 82.80, 54.43, 50.75, 46.30, 46.03, 42.57, 37.89, 36.89, 36.04, 35.21, 31.54, 28.53, 27.46, 24.76, 23.45, 21.17, 20.42, 12.29, 12.08. **HRMS** (ESI) Calcd. For $[M+H]^+$:457. 2504, Found 457.251.

Methyl 4-(cyclohexanecarbonyl)benzoate (5w)

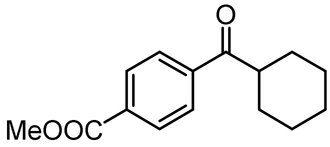

Following the general procedure E, the product was obtained as a white solid, 26.1 mg, 53% yield. **^{1}H NMR** (300 MHz, $CDCl_3$) δ 8.13-8.09 (m, 2H), 7.99-7.95 (m, 2H), 3.94 (s, 3H), 3.30-3.20 (m, 1H), 1.92-1.70 (m, 5H), 1.52-1.27 (m, 5H). **^{13}C NMR** (75 MHz, $CDCl_3$) δ 203.38, 166.28, 139.69, 133.49, 129.80, 128.12, 52.42, 45.96, 29.23, 25.86, 25.73. **HRMS** (EI): Calcd. For $C_{15}H_{18}O_3$ $[M]^+$:246.1256, Found: 246.1255. Spectroscopic results agree with preciously reported data.[8]

Cyclohexyl(4-chlorophenyl)methanone (5x)

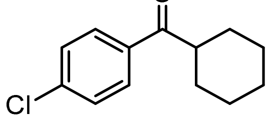

Following the general procedure E, the product was obtained as a white solid, 24.4 mg, 55% yield. **^{1}H NMR** (400 MHz, $CDCl_3$) δ 7.90-7.86 (m, 2H), 7.44-7.41 (m, 2H), 3.23-3.16 (m, 1H), 1.90 -1.82 (m, 4H), 1.77-1.70 (m, 1H), 1.51-1.21 (m, 6H). **^{13}C NMR** (101 MHz, $CDCl_3$) δ 202.58, 139.10, 134.61, 129.68, 128.87, 45.62, 29.34, 25.88, 25.78. **HRMS** (EI): Calcd. For $C_{13}H_{15}OCl$ $[M]^+$:222.0806, Found: 222.0805. Spectroscopic results agree with preciously reported data.[8]

Cyclohexyl (2-fluoro-3-methoxyphenyl) methanone (5y)

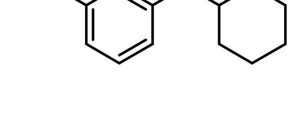

Following the general procedure E, the product was obtained as a white solid, 34.4 mg, 73% yield. **^{1}H NMR** (300 MHz, $CDCl_3$) δ 7.37-7.32 (m, 1H), 7.23-7.17 (m, 2H), 4.02 (s, 3H), 3.26-3.16 (m, 1H), 2.09 -2.02 (m, 2H), 1.94-1.75 (m, 3H), 1.58-1.33 (m, 5H). **^{13}C NMR** (75 MHz, $CDCl_3$) δ 203.05 (d, *J* = 3.5 Hz), 151.10 (d, *J* = 253.0 Hz), 148.12 (d, *J* = 11.9 Hz), 127.04 (d, *J* = 11.5 Hz), 124.14 (d, *J* = 4.4 Hz), 121.23 (d, *J* = 2.4 Hz), 116.39 (d, *J* = 2.5 Hz), 56.52, 50.30 (d, *J* = 5.9 Hz), 28.79, 26.01, 25.82. **^{19}F NMR** (282 MHz, $CDCl_3$) δ -135.60. **HRMS** (EI): Calcd. For $C_{14}H_{17}O_2F$ $[M]^+$: 236.1207, Found 236.1206.

cyclohexyl(pyridin-3-yl)methanone (5z)

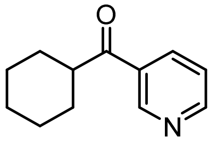

Following the general procedure E, the product was obtained as a colorless oil, 15 mg, 40% yield. **^{1}H NMR (400 MHz, Chloroform-*d*)** δ 9.14 (s, 1H), 8.75 (d, *J* = 4.2 Hz, 1H), 8.20 (d, *J* = 8.4 Hz, 1H), 7.42 – 7.39 (m, 1H), 3.25-3.19 (m, 1H), 1.91 – 1.73 (m, 5H), 1.55-1.25 (m, 5H). **^{13}C NMR (101 MHz, Chloroform-*d*)** δ 202.55, 153.15, 149.70, 135.68, 131.49, 123.66, 46.06, 29.13, 25.82, 25.68. **HRMS (ESI):** $[M+H]^+$ calcd for $C_{12}H_{16}NO$ 190.1226, found 190.1230.

(4-(allyloxy)phenyl)(cyclohexyl)methanone (5aa)

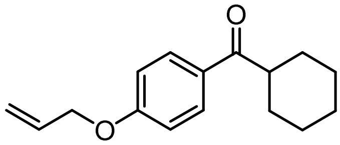

Following the general procedure E, the product was obtained as a colorless oil, 17.1 mg, 35% yield. **^{1}H NMR (400 MHz, Chloroform-*d*)** δ 7.93 (d, *J* = 8.4 Hz, 2H), 6.94 (d, *J* = 8.4 Hz, 2H), 6.10-6.00 (m, 1H), 5.42 (d, *J* = 17.2 Hz, 1H), 5.31 (d, *J* = 10.4 Hz, 1H), 4.60 (d, *J* = 5.2 Hz, 2H), 3.25-3.18 (m, 1H), 1.88 – 1.71 (m, 5H), 1.54 – 1.24 (m, 5H). **^{13}C NMR (101 MHz, Chloroform-*d*)** δ 202.41, 162.19, 132.54, 130.45, 129.35, 118.13, 114.40, 68.84, 45.29, 29.53, 25.96, 25.89. **HRMS (ESI):** $[M+H]^+$ calcd for $C_{16}H_{21}O_2$ 245.1536, found 245.1542.

(4-(but-2-yn-1-yloxy)phenyl)(cyclohexyl)methanone (5ab)

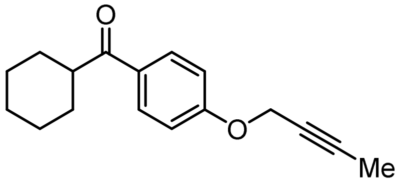

Following the general procedure E, the product was obtained as a colorless oil, 244.6 mg, 48% yield. **^{1}H NMR (400 MHz, Chloroform-*d*)** δ 7.93 (d, *J* = 8.4 Hz, 2H), 6.99 (d, *J* = 8.4 Hz, 2H), 4.70 (s, 2H), 3.24-3.18 (m, 1H), 1.88 – 1.70 (m, 8H), 1.54 – 1.21 (m, 5H). **^{13}C NMR (101 MHz, Chloroform-*d*)** δ 202.39, 161.35, 130.36, 129.65, 114.52, 84.35, 73.32, 56.46, 45.30, 29.50, 25.94, 25.87. **HRMS (ESI):** $[M+H]^+$ calcd for $C_{17}H_{21}O_2$ 257.1536, found 257.1534.

cyclohexyl(4-(pyridin-2-ylmethoxy)phenyl)methanone (5ac)

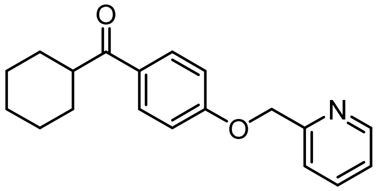

Following the general procedure E, the product was obtained as a colorless oil, 30 mg, 51% yield. **^{1}H NMR (400 MHz, Chloroform-*d*)** δ 8.65 (d, *J* = 35.2 Hz, 2H), 7.94 (d, *J* = 8.4 Hz, 2H), 7.76 (d, *J* = 7.6 Hz, 1H), 7.34 (s, 1H), 7.00 (d, *J* = 8.4 Hz, 2H), 5.13 (s, 2H), 3.24-3.17 (m, 1H), 1.87 – 1.70 (m, 5H), 1.53 – 1.22 (m, 5H). **^{13}C NMR (101 MHz, Chloroform-*d*)** δ 201.38, 160.88, 134.35, 129.57, 128.86, 113.50, 66.67, 44.35, 28.51, 24.95, 24.88. **HRMS (ESI):** [M+H]$^+$ calcd for $C_{19}H_{22}NO_2$ 296.1645, found 296.1650.

(4-Chlorophenyl)(cyclododecyl)methanone (5ad)

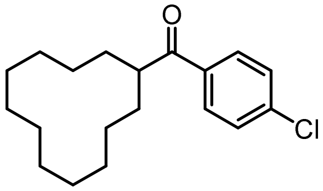

Following the general procedure E, the product was obtained as a white solid, 43.4 mg, 71% yield. **^{1}H NMR** (300 MHz, $CDCl_3$) δ 7.90-7.86 (m, 2H), 7.46-7.42 (m, 2H), 3.52-3.44 (m, 1H), 1.74-1.57 (m, 4H), 1.47-1.25 (m, 18H). **^{13}C NMR** (75 MHz, $CDCl_3$) δ 202.99, 139.07, 135.37, 129.60, 128.92, 42.19, 26.41, 23.94, 23.67, 23.37, 22.47. **HRMS** (EI): Calcd. For $C_{19}H_{27}OCl$ $[M]^+$:306.1744, Found: 306.1734.

1-(4-Chlorophenyl)-4-(4-methoxyphenyl)-2-methylbutan-1-one (5ae)

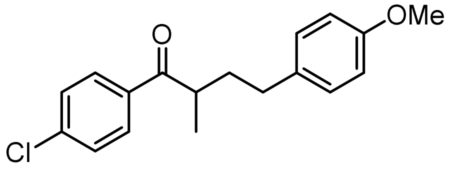

Following the general procedure E, the product was obtained as a white solid, 24.4 mg, 49% yield. **^{1}H NMR** (300 MHz, $CDCl_3$) δ 7.80 – 7.75 (m, 2H), 7.42 – 7.38 (m, 2H), 7.07 – 7.02 (m, 2H), 6.84 – 6.79 (m, 2H), 3.79 (s, 3H), 3.44-3.33 (m, 1H), 2.61-2.56 (m, 2H), 2.18-2.05 (m, 1H), 1.76 – 1.64 (m, 1H), 1.21 (d, *J* = 6.9 Hz, 3H). **^{13}C NMR** (75 MHz, $CDCl_3$) δ 202.88, 157.87, 139.25, 134.75, 133.54, 129.67, 129.37, 128.87, 113.78, 55.24, 39.53, 35.30, 32.42, 17.10. **HRMS** (EI) Calcd. For $C_{18}H_{19}O_2Cl$ $[M]^+$:302.1074, Found: 302.1075.

1-(4-Chlorophenyl)-2-methyldecan-1-one (5af)

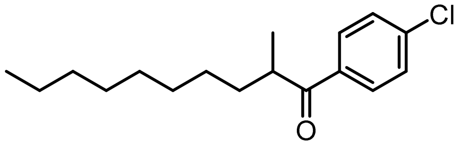

Following the general procedure E, the product was obtained as a white solid, 25.8 mg, 46% yield. **^{1}H NMR** (300 MHz, $CDCl_3$) δ 7.91-7.86 (m, 2H), 7.46-7.41 (m, 2H), 3.45-3.34 (m, 1H), 1.83-1.72 (m, 1H), 1.48-1.37 (m, 1H), 1.30-1.23 (m, 12H), 1.18 (d, *J* = 6.6 Hz, 3H), 0.89-0.84 (m, 3H). **^{13}C NMR** (75 MHz, $CDCl_3$) δ 203.32, 139.19, 135.02, 129.66, 128.90, 40.61, 33.67, 31.82, 29.69, 29.41, 29.22, 27.35, 22.63, 17.11, 14.08. **HRMS** (EI) Calcd. For $C_{17}H_{25}OCl$ $[M]^+$:280.1588, Found: 280.1591.

2-Cyclopropyl-1-phenylpropan-1-one (5ag)

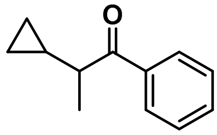

Following the general procedure E, the product was obtained as a colorless oil, 18.2 mg, 52% yield. **^{1}H NMR** (400 MHz, $CDCl_3$) δ 7.93 – 7.01 (M, 2H), 7.58 – 7.53 (M, 1H), 7.48 – 7.44 (m, 2H), 2.80 (dq, J = 7.0, 7.6 Hz, 1H), 1.28 (d, J = 7.0 Hz, 3H), 1.12 – 1.03 (m, 1H), 0.59 – 0.53 (m, 1H), 0.50 – 0.44 (m, 1H), 0.23 – 0.14 (m, 2H). **^{13}C NMR** (101 MHz, $CDCl_3$) δ 204.14, 136.95, 132.81, 128.55, 128.30, 45.11, 17.12, 14.58, 4.35, 3.41. This compound is commerically available (CAS number: 69096-84-0).

1-(Benzo[*d*][1,3]dioxol-5-yl)-2,5-dimethyloctan-3-one (5ah)

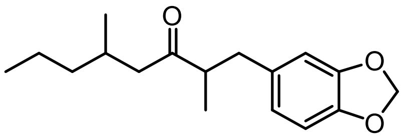

Following the general procedure F, the product was obtained as a colorless oil, 35.1 mg, 64% yield (d.r. = 1:1). Purification was achieved by silica column chromatography (PE/EtOAc = 30:1). **^{1}H NMR** (400 MHz, $CDCl_3$) δ 6.70 (d, *J* = 7.9 Hz, 1H), 6.63 (d, *J* = 1.6 Hz, 1H), 6.58 (dd, *J* = 7.9, 1.6 Hz, 1H), 5.91 (s, 2H), 2.88 (dd, *J* = 13.5, 7.3 Hz, 1H), 2.75 (dqd, *J* = 14.1, 7.0, 2.5 Hz, 1H), 2.45 (ddd, J = 13.5, 7.3, 2.9 Hz, 1H), 2.30 (ddd, *J* = 31.7, 16.4, 5.8 Hz, 1H), 2.15 (ddd, *J* = 30.8, 16.4, 7.7 Hz, 1H), 2.01 – 1.89 (m, 1H), 1.32 – 1.07 (m, 4H), 1.05 (d, *J* = 6.9 Hz, 3H), 0.85 (td, *J* = 7.2, 2.2 Hz, 3H), 0.80 (dd, *J* = 6.6 1.5 Hz, 3H). **^{13}C NMR** (101 MHz, $CDCl_3$) δ 214.2, 214.2, 147.7, 147.7, 146.0, 146.0, 133.8, 133.8, 122.1, 109.5, 109.5, 108.3, 100.9, 49.8, 49.8, 48.7, 48.7, 39.3, 39.3, 38.9, 38.8, 28.7, 28.6, 20.2, 20.2, 20.0, 16.6, 14.3, 14.3. **HRMS** (ESI) Calcd. For $[M+H]^+$: 277.1798, Found 277.1799.

12-Hydroxy-4,8,12-trimethyltridecan-6-one (5ai)

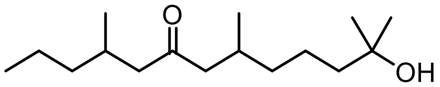

Following the general procedure F, the product was obtained as a colorless oil, 37.0 mg, 72% yield (d.r. = 1:1). Purification was achieved by silica column chromatography (PE/EtOAc = 4:1). **^{1}H NMR** (400 MHz, $CDCl_3$) δ 2.38 – 2.32 (m, 2H), 2.22 – 2.14 (m, 2H), 2.07 – 1.94 (m, 2H), 1.65 (br, 1H), 1.46 – 1.11 (m, 16H), 0.89 – 0.86 (m, 9H). **^{13}C NMR** (101 MHz, $CDCl_3$) δ 211.3, 211.2, 71.1, 51.1, 51.1, 51.0, 51.0, 44.1, 39.4, 39.3, 37.5, 37.5, 29.5, 29.4, 29.2, 29.2, 29.1, 29.0, 21.8, 21.8, 20.2, 20.2, 20.0, 20.0, 20.0, 14.3. **HRMS** (ESI) Calcd. For $[M+NH4]^+$: 274.2741, Found 274.274.

1-(2-Fluoro-3-methoxyphenyl)-3-methylhexan-1-one (5aj)

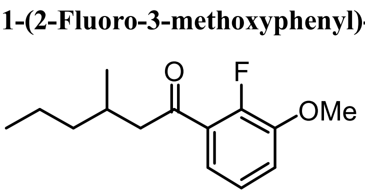

Following the general procedure F, the product was obtained as a colorless oil, 14.2 mg, 30% yield. Purification was achieved by silica column chromatography (PE/EtOAc = 9:1).

^{1}H NMR (400 MHz, $CDCl_3$) δ 7.34 – 7.30 (m, 1H), 7.15 – 7.08 (m, 2H), 3.91 (s, 3H), 2.96 (ddd, *J* =

16.1, 5.6, 2.9 Hz, 1H), 2.74 (ddd, J = 16.1, 8.0, 2.9 Hz, 1H), 2.18 – 2.06 (m, 1H), 1.39 – 1.17 (m, 4H), 0.93 (d, J = 6.7 Hz, 3H), 0.88 (t, J = 7.0 Hz, 3H). **^{13}C NMR** (101 MHz, $CDCl_3$) δ 214.2, 214.2, 147.7, 147.7, 146.0, 146.0, 133.8, 133.8, 122.1, 109.5, 109.5, 108.3, 100.9, 49.8, 49.8, 48.7, 48.7, 39.3, 39.3, 38.9, 38.8, 28.7, 28.6, 20.2, 20.2, 20.0, 16.6, 14.3. **^{19}F NMR** (377 MHz, $CDCl_3$) δ -133.93. **HRMS** (EI+) Calcd. For $[M]^+$: 238.13636, Found 238.13622.

1-(Benzo[*d*][1,3]dioxol-5-yl)-10-hydroxy-2,6,10-trimethylundecan-4-one (5ak)

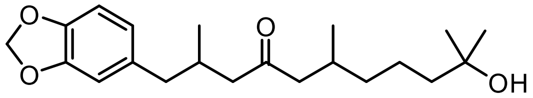

Following the general procedure F, the product was obtained as a colorless oil, 42.2 mg, 61% yield (d.r. = 1:1). Purification was achieved by silica column chromatography (PE/EtOAc = 5:2).
^{1}H NMR (400 MHz, $CDCl_3$) δ 6.71 (d, J = 7.9 Hz, 1H), 6.65 (d, J = 1.5 Hz, 1H), 6.58 (d, J = 7.9, 1.5 Hz, 1H), 5.91 (s, 2H), 2.48 (dd, J = 13.8, 6.2 Hz, 1H), 2.41 – 2.28 (m, 3H), 2.28 – 2.11 (m, 3H), 2.03 – 1.93 (m, 1H), 1.71 (br, 1H), 1.45 – 1.09 (m, 12H), 0.90 – 0.84 (m, 6H). **^{13}C NMR** (101 MHz, $CDCl_3$) δ 210.8, 210.8, 147.6, 145.9, 134.4, 122.1, 109.6, 108.1, 100.9, 71.06, 51.0, 51.0, 50.0, 49.9, 44.0, 43.0, 43.0, 37.5, 37.4, 31.3, 31.3, 29.5, 29.3, 29.2, 29.1, 21.8, 21.8, 20.0, 20.0, 19.9, 19.9. **HRMS** (ESI) Calcd. For $[M+H]^+$:349.2373, Found 349.237.

4-(Benzo[*d*][1,3]dioxol-5-yl)-1-(cyclohex-3-en-1-yl)-3-methylbutan-1-one (5al)

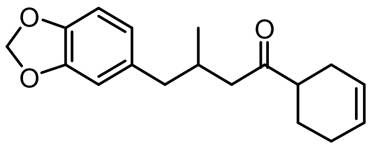

Following the general procedure F, the product was obtained as a colorless oil, 29.2 mg, 51% yield (d.r. = 1:1). Purification was achieved by silica column chromatography (PE/EtOAc = 15:1).
^{1}H NMR (400 MHz, $CDCl_3$) δ 6.72 (d, J = 7.9 Hz, 1H), 6.66 (d, J = 1.3 Hz, 1H), 6.59 (d, J = 7.9, 1.3 Hz, 1H), 5.91 (s, 2H), 5.73 – 5.61 (m, 2H), 2.59 – 2.46 (m, 2H), 2.46 – 2.25 (m, 4H), 2.25 – 1.99 (m, 4H), 1.95 – 1.83 (m, 1H), 1.60 – 1.45 (m, 1H), 0.88 (d, J = 6.2 Hz, 3H). **^{13}C NMR** (101 MHz, $CDCl_3$) δ 213.4, 213.4, 147.6, 145.9, 134.5, 134.5, 126.8, 126.7, 125.6, 125.5, 122.1, 109.6, 108.1, 100.9, 47.4, 47.4, 47.1, 47.1, 43.0, 43.0, 31.2, 31.2, 27.0, 26.8, 24.9, 24.9, 24.8, 24.6, 19.9. **HRMS** (ESI) Calcd. For $[M+H]^+$: 287.1642, Found 287.1641.

2,2,6,10-Tetramethylundec-9-en-4-one (5am)

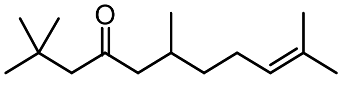

Following the general procedure F, the product was obtained as a colorless oil, 22.6 mg, 50% yield. Purification was achieved by silica column chromatography (PE/EtOAc = 50:1).
^{1}H NMR (400 MHz, $CDCl_3$) δ 5.10 – 5.06 (m, 1H), 2.39 – 2.16 (m, 4H), 2.04 – 1.90 (m, 3H), 1.68 (s, 3H), 1.59 (s, 3H), 1.35 – 1.25 (m, 1H), 1.21 – 1.12 (m, 1H), 1.01 (s, 9H), 0.88 (d, J = 6.6 Hz, 3H). **^{13}C NMR** (101 MHz, $CDCl_3$) δ 211.0, 131.6, 124.6, 55.6, 52.7, 37.1, 31.1, 29.9, 29.0, 25.9, 25.6, 19.9,

17.8. **HRMS** (ESI) Calcd. For $[M+H]^+$: 225.2213, Found 225.2213.

1-(Benzo[*d*][1,3]dioxol-5-yl)-2,5,5-trimethylhexan-3-one (5an)

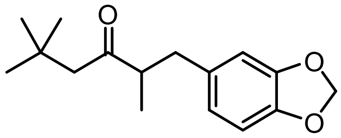

Following the general procedure F, the product was obtained as a colorless oil, 25.4 mg, 48% yield. Purification was achieved by silica column chromatography (PE/EtOAc = 30:1).

^{1}H NMR (400 MHz, $CDCl_3$) δ 6.71 (d, J = 7.8 Hz, 1H), 6.63 (d, J = 1.6 Hz, 1H), 6.58 (dd, J = 7.8, 1.6 Hz, 1H), 5.91 (s, 2H), 2.87 (dd, J = 13.5, 6.9 Hz, 1H), 2.72 (dq, J = 13.9, 7.0 Hz 1H), 2.42 (dd, J = 13.5, 7.5 Hz, 1H), 2.24 (dd, J = 34.9, 15.4 Hz, 2H), 1.02 (d, J = 6.9 Hz, 3H), 0.96 (s, 9H). **^{13}C NMR** (101 MHz, $CDCl_3$) δ 214.1, 147.7, 146.0, 133.8, 122.1, 109.5, 108.3, 100.9, 54.5, 49.8, 38.8, 31.1, 29.8, 16.3. **HRMS** (ESI) Calcd. For $[M+H]^+$: 263.1642, Found 263.1641.

1-(4-Methoxyphenyl)-3,5-dimethyl-7-(2,6,6-trimethylcyclohex-1-en-1-yl)-heptan-4-one (5ao)

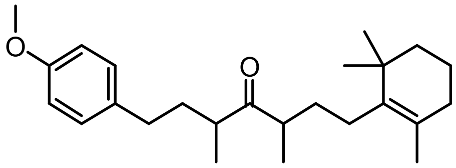

Following the general procedure G using dihydro-β-inone (0.4 mmol), the product was obtained as a colorless oil, 43.5 mg, 29% yield over 2 steps (d.r.=3.9:1). Following the general procedure F using 4-(4-methoxyphenyl)butan-2-one (0.4 mmol), the product was obtained as a colorless oil, 38.3 mg, 26% yield over 2 steps (d.r.=3.9:1). **^{1}H NMR** (400 MHz, $CDCl_3$) δ 7.12 - 7.05 (m, 2H), 6.85 – 6.79 (m, 2H), 3.78 (s, 2.4H), 3.78 (s, 0.6H), 2.70 – 2.60 (m, 1H), 2.59 – 2.46 (m, 2H), 2.09 – 1.81 (m, 5H), 1.63 – 1.52 (m, 8H), 1.43 – 1.26 (m, 4H), 1.15 – 1.03 (m, 5H), 1.02 – 0.94 (m, 6H). **^{13}C NMR** (101 MHz, $CDCl_3$) δ 217.75, 217.65, 157.91, 157.81, 136.77, 133.91, 129.20, 129.18, 129.11, 127.37, 127.34, 113.88, 113.80, 113.79, 55.24, 45.88, 45.83, 44.67, 44.63, 39.78, 34.91, 34.61, 34.56, 33.50, 33.43, 32.72, 32.65, 32.59, 28.61, 28.56, 28.53, 26.86, 26.82, 19.78, 19.75, 19.53, 19.49, 16.61, 16.55, 16.51, 16.43. **HRMS** (ESI) Calcd. For $[M+NH_4]^+$:388.3210, Found 388.3209.

2-(4-(4-Methoxyphenyl)butan-2-yl)hexanal (3ai)

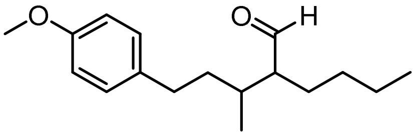

Following the general procedure G using (4-methoxyphenyl)butan-2-one (0.4 mmol), the product was obtained as a colorless oil, 27.7 mg, 26% yield over 2 steps (d.r.=1.3:1). **^{1}H NMR** (400 MHz, $CDCl_3$) δ 9.63 (d, J = 3.2 Hz, 0.4 H), 9.60 (d, J = 2.8 Hz, 0.5 H), 7.08 (d, J = 8.4 Hz, 2H), 6.83 (d, J = 8.8 Hz, 2H), 3.79 (s, 3H), 2.70-2.61 (m, 1H), 2.55-2.46 (m, 1H), 2.21-2.14 (m, 1H), 1.96-1.82 (m, 1H), 1.74-1.60 (m, 2H), 1.55-1.36 (m, 2H), 1.32- 1.25 (m, 4H), 1.02 (d, J = 6.8 Hz, 1.3 H), 0.95 (d, J = 6.8 Hz, 1.7 H), 0.88 (t, J = 7.2 Hz, 3H). **^{13}C NMR** (101 MHz, $CDCl_3$) δ 206.01, 205.75, 157.78, 134.12, 134.07, 129.17, 113.81, 56.87, 56.79, 55.24, 36.52, 36.15, 33.15, 32.60, 32.44, 32.36, 30.10, 29.92, 25.95, 24.55, 22.83, 22.75, 16.83, 16.25, 13.89. **HRMS** (EI) Calcd. For $C_{17}H_{26}O_2[M]^+$:262.1933, Found 262.1926.

4-(4-Methoxyphenyl)-2-methylbutanal-1,2-d$_2$

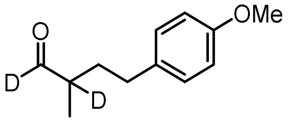

To a 5 mL snap vial with a magnetic stirring bar, tosylhydrazone (**1t**, 0.2 mmol, 1.0 eq.), Cs_2CO_3 (0.3 mmol, 1.5 eq.), and paraformaldehyde-d2 (**2b,** 0.24 mmol, 1.2 eq.) were added. The vial was evacuated and back filled with N_2 for three times. Then dry MeCN (2 mL) was added by syringe, and the mixture was irradiated with a 385 nm LED at 25 °C. After 20 h, the mixture was quenched with water and extracted with Et_2O (10 mL *3). The combined organic phase was then washed with H_2O (10 mL) and brine, dried over sodium sulfate, concentrated under vacuum. The residue was purified by silica gel flash chromatography (gradient eluent: petroleum ether in EtOAc =2% to 3%), to give the desired product as a colorless oil, 24.8 mg, 64% yield, overall deuterium content >98%. **^{1}H NMR** (400 MHz, $CDCl_3$) δ 7.12-7.08 (m, 2H), 6.85-6.82 (m, 2H), 3.79 (s, 3H), 2.67-2.55 (m, 2H), 2.40-2.32 (m, 0.03H), 2.06-1.98 (m, 1H), 1.661.59 (m, 1H), 1.13 (m, 3H). **^{13}C NMR** (101 MHz, $CDCl_3$) δ 204.61 (t, J = 26.0 Hz), 157.91, 133.34, 129.25, 113.84, 55.20, 32.16 (d, J = 19.9 Hz), 31.74, 13.19.

HRMS (EI): Calcd. For $C_{12}H_{14}O_2D_2$ $[M]^+$: 194.1276, Found: 194.1270.

1,2-Diphenylspiro[2.5]octane (8)

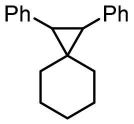

To a 5 mL snap vial with a magnetic stirring bar, tosylhydrazone (**1a**, 0.2 mmol, 1.0 eq.), and Cs_2CO_3 (0.3 mmol, 1.5 eq.) were added. The vial was evacuated and back filled with N_2 for three times. Then a solution of E-stilbene (0.4 mmol, 2.0 eq.) in MeCN (2 mL) was added by syringe, and the mixture was irradiated with a 385 nm LED at 25 ^oC. After 20 h, the mixture was quenched with water and extracted with Et_2O (10 mL *3). The combined organic phase was then washed with H_2O (10 mL) and brine, dried over sodium sulfate, concentrated under vacuum. The residue was purified by silica gel flash chromatography (gradient eluent: petroleum ether in EtOAc =2% to 3%), to give the desired product as a colorless oil, 23 mg, yield = 24%. **^{1}H NMR** (400 MHz, $CDCl_3$) δ 7.20 – 7.12 (m, 5H), 2.42 (s, 2H), 1.41 – 1.29 (m, 4H), 1.23 (dd, J = 6.7, 4.2 Hz, 4H). **^{13}C NMR** (101 MHz, $CDCl_3$) δ 139.14, 128.99, 127.90, 125.75, 33.72, 33.52, 32.45, 26.22, 25.16. **HRMS** (EI) calcd for $C_{20}H_{22}$ $[M]^+$:262.1722 found: 262.1718 *Note: The product was contaminated with cis-stilbene, the yield has been calculated taking this into account.*

7. X-ray characterization data

X-ray crystal structure of **5v** (CCDC 2210656)

The XRD data of **5v** indicated that the 4-chloro-benzoyl group is anti to the methyl group in 4-position of the ketone.The detail of the obtained data is available as a crystallographic information file (CIF), which is available from CCDC (2210656). The crystal data are shown in Table S9.

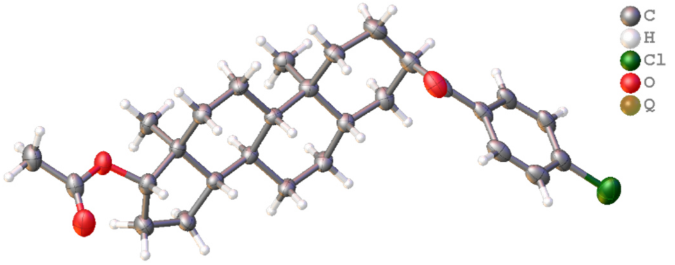

Table S9. Crystal data and structure refinement for **5v**

Crystal Data. $C_{28}H_{37}O_3Cl$, M_r = 457.02, orthorhombic, $P2_12_12_1$ (No. 19), a = 7.1817(2) Å, b = 10.2410(2) Å, c = 33.2457(6) Å, $a = b = g = 90^\circ$, V = 2445.15(9) Å^3, T = 123.00(10) K, Z = 4, Z' = 1, m(Cu K_a) = 1.586, 21249 reflections measured, 4307 unique (R_{int} = 0.0589) which were used in all calculations. The final wR_2 was 0.1117 (all data) and R_1 was 0.0397 (I≥2 s(I)).

**Compound**	**V106**
Formula	$C_{28}H_{37}O_3Cl$
$D_{calc.}$/ g cm^{-3}	1.241
m/mm^{-1}	1.586
Formula Weight	457.02
Colour	clear colourless
Shape	block-shaped
Size/mm^3	0.14×0.11×0.09
T/K	123.00(10)
Crystal System	orthorhombic
Flack Parameter	-0.008(19)
Hooft Parameter	-0.017(8)
Space Group	$P2_12_12_1$
a/Å	7.1817(2)
b/Å	10.2410(2)
c/Å	33.2457(6)
a/°	90
b/°	90
g/°	90
V/Å^3	2445.15(9)
Z	4
Z'	1
Wavelength/Å	1.54184
Radiation type	Cu K_a
Q_{min}/°	4.518
Q_{max}/°	66.899
Measured Refl's.	21249
Indep't Refl's	4307
Refl's I≥2 *s*(I)	3978
R_{int}	0.0589
Parameters	292
Restraints	0
Largest Peak	0.165
Deepest Hole	-0.329
GooF	1.100
wR_2 (all data)	0.1117
wR_2	0.1088
R_1 (all data)	0.0435
R_1	0.0397

8. Copies of NMR spectra

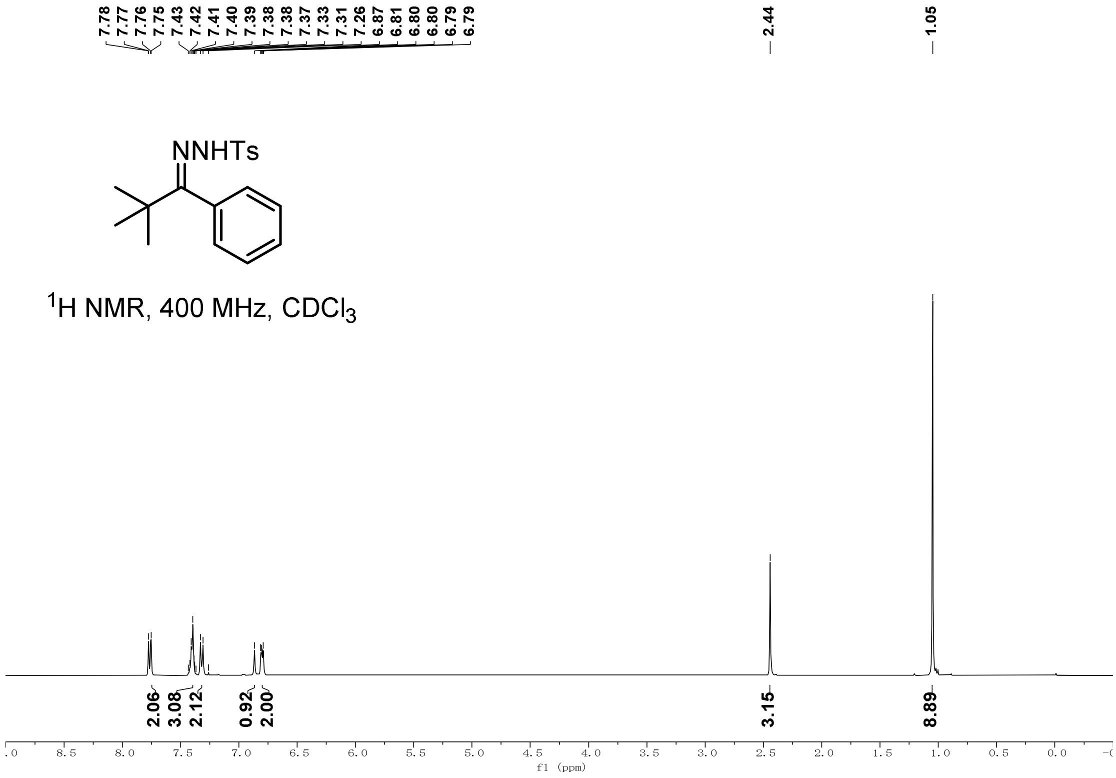

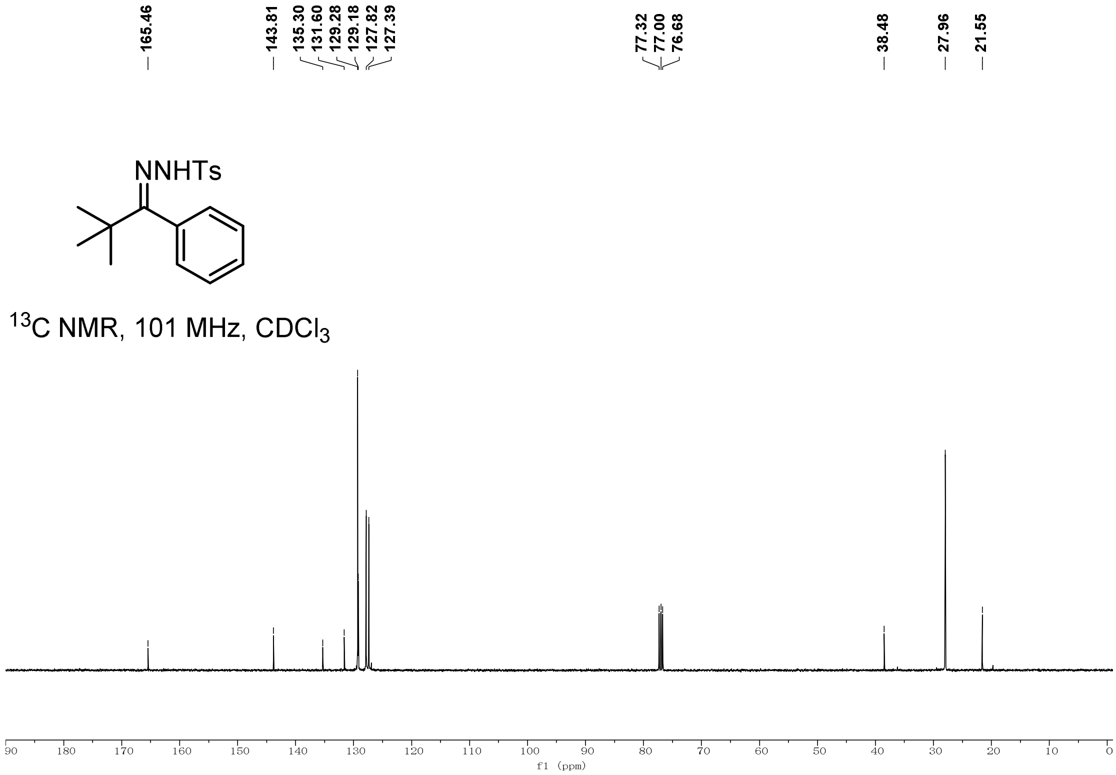

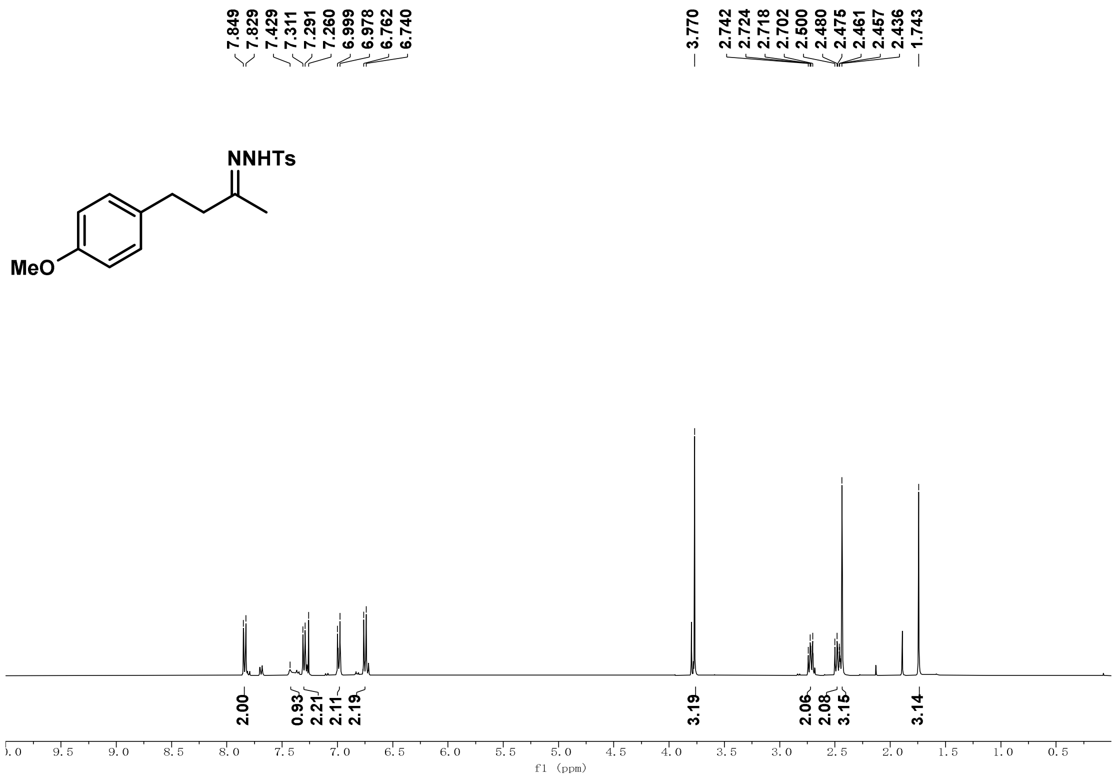
7.849
7.829
7.429
7.311
7.291
7.260
6.999
6.978
6.762
6.740
3.770
2.742
2.724
2.718
2.702
2.500
2.480
2.475
2.461
2.457
2.436
1.743
NNHTs
MeO
2.00
0.93
2.21
2.11
2.19
3.19
2.06
2.08
3.15
3.14
f1 (ppm)

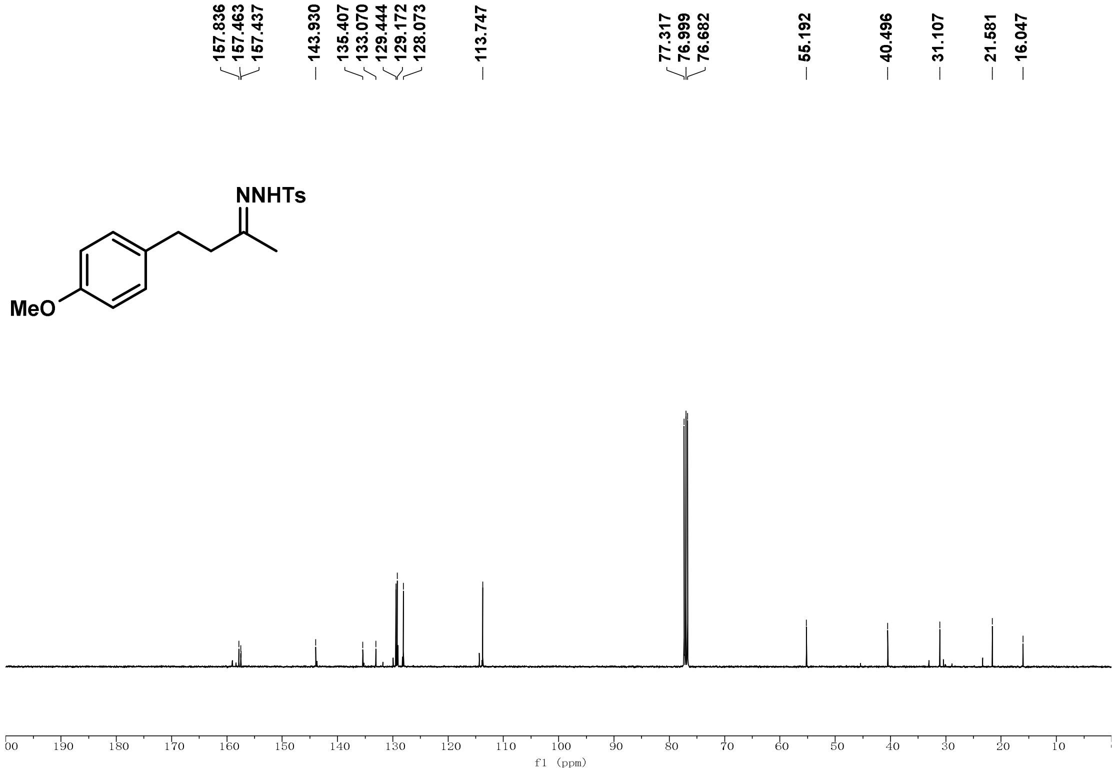
157.836
157.463
157.437
143.930
135.407
133.070
129.444
129.172
128.073
113.747
77.317
76.999
76.682
55.192
40.496
31.107
21.581
16.047
NNHTs
MeO
f1 (ppm)

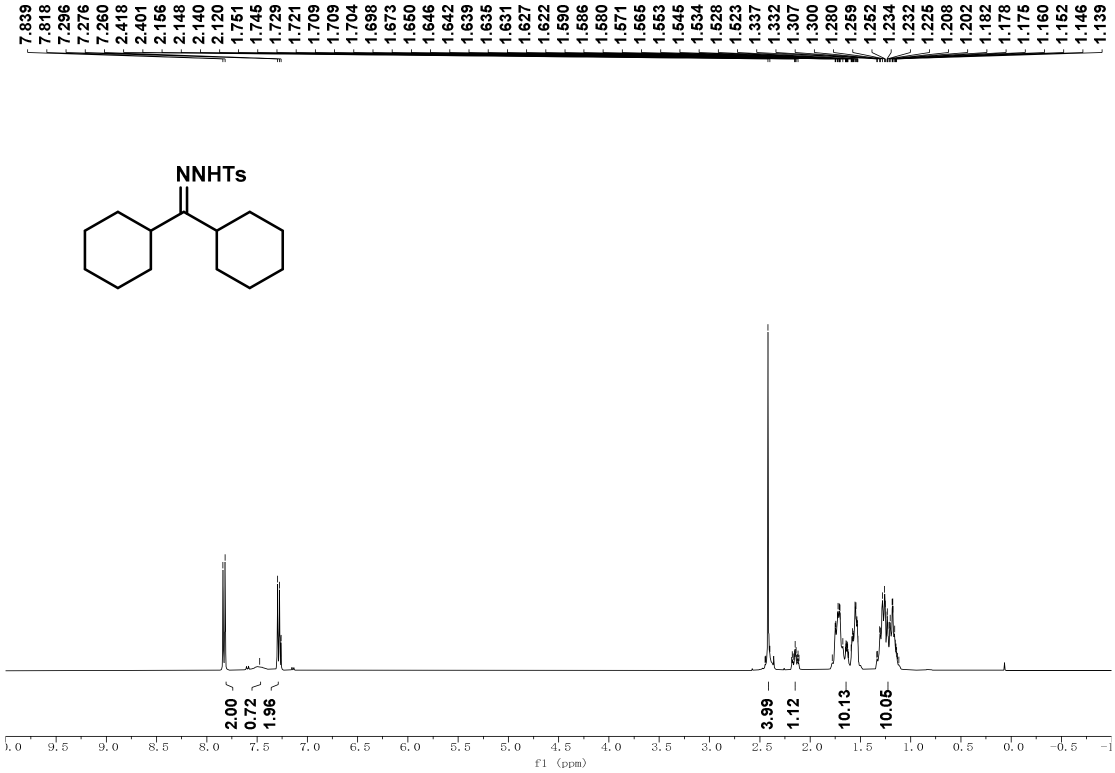
7.839 7.818 7.296 7.276 7.260 2.418 2.401 2.156 2.148 2.140 2.120 1.751 1.745 1.729 1.721 1.709 1.709 1.704 1.698 1.673 1.650 1.646 1.642 1.639 1.635 1.631 1.627 1.622 1.590 1.586 1.580 1.571 1.565 1.553 1.545 1.534 1.528 1.523 1.337 1.332 1.307 1.300 1.280 1.259 1.252 1.234 1.232 1.225 1.208 1.202 1.182 1.178 1.175 1.160 1.152 1.146 1.139
NNHTs
2.00
0.72
1.96
3.99
1.12
10.13
10.05
f1 (ppm)

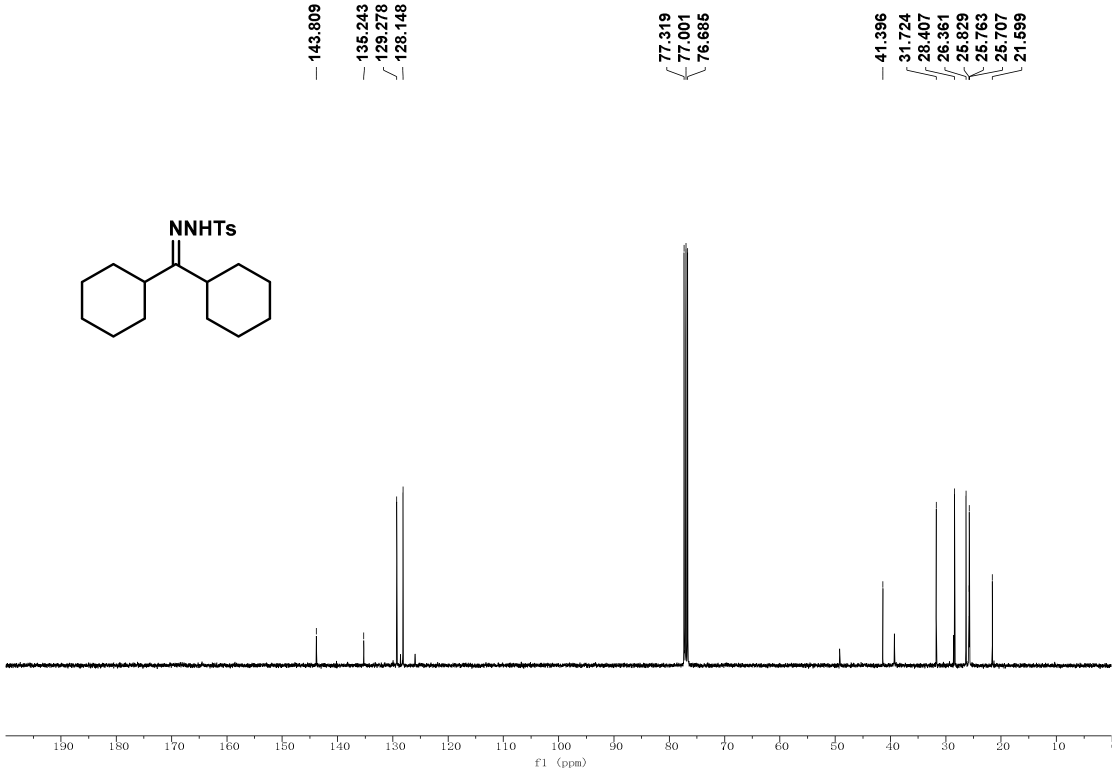
143.809
135.243
129.278
128.148
77.319
77.001
76.685
41.396
31.724
28.407
26.361
25.829
25.763
25.707
21.599
NNHTs
f1 (ppm)

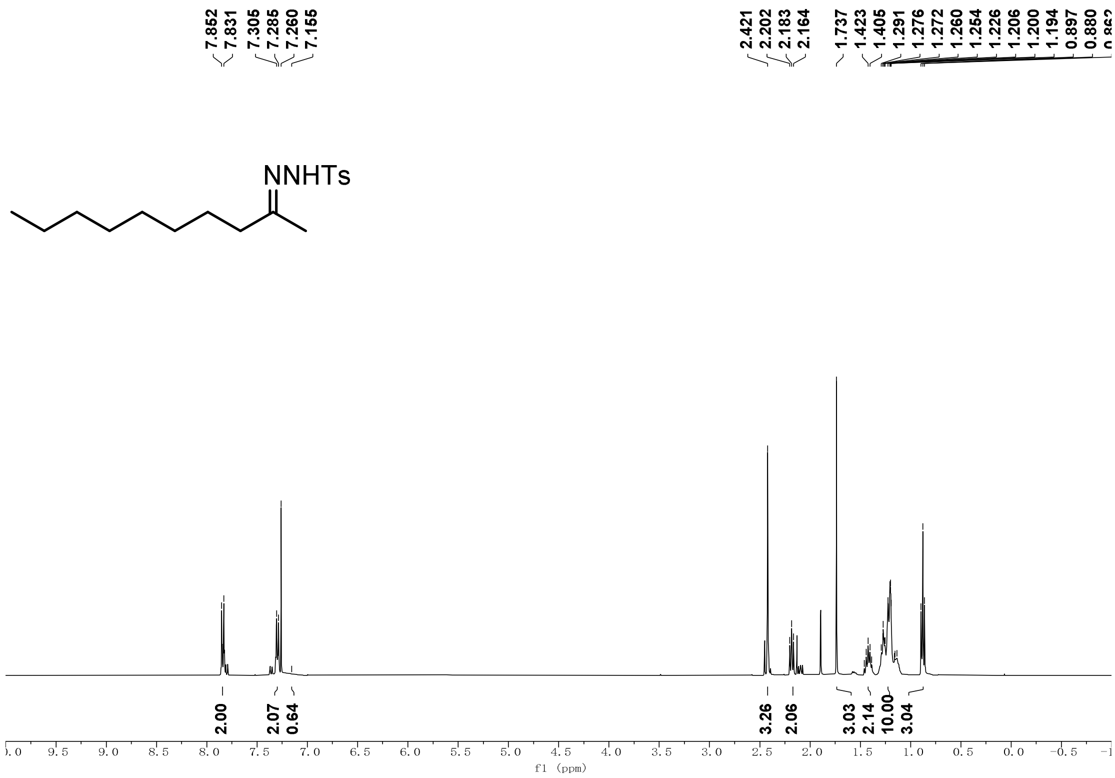

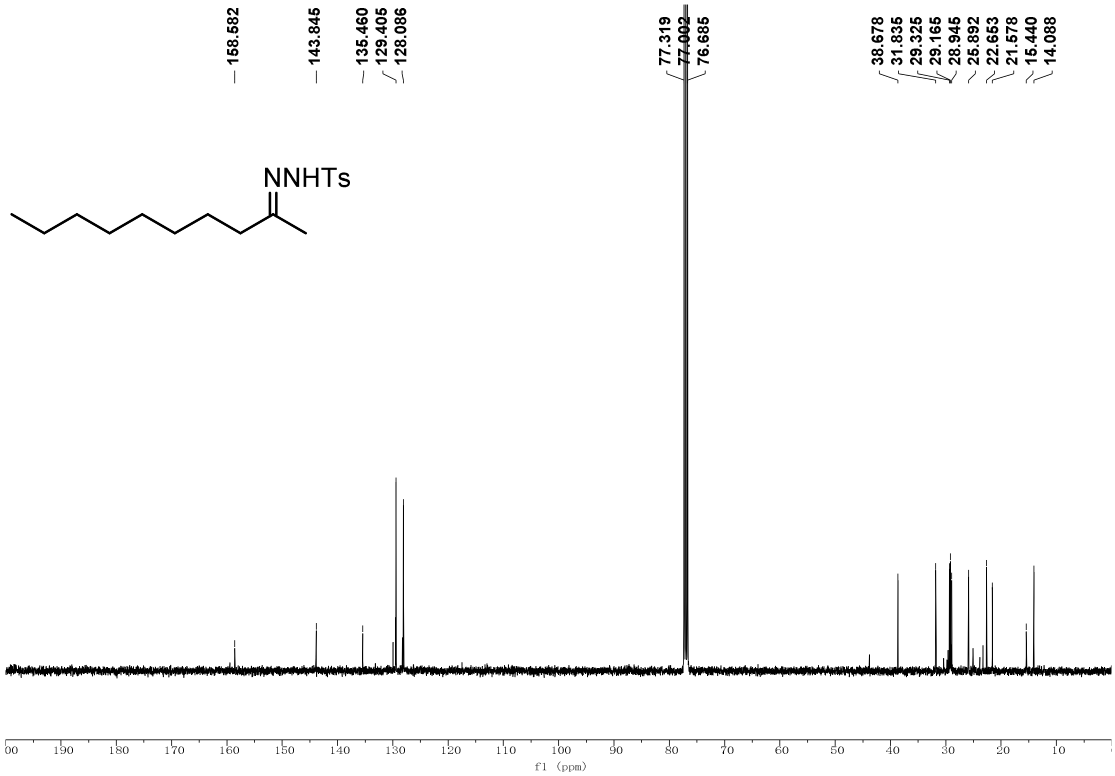

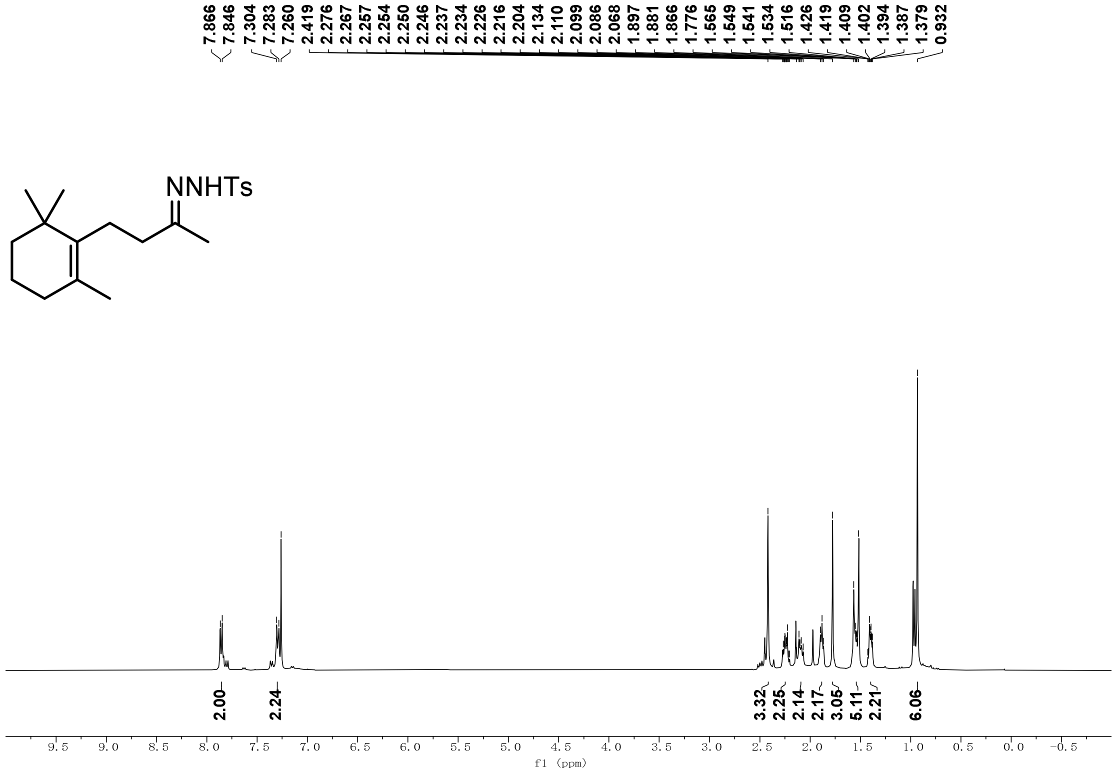
7.866
7.846
7.304
7.283
7.260
2.419
2.276
2.267
2.257
2.254
2.250
2.246
2.237
2.234
2.226
2.216
2.204
2.134
2.110
2.099
2.086
2.068
1.897
1.881
1.866
1.776
1.565
1.549
1.541
1.534
1.516
1.426
1.419
1.409
1.402
1.394
1.387
1.379
0.932
NNHTs
2.00
2.24
3.32
2.25
2.14
2.17
3.05
5.11
2.21
6.06
9.5 9.0 8.5 8.0 7.5 7.0 6.5 6.0 5.5 5.0 4.5 4.0 3.5 3.0 2.5 2.0 1.5 1.0 0.5 0.0 -0.5
f1 (ppm)

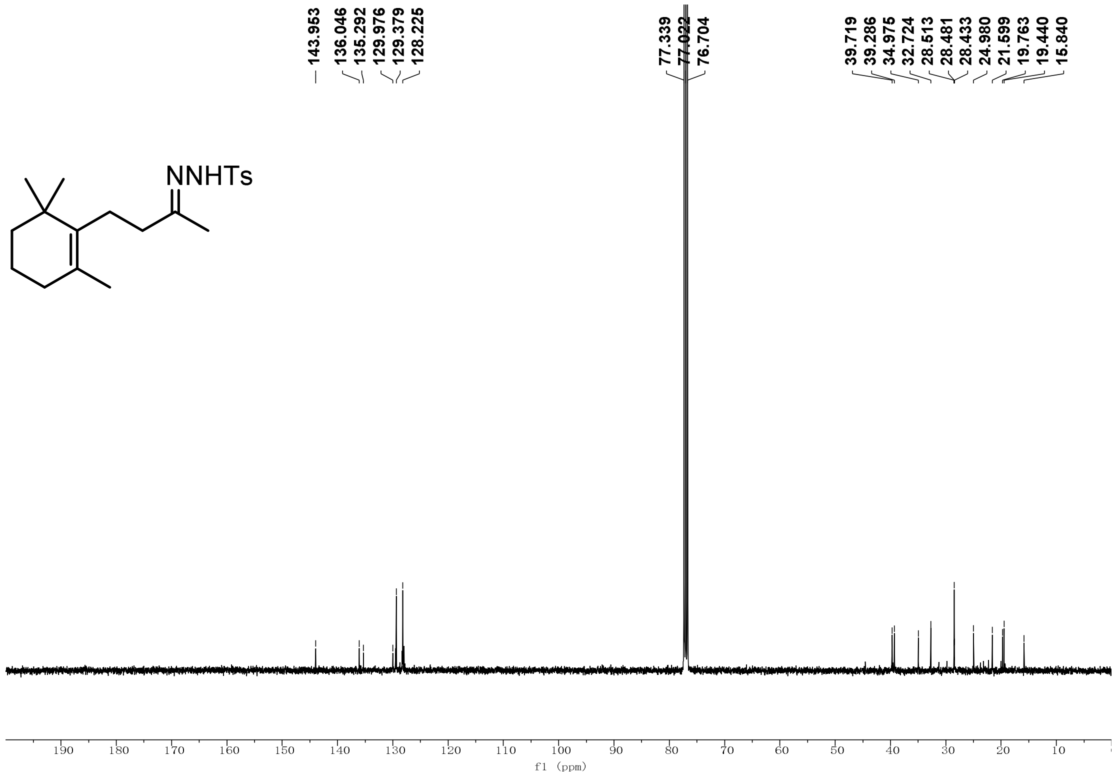
143.953
136.046
135.292
129.976
129.379
128.225
77.339
77.022
76.704
39.719
39.286
34.975
32.724
28.513
28.481
28.433
24.980
21.599
19.763
19.440
15.840
NNHTs
190 180 170 160 150 140 130 120 110 100 90 80 70 60 50 40 30 20 10
f1 (ppm)

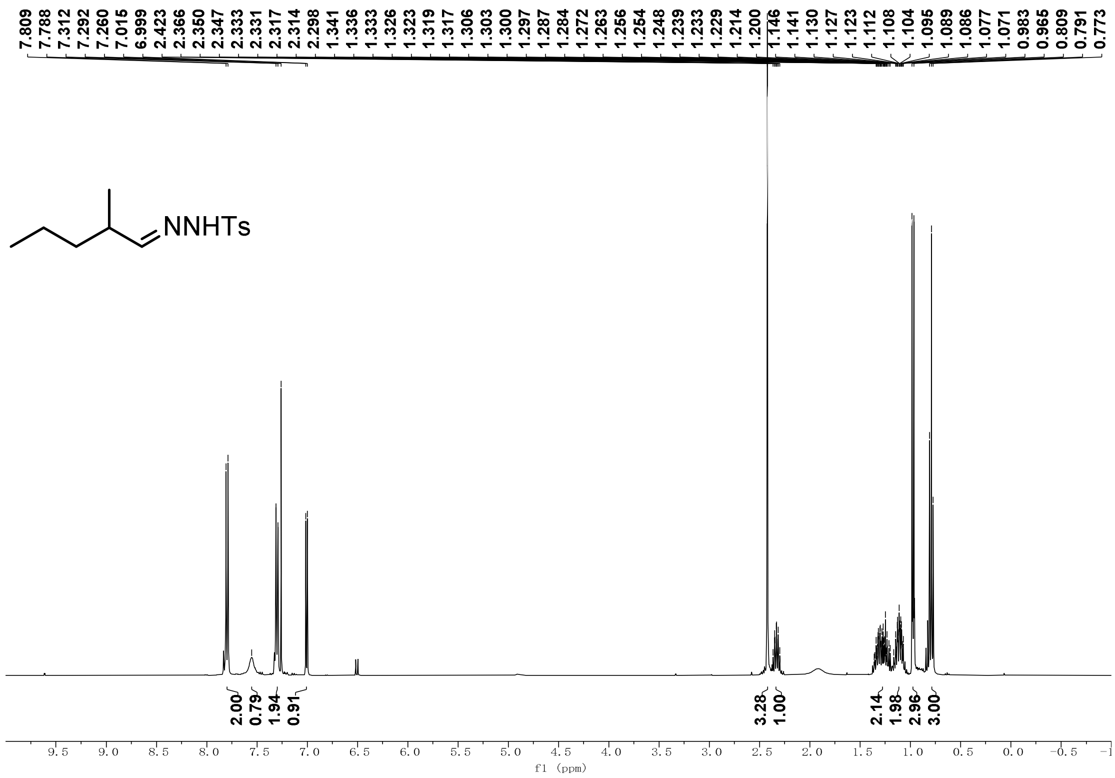

7.809 7.788 7.312 7.292 7.260 7.015 6.999 2.423 2.366 2.350 2.347 2.333 2.331 2.317 2.314 2.298 1.341 1.336 1.333 1.326 1.323 1.319 1.317 1.306 1.303 1.300 1.297 1.287 1.284 1.272 1.263 1.256 1.254 1.248 1.239 1.233 1.229 1.214 1.200 1.146 1.141 1.130 1.127 1.123 1.112 1.108 1.104 1.095 1.089 1.086 1.077 1.071 0.983 0.965 0.809 0.791 0.773
NNHTs
2.00 0.79 1.94 0.91 3.28 1.00 2.14 1.98 2.96 3.00
f1 (ppm)

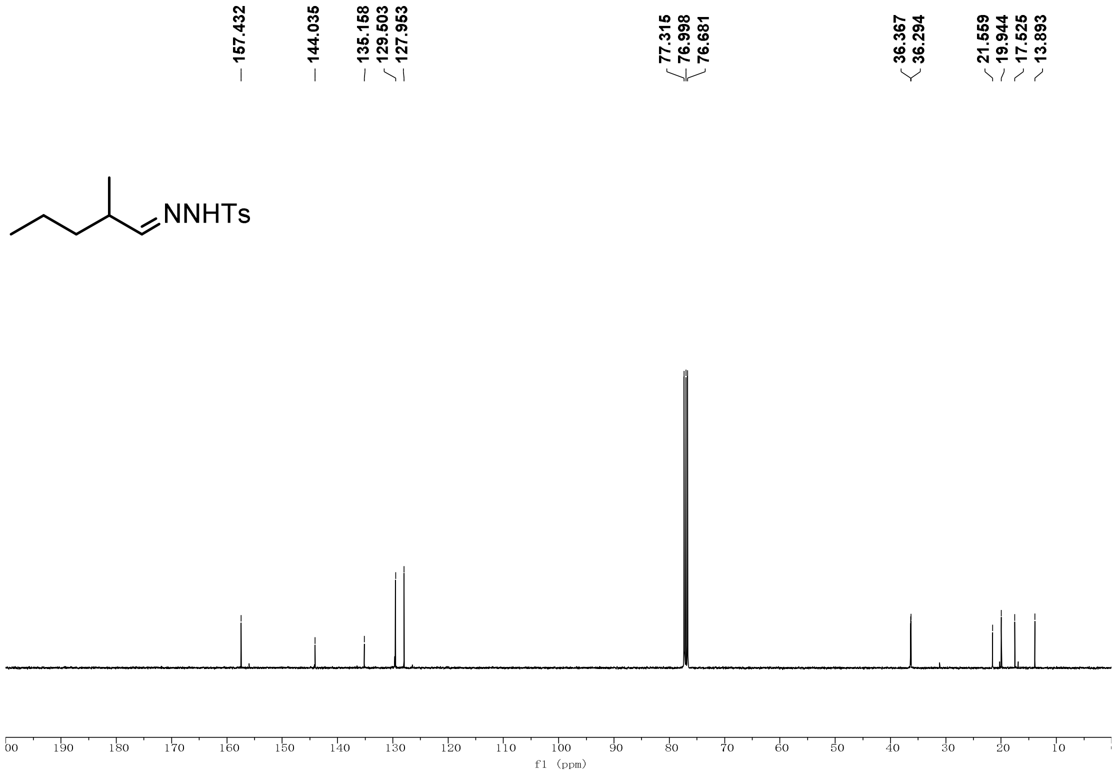

157.432 144.035 135.158 129.503 127.953 77.315 76.998 76.681 36.367 36.294 21.559 19.944 17.525 13.893
NNHTs
f1 (ppm)

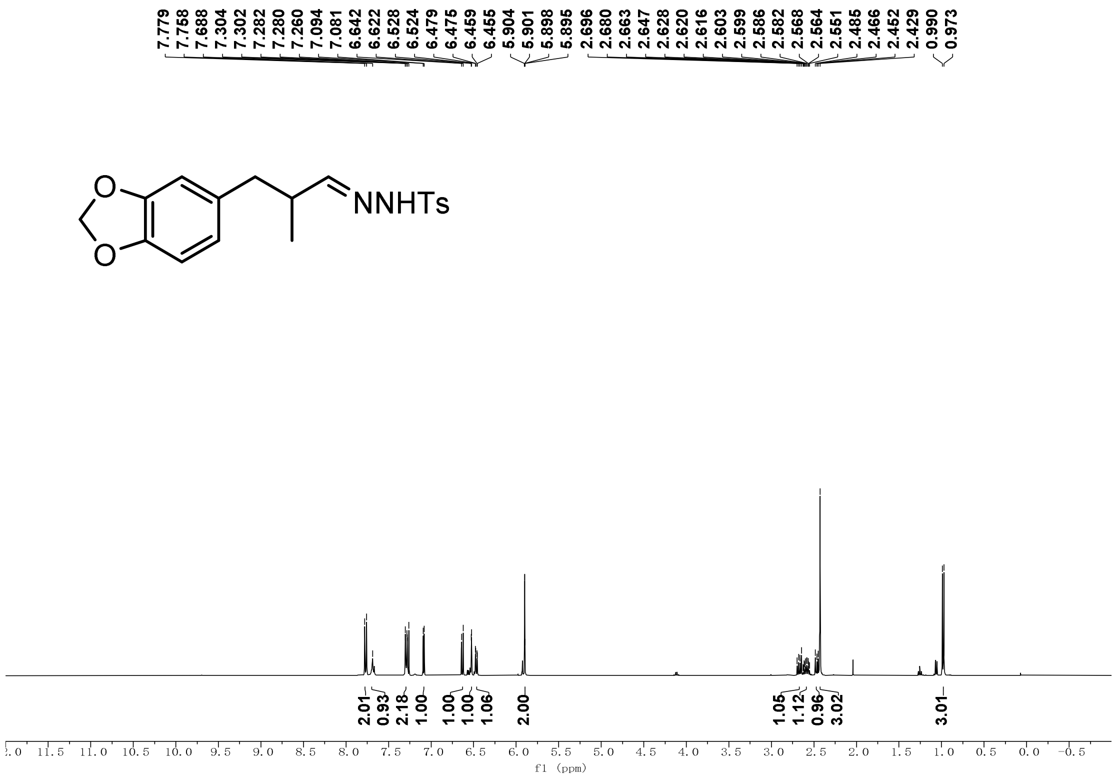
7.779
7.758
7.688
7.304
7.302
7.282
7.280
7.260
7.094
7.081
6.642
6.622
6.528
6.524
6.479
6.475
6.459
6.455
5.904
5.901
5.898
5.895
2.696
2.680
2.663
2.647
2.628
2.620
2.616
2.603
2.599
2.586
2.582
2.568
2.564
2.551
2.485
2.466
2.452
2.429
0.990
0.973
NNHTs
2.01
0.93
2.18
1.00
1.00
1.00
1.06
2.00
1.05
1.12
0.96
3.02
3.01
f1 (ppm)

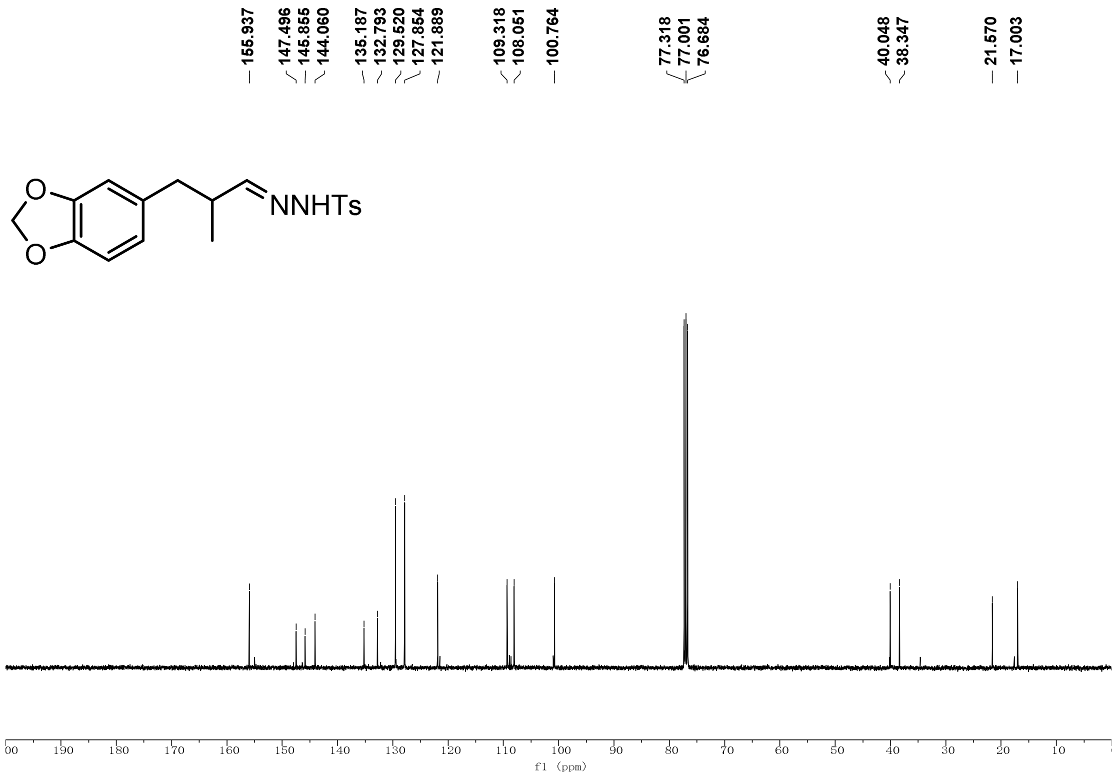
155.937
147.496
145.855
144.060
135.187
132.793
129.520
127.854
121.889
109.318
108.051
100.764
77.318
77.001
76.684
40.048
38.347
21.570
17.003
NNHTs
f1 (ppm)

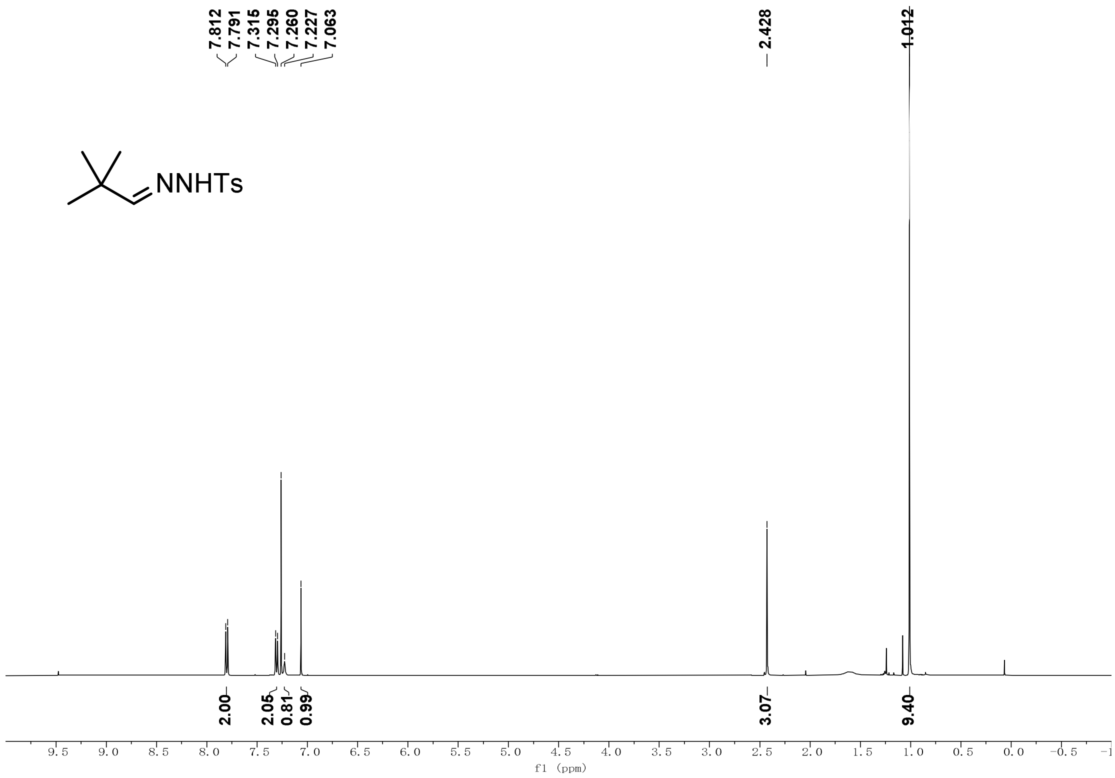
7.812
7.791
7.315
7.295
7.260
7.227
7.063
2.428
1.012
NNHTs
2.00
2.05
0.81
0.99
3.07
9.40
9.5 9.0 8.5 8.0 7.5 7.0 6.5 6.0 5.5 5.0 4.5 4.0 3.5 3.0 2.5 2.0 1.5 1.0 0.5 0.0 -0.5
f1 (ppm)

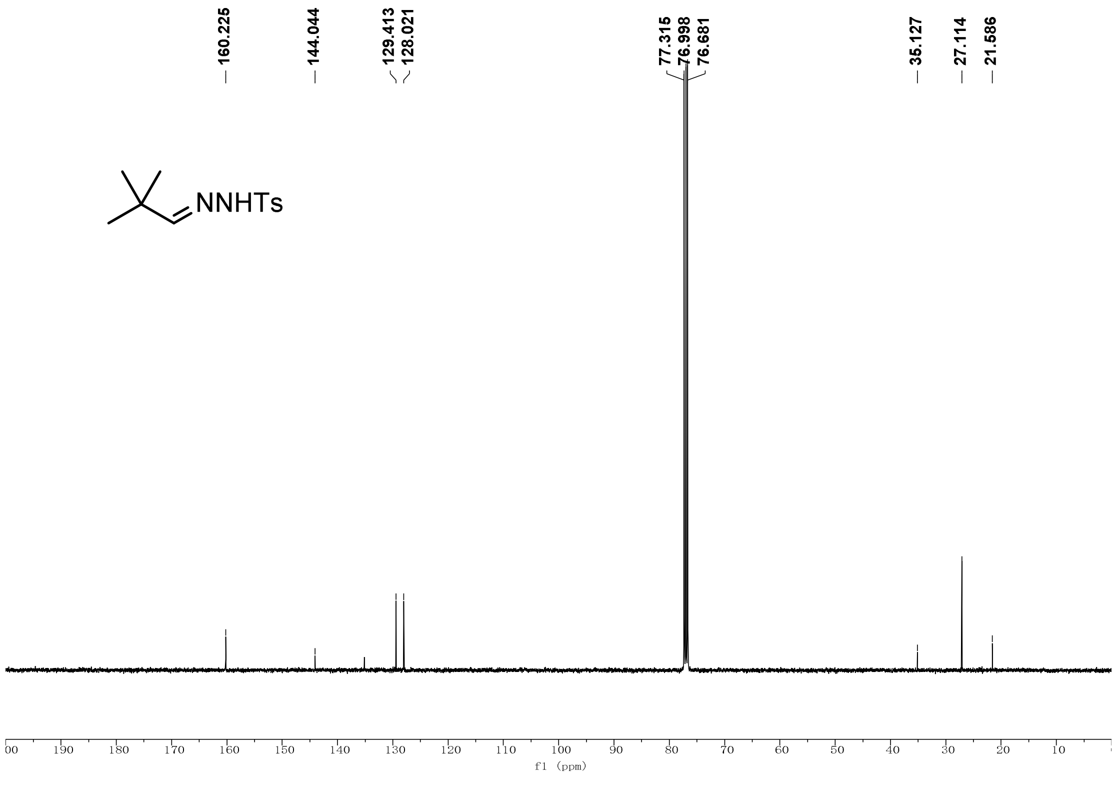
160.225
144.044
129.413
128.021
77.315
76.998
76.681
35.127
27.114
21.586
NNHTs
190 180 170 160 150 140 130 120 110 100 90 80 70 60 50 40 30 20 10
f1 (ppm)

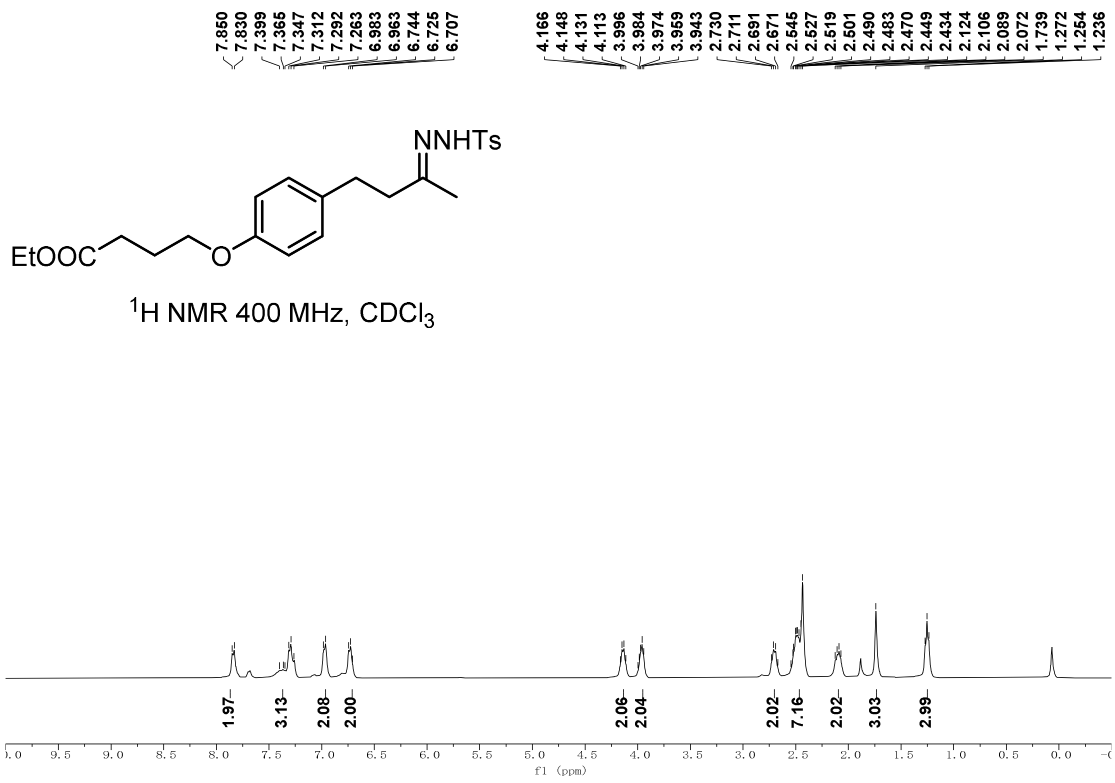

^{1}H NMR 400 MHz, $CDCl_3$

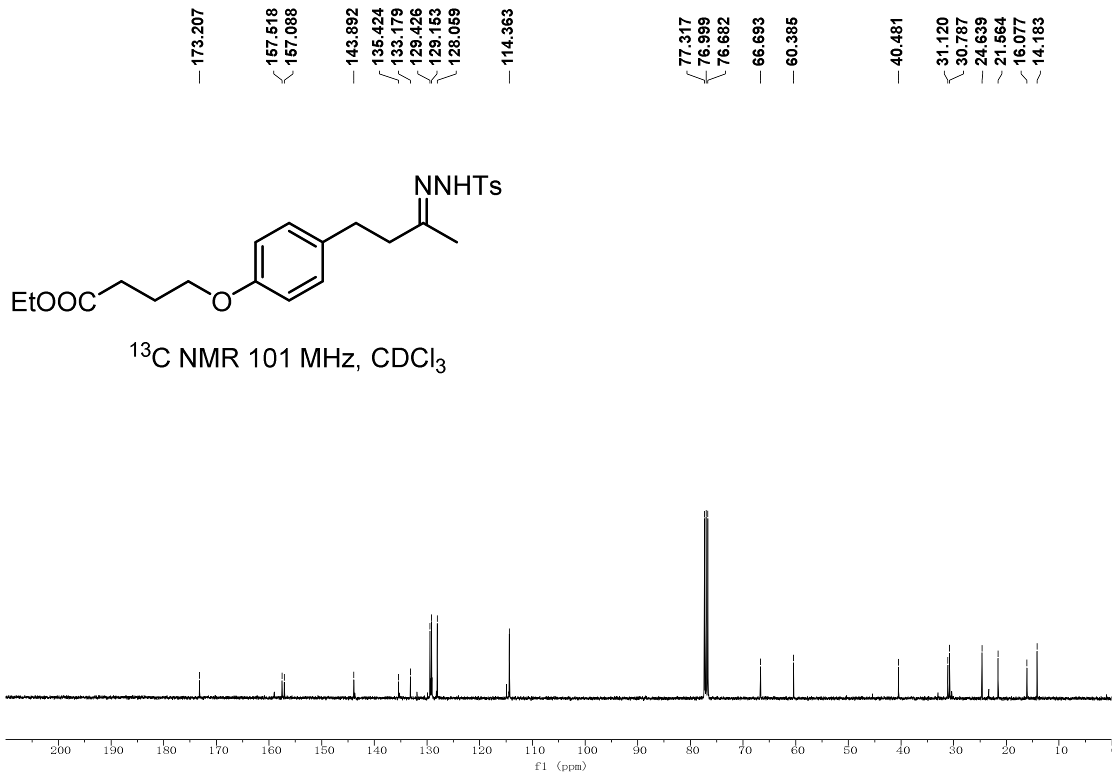

^{13}C NMR 101 MHz, $CDCl_3$

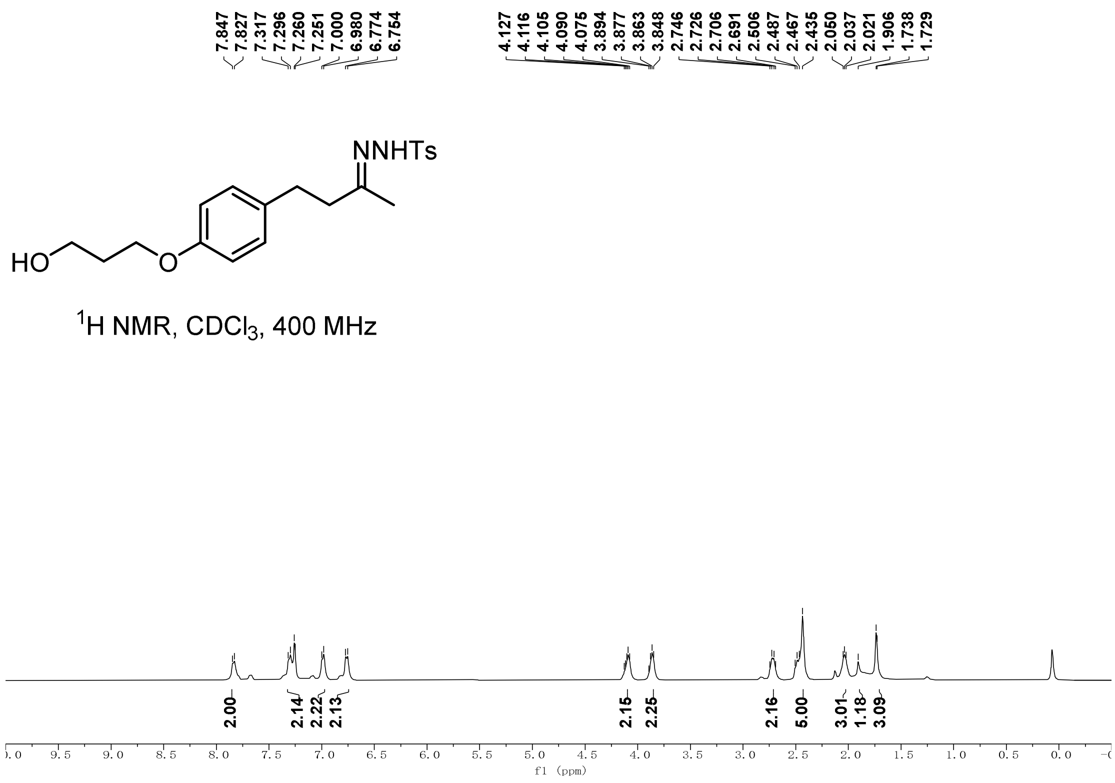
7.847
7.827
7.317
7.296
7.260
7.251
7.000
6.980
6.774
6.754
4.127
4.116
4.105
4.090
4.075
3.894
3.877
3.863
3.848
2.746
2.726
2.706
2.691
2.506
2.487
2.467
2.435
2.050
2.037
2.021
1.906
1.738
1.729
NNHTs
HO
O
^{1}H NMR, $CDCl_3$, 400 MHz
2.00
2.14
2.22
2.13
2.15
2.25
2.16
5.00
3.01
1.18
3.09
f1 (ppm)

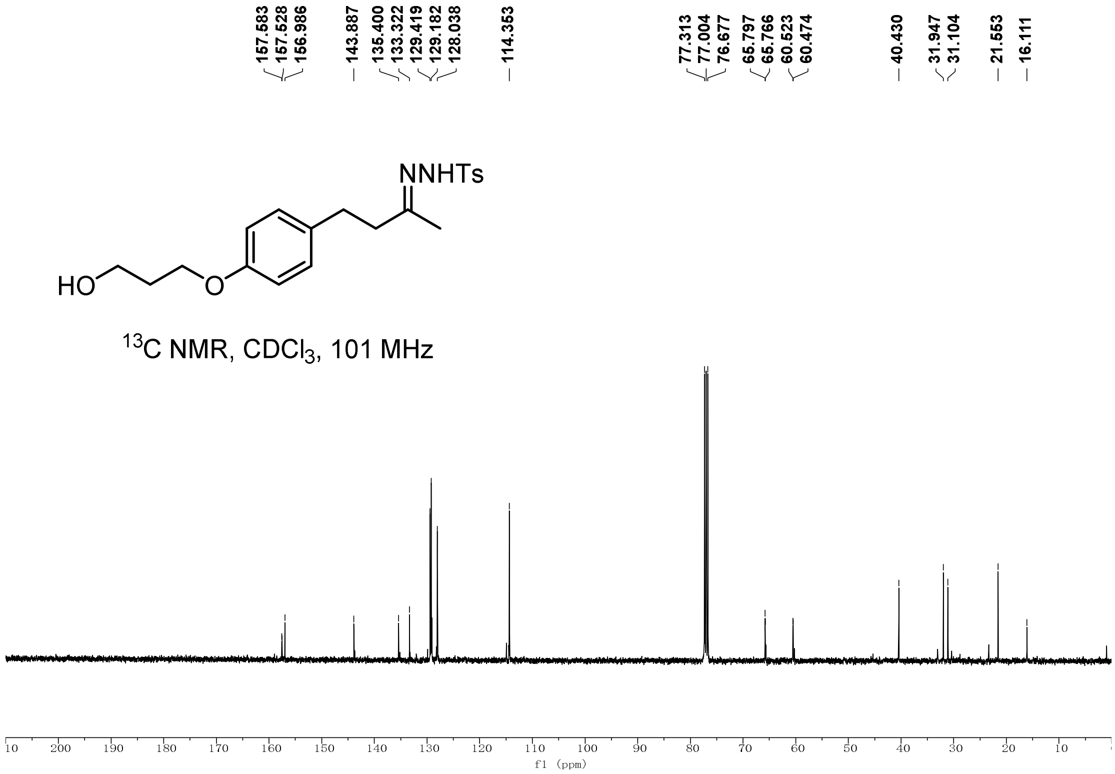
157.583
157.528
156.986
143.887
135.400
133.322
129.419
129.182
128.038
114.353
77.313
77.004
76.677
65.797
65.766
60.523
60.474
40.430
31.947
31.104
21.553
16.111
NNHTs
HO
O
^{13}C NMR, $CDCl_3$, 101 MHz
f1 (ppm)

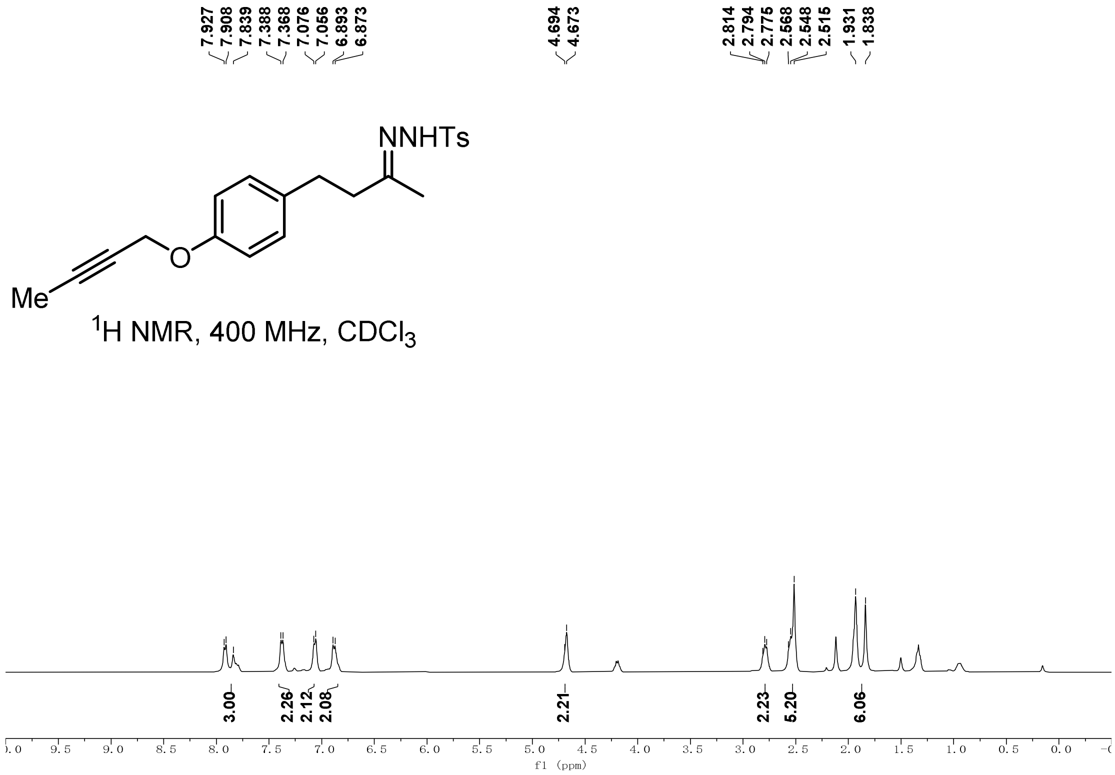
7.927 7.908 7.839 7.388 7.368 7.076 7.056 6.893 6.873
4.694 4.673
2.814 2.794 2.775 2.568 2.548 2.515 1.931 1.838
NNHTs
O
Me
^{1}H NMR, 400 MHz, CDCl$_3$
3.00 2.26 2.12 2.08 2.21 2.23 5.20 6.06
f1 (ppm)

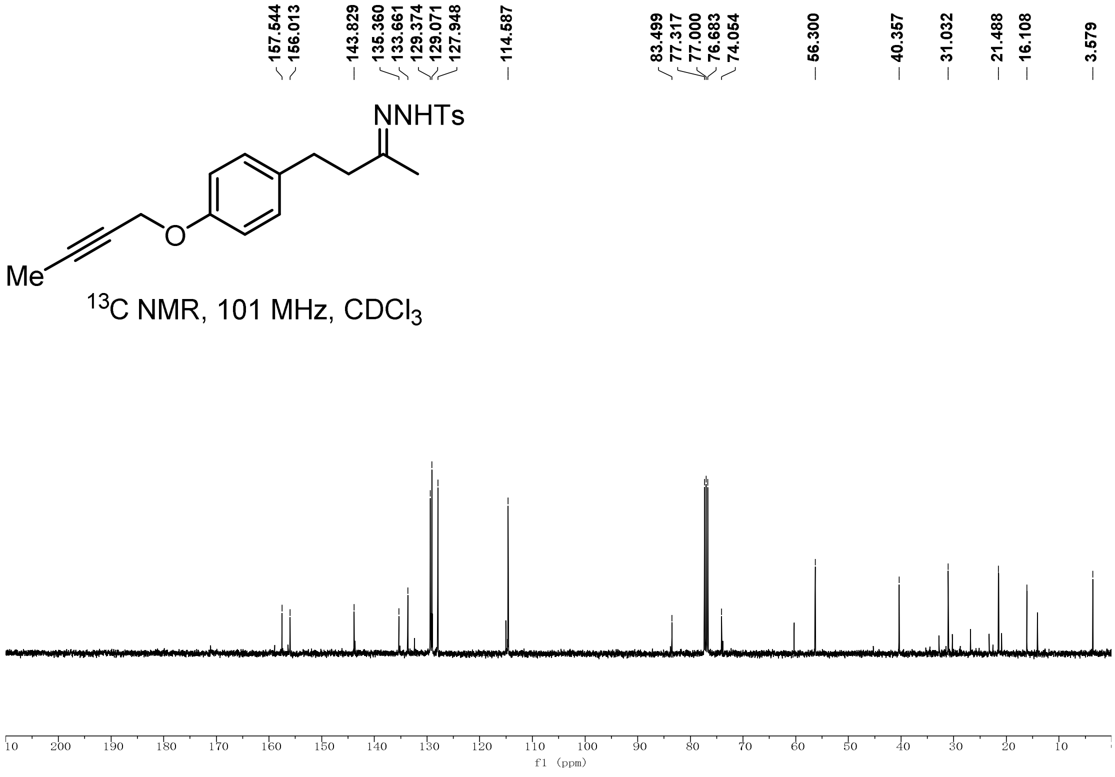
157.544 156.013 143.829 135.360 133.661 129.374 129.071 127.948 114.587
83.499 77.317 77.000 76.683 74.054 56.300 40.357 31.032 21.488 16.108 3.579
NNHTs
O
Me
^{13}C NMR, 101 MHz, CDCl$_3$
f1 (ppm)

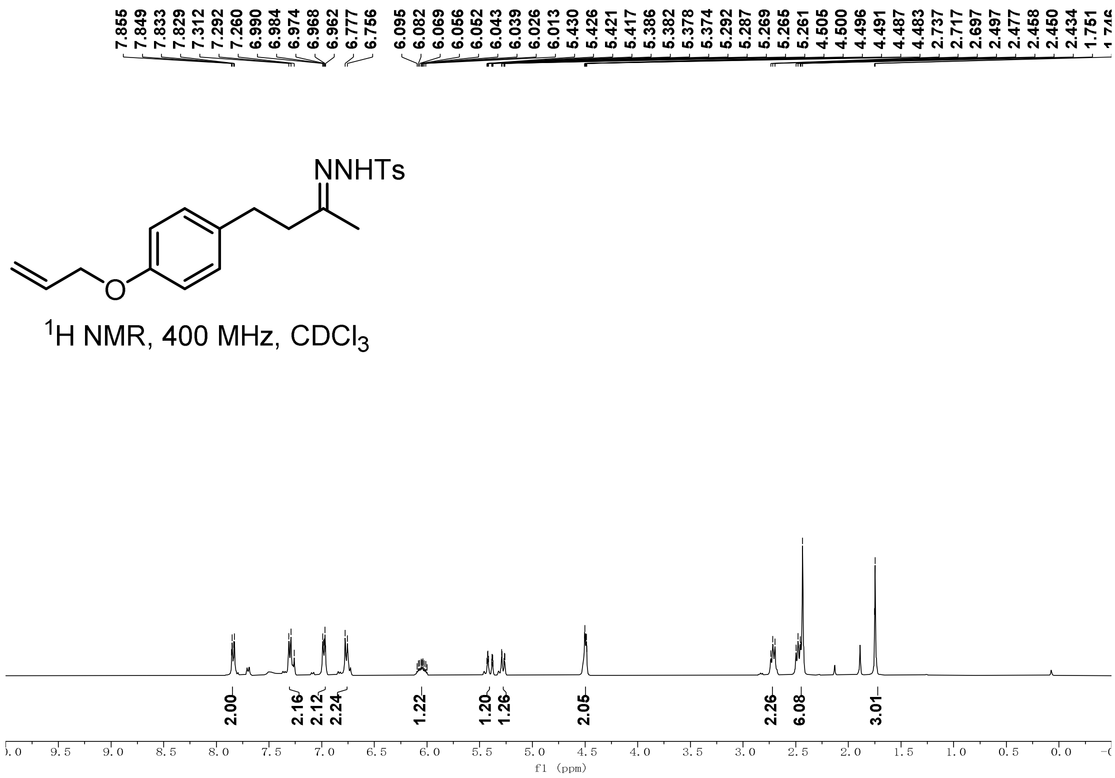
NNHTs
O
1H NMR, 400 MHz, CDCl3
7.855
7.849
7.833
7.829
7.312
7.292
7.260
6.990
6.984
6.974
6.968
6.962
6.777
6.756
6.095
6.082
6.069
6.056
6.052
6.043
6.039
6.026
6.013
5.430
5.426
5.421
5.417
5.386
5.382
5.378
5.374
5.292
5.287
5.269
5.265
5.261
4.505
4.500
4.496
4.491
4.487
4.483
2.737
2.717
2.697
2.497
2.477
2.458
2.450
2.434
1.751
2.00
2.16
2.12
2.24
1.22
1.20
1.26
2.05
2.26
6.08
3.01
f1 (ppm)

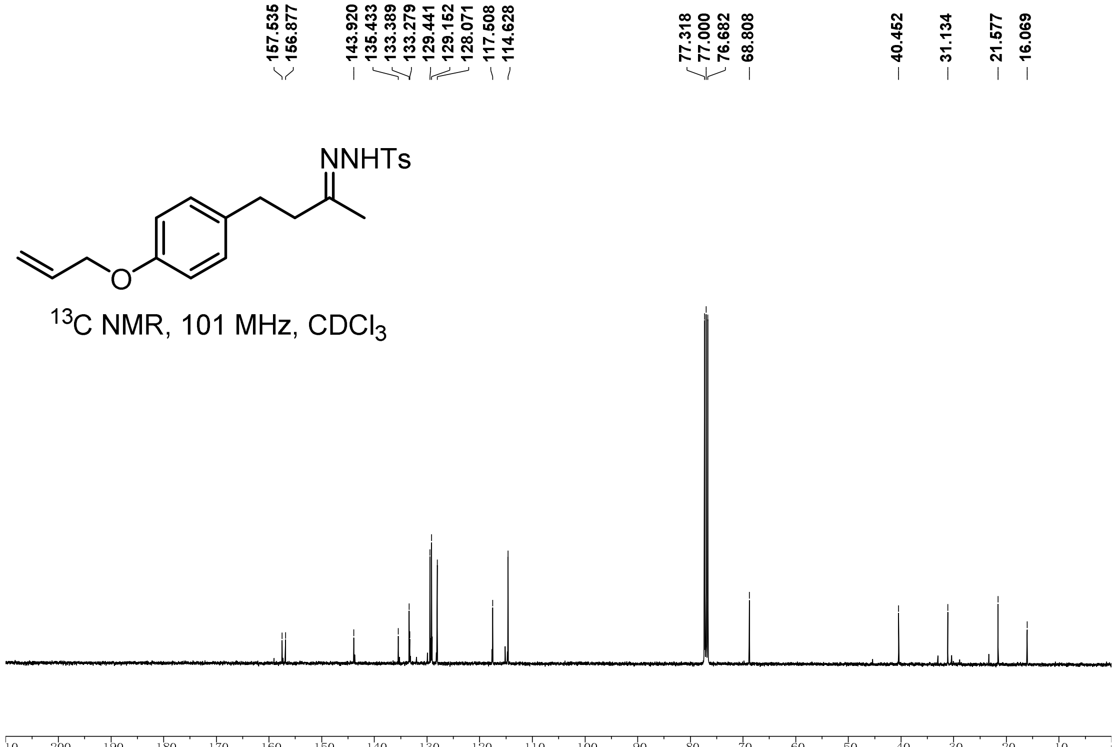
157.535
156.877
143.920
135.433
133.389
133.279
129.441
129.152
128.071
117.508
114.628
77.318
77.000
76.682
68.808
40.452
31.134
21.577
16.069
NNHTs
O
13C NMR, 101 MHz, CDCl3
f1 (ppm)

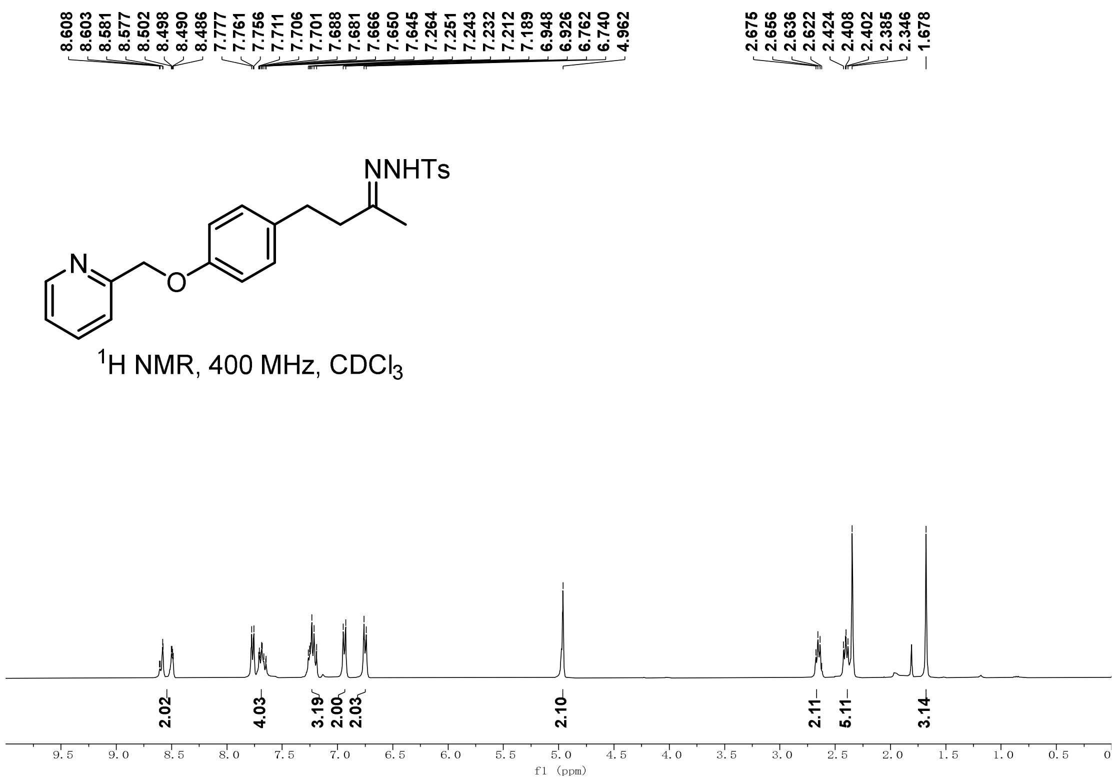
8.608
8.603
8.581
8.577
8.502
8.498
8.490
8.486
7.777
7.761
7.756
7.711
7.706
7.701
7.688
7.681
7.666
7.650
7.645
7.264
7.251
7.243
7.232
7.212
7.189
6.948
6.926
6.762
6.740
4.962
2.675
2.656
2.636
2.622
2.424
2.408
2.402
2.385
2.346
1.678
NNHTs
N
O
^{1}H NMR, 400 MHz, $CDCl_3$
2.02
4.03
3.19
2.00
2.03
2.10
2.11
5.11
3.14
f1 (ppm)

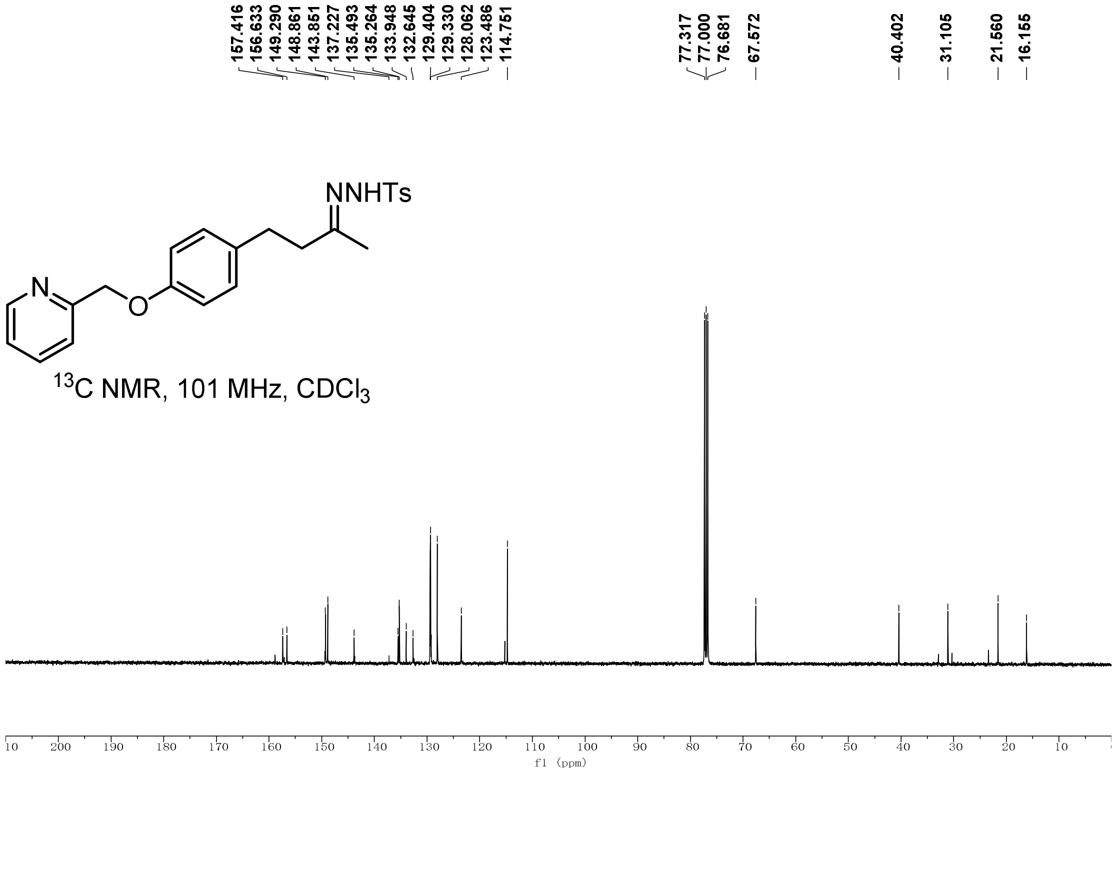
157.416
156.633
149.290
148.861
143.851
137.227
135.493
135.264
133.948
132.645
129.404
129.330
128.062
123.486
114.751
77.317
77.000
76.681
67.572
40.402
31.105
21.560
16.155
NNHTs
N
O
^{13}C NMR, 101 MHz, $CDCl_3$
f1 (ppm)

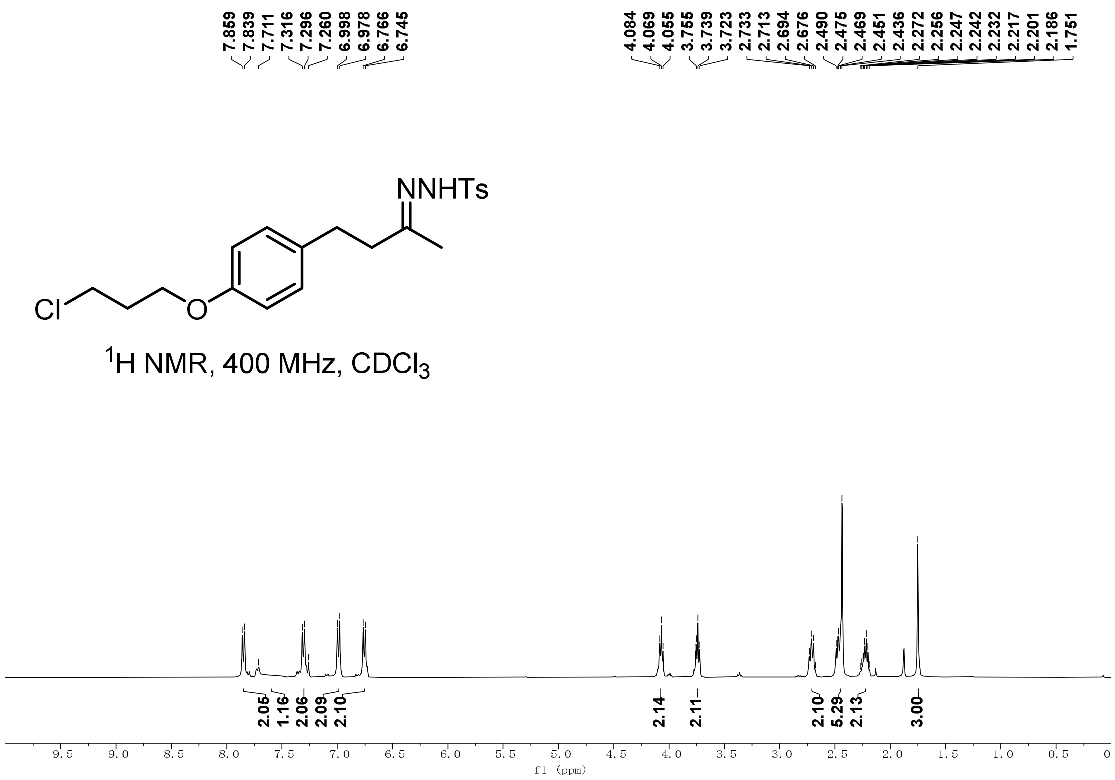

7.859
7.839
7.711
7.316
7.296
7.260
6.998
6.978
6.766
6.745
4.084
4.069
4.055
3.755
3.739
3.723
2.733
2.713
2.694
2.676
2.490
2.475
2.469
2.451
2.436
2.272
2.256
2.247
2.242
2.232
2.217
2.201
2.186
1.751
NNHTs
Cl
O
¹H NMR, 400 MHz, CDCl₃
2.05
1.16
2.06
2.09
2.10
2.14
2.11
2.10
5.29
2.13
3.00
f1 (ppm)

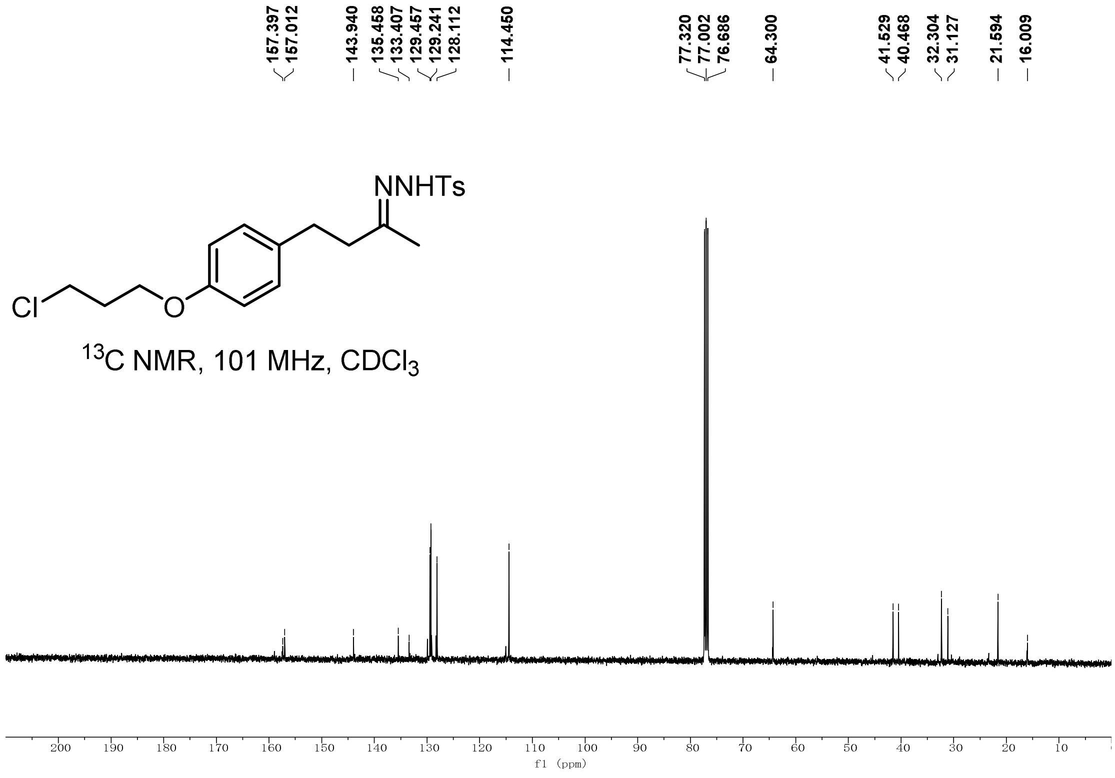

157.397
157.012
143.940
135.458
133.407
129.457
129.241
128.112
114.450
77.320
77.002
76.686
64.300
41.529
40.468
32.304
31.127
21.594
16.009
NNHTs
Cl
O
¹³C NMR, 101 MHz, CDCl₃
f1 (ppm)

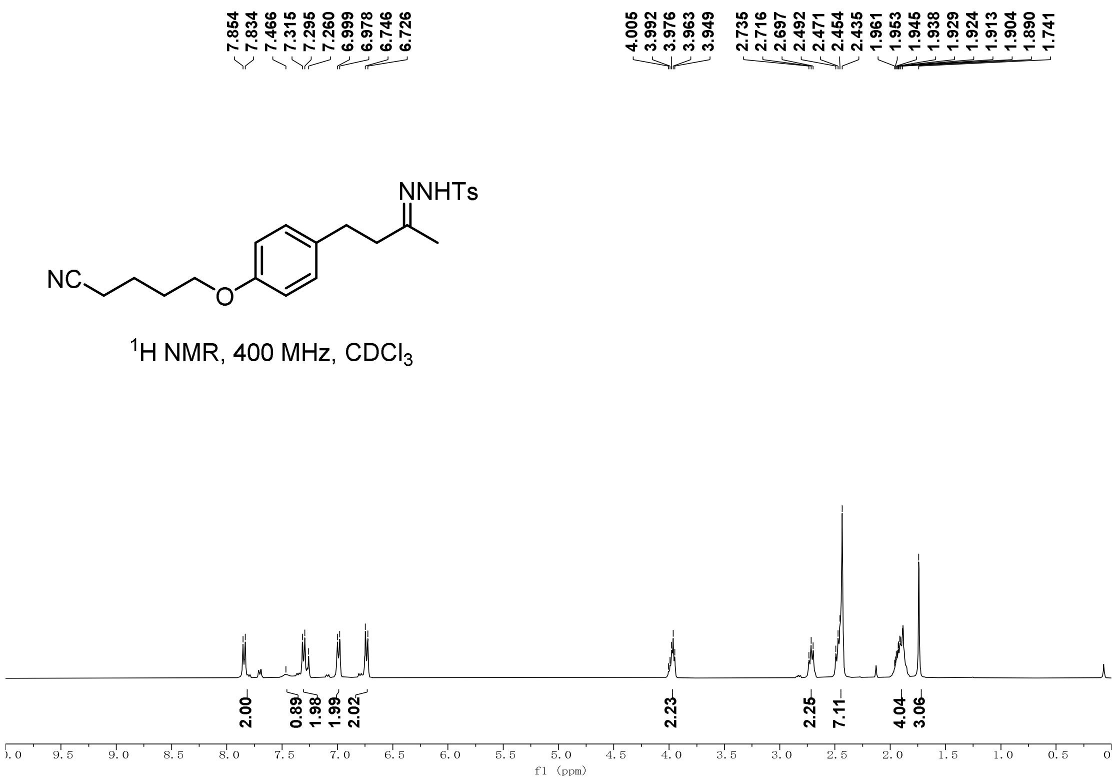
7.854 7.834 7.466 7.316 7.295 7.260 6.999 6.978 6.746 6.726
4.005 3.992 3.976 3.963 3.949
2.735 2.716 2.697 2.492 2.471 2.454 2.435
1.961 1.953 1.945 1.938 1.929 1.924 1.913 1.904 1.890 1.741
NNHTs
NC
O
^{1}H NMR, 400 MHz, $CDCl_3$
2.00 0.89 1.98 1.99 2.02 2.23 2.25 7.11 4.04 3.06
f1 (ppm)

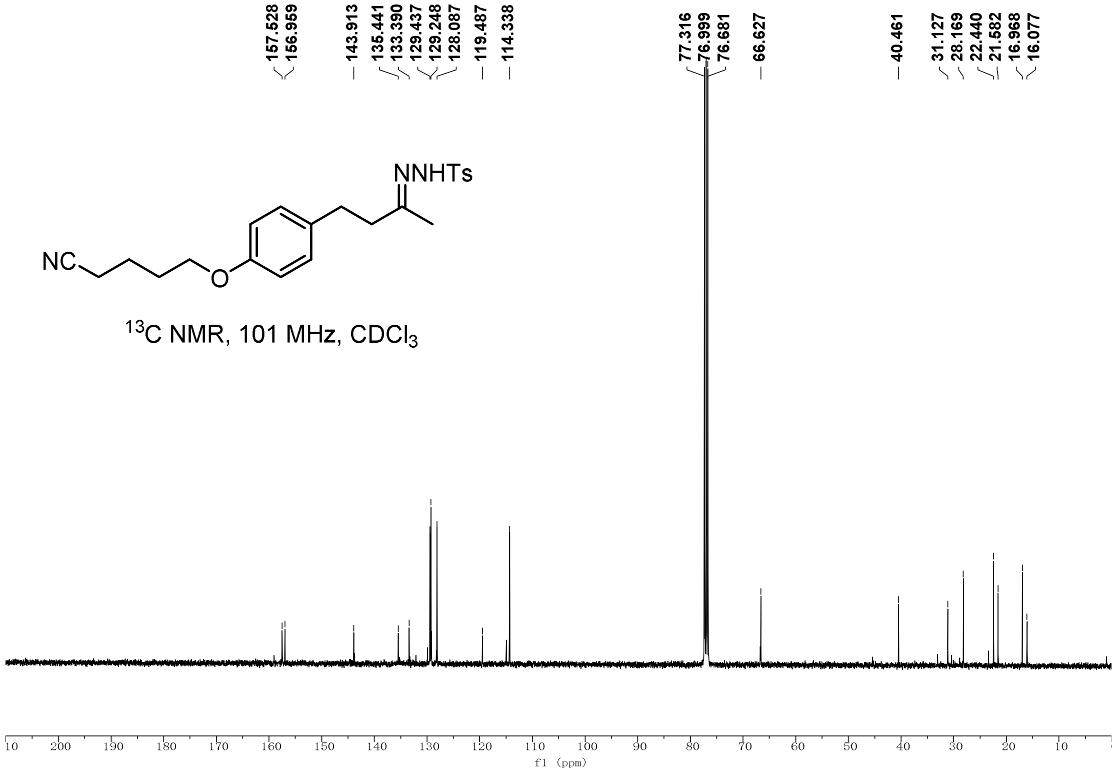
157.528 156.959 143.913 135.441 133.390 129.437 129.248 128.087 119.487 114.338
77.316 76.999 76.681 66.627
40.461 31.127 28.169 22.440 21.582 16.968 16.077
NNHTs
NC
O
^{13}C NMR, 101 MHz, $CDCl_3$
f1 (ppm)

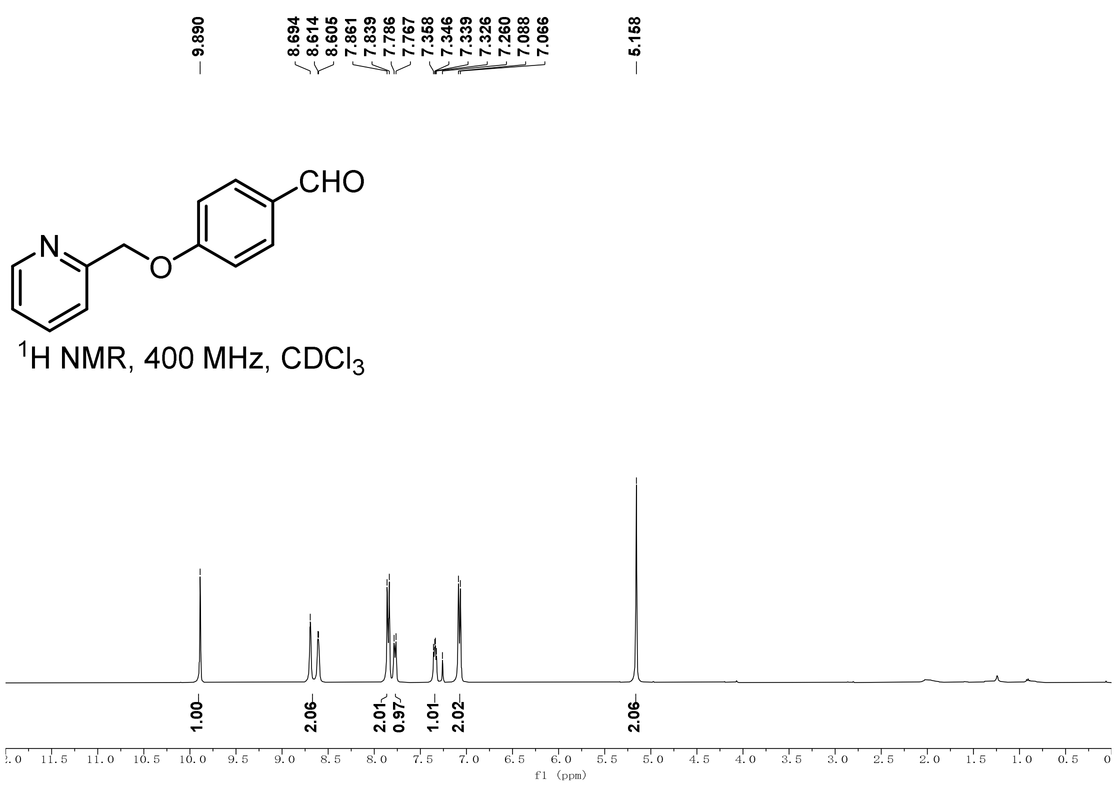
9.890
8.694
8.614
8.605
7.861
7.839
7.786
7.767
7.358
7.346
7.339
7.326
7.260
7.088
7.066
5.158
CHO
N
O
^{1}H NMR, 400 MHz, $CDCl_3$
1.00
2.06
2.01
0.97
1.01
2.02
2.06
f1 (ppm)

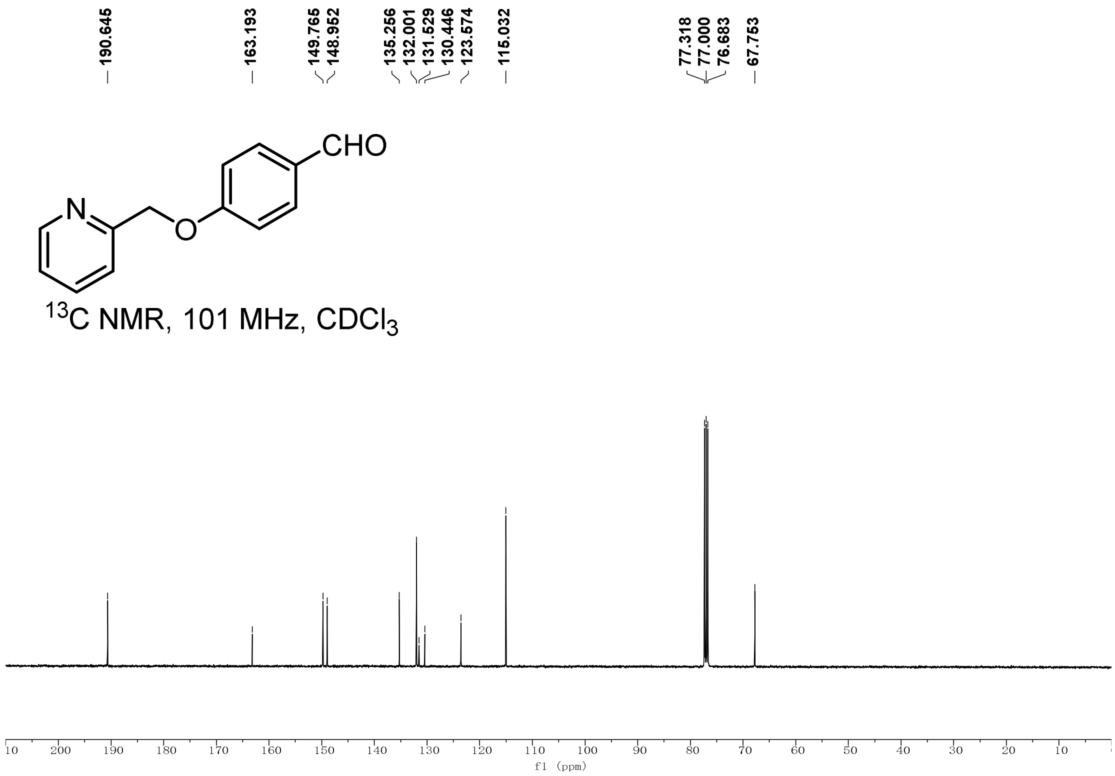
190.645
163.193
149.765
148.952
135.256
132.001
131.529
130.446
123.574
115.032
77.318
77.000
76.683
67.753
CHO
N
O
^{13}C NMR, 101 MHz, $CDCl_3$
f1 (ppm)

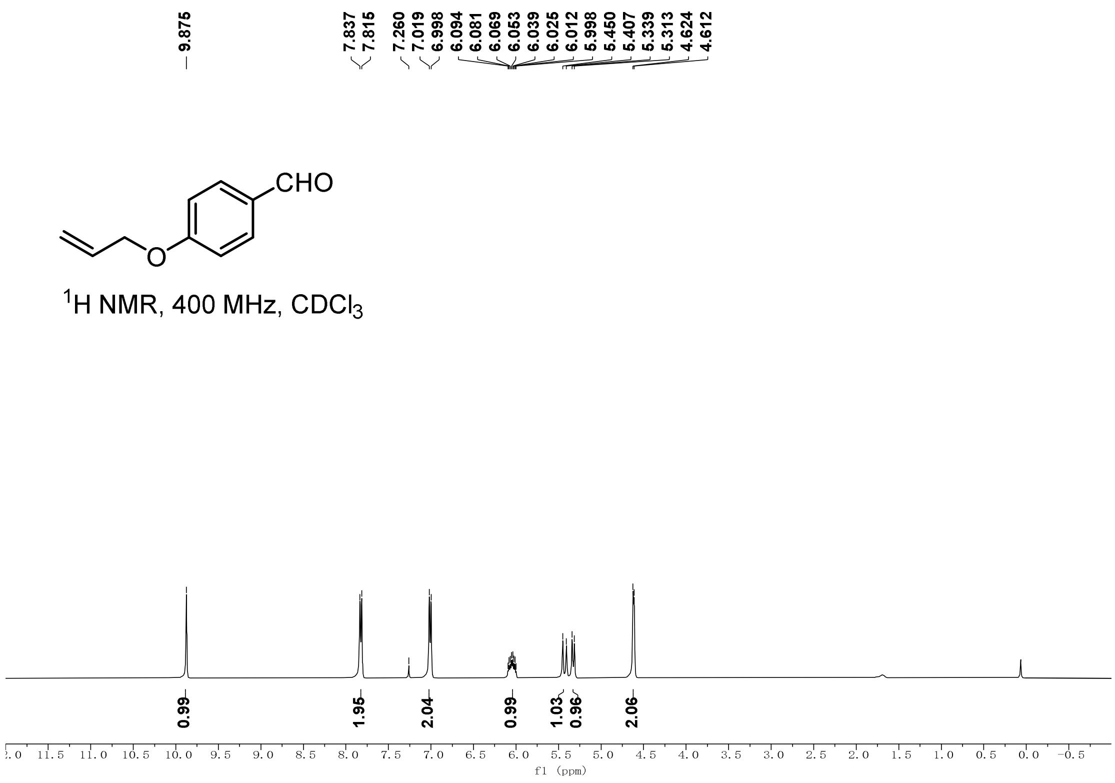
9.875
7.837
7.815
7.260
7.019
6.998
6.094
6.081
6.069
6.053
6.039
6.025
6.012
5.998
5.450
5.407
5.339
5.313
4.624
4.612
CHO
O
^{1}H NMR, 400 MHz, $CDCl_3$
0.99
1.95
2.04
0.99
1.03
0.96
2.06
f1 (ppm)

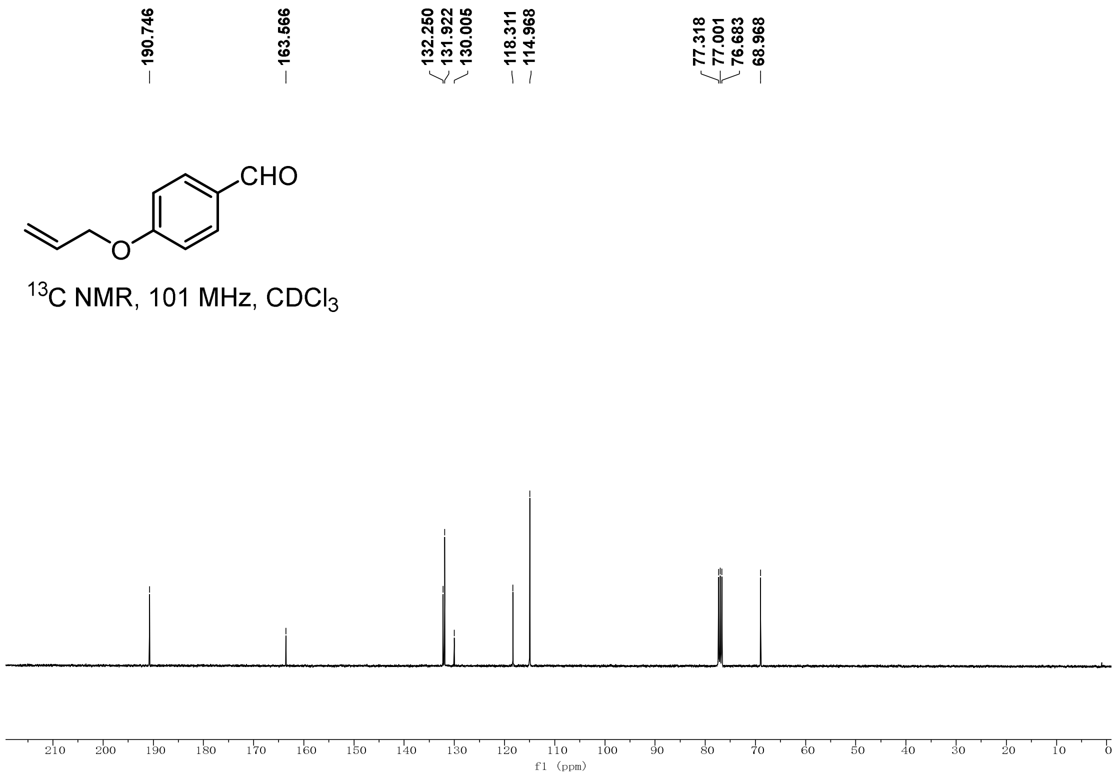
190.746
163.566
132.250
131.922
130.005
118.311
114.968
77.318
77.001
76.683
68.968
CHO
O
^{13}C NMR, 101 MHz, $CDCl_3$
f1 (ppm)

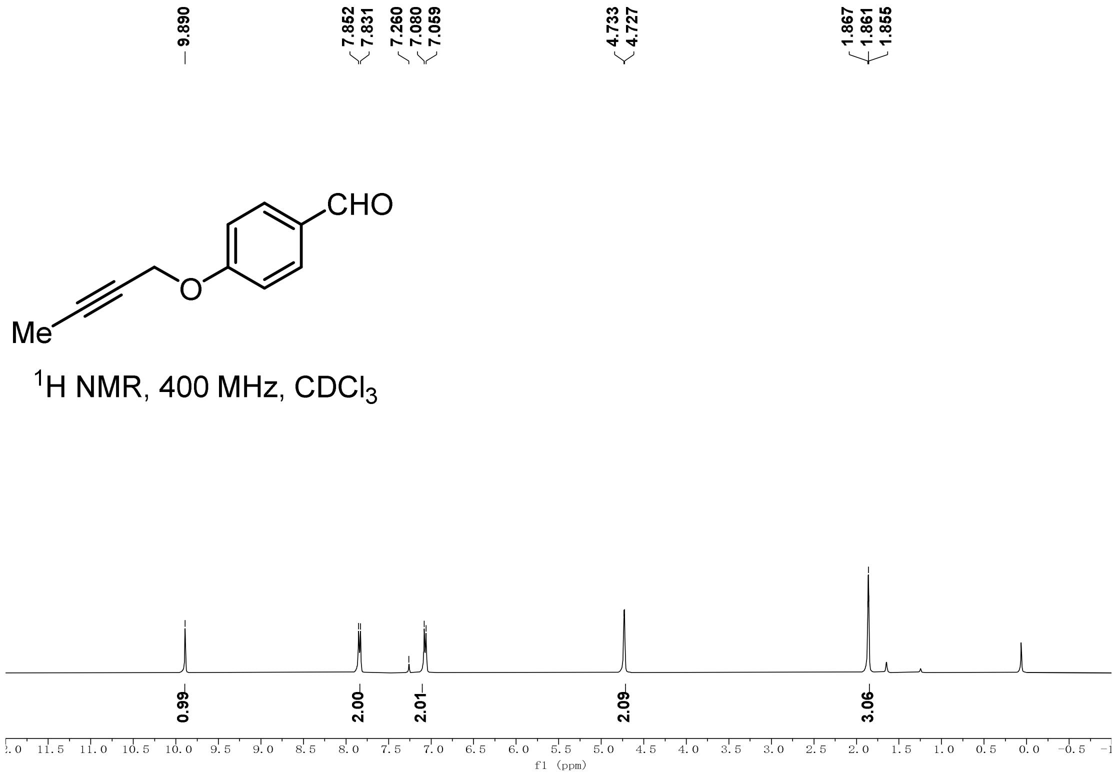
9.890
7.852
7.831
7.260
7.080
7.059
4.733
4.727
1.867
1.861
1.855
CHO
O
Me
1H NMR, 400 MHz, CDCl3
0.99
2.00
2.01
2.09
3.06
f1 (ppm)

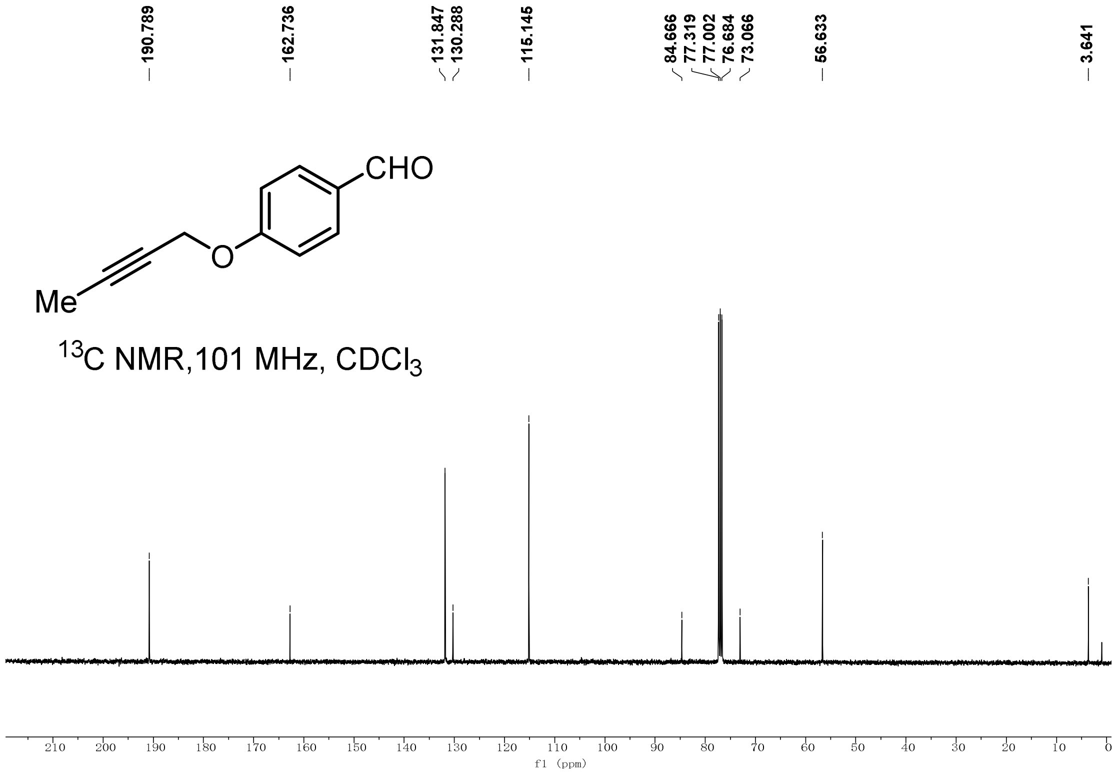
190.789
162.736
131.847
130.288
115.145
84.666
77.319
77.002
76.684
73.066
56.633
3.641
CHO
O
Me
13C NMR,101 MHz, CDCl3
f1 (ppm)

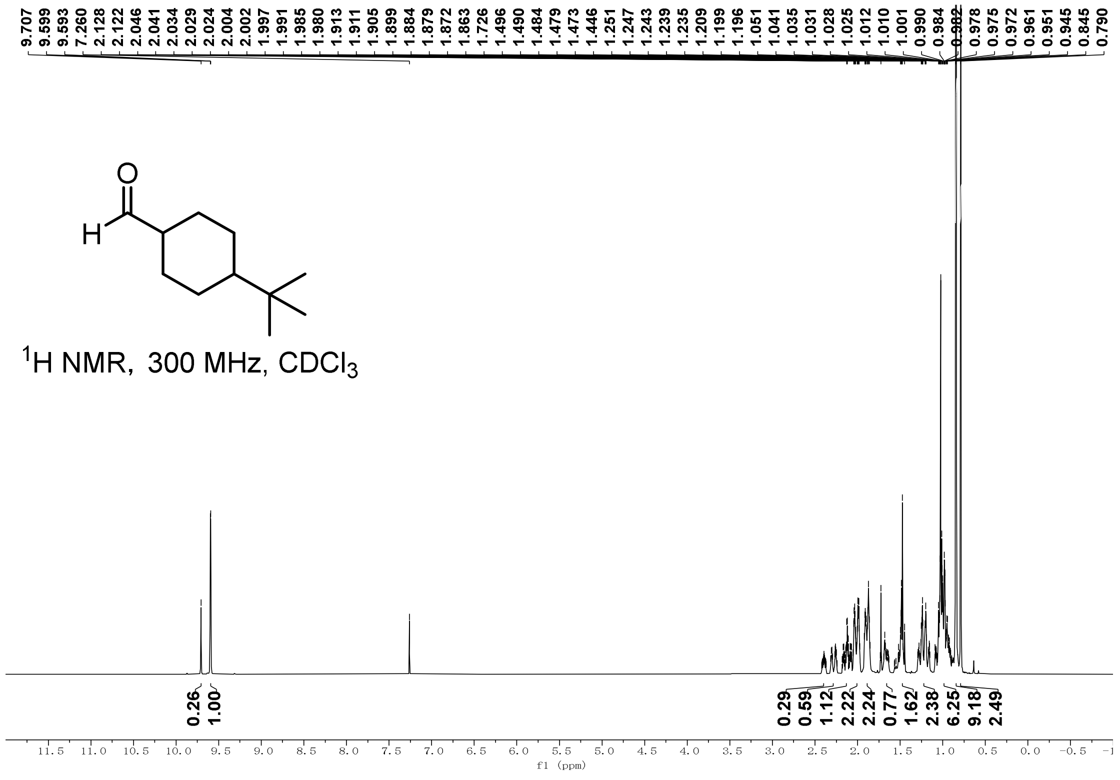
9.707
9.599
9.593
7.260
2.128
2.122
2.046
2.041
2.034
2.029
2.024
2.004
2.002
1.997
1.991
1.985
1.980
1.913
1.911
1.905
1.899
1.884
1.879
1.872
1.863
1.726
1.496
1.490
1.484
1.479
1.473
1.446
1.251
1.247
1.243
1.239
1.235
1.209
1.199
1.196
1.051
1.041
1.035
1.031
1.028
1.025
1.012
1.010
1.001
0.990
0.984
0.978
0.975
0.972
0.961
0.951
0.945
0.845
0.790
H
O
^{1}H NMR, 300 MHz, $CDCl_3$
0.26
1.00
0.29
0.59
1.12
2.22
2.24
0.77
1.62
2.38
6.25
9.18
2.49
f1 (ppm)

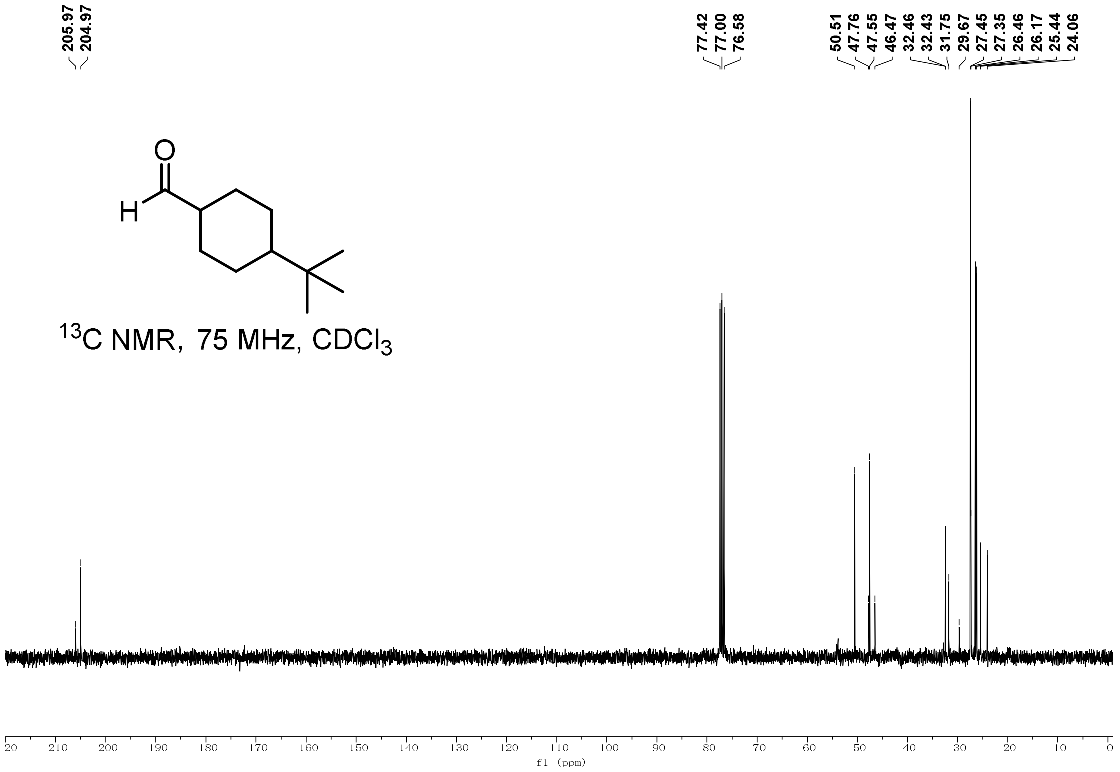
205.97
204.97
77.42
77.00
76.58
50.51
47.76
47.55
46.47
32.46
32.43
31.75
29.67
27.45
27.35
26.46
26.17
25.44
24.06
H
O
^{13}C NMR, 75 MHz, $CDCl_3$
f1 (ppm)

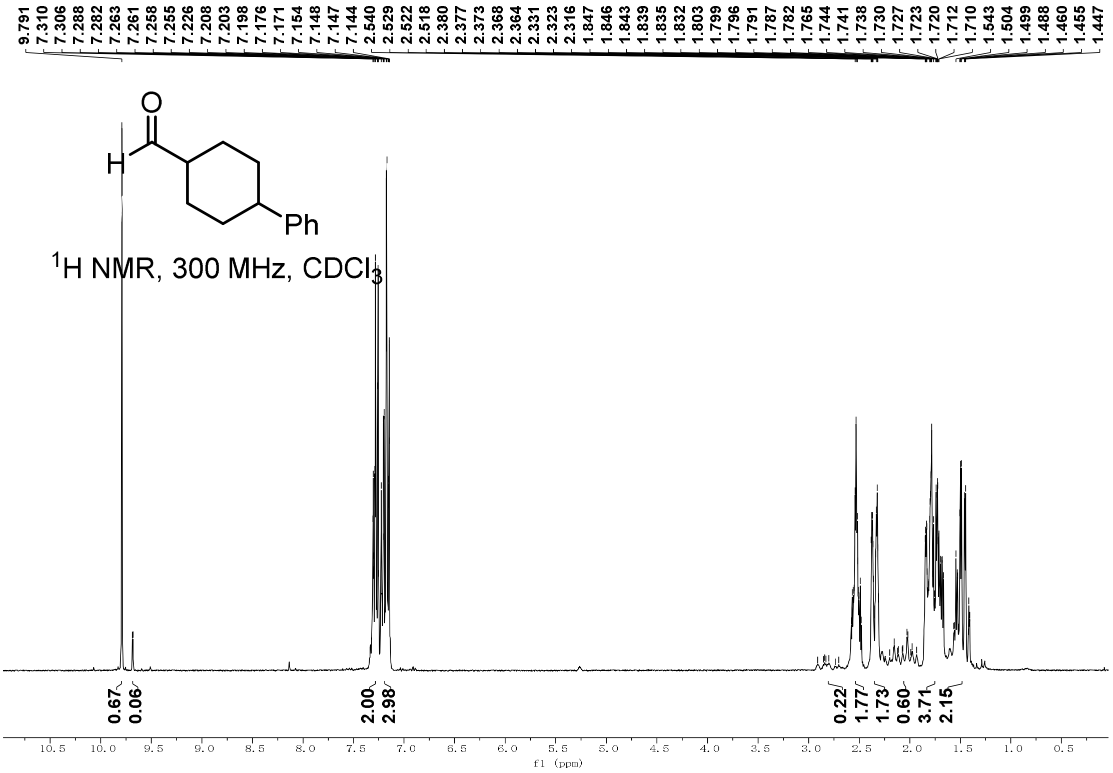
9.791 7.310 7.306 7.288 7.282 7.263 7.261 7.258 7.255 7.226 7.208 7.203 7.198 7.176 7.171 7.154 7.148 7.147 7.144 2.540 2.529 2.522 2.518 2.380 2.377 2.373 2.368 2.364 2.331 2.323 2.316 1.847 1.846 1.843 1.839 1.835 1.832 1.803 1.799 1.796 1.791 1.787 1.782 1.765 1.744 1.741 1.738 1.730 1.727 1.723 1.720 1.712 1.710 1.543 1.504 1.499 1.488 1.460 1.455 1.447
O
H
Ph
^{1}H NMR, 300 MHz, CDCl$_3$
0.67 0.06 2.00 2.98 0.22 1.77 1.73 0.60 3.71 2.15
10.5 10.0 9.5 9.0 8.5 8.0 7.5 7.0 6.5 6.0 5.5 5.0 4.5 4.0 3.5 3.0 2.5 2.0 1.5 1.0 0.5
f1 (ppm)

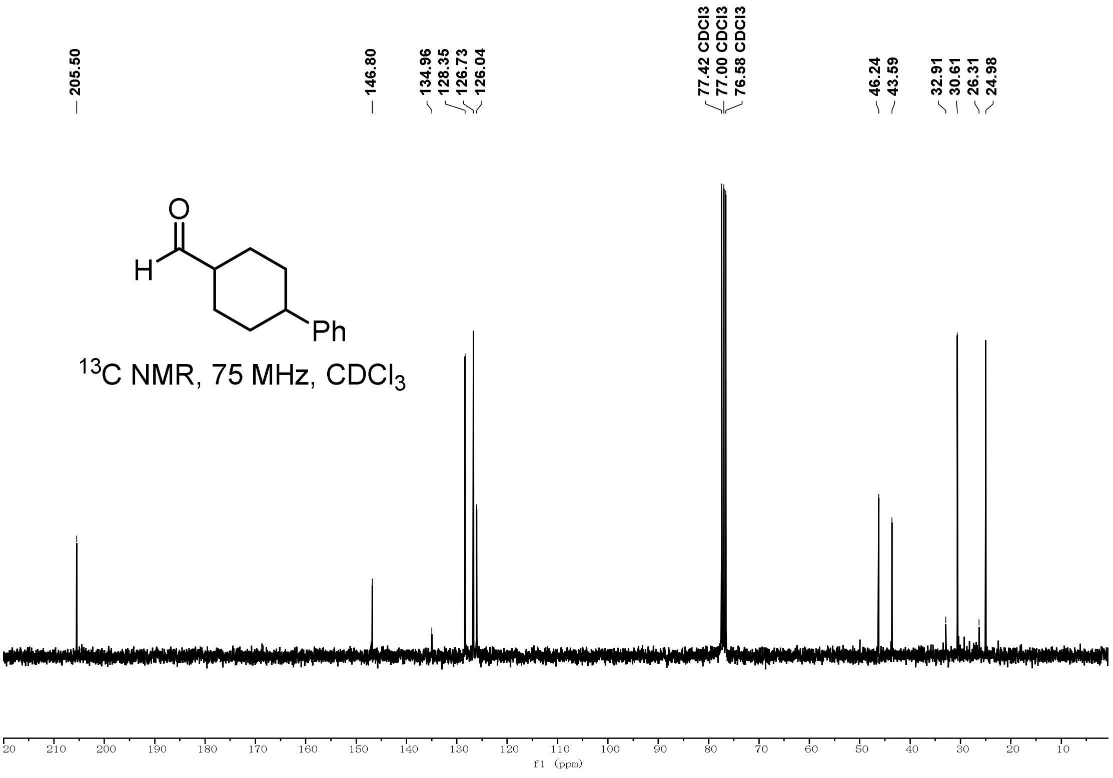
205.50 146.80 134.96 128.35 126.73 126.04 77.42 CDCl3 77.00 CDCl3 76.58 CDCl3 46.24 43.59 32.91 30.61 26.31 24.98
O
H
Ph
^{13}C NMR, 75 MHz, CDCl$_3$
20 210 200 190 180 170 160 150 140 130 120 110 100 90 80 70 60 50 40 30 20 10
f1 (ppm)

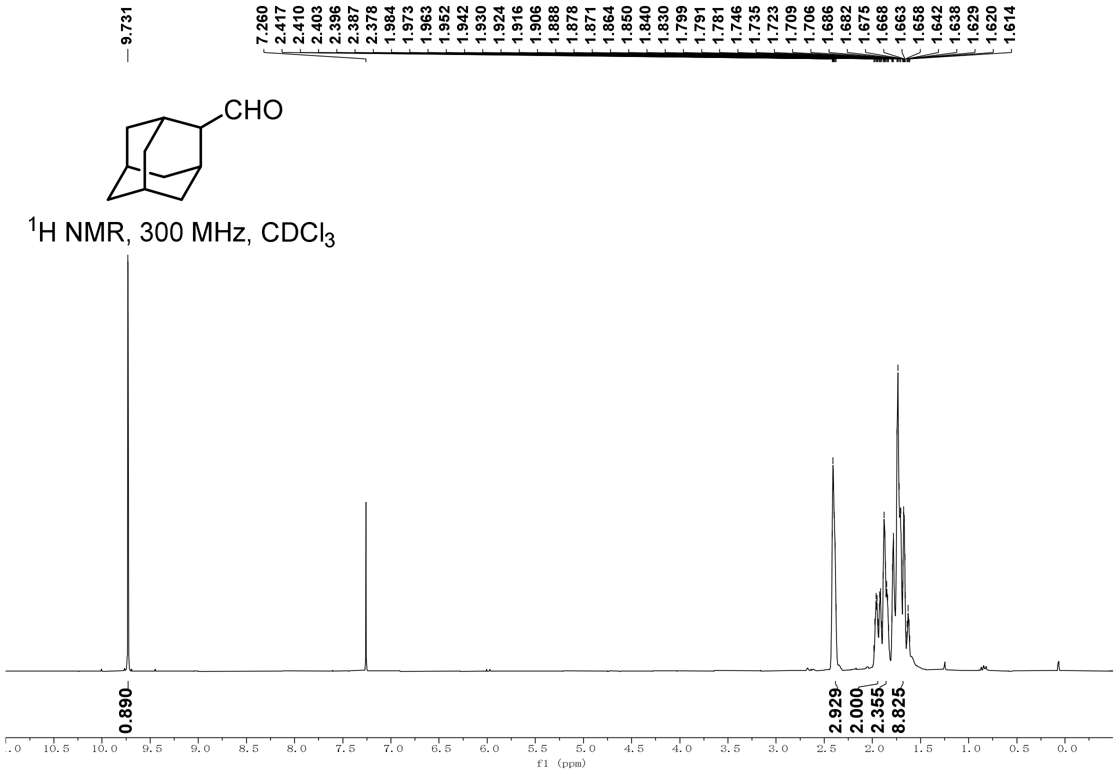
9.731
7.260
2.417
2.410
2.403
2.396
2.387
2.378
1.984
1.973
1.963
1.952
1.942
1.930
1.924
1.916
1.906
1.888
1.878
1.871
1.864
1.850
1.840
1.830
1.799
1.791
1.781
1.746
1.735
1.723
1.709
1.706
1.686
1.682
1.675
1.668
1.663
1.658
1.642
1.638
1.629
1.620
1.614
CHO
1H NMR, 300 MHz, CDCl3
0.890
2.929
2.000
2.355
8.825
f1 (ppm)

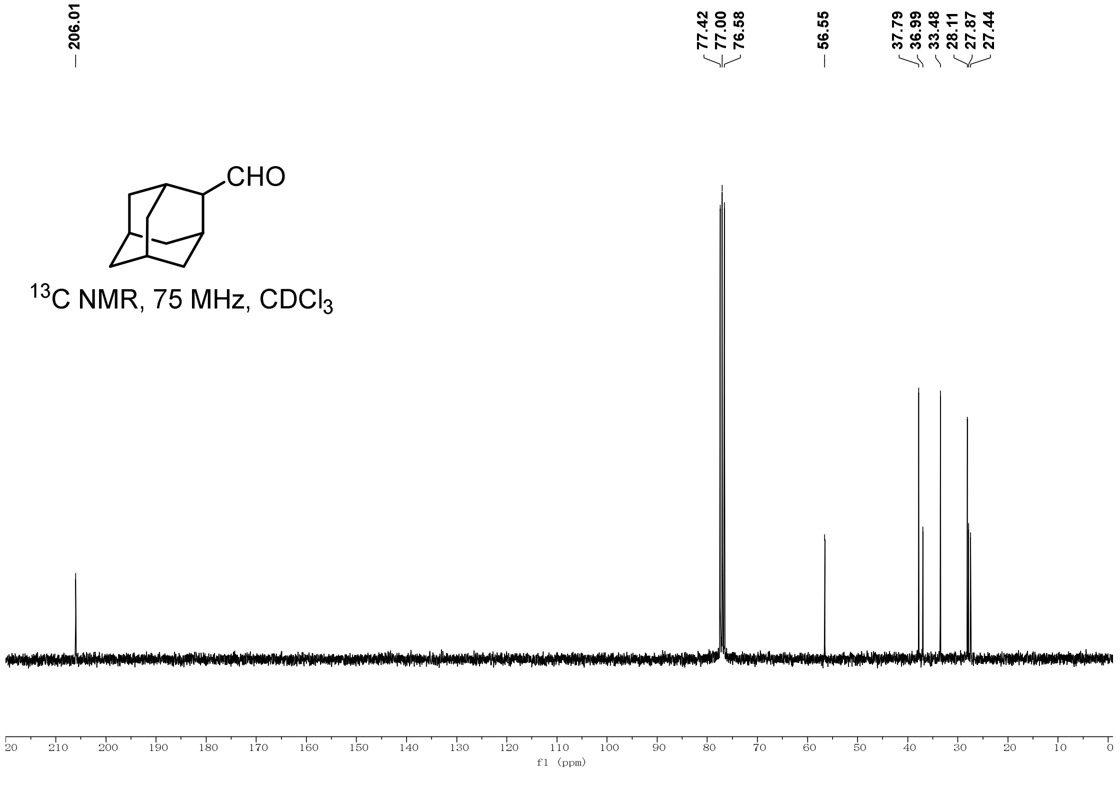
206.01
77.42
77.00
76.58
56.55
37.79
36.99
33.48
28.11
27.87
27.44
CHO
13C NMR, 75 MHz, CDCl3
f1 (ppm)

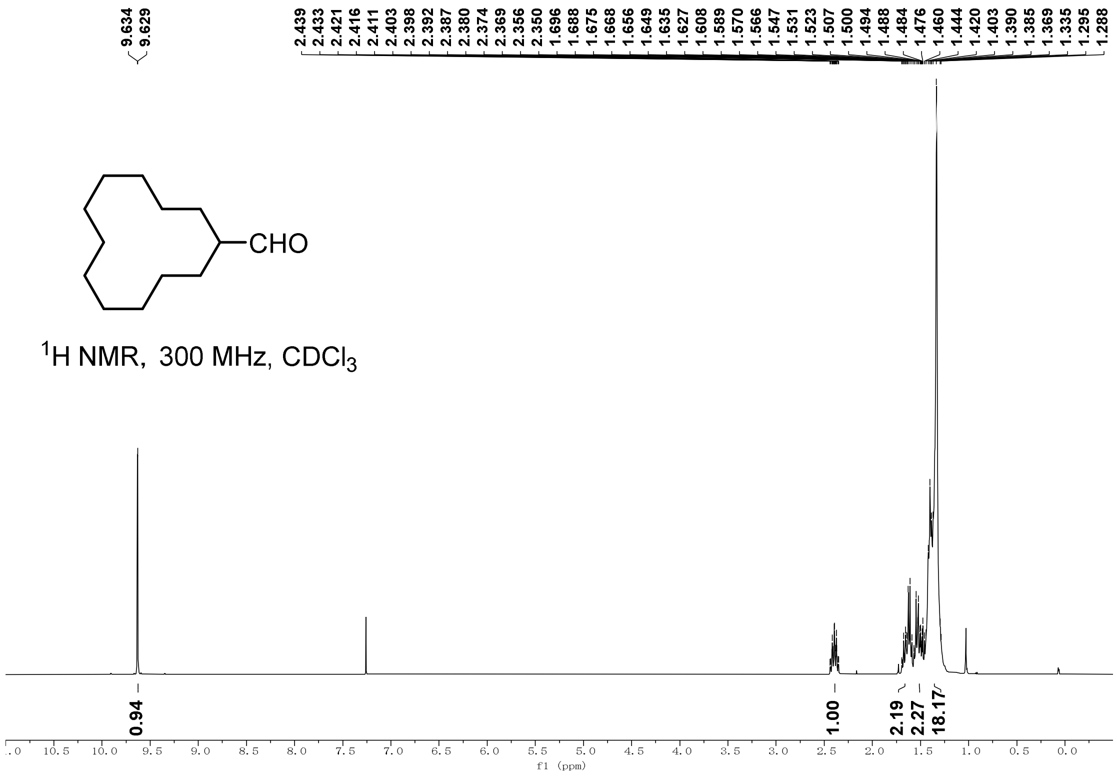
9.634
9.629
2.439
2.433
2.421
2.416
2.411
2.403
2.398
2.392
2.387
2.380
2.374
2.369
2.356
2.350
1.696
1.688
1.675
1.668
1.656
1.649
1.635
1.627
1.608
1.589
1.570
1.566
1.547
1.531
1.523
1.507
1.500
1.494
1.488
1.484
1.476
1.460
1.444
1.420
1.403
1.390
1.385
1.369
1.335
1.295
1.288
CHO
1H NMR, 300 MHz, CDCl3
0.94
1.00
2.19
2.27
18.17
f1 (ppm)

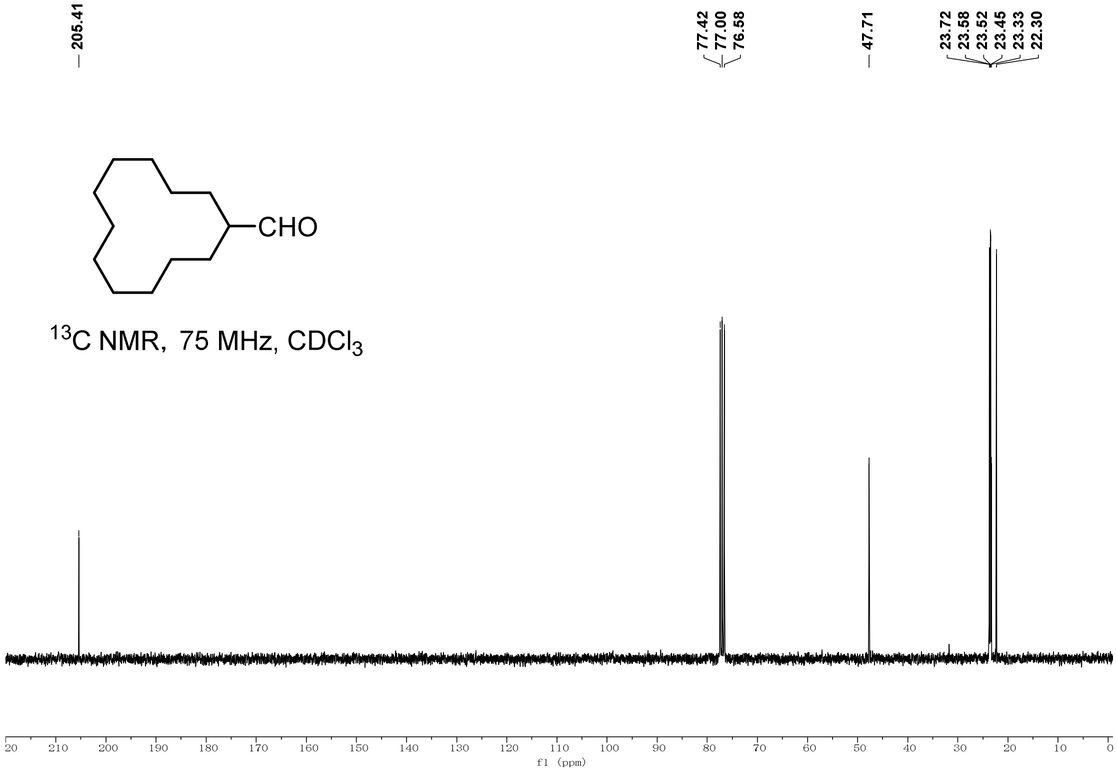
205.41
77.42
77.00
76.58
47.71
23.72
23.58
23.52
23.45
23.33
22.30
CHO
13C NMR, 75 MHz, CDCl3
f1 (ppm)

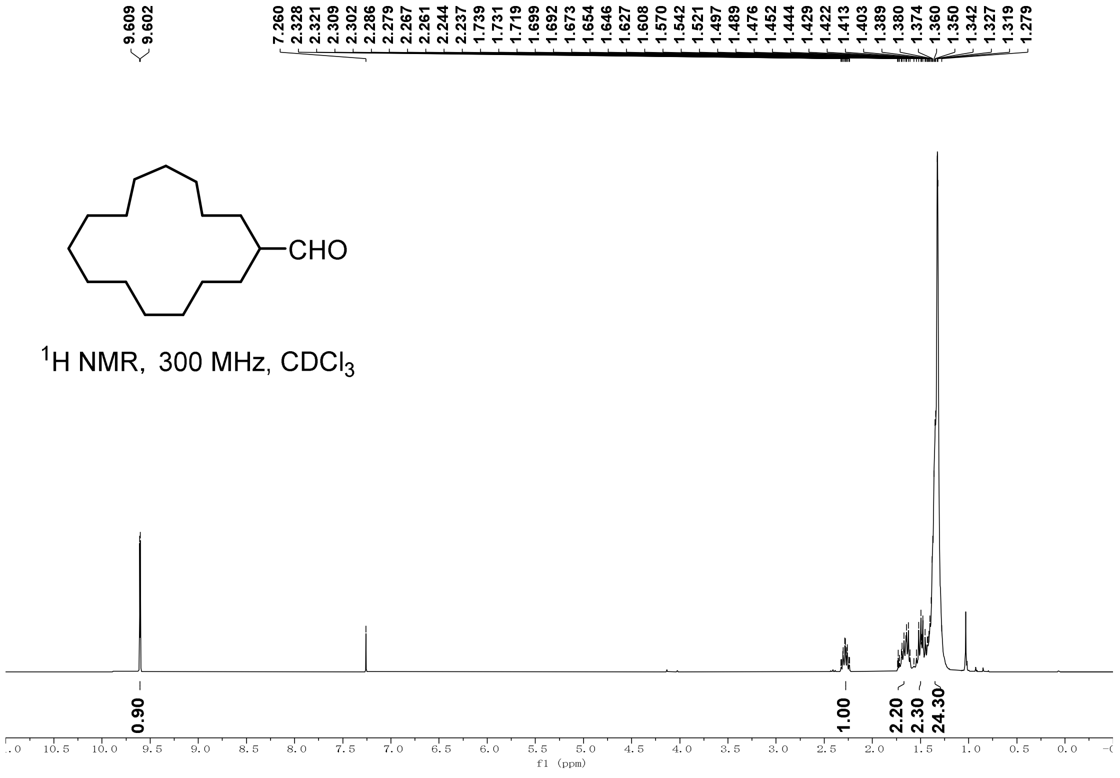
9.609
9.602
7.260
2.328
2.321
2.309
2.302
2.286
2.279
2.267
2.261
2.244
2.237
1.739
1.731
1.719
1.699
1.692
1.673
1.654
1.646
1.627
1.608
1.570
1.542
1.521
1.497
1.489
1.476
1.452
1.444
1.429
1.422
1.413
1.403
1.389
1.380
1.374
1.360
1.350
1.342
1.327
1.319
1.279
CHO
^{1}H NMR, 300 MHz, $CDCl_3$
0.90
1.00
2.20
2.30
24.30
f1 (ppm)

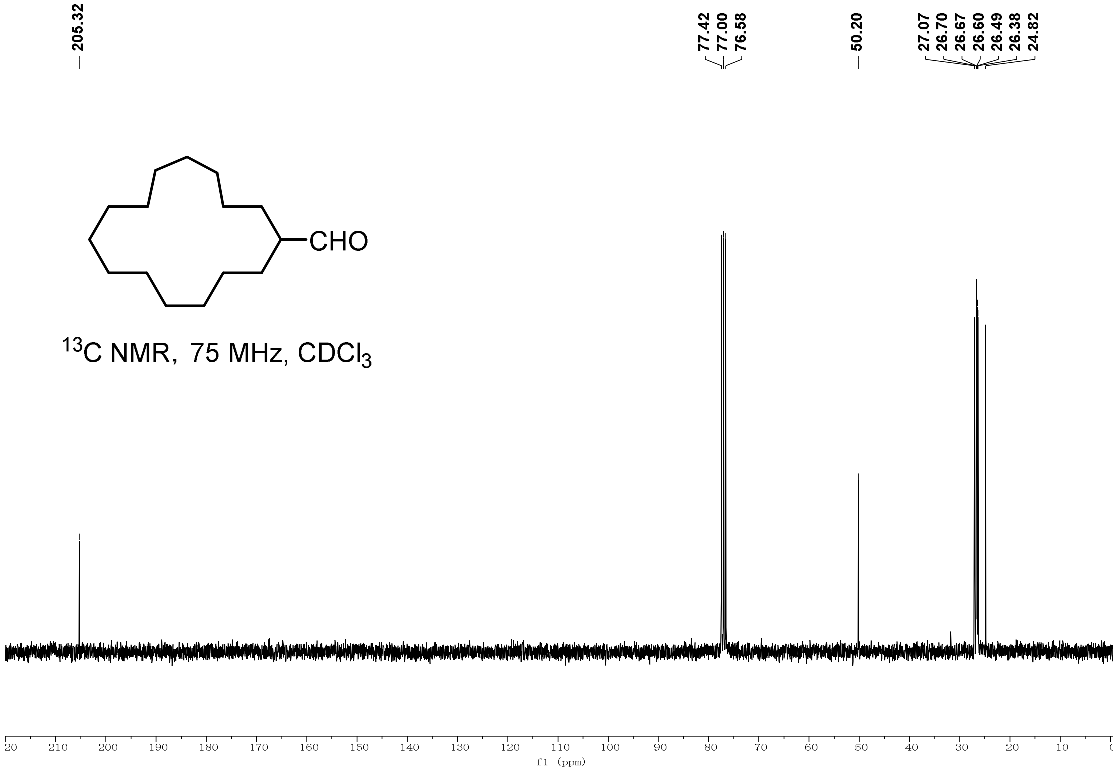
205.32
77.42
77.00
76.58
50.20
27.07
26.70
26.67
26.60
26.49
26.38
24.82
CHO
^{13}C NMR, 75 MHz, $CDCl_3$
f1 (ppm)

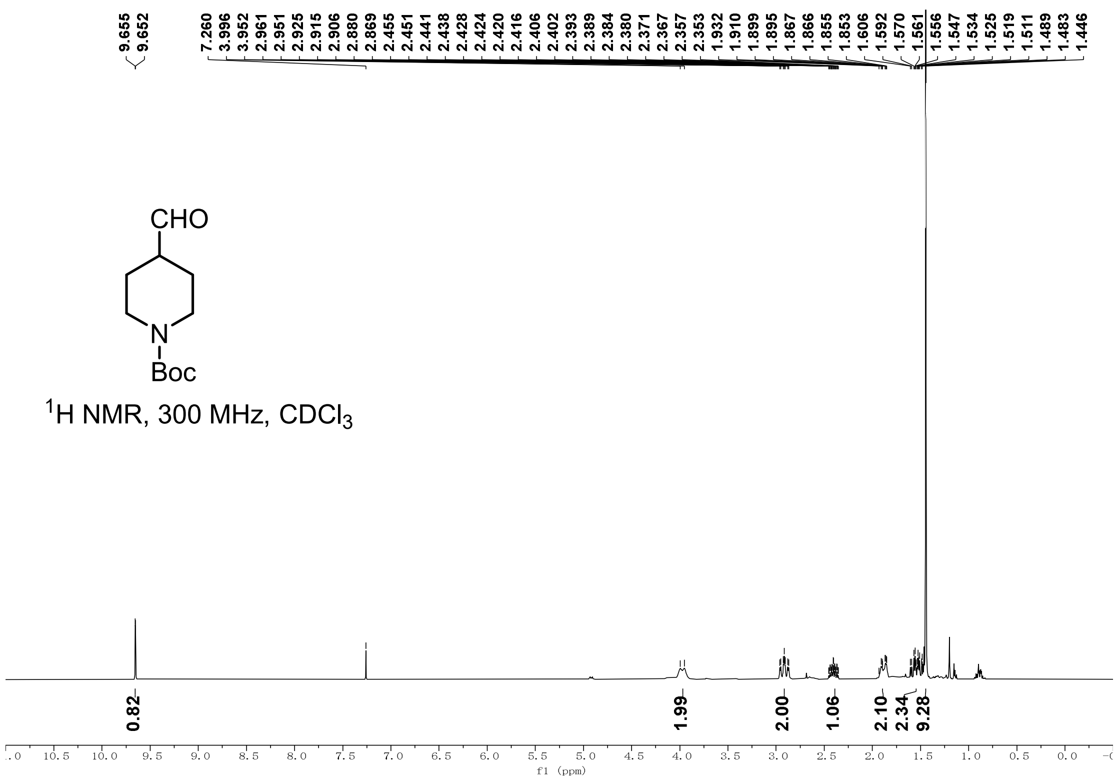

^{1}H NMR, 300 MHz, $CDCl_3$

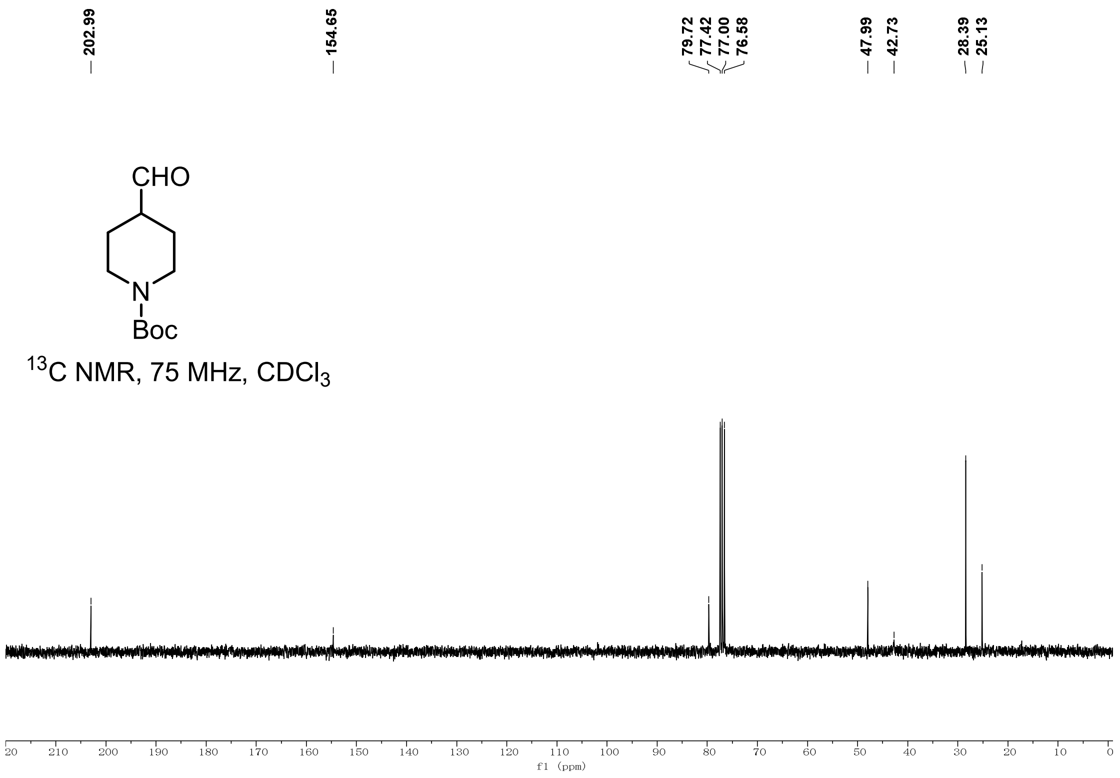

^{13}C NMR, 75 MHz, $CDCl_3$

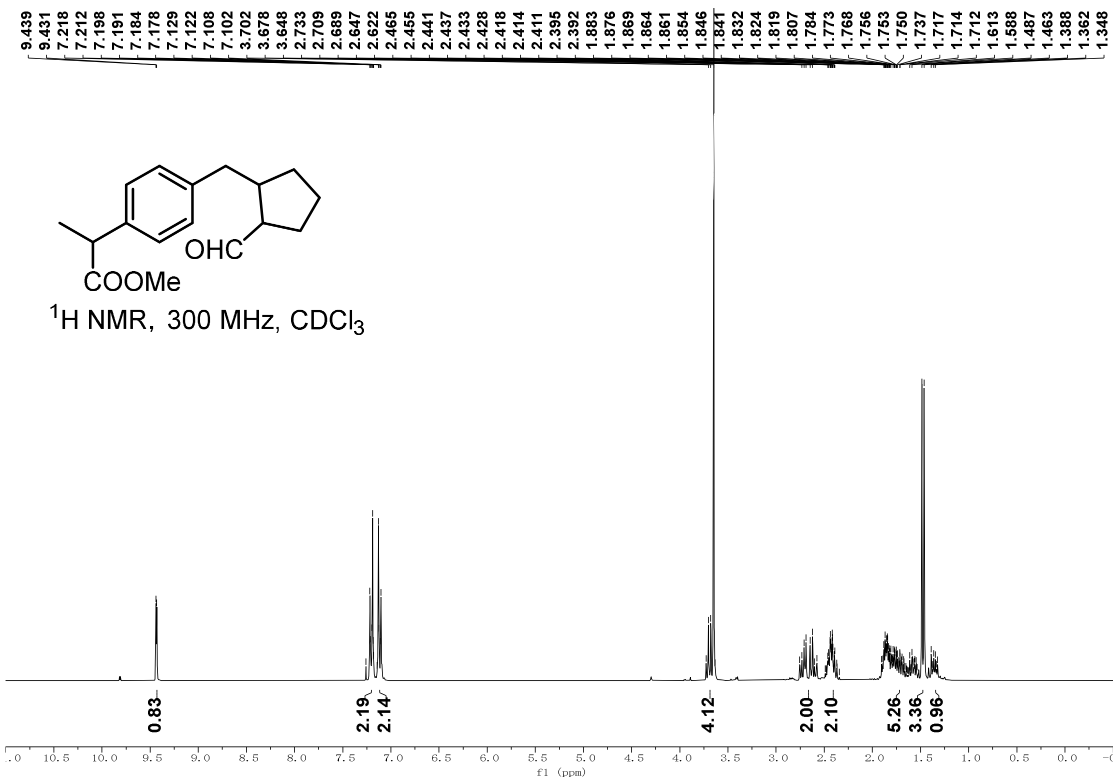
9.439 9.431 7.218 7.212 7.198 7.191 7.184 7.178 7.129 7.122 7.108 7.102 3.702 3.678 3.648 2.733 2.709 2.689 2.647 2.622 2.465 2.455 2.441 2.437 2.433 2.428 2.418 2.414 2.411 2.395 2.392 1.883 1.876 1.869 1.864 1.861 1.854 1.846 1.841 1.832 1.824 1.819 1.807 1.784 1.773 1.768 1.756 1.753 1.750 1.737 1.717 1.714 1.712 1.613 1.588 1.487 1.463 1.388 1.362 1.348
OHC
COOMe
^{1}H NMR, 300 MHz, $CDCl_3$
0.83
2.19
2.14
4.12
2.00
2.10
5.26
3.36
0.96
f1 (ppm)

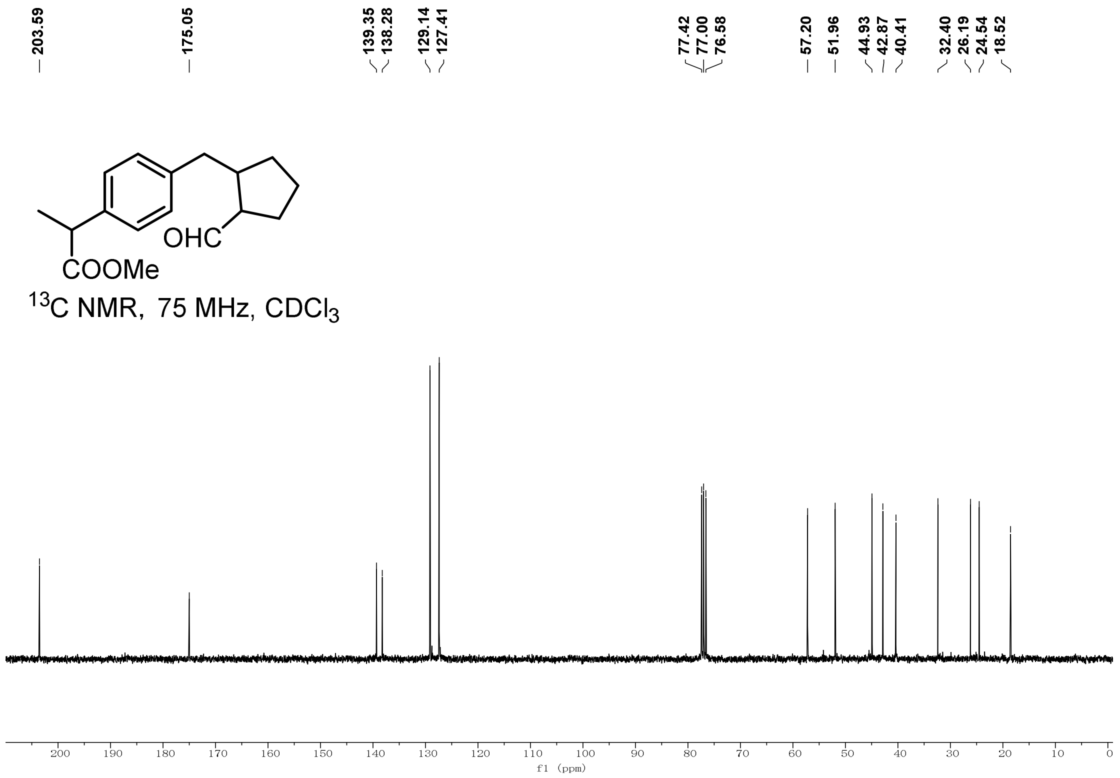
203.59
175.05
139.35 138.28
129.14 127.41
77.42 77.00 76.58
57.20
51.96
44.93 42.87 40.41
32.40
26.19 24.54
18.52
OHC
COOMe
^{13}C NMR, 75 MHz, $CDCl_3$
f1 (ppm)

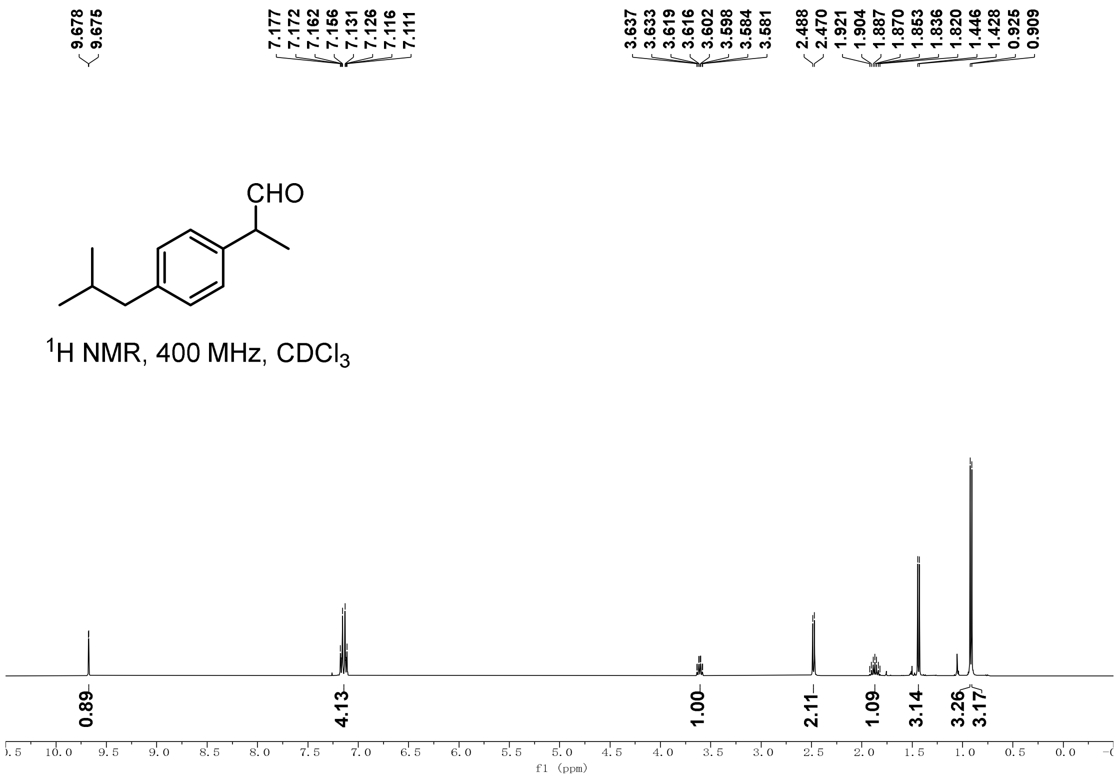
9.678
9.675
7.177
7.172
7.162
7.156
7.131
7.126
7.116
7.111
3.637
3.633
3.619
3.616
3.602
3.598
3.584
3.581
2.488
2.470
1.921
1.904
1.887
1.870
1.853
1.836
1.820
1.446
1.428
0.925
0.909
CHO
^{1}H NMR, 400 MHz, $CDCl_3$
0.89
4.13
1.00
2.11
1.09
3.14
3.26
3.17
f1 (ppm)

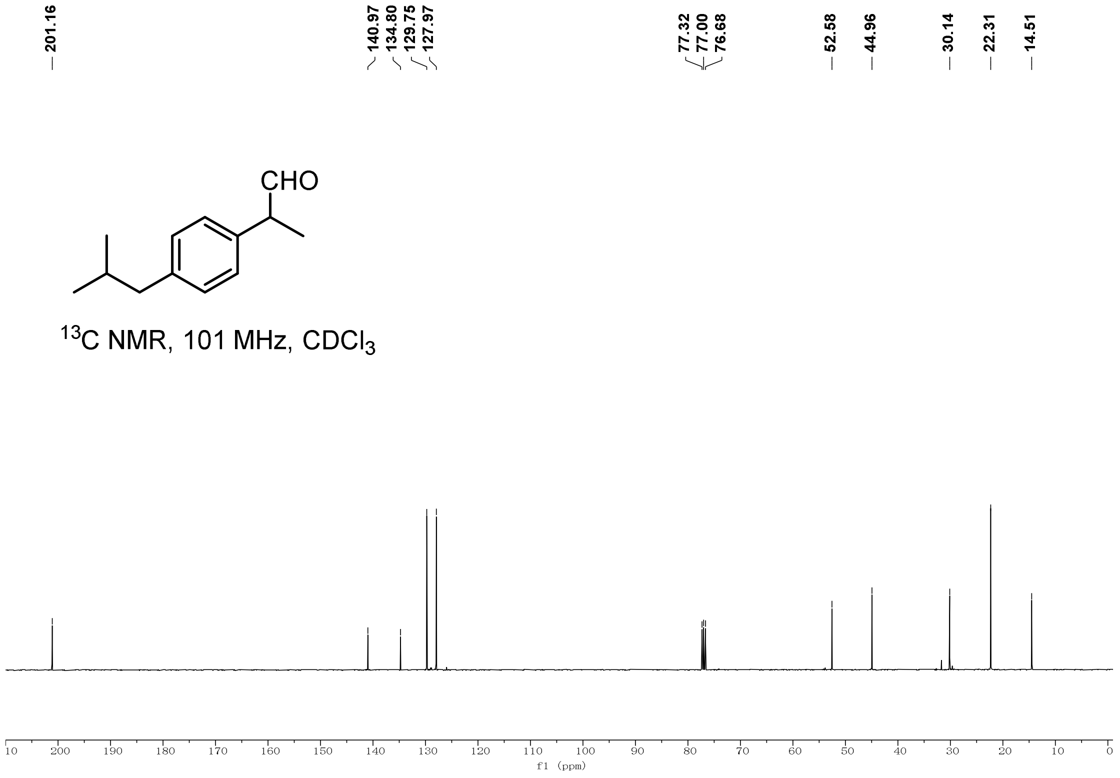
201.16
140.97
134.80
129.75
127.97
77.32
77.00
76.68
52.58
44.96
30.14
22.31
14.51
CHO
^{13}C NMR, 101 MHz, $CDCl_3$
f1 (ppm)

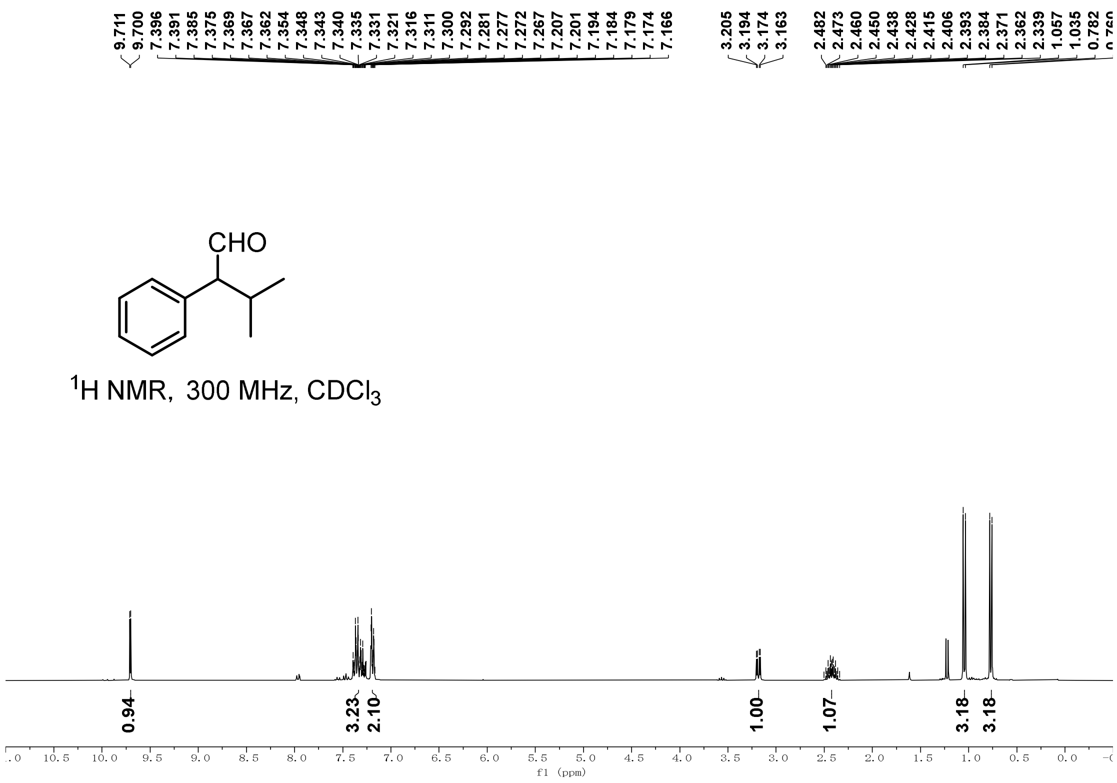
9.711
9.700
7.396
7.391
7.385
7.375
7.369
7.367
7.362
7.354
7.348
7.343
7.340
7.335
7.331
7.321
7.316
7.311
7.300
7.292
7.281
7.277
7.272
7.267
7.207
7.201
7.194
7.184
7.179
7.174
7.166
3.205
3.194
3.174
3.163
2.482
2.473
2.460
2.450
2.438
2.428
2.415
2.406
2.393
2.384
2.371
2.362
2.339
1.057
1.035
0.782
CHO
^{1}H NMR, 300 MHz, CDCl$_3$
0.94
3.23
2.10
1.00
1.07
3.18
3.18
f1 (ppm)

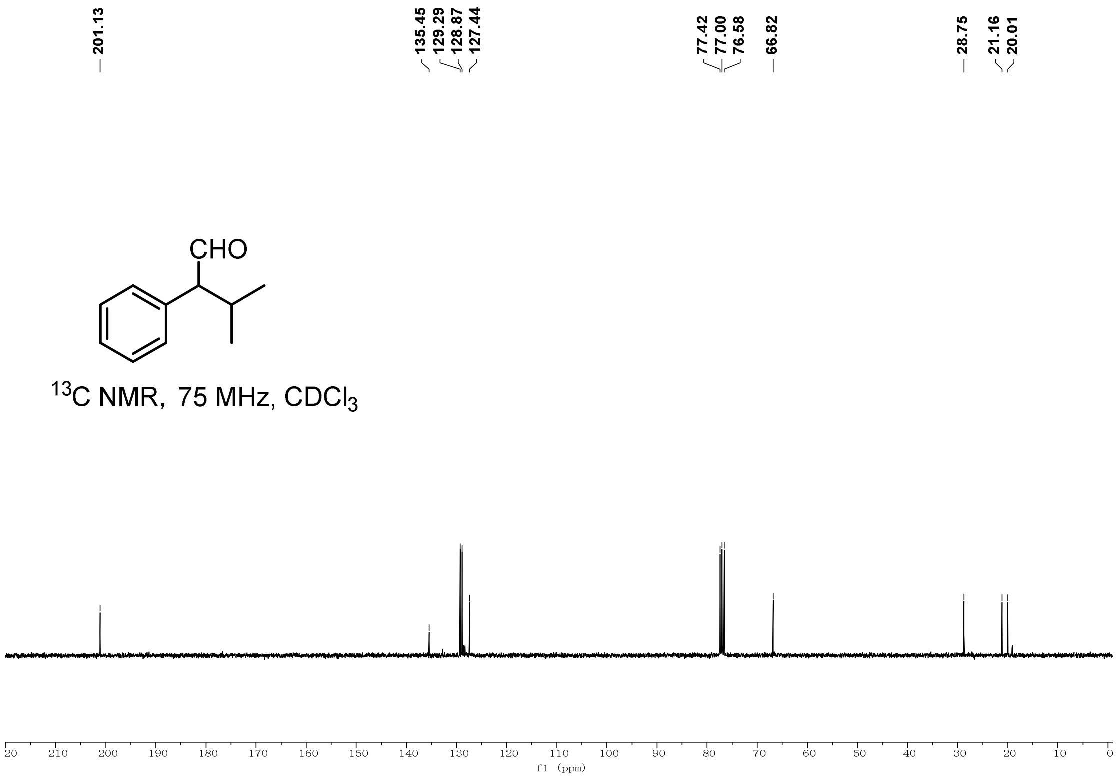
201.13
135.45
129.29
128.87
127.44
77.42
77.00
76.58
66.82
28.75
21.16
20.01
CHO
^{13}C NMR, 75 MHz, CDCl$_3$
f1 (ppm)

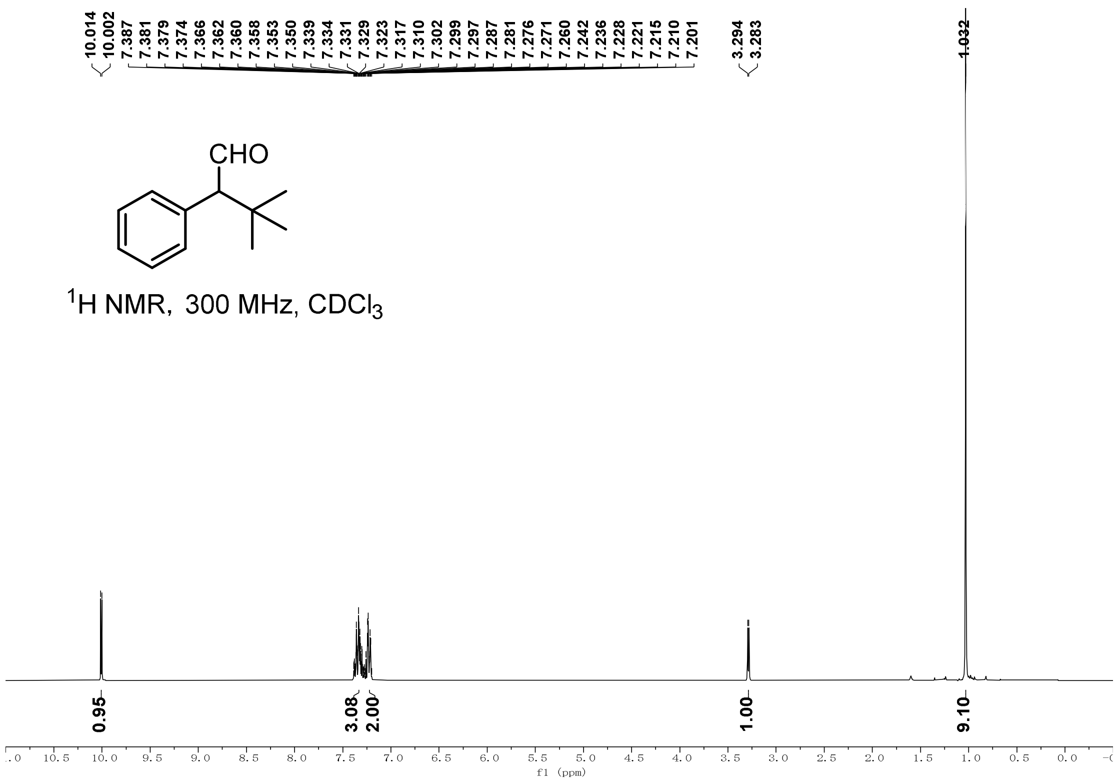
10.014
10.002
7.387
7.381
7.379
7.374
7.366
7.362
7.360
7.358
7.353
7.350
7.339
7.334
7.331
7.329
7.323
7.317
7.310
7.302
7.299
7.297
7.287
7.281
7.276
7.271
7.260
7.242
7.236
7.228
7.221
7.215
7.210
7.201
3.294
3.283
1.032
CHO
^{1}H NMR, 300 MHz, $CDCl_3$
0.95
3.08
2.00
1.00
9.10
f1 (ppm)

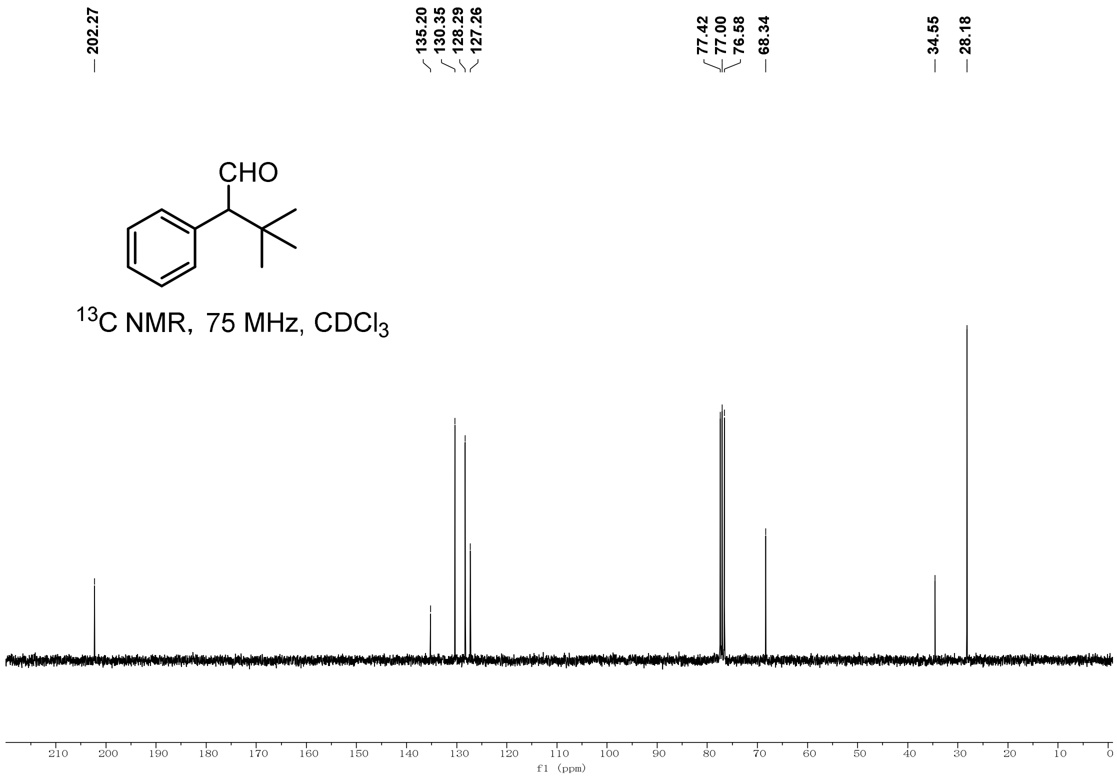
202.27
135.20
130.35
128.29
127.26
77.42
77.00
76.58
68.34
34.55
28.18
CHO
^{13}C NMR, 75 MHz, $CDCl_3$
f1 (ppm)

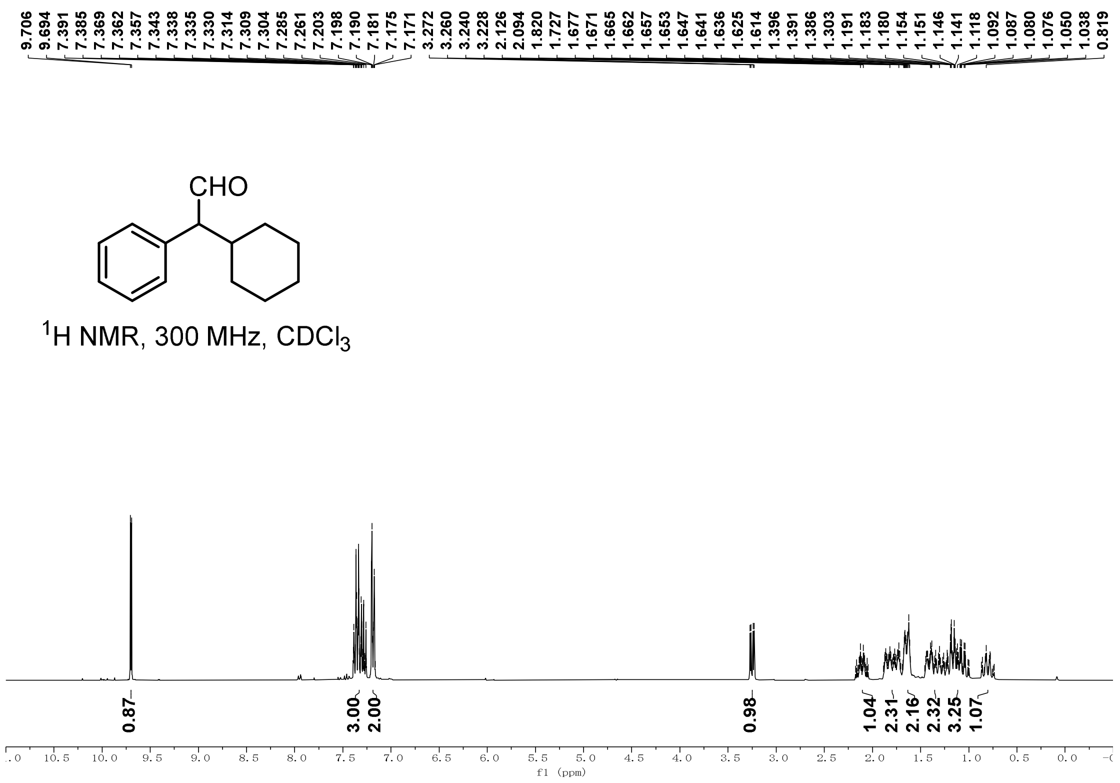

^{1}H NMR, 300 MHz, $CDCl_3$

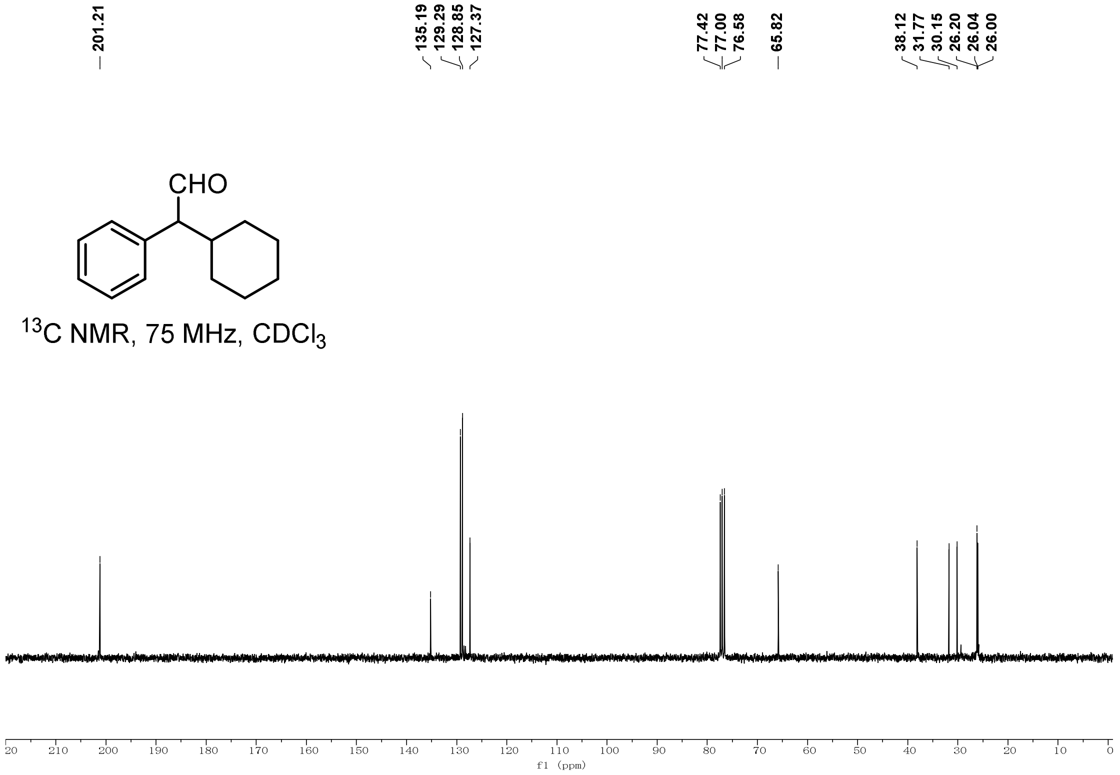

^{13}C NMR, 75 MHz, $CDCl_3$

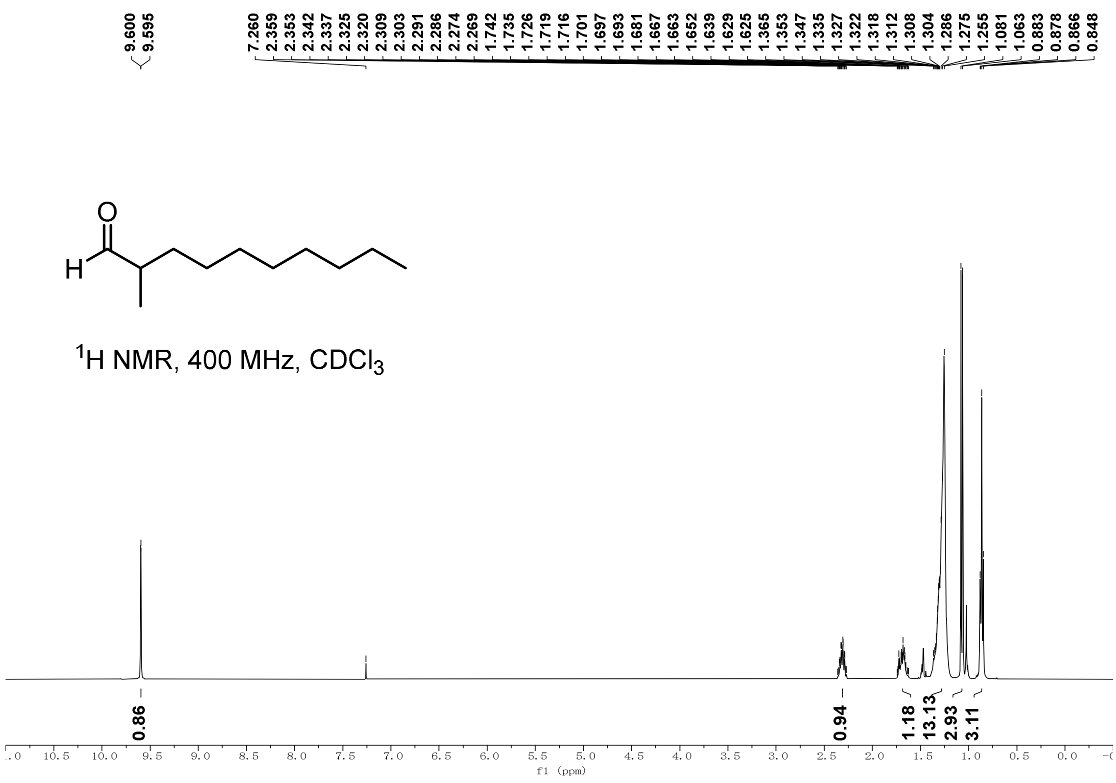
1H NMR, 400 MHz, CDCl3
f1 (ppm)

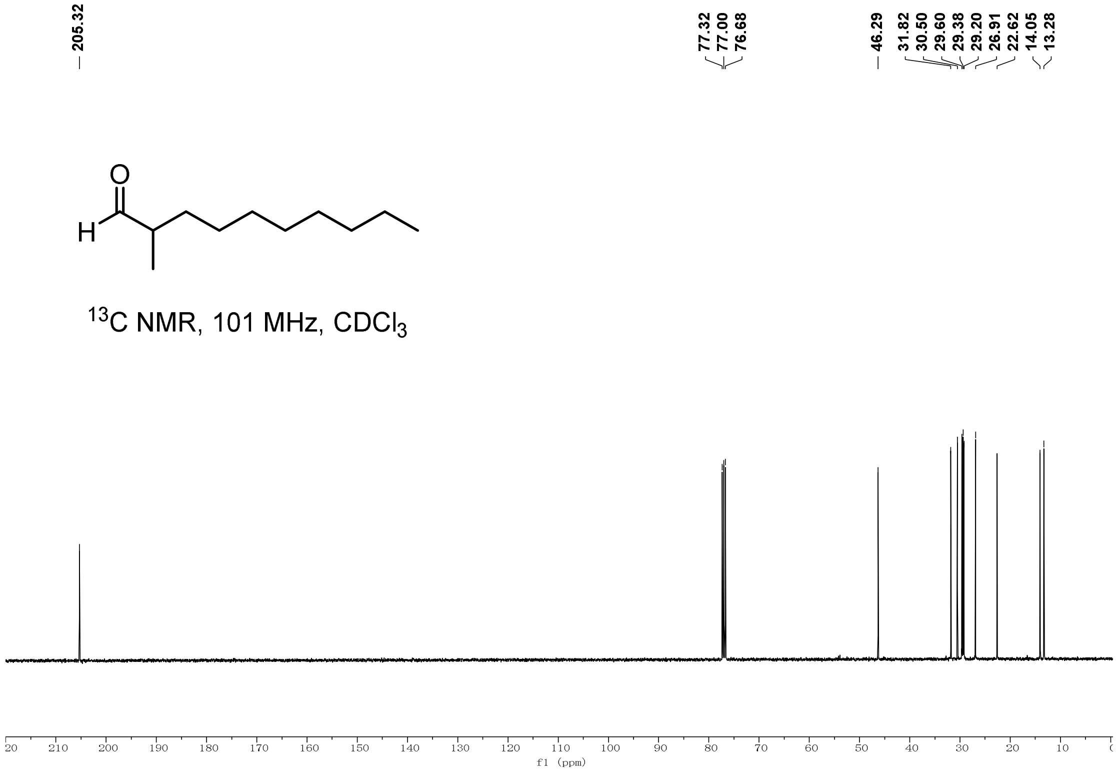
13C NMR, 101 MHz, CDCl3
f1 (ppm)

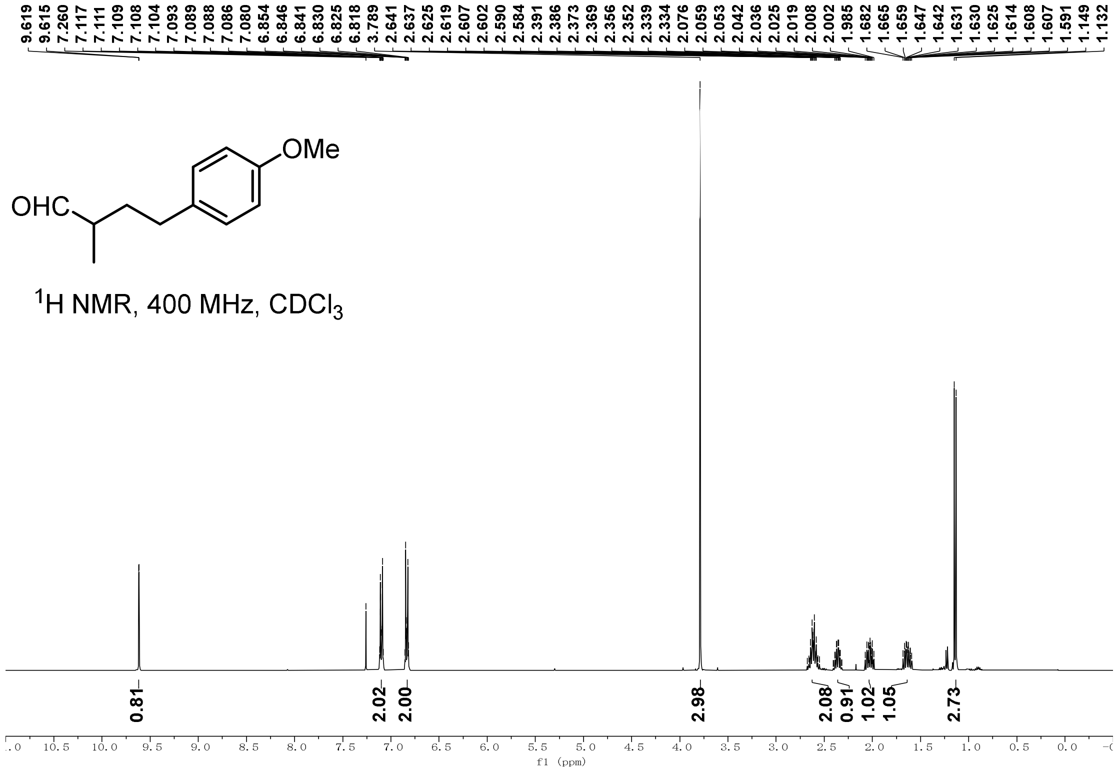
9.619 9.615 7.260 7.117 7.111 7.109 7.108 7.104 7.093 7.089 7.088 7.086 7.080 6.854 6.846 6.841 6.830 6.825 6.818 3.789 2.641 2.637 2.625 2.619 2.607 2.602 2.590 2.584 2.391 2.386 2.373 2.369 2.356 2.352 2.339 2.334 2.076 2.059 2.053 2.042 2.036 2.025 2.019 2.008 2.002 1.985 1.682 1.665 1.659 1.647 1.642 1.631 1.630 1.625 1.614 1.608 1.607 1.591 1.149 1.132
OMe
OHC
1H NMR, 400 MHz, CDCl3
0.81
2.02
2.00
2.98
2.08
0.91
1.02
1.05
2.73
f1 (ppm)

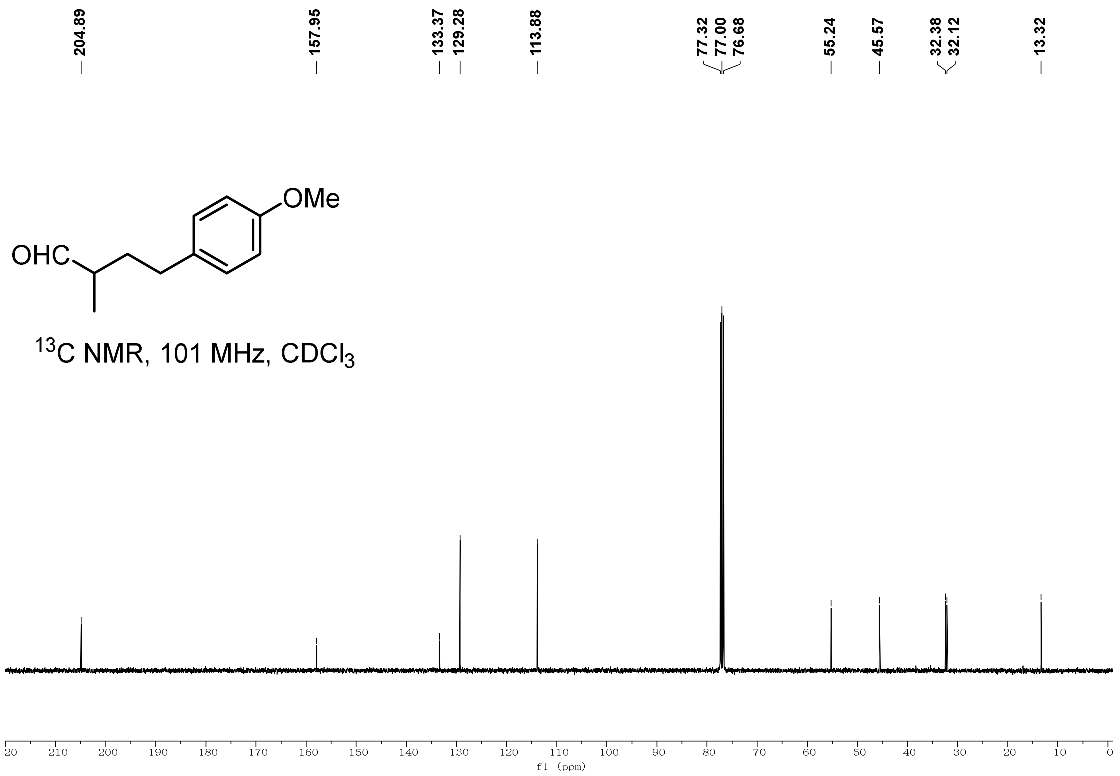
204.89
157.95
133.37
129.28
113.88
77.32
77.00
76.68
55.24
45.57
32.38
32.12
13.32
OMe
OHC
13C NMR, 101 MHz, CDCl3
f1 (ppm)

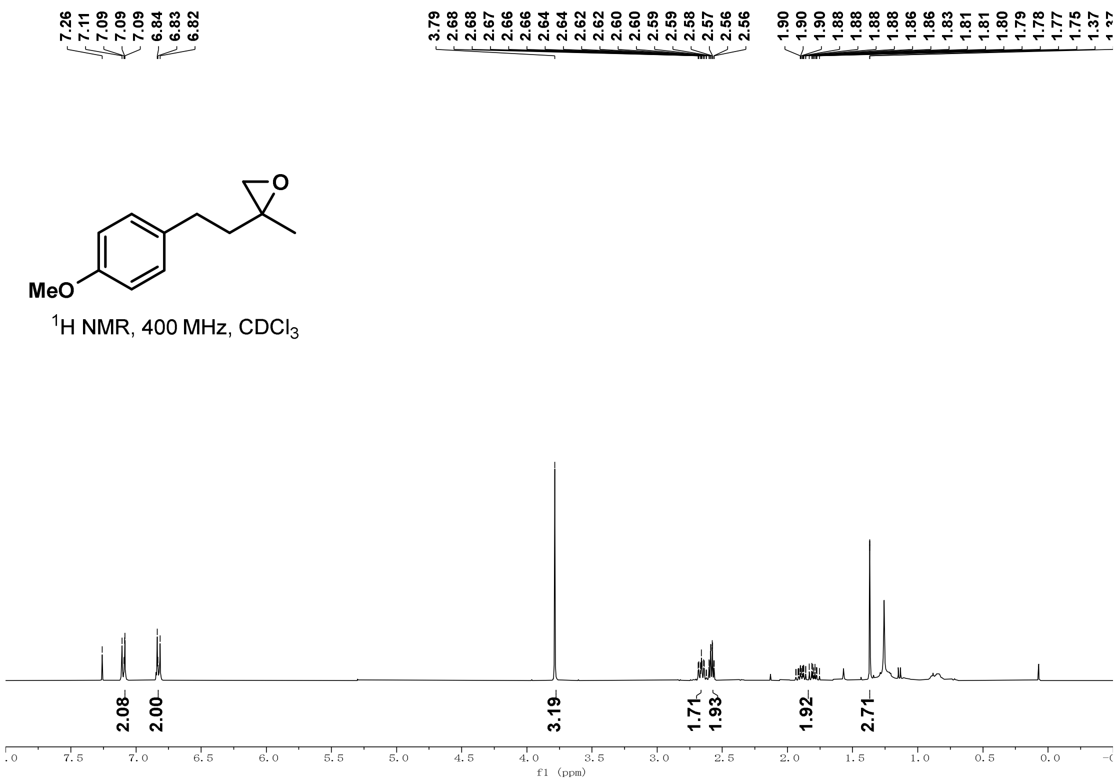
MeO
^{1}H NMR, 400 MHz, CDCl$_3$
f1 (ppm)

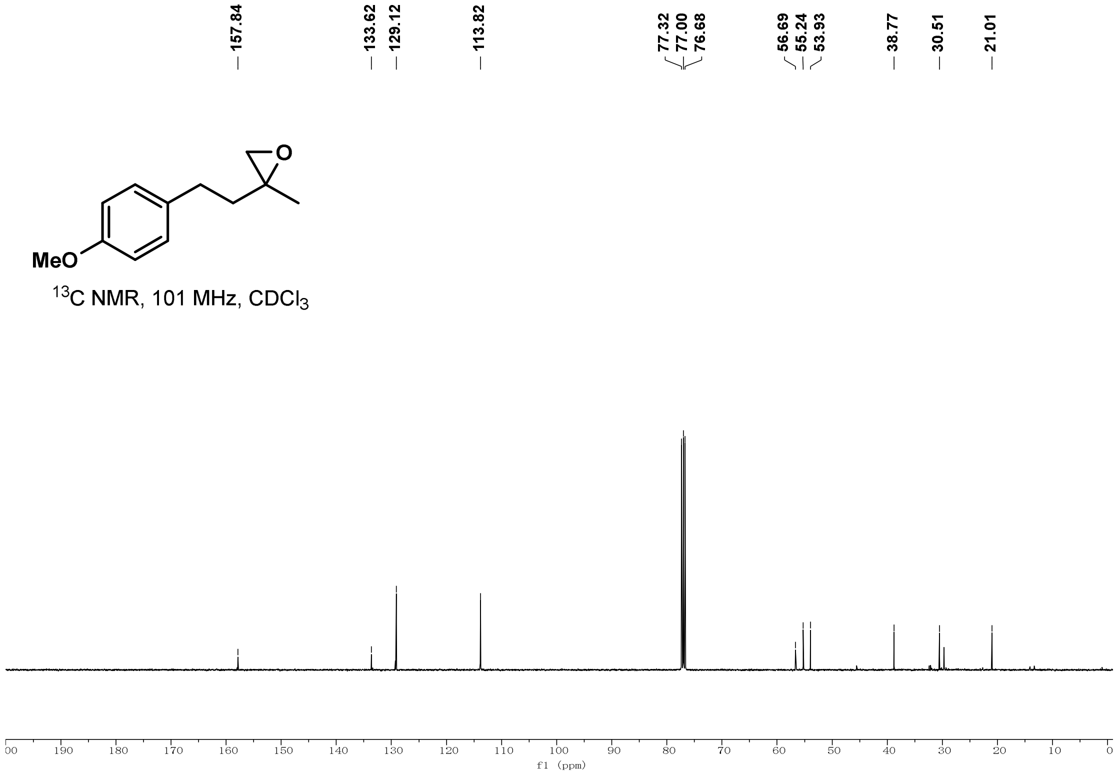
157.84
133.62
129.12
113.82
77.32
77.00
76.68
56.69
55.24
53.93
38.77
30.51
21.01
MeO
^{13}C NMR, 101 MHz, CDCl$_3$
f1 (ppm)

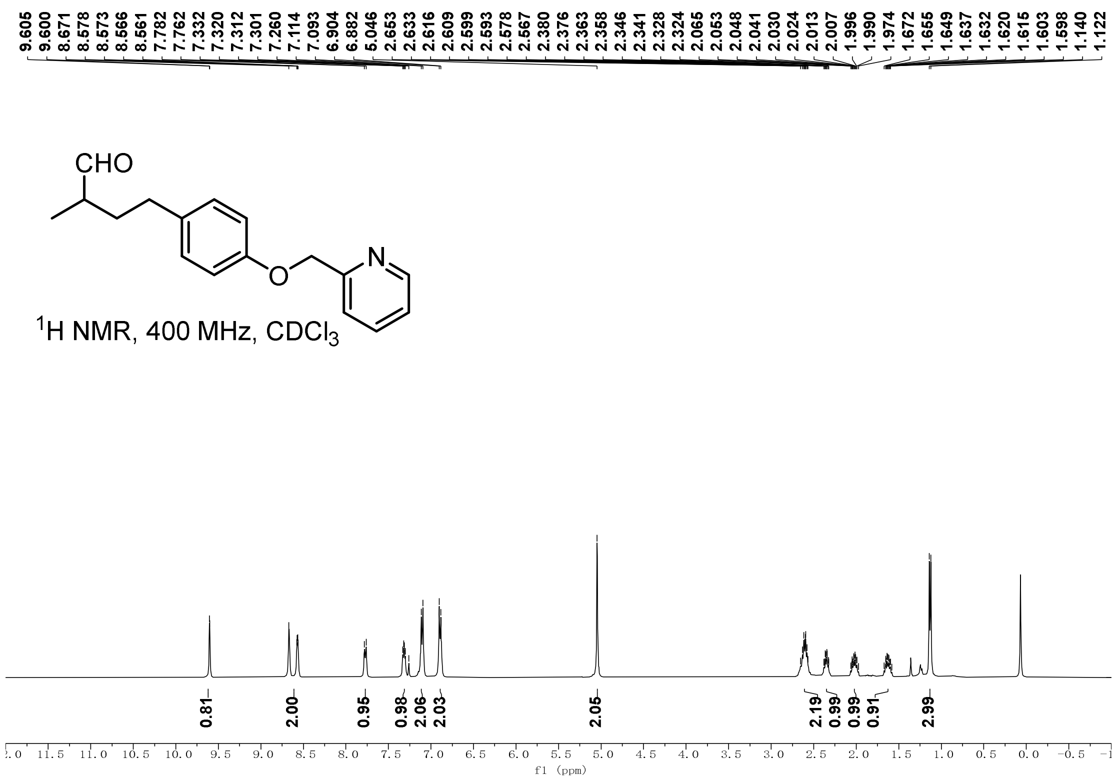
9.605 9.600 8.671 8.578 8.573 8.566 8.561 7.782 7.762 7.332 7.320 7.312 7.301 7.260 7.114 7.093 6.904 6.882 5.046 2.653 2.633 2.616 2.609 2.599 2.593 2.578 2.567 2.380 2.376 2.363 2.358 2.346 2.341 2.328 2.324 2.065 2.053 2.048 2.041 2.030 2.024 2.013 2.007 1.996 1.990 1.974 1.672 1.655 1.649 1.637 1.632 1.620 1.615 1.603 1.598 1.140 1.122
CHO
O
N
^{1}H NMR, 400 MHz, $CDCl_3$
0.81 2.00 0.95 0.98 2.06 2.03 2.05 2.19 0.99 0.99 0.91 2.99
f1 (ppm)

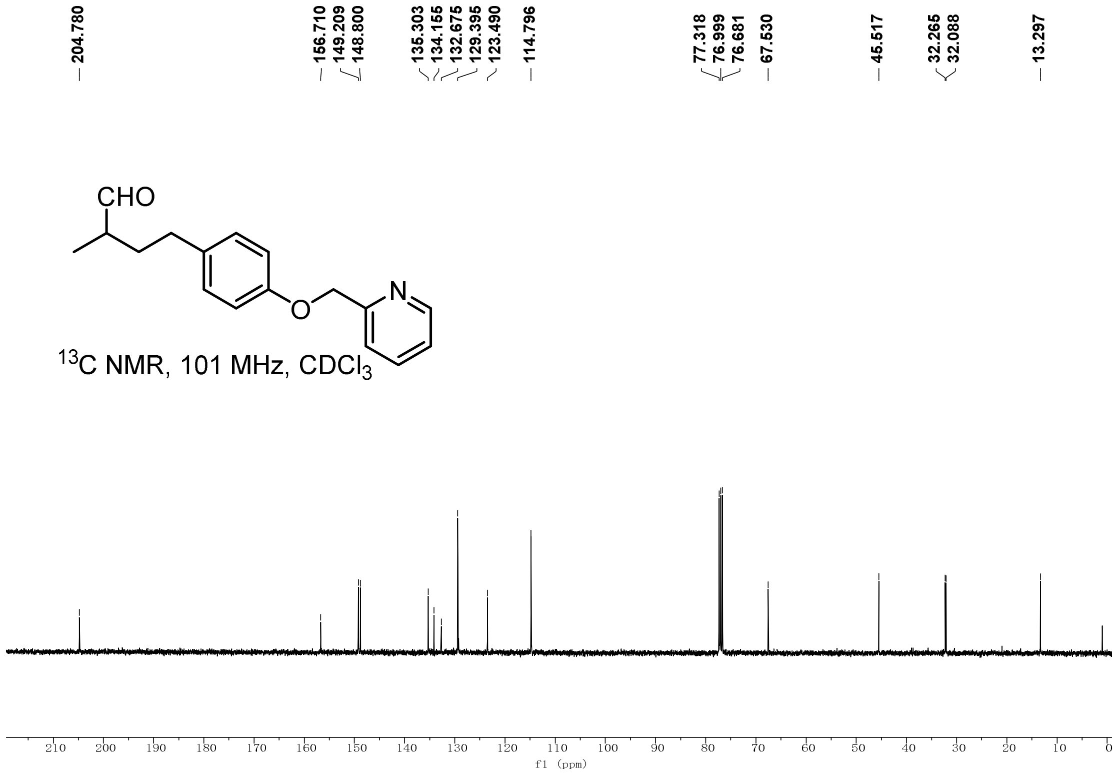
204.780
156.710 149.209 148.800
135.303 134.155 132.675 129.395 123.490
114.796
77.318 76.999 76.681 67.530
45.517
32.265 32.088
13.297
CHO
O
N
^{13}C NMR, 101 MHz, $CDCl_3$
210 200 190 180 170 160 150 140 130 120 110 100 90 80 70 60 50 40 30 20 10 0
f1 (ppm)

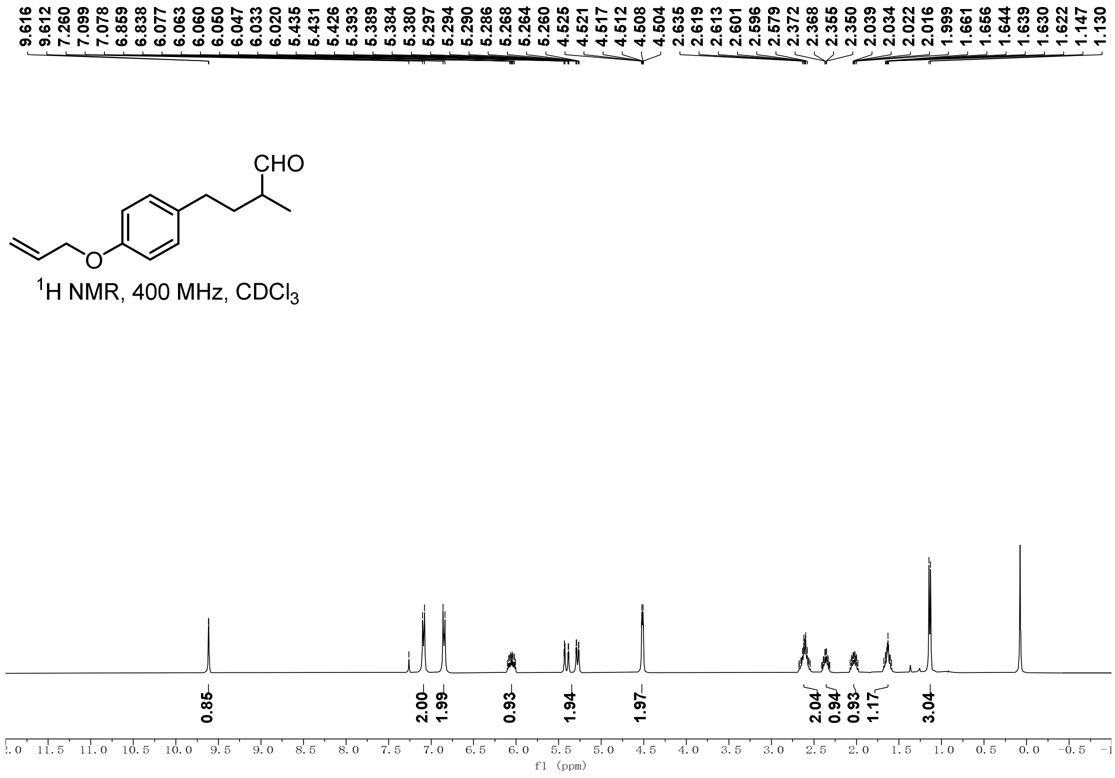
9.616 9.612 7.260 7.099 7.078 6.859 6.838 6.077 6.063 6.060 6.050 6.047 6.033 6.020 5.435 5.431 5.426 5.393 5.389 5.384 5.380 5.297 5.294 5.290 5.286 5.268 5.264 5.260 4.525 4.521 4.517 4.512 4.508 4.504 2.635 2.619 2.613 2.601 2.596 2.579 2.372 2.368 2.355 2.350 2.039 2.034 2.022 2.016 1.999 1.661 1.656 1.644 1.639 1.630 1.622 1.147 1.130
CHO
O
1H NMR, 400 MHz, CDCl3
0.85 2.00 1.99 0.93 1.94 1.97 2.04 0.94 0.93 1.17 3.04
f1 (ppm)

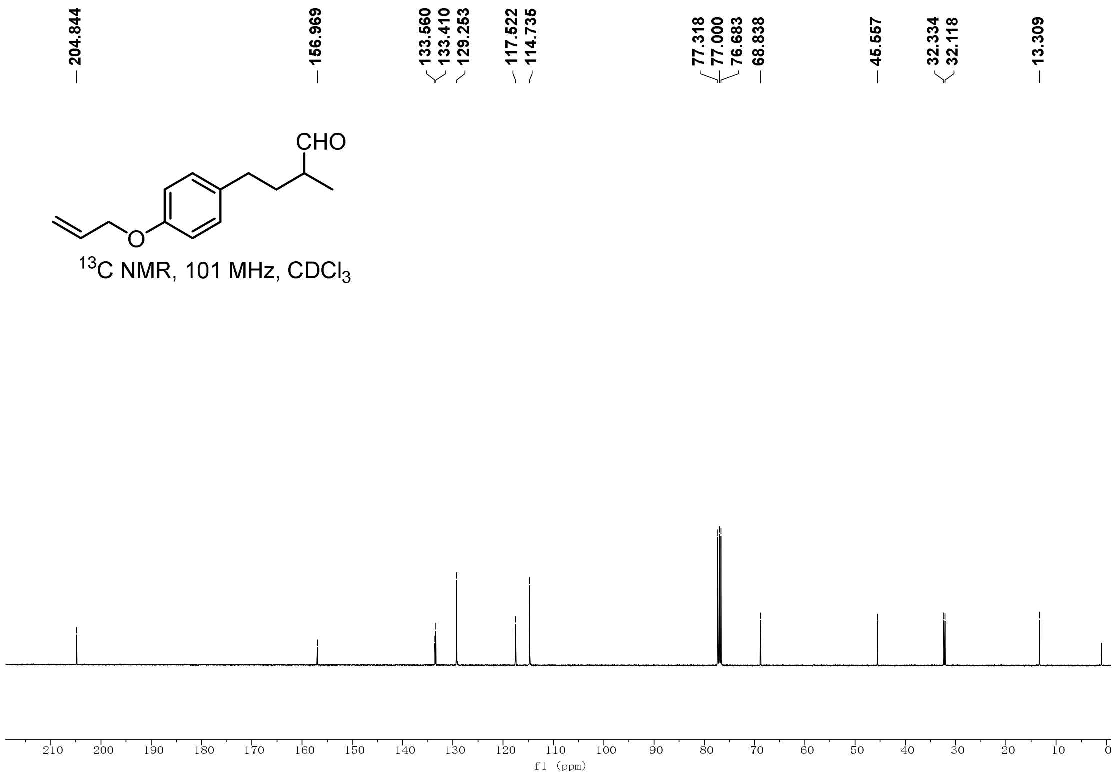
204.844 156.969 133.560 133.410 129.253 117.522 114.735 77.318 77.000 76.683 68.838 45.557 32.334 32.118 13.309
CHO
O
13C NMR, 101 MHz, CDCl3
f1 (ppm)

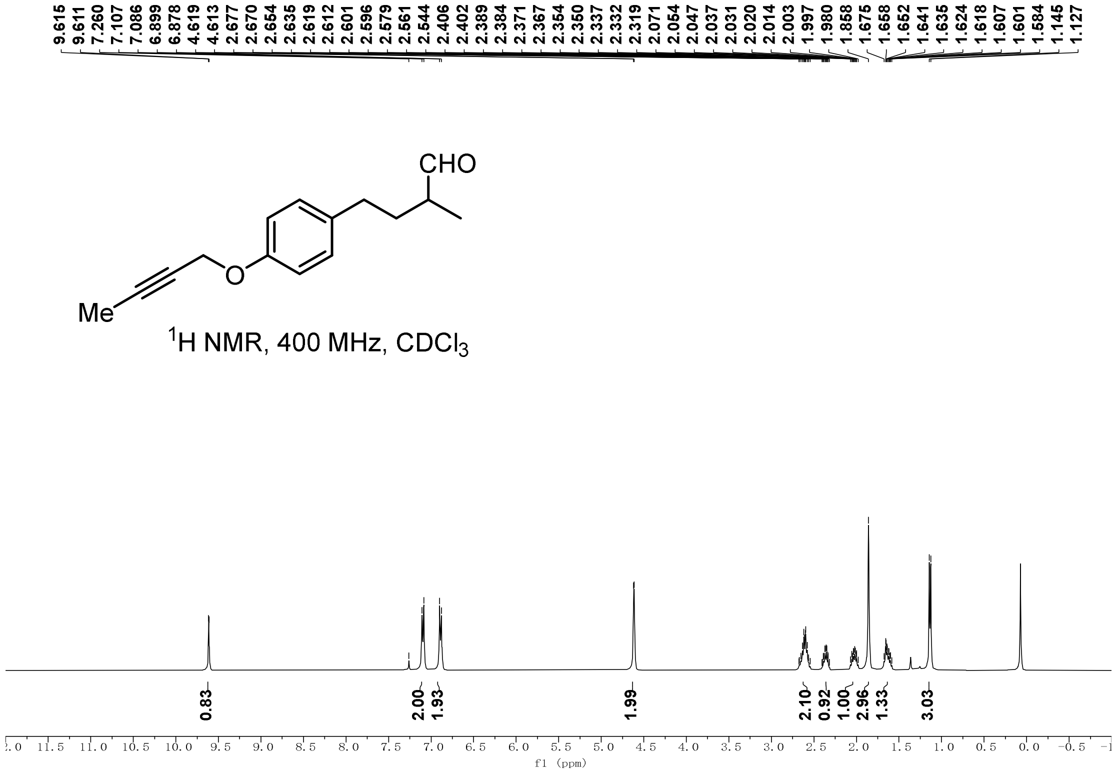
9.615 9.611 7.260 7.107 7.086 6.899 6.878 4.619 4.613 2.677 2.670 2.654 2.635 2.619 2.612 2.601 2.596 2.579 2.561 2.544 2.406 2.402 2.389 2.384 2.371 2.367 2.354 2.350 2.337 2.332 2.319 2.071 2.054 2.047 2.037 2.031 2.020 2.014 2.003 1.997 1.980 1.858 1.675 1.658 1.652 1.641 1.635 1.624 1.618 1.607 1.601 1.584 1.145 1.127
CHO
O
Me
1H NMR, 400 MHz, CDCl3
0.83 2.00 1.93 1.99 2.10 0.92 1.00 2.96 1.33 3.03
f1 (ppm)

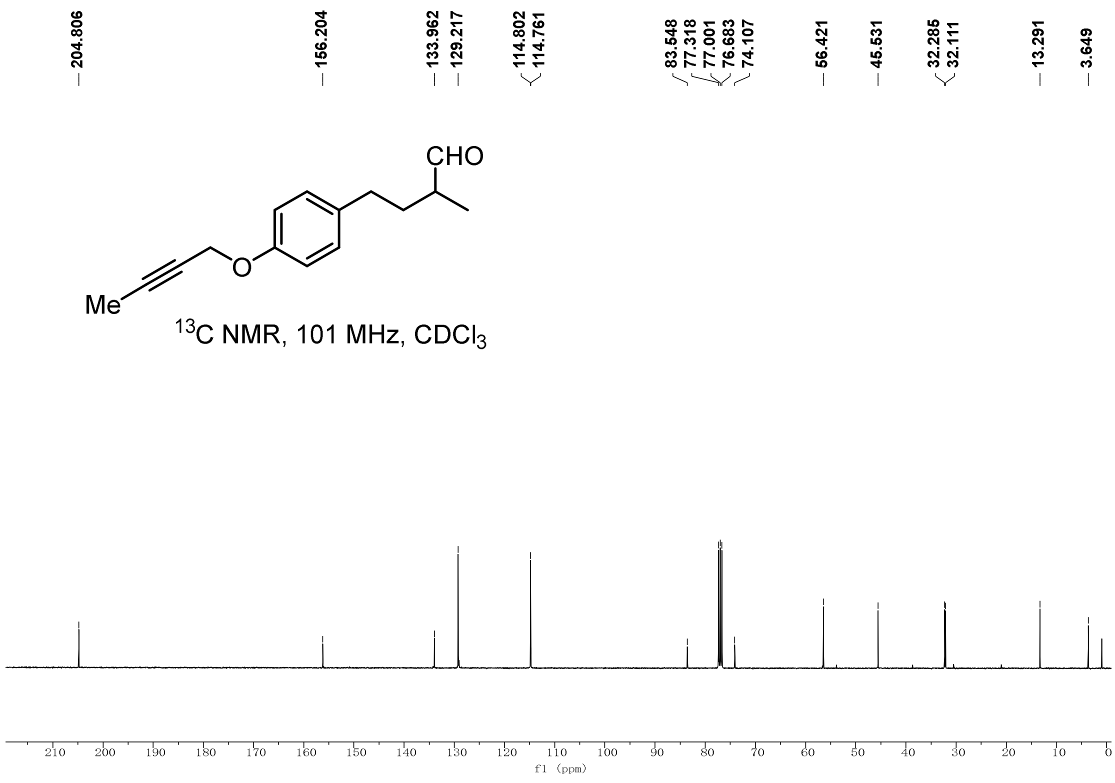
204.806 156.204 133.962 129.217 114.802 114.761 83.548 77.318 77.001 76.683 74.107 56.421 45.531 32.285 32.111 13.291 3.649
CHO
O
Me
13C NMR, 101 MHz, CDCl3
f1 (ppm)

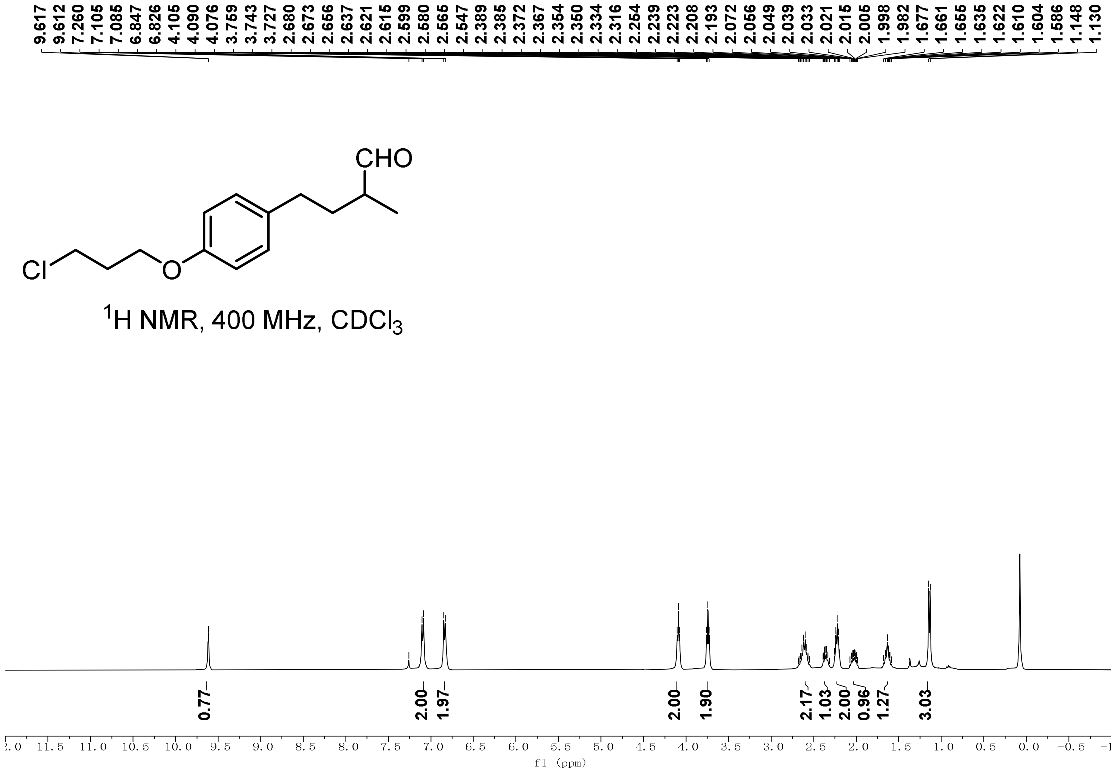
9.617 9.612 7.260 7.105 7.085 6.847 6.826 4.105 4.090 4.076 3.759 3.743 3.727 2.680 2.673 2.656 2.637 2.621 2.615 2.599 2.580 2.565 2.547 2.389 2.385 2.372 2.367 2.354 2.350 2.334 2.316 2.254 2.239 2.223 2.208 2.193 2.072 2.056 2.049 2.039 2.033 2.021 2.015 2.005 1.998 1.982 1.677 1.661 1.655 1.635 1.622 1.610 1.604 1.586 1.148 1.130
CHO
Cl
O
^{1}H NMR, 400 MHz, CDCl$_3$
0.77
2.00
1.97
2.00
1.90
2.17
1.03
2.00
0.96
1.27
3.03
f1 (ppm)

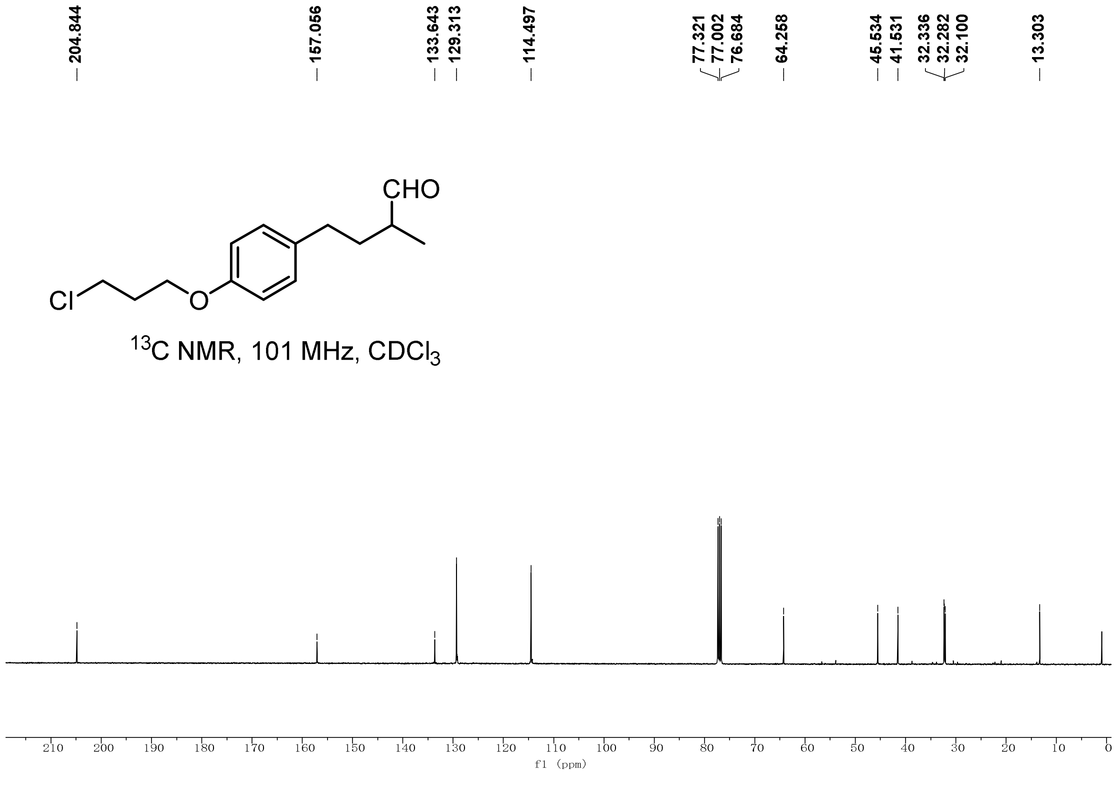
204.844
157.056
133.643
129.313
114.497
77.321
77.002
76.684
64.258
45.534
41.531
32.336
32.282
32.100
13.303
CHO
Cl
O
^{13}C NMR, 101 MHz, CDCl$_3$
f1 (ppm)

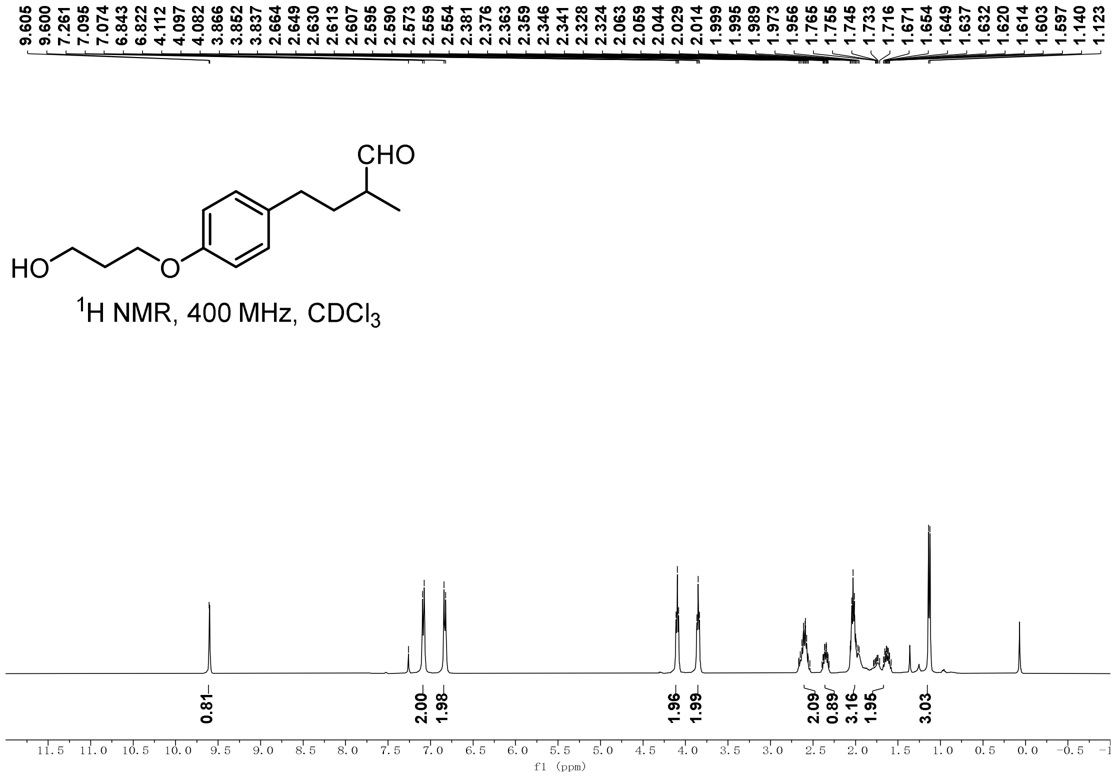
9.605 9.600 7.261 7.096 7.074 6.843 6.822 4.112 4.097 4.082 3.866 3.852 3.837 2.664 2.649 2.630 2.613 2.607 2.596 2.590 2.573 2.559 2.554 2.381 2.376 2.363 2.359 2.346 2.341 2.328 2.324 2.063 2.059 2.044 2.029 2.014 1.999 1.995 1.989 1.973 1.956 1.765 1.755 1.746 1.733 1.716 1.671 1.654 1.649 1.637 1.632 1.620 1.614 1.603 1.597 1.140 1.123
CHO
HO
O
^{1}H NMR, 400 MHz, $CDCl_3$
0.81
2.00
1.98
1.96
1.99
2.09
0.89
3.16
1.95
3.03
f1 (ppm)

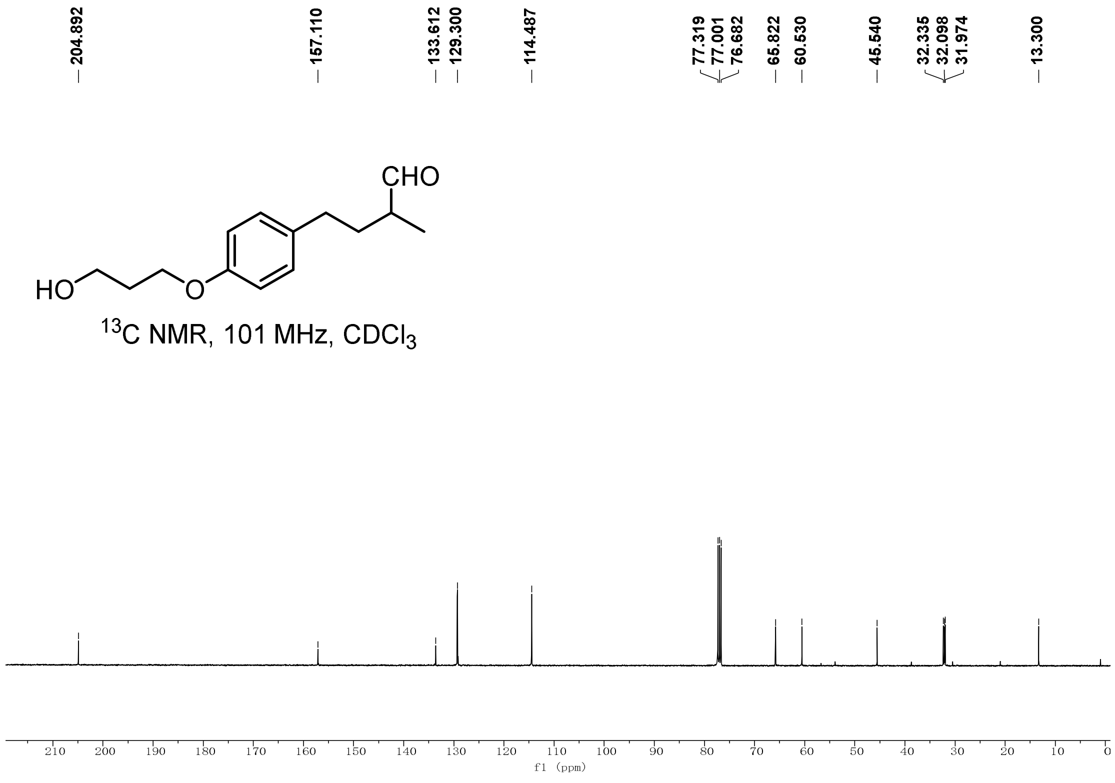
204.892 157.110 133.612 129.300 114.487 77.319 77.001 76.682 65.822 60.530 45.540 32.335 32.098 31.974 13.300
CHO
HO
O
^{13}C NMR, 101 MHz, $CDCl_3$
f1 (ppm)

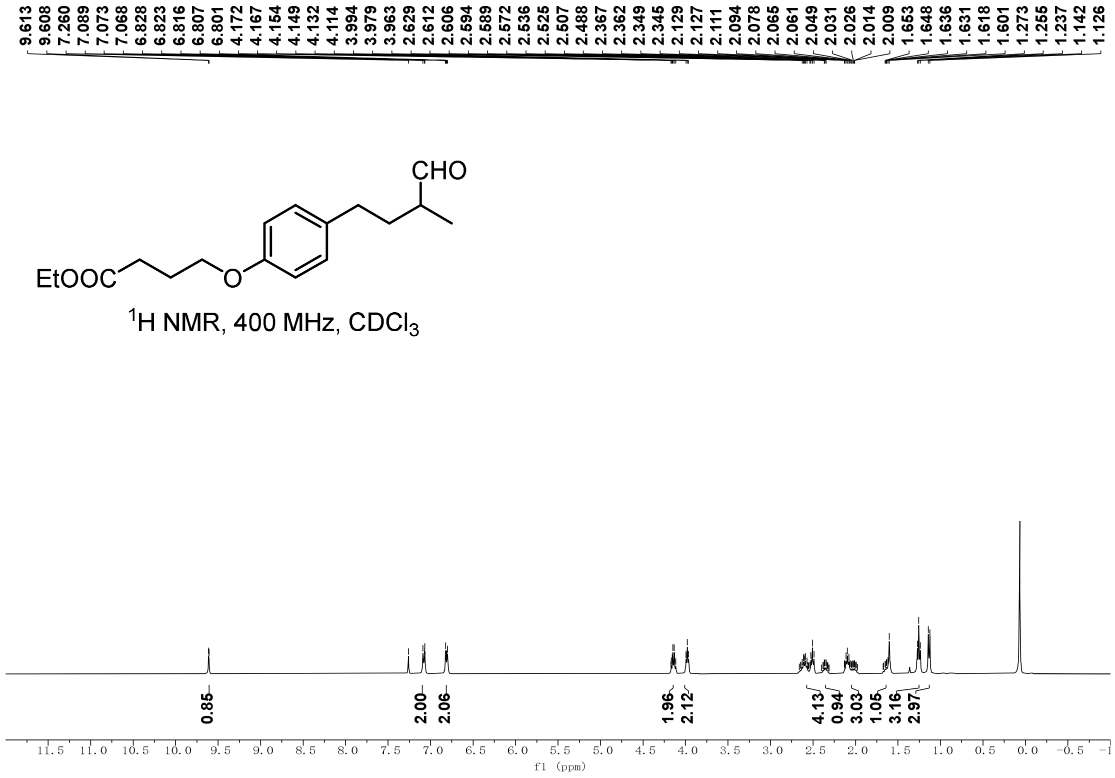

^{1}H NMR, 400 MHz, $CDCl_3$

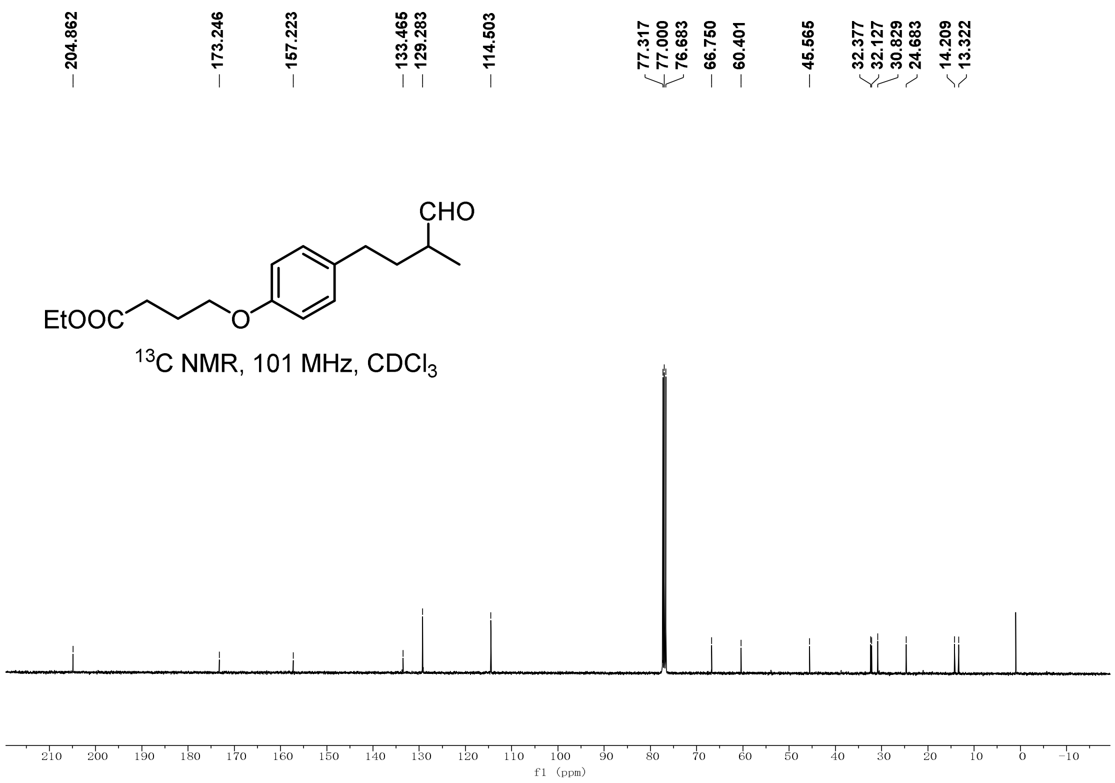

^{13}C NMR, 101 MHz, $CDCl_3$

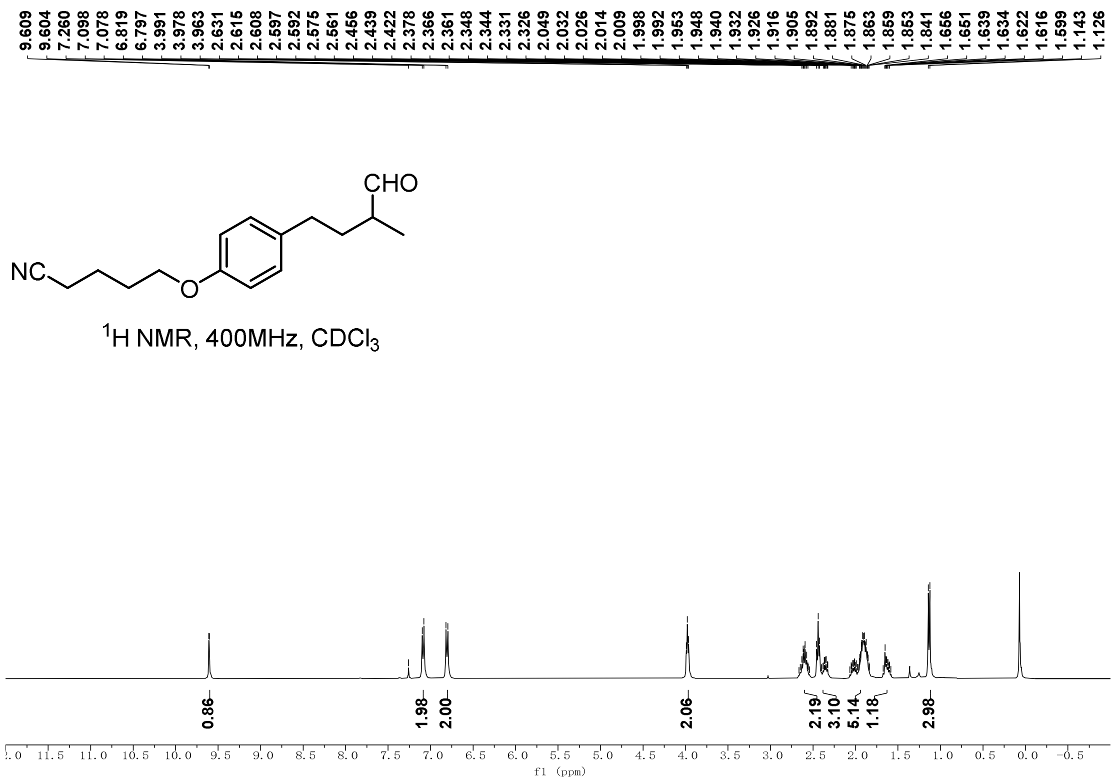

^{1}H NMR, 400MHz, $CDCl_3$

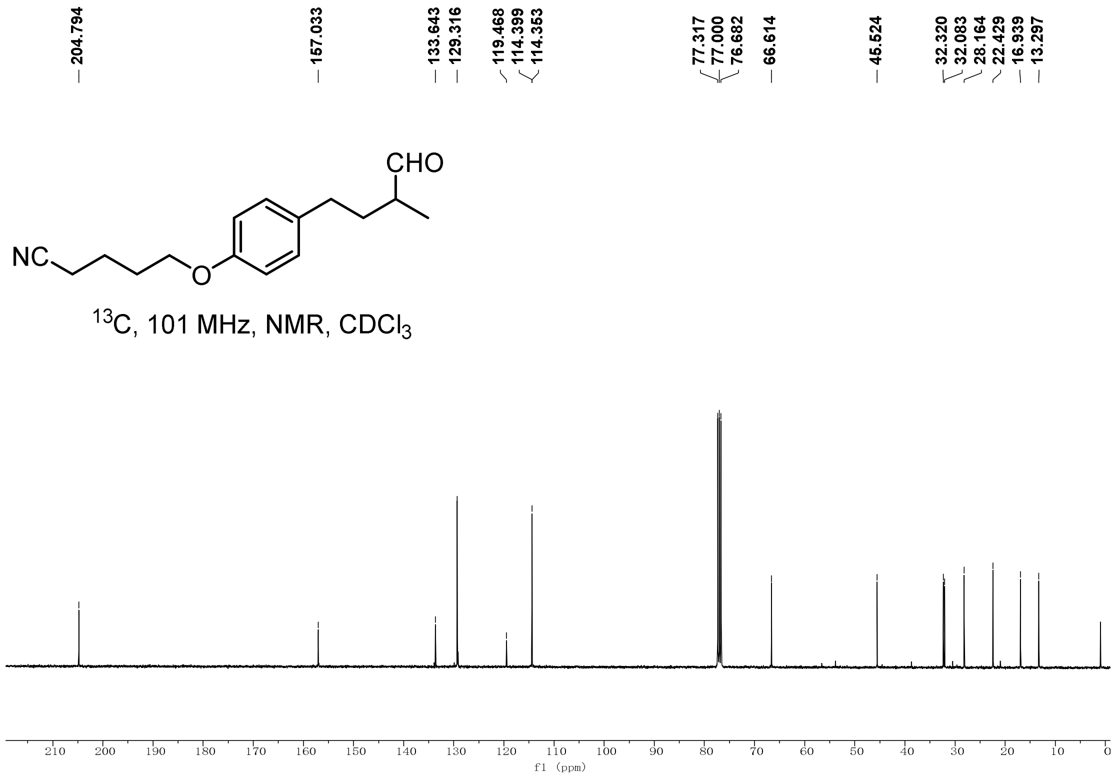

^{13}C, 101 MHz, NMR, $CDCl_3$

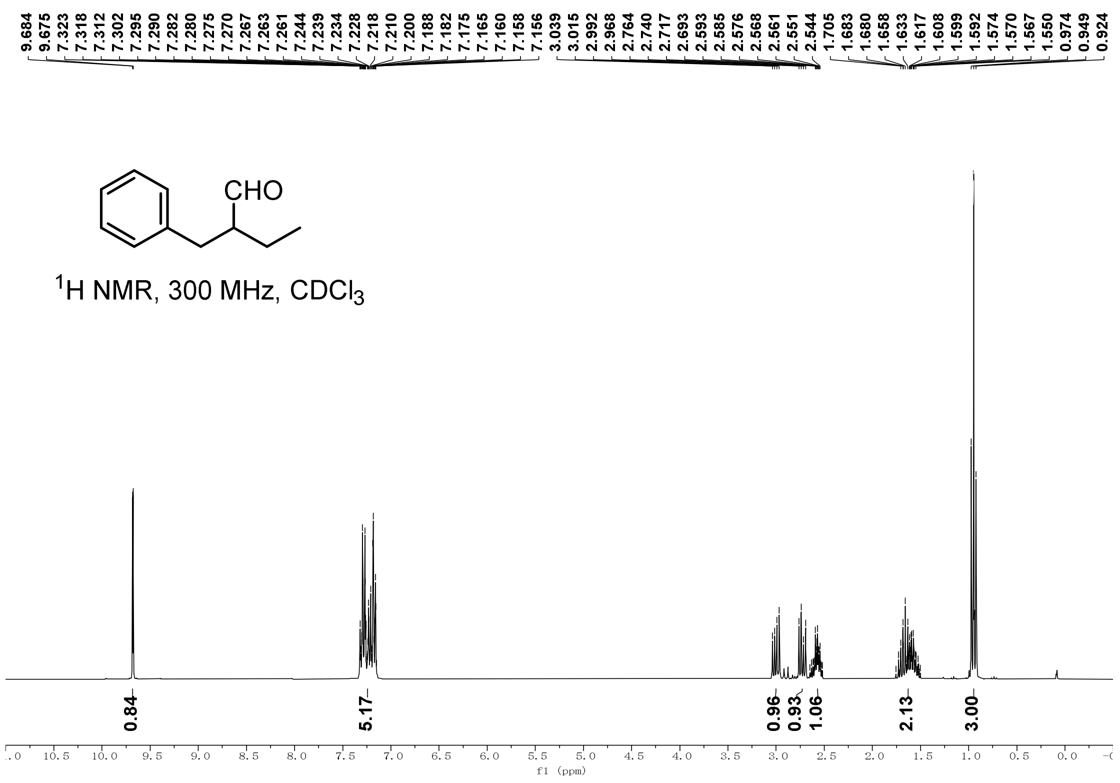

^{1}H NMR, 300 MHz, $CDCl_3$

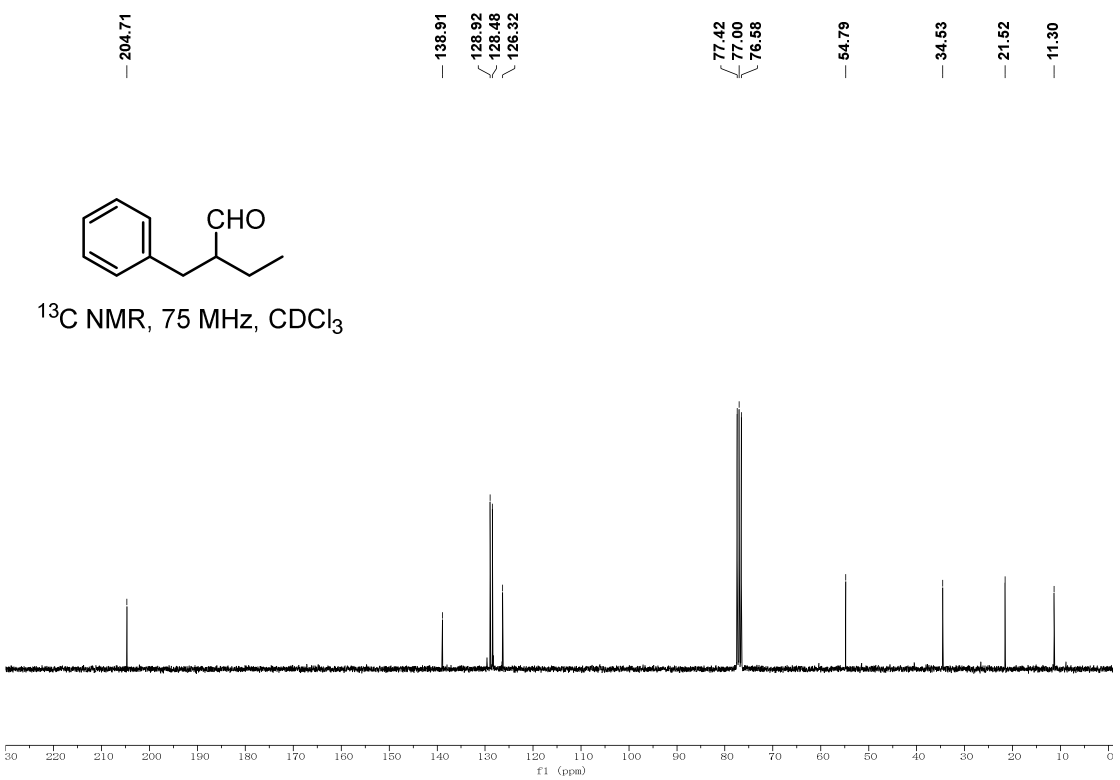

^{13}C NMR, 75 MHz, $CDCl_3$

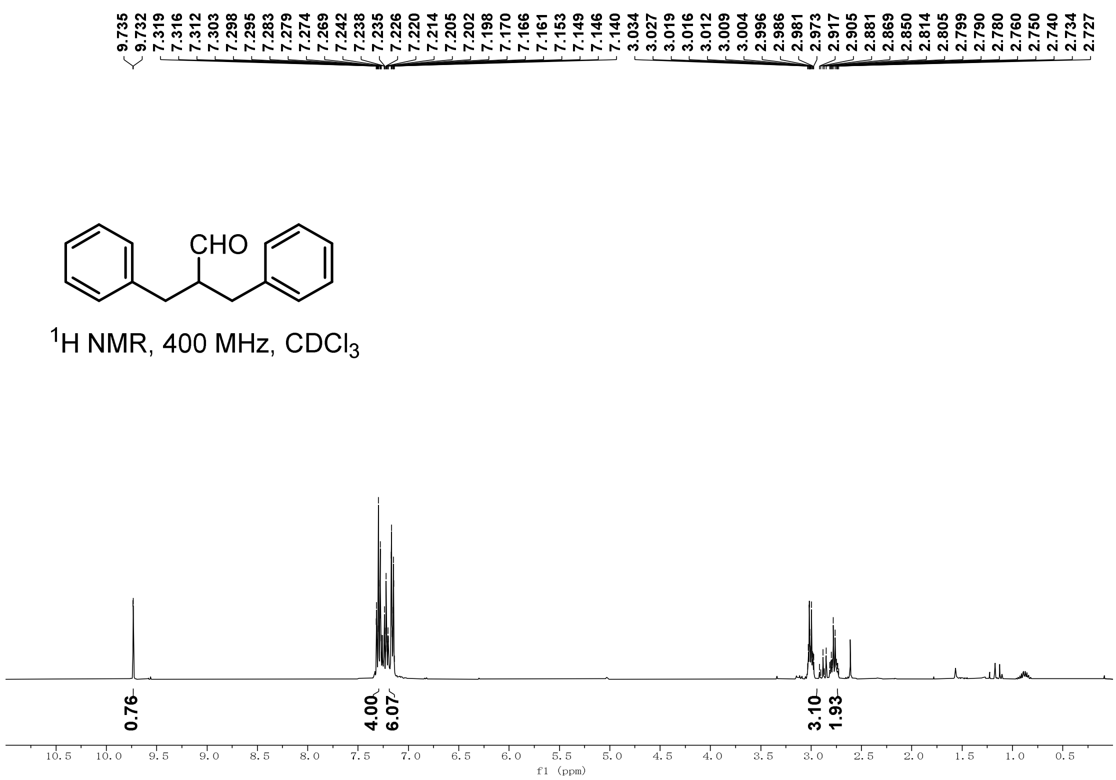
9.735
9.732
7.319
7.316
7.312
7.303
7.298
7.295
7.283
7.279
7.274
7.269
7.242
7.238
7.235
7.226
7.220
7.214
7.205
7.202
7.198
7.170
7.166
7.161
7.153
7.149
7.146
7.140
3.034
3.027
3.019
3.016
3.012
3.009
3.004
2.996
2.986
2.981
2.973
2.917
2.905
2.881
2.869
2.850
2.814
2.805
2.799
2.790
2.780
2.760
2.750
2.740
2.734
2.727
CHO
^{1}H NMR, 400 MHz, CDCl$_3$
0.76
4.00
6.07
3.10
1.93
10.5 10.0 9.5 9.0 8.5 8.0 7.5 7.0 6.5 6.0 5.5 5.0 4.5 4.0 3.5 3.0 2.5 2.0 1.5 1.0 0.5
f1 (ppm)

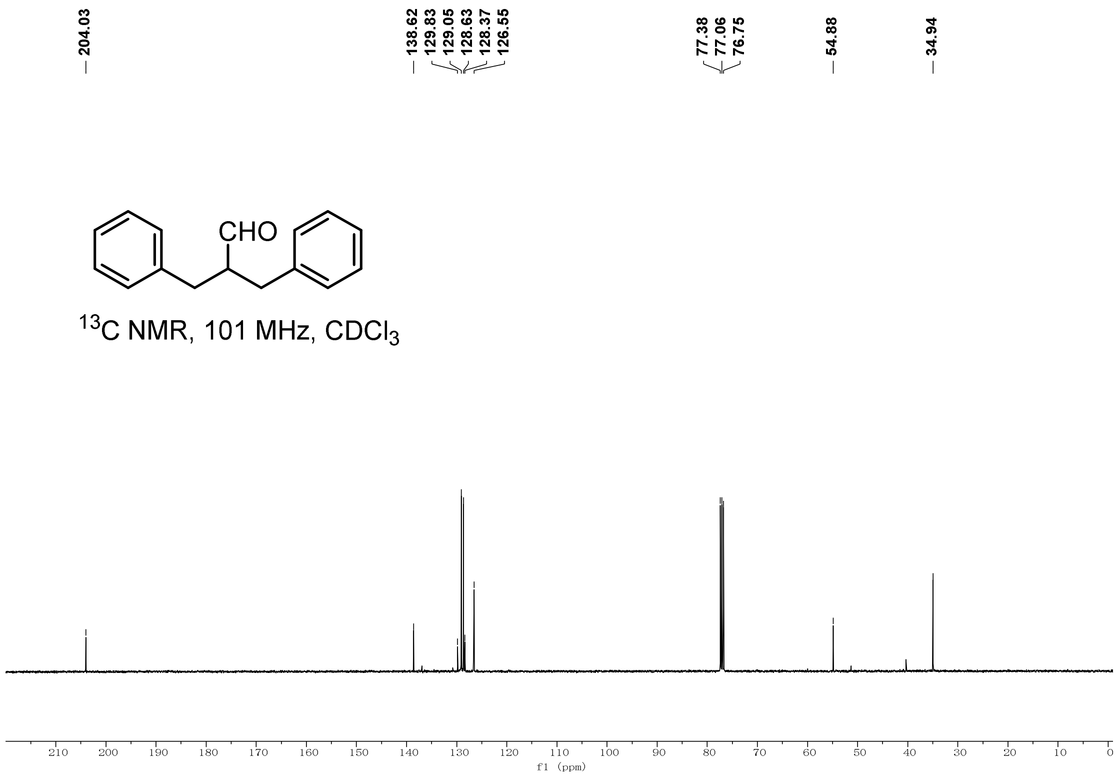
204.03
138.62
129.83
129.05
128.63
128.37
126.55
77.38
77.06
76.75
54.88
34.94
CHO
^{13}C NMR, 101 MHz, CDCl$_3$
210 200 190 180 170 160 150 140 130 120 110 100 90 80 70 60 50 40 30 20 10 0
f1 (ppm)

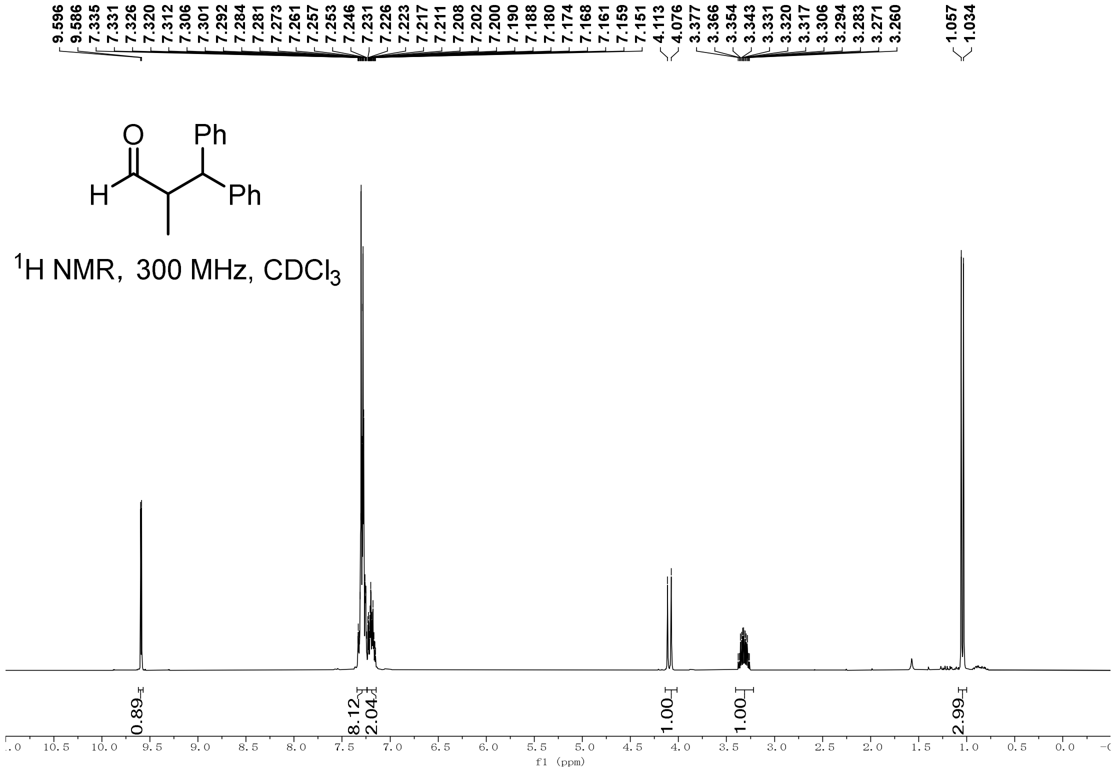
9.596 9.586 7.335 7.331 7.326 7.320 7.312 7.306 7.301 7.292 7.284 7.281 7.273 7.261 7.257 7.253 7.246 7.231 7.226 7.223 7.217 7.211 7.208 7.202 7.200 7.190 7.188 7.180 7.174 7.168 7.161 7.159 7.151 4.113 4.076 3.377 3.366 3.354 3.343 3.331 3.320 3.317 3.306 3.294 3.283 3.271 3.260 1.057 1.034
O
Ph
H
Ph
^{1}H NMR, 300 MHz, CDCl$_3$
0.89
8.12
2.04
1.00
1.00
2.99
f1 (ppm)

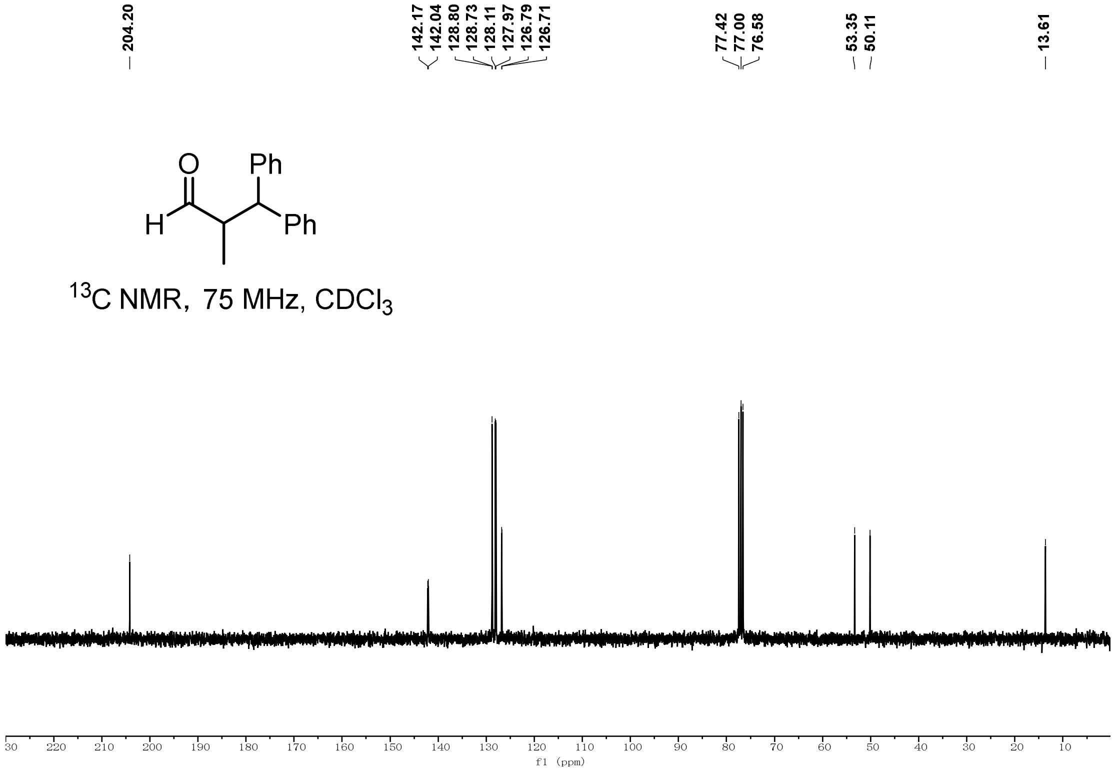
204.20
142.17 142.04 128.80 128.73 128.11 127.97 126.79 126.71
77.42 77.00 76.58
53.35 50.11
13.61
O
Ph
H
Ph
^{13}C NMR, 75 MHz, CDCl$_3$
f1 (ppm)

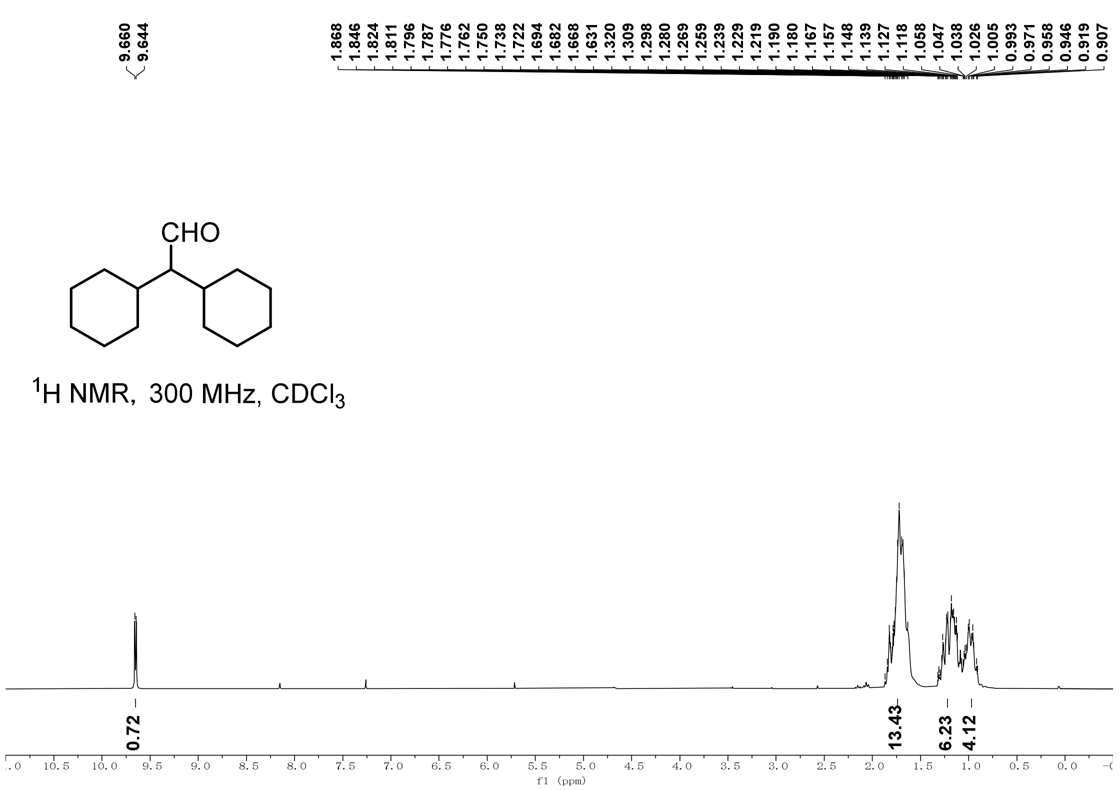
9.660
9.644
1.868
1.846
1.824
1.811
1.796
1.787
1.776
1.762
1.750
1.738
1.722
1.694
1.682
1.668
1.631
1.320
1.309
1.298
1.280
1.269
1.259
1.239
1.229
1.219
1.190
1.180
1.167
1.157
1.148
1.139
1.127
1.118
1.058
1.047
1.038
1.026
1.005
0.993
0.971
0.958
0.946
0.919
0.907
CHO
^{1}H NMR, 300 MHz, $CDCl_3$
0.72
13.43
6.23
4.12
f1 (ppm)

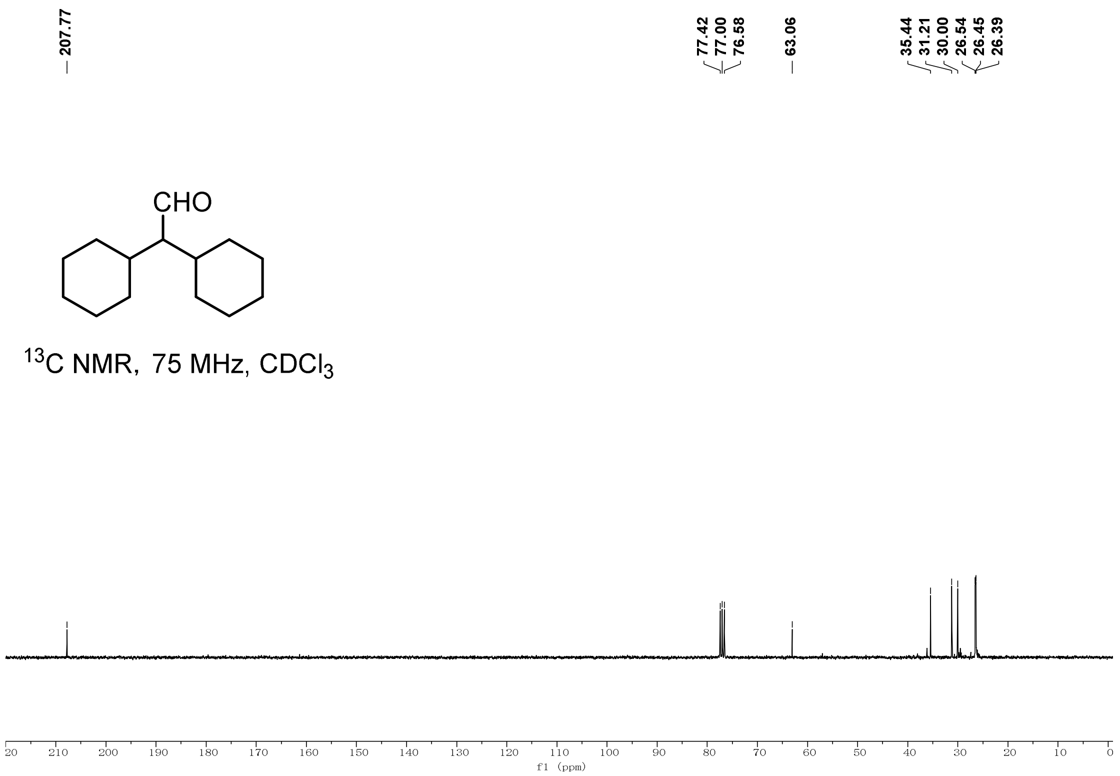
207.77
77.42
77.00
76.58
63.06
35.44
31.21
30.00
26.54
26.45
26.39
CHO
^{13}C NMR, 75 MHz, $CDCl_3$
f1 (ppm)

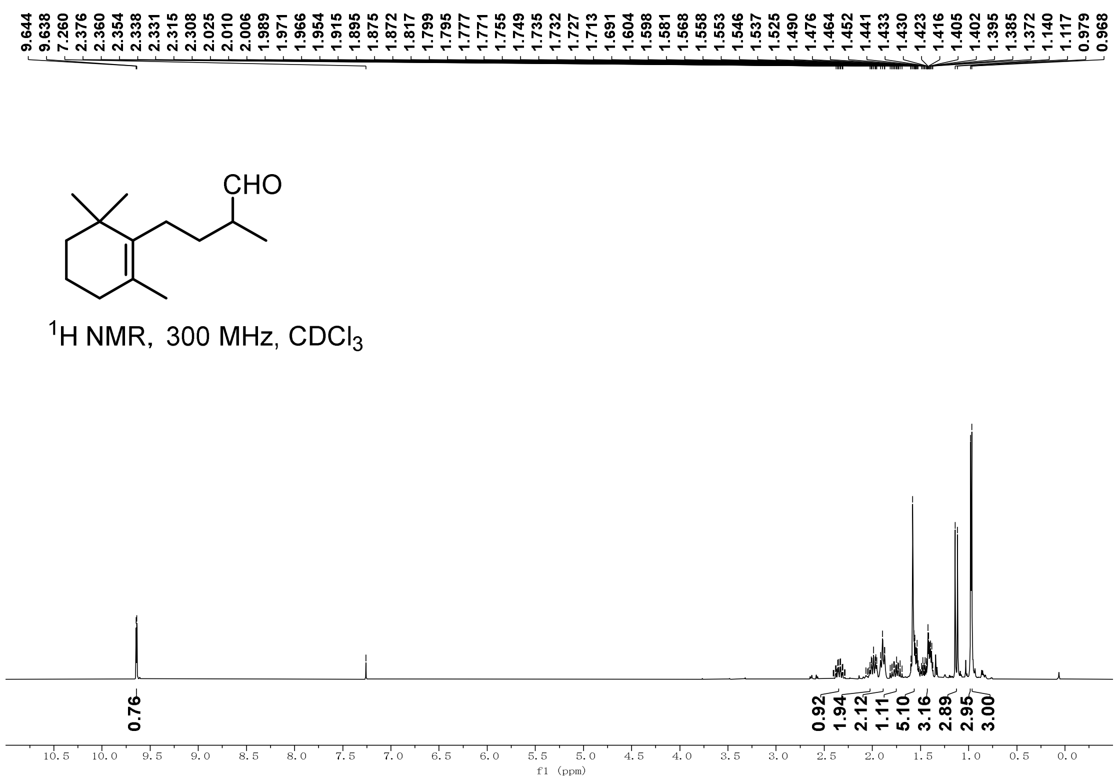
9.644 9.638 7.260 2.376 2.360 2.354 2.338 2.331 2.315 2.308 2.025 2.010 2.006 1.989 1.971 1.966 1.954 1.915 1.895 1.875 1.872 1.817 1.799 1.795 1.777 1.771 1.755 1.749 1.735 1.732 1.727 1.713 1.691 1.604 1.598 1.581 1.568 1.558 1.553 1.546 1.537 1.525 1.490 1.476 1.464 1.452 1.441 1.433 1.430 1.423 1.416 1.405 1.402 1.395 1.385 1.372 1.140 1.117 0.979 0.968
CHO
^{1}H NMR, 300 MHz, $CDCl_3$
0.76
0.92 1.94 2.12 1.11 5.10 3.16 2.89 2.95 3.00
f1 (ppm)

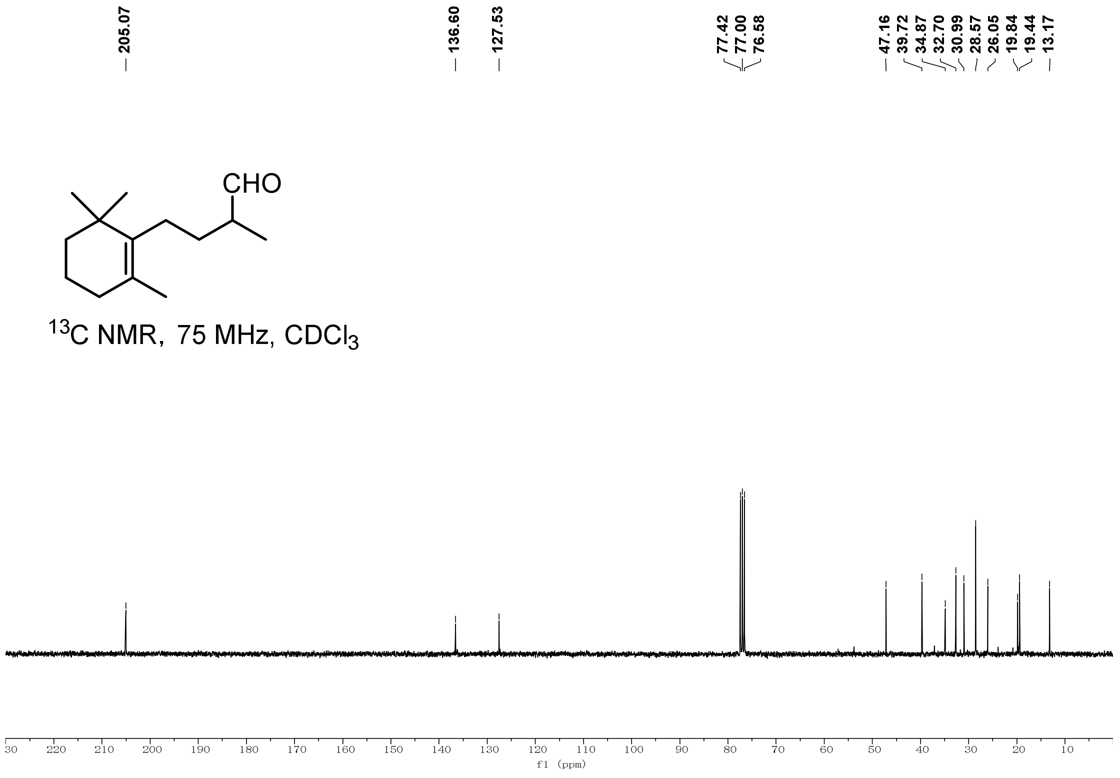
205.07
136.60 127.53
77.42 77.00 76.58
47.16 39.72 34.87 32.70 30.99 28.57 26.05 19.84 19.44 13.17
CHO
^{13}C NMR, 75 MHz, $CDCl_3$
f1 (ppm)

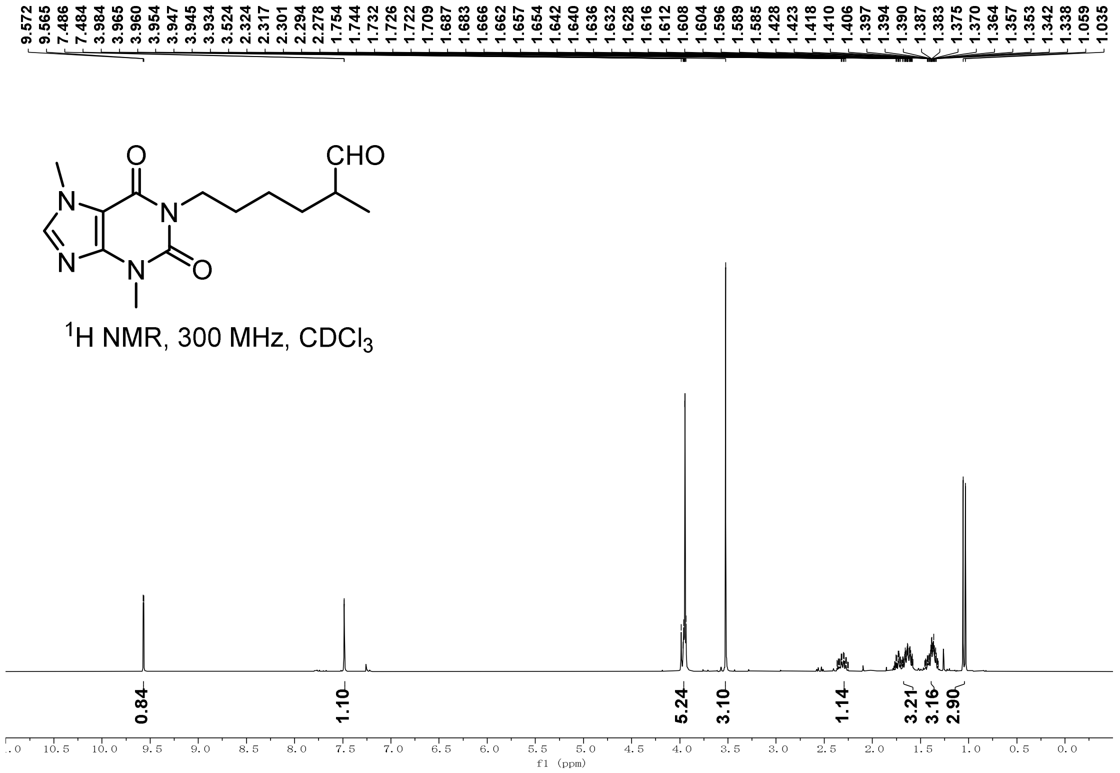
9.572
9.565
7.486
7.484
3.984
3.965
3.960
3.954
3.947
3.945
3.934
3.524
2.324
2.317
2.301
2.294
2.278
1.754
1.744
1.732
1.726
1.722
1.709
1.687
1.683
1.666
1.662
1.657
1.654
1.642
1.640
1.636
1.632
1.628
1.616
1.612
1.608
1.604
1.596
1.589
1.585
1.428
1.423
1.418
1.410
1.406
1.397
1.394
1.390
1.387
1.383
1.375
1.370
1.364
1.357
1.353
1.342
1.338
1.059
1.035
CHO
O
N
N
N
N
O
^{1}H NMR, 300 MHz, $CDCl_3$
0.84
1.10
5.24
3.10
1.14
3.21
3.16
2.90
f1 (ppm)

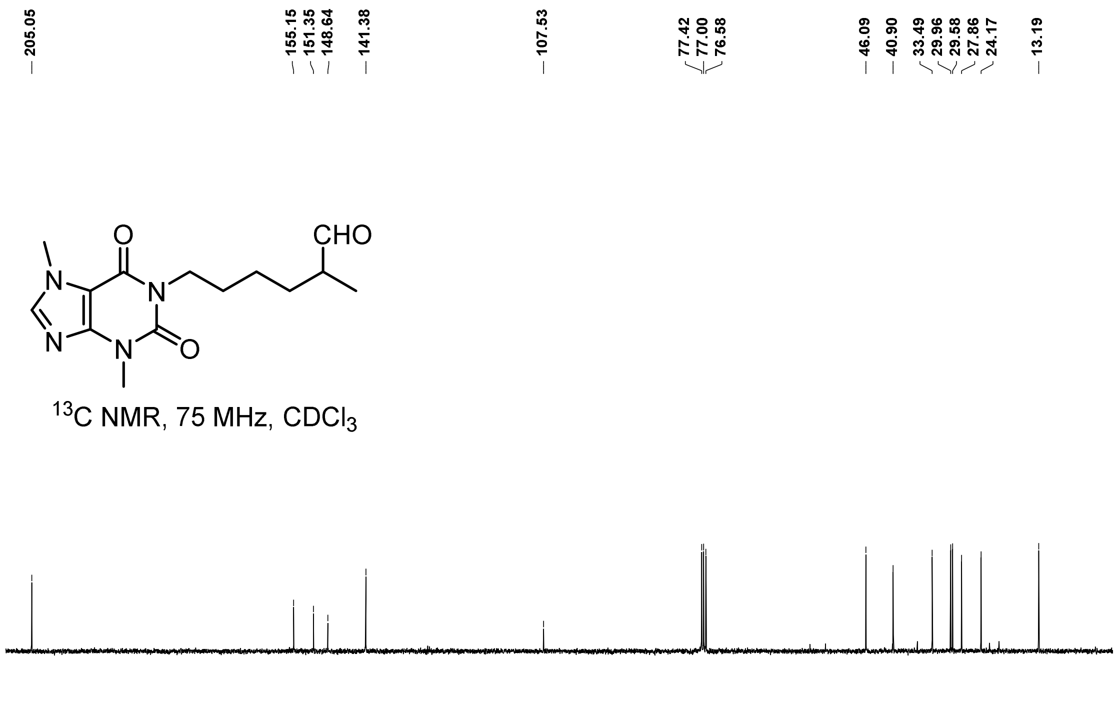
205.05
155.15
151.35
148.64
141.38
107.53
77.42
77.00
76.58
46.09
40.90
33.49
29.96
29.58
27.86
24.17
13.19
CHO
O
N
N
N
N
O
^{13}C NMR, 75 MHz, $CDCl_3$
f1 (ppm)

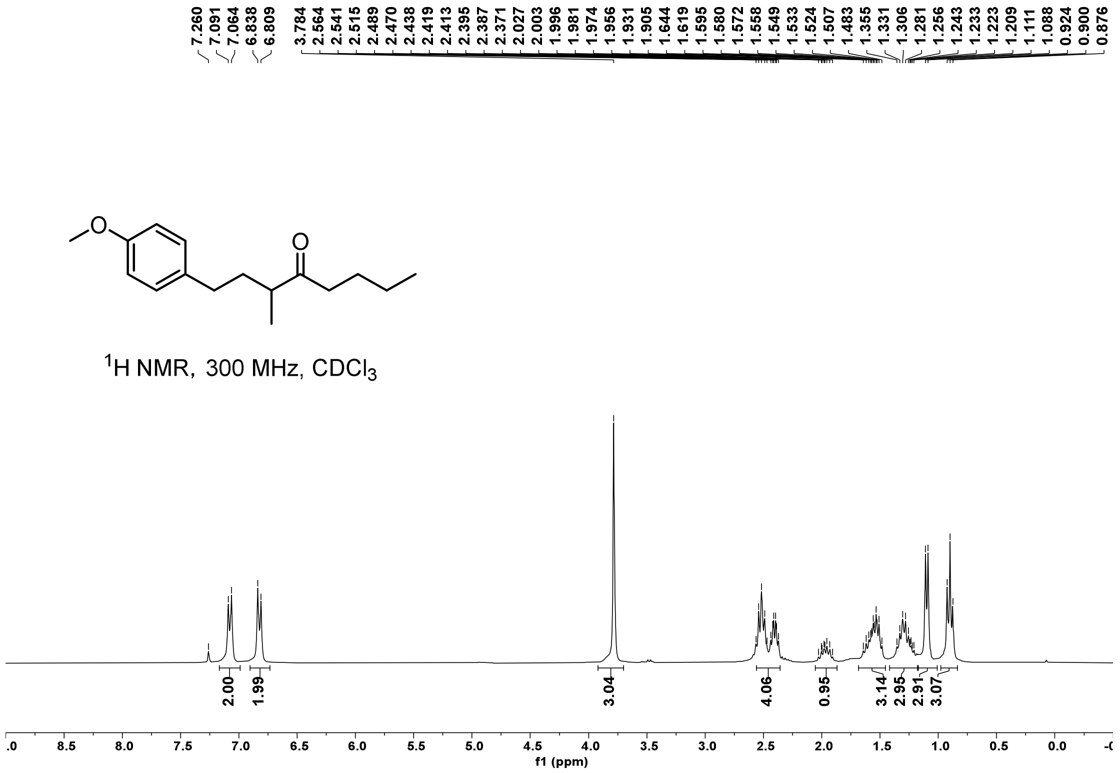
7.260 7.091 7.064 6.838 6.809 3.784 2.564 2.541 2.515 2.489 2.470 2.438 2.419 2.413 2.395 2.387 2.371 2.027 2.003 1.996 1.981 1.974 1.956 1.931 1.905 1.644 1.619 1.595 1.580 1.572 1.558 1.549 1.533 1.524 1.507 1.483 1.355 1.331 1.306 1.281 1.256 1.243 1.233 1.223 1.209 1.111 1.088 0.924 0.900 0.876
O
O
^{1}H NMR, 300 MHz, CDCl$_3$
2.00 1.99 3.04 4.06 0.95 3.14 2.95 2.91 3.07
f1 (ppm)

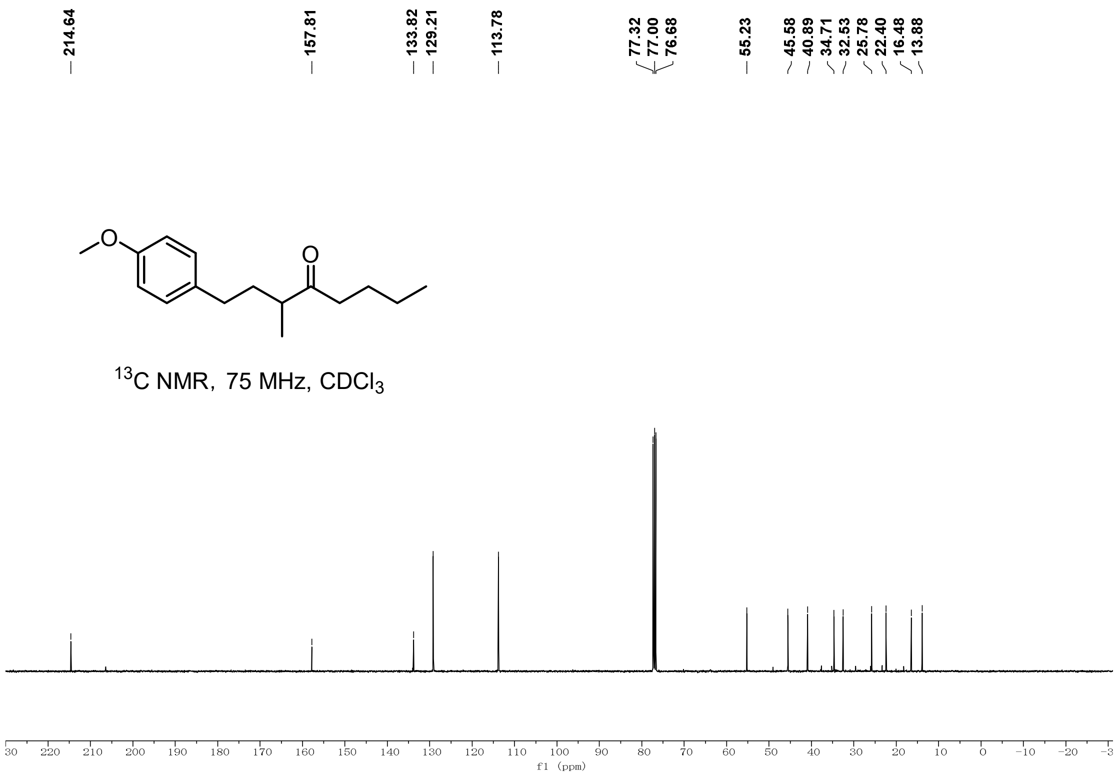
214.64 157.81 133.82 129.21 113.78 77.32 77.00 76.68 55.23 45.58 40.89 34.71 32.53 25.78 22.40 16.48 13.88
O
O
^{13}C NMR, 75 MHz, CDCl$_3$
f1 (ppm)

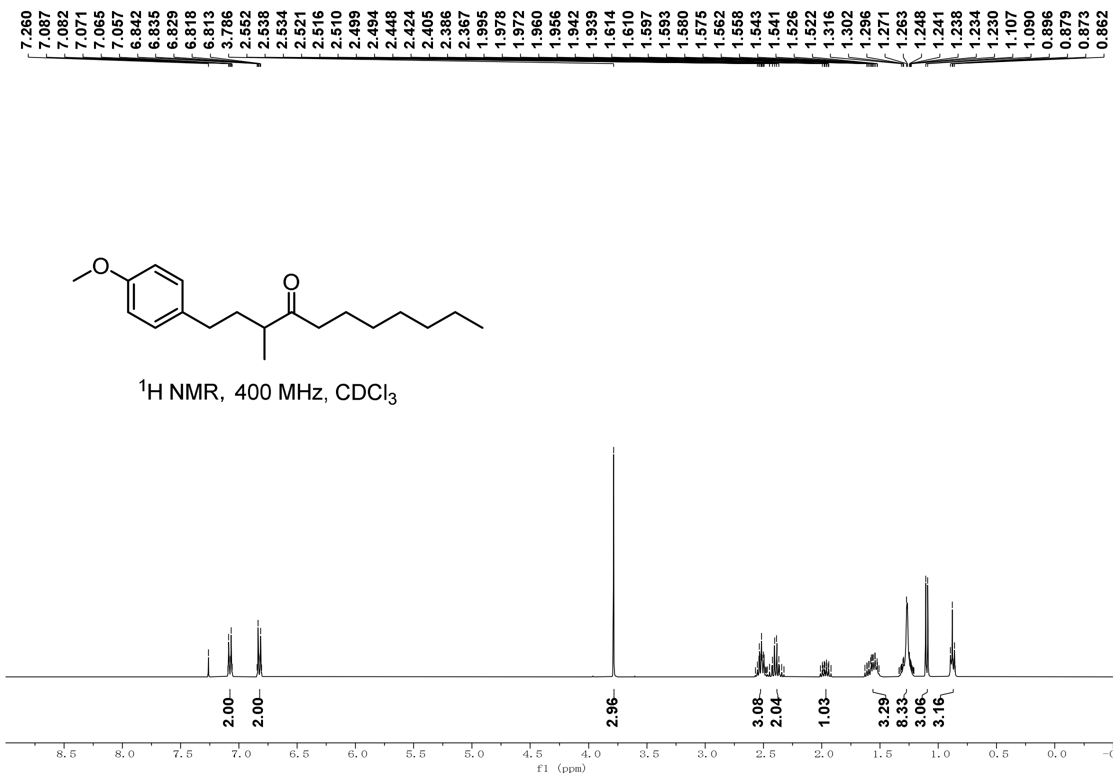

7.260 7.087 7.082 7.071 7.065 7.057 6.842 6.835 6.829 6.818 6.813 3.786 2.552 2.538 2.534 2.521 2.516 2.510 2.499 2.494 2.448 2.424 2.405 2.386 2.367 1.995 1.978 1.972 1.960 1.956 1.942 1.939 1.614 1.610 1.597 1.593 1.580 1.575 1.562 1.558 1.543 1.541 1.526 1.522 1.316 1.302 1.296 1.271 1.263 1.248 1.241 1.238 1.234 1.230 1.107 1.090 0.896 0.879 0.873 0.862
O
^{1}H NMR, 400 MHz, CDCl$_3$
2.00 2.00 2.96 3.08 2.04 1.03 3.29 8.33 3.06 3.16
f1 (ppm)

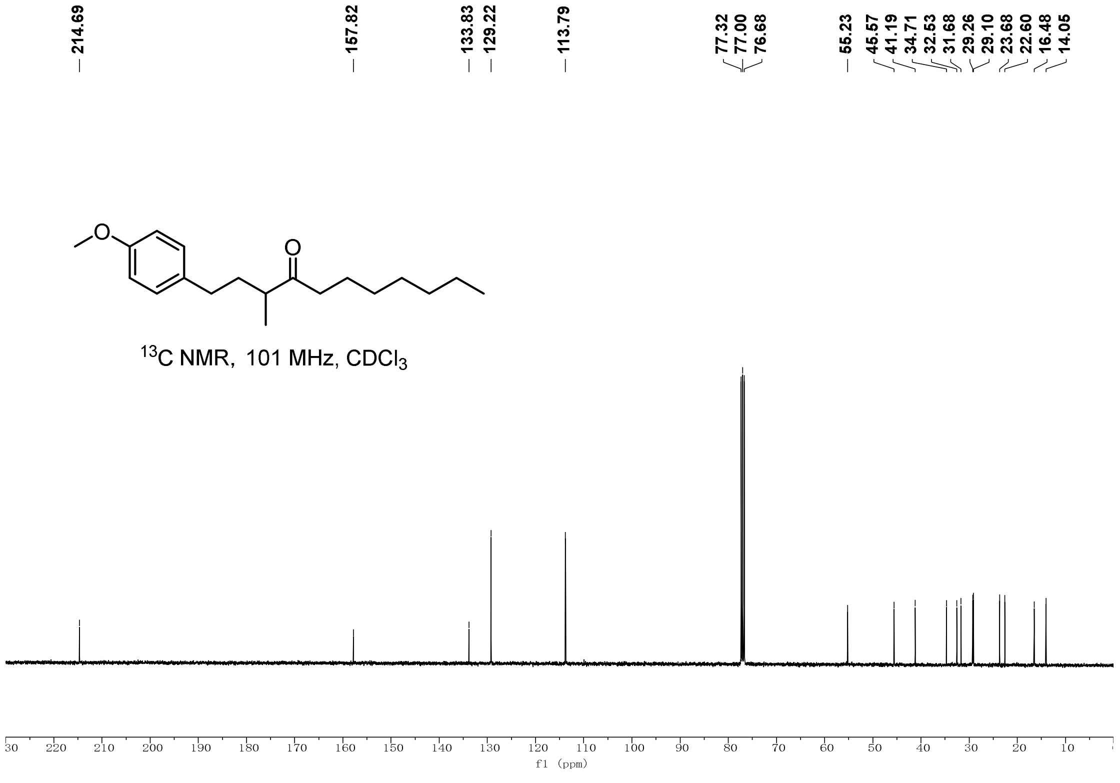

214.69 157.82 133.83 129.22 113.79 77.32 77.00 76.68 55.23 45.57 41.19 34.71 32.53 31.68 29.26 29.10 23.68 22.60 16.48 14.05
O
^{13}C NMR, 101 MHz, CDCl$_3$
f1 (ppm)

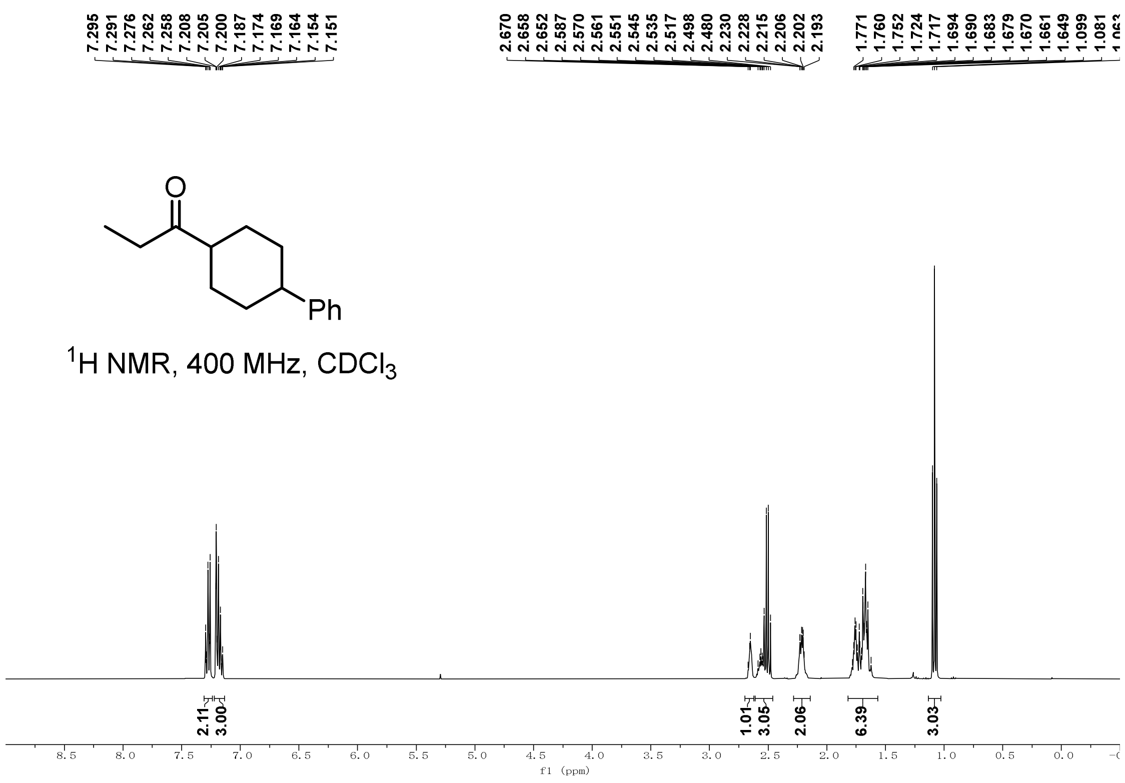
7.295
7.291
7.276
7.262
7.258
7.208
7.205
7.200
7.187
7.174
7.169
7.164
7.154
7.151
2.670
2.658
2.652
2.587
2.570
2.561
2.551
2.545
2.535
2.517
2.498
2.480
2.230
2.228
2.215
2.206
2.202
2.193
1.771
1.760
1.752
1.724
1.717
1.694
1.690
1.683
1.679
1.670
1.661
1.649
1.099
1.081
O
Ph
1H NMR, 400 MHz, CDCl3
2.11
3.00
1.01
3.05
2.06
6.39
3.03
f1 (ppm)

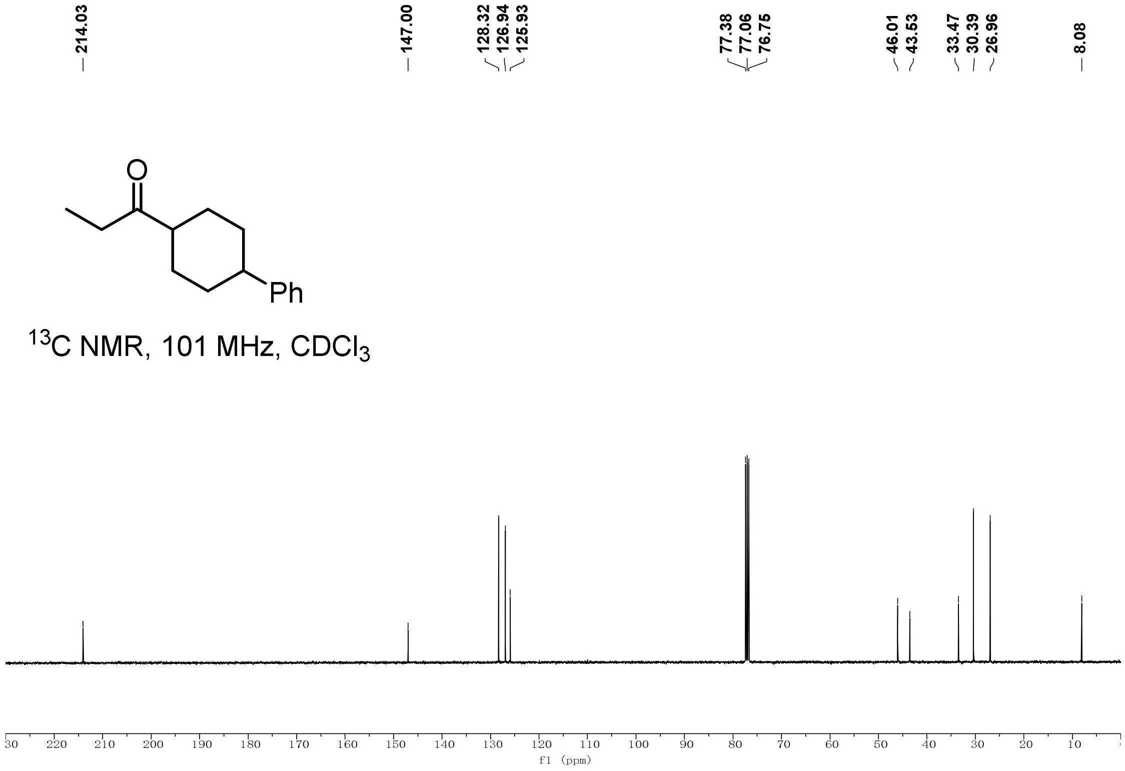
214.03
147.00
128.32
126.94
125.93
77.38
77.06
76.75
46.01
43.53
33.47
30.39
26.96
8.08
O
Ph
13C NMR, 101 MHz, CDCl3
f1 (ppm)

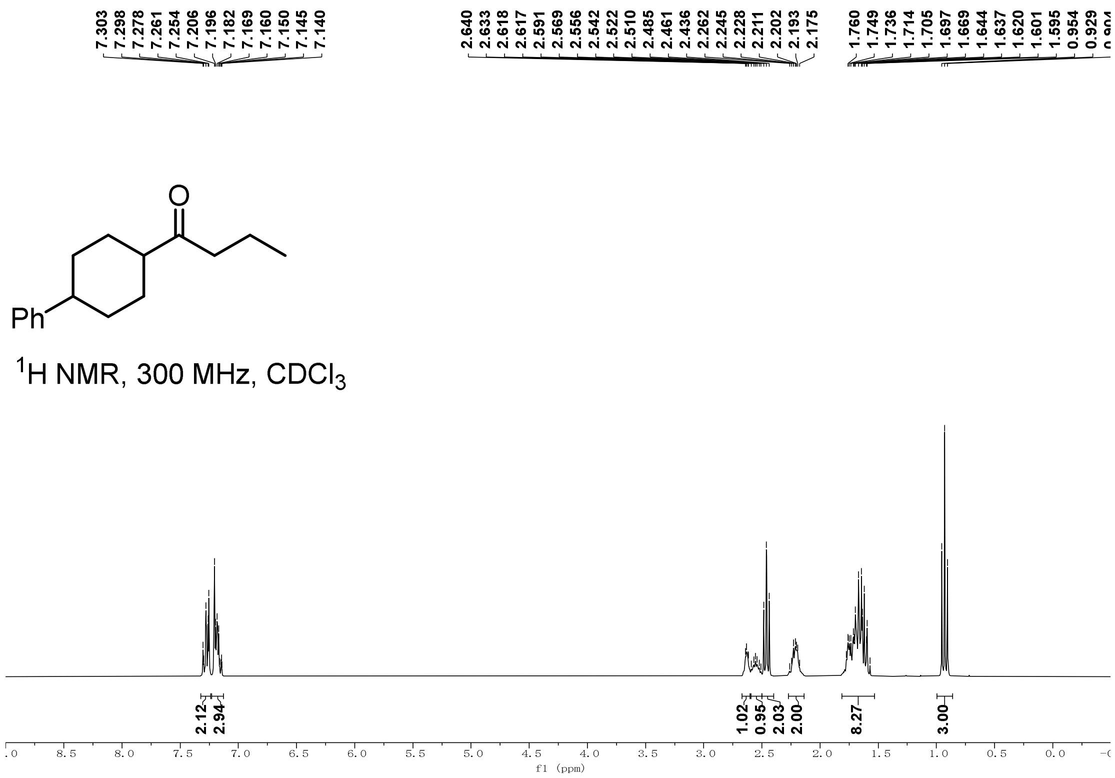
7.303 7.298 7.278 7.261 7.254 7.206 7.196 7.182 7.169 7.160 7.150 7.145 7.140
2.640 2.633 2.618 2.617 2.591 2.569 2.556 2.542 2.522 2.510 2.485 2.461 2.436 2.262 2.245 2.228 2.211 2.202 2.193 2.175
1.760 1.749 1.736 1.714 1.705 1.697 1.669 1.644 1.637 1.620 1.601 1.595 0.954 0.929
O
Ph
^{1}H NMR, 300 MHz, $CDCl_3$
2.12 2.94 1.02 0.95 2.03 2.00 8.27 3.00
f1 (ppm)

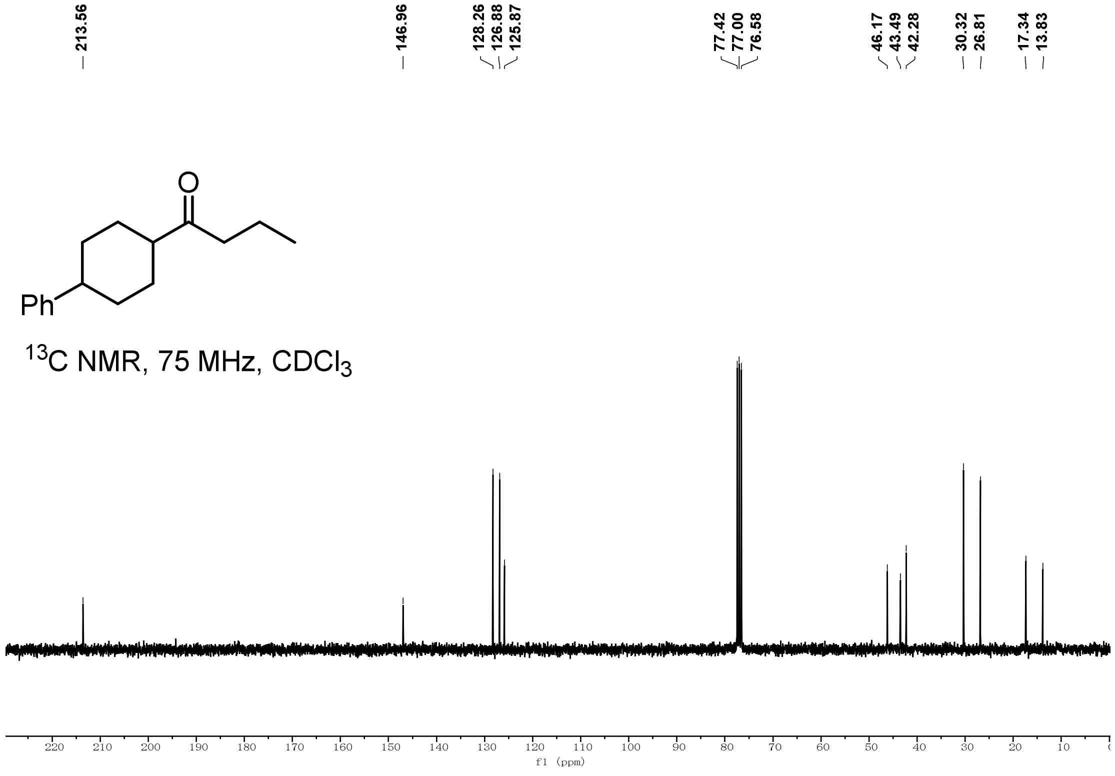
213.56 146.96 128.26 126.88 125.87 77.42 77.00 76.58 46.17 43.49 42.28 30.32 26.81 17.34 13.83
O
Ph
^{13}C NMR, 75 MHz, $CDCl_3$
f1 (ppm)

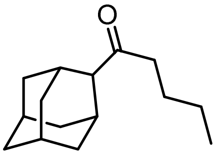

^{1}H NMR, 300 MHz, $CDCl_3$

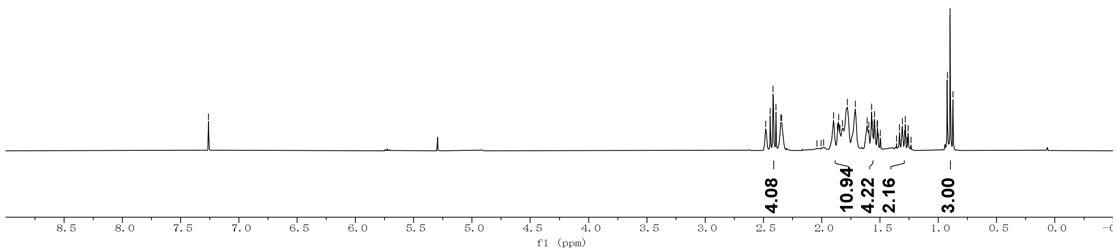

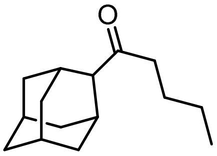

^{13}C NMR, 75 MHz, $CDCl_3$

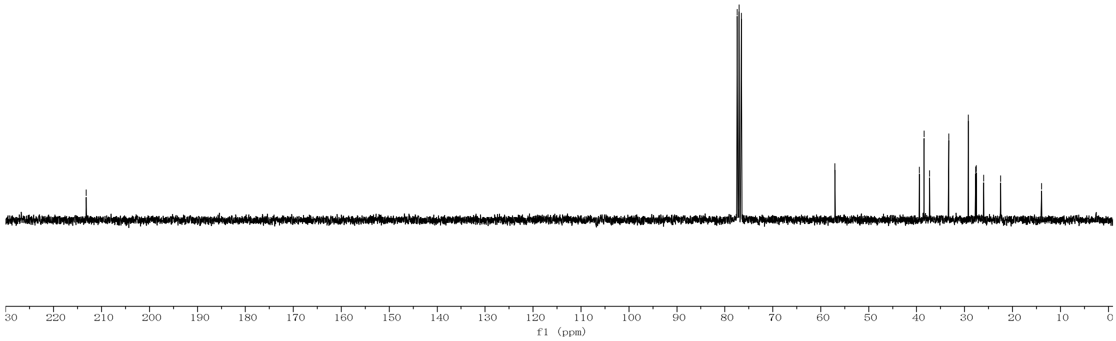

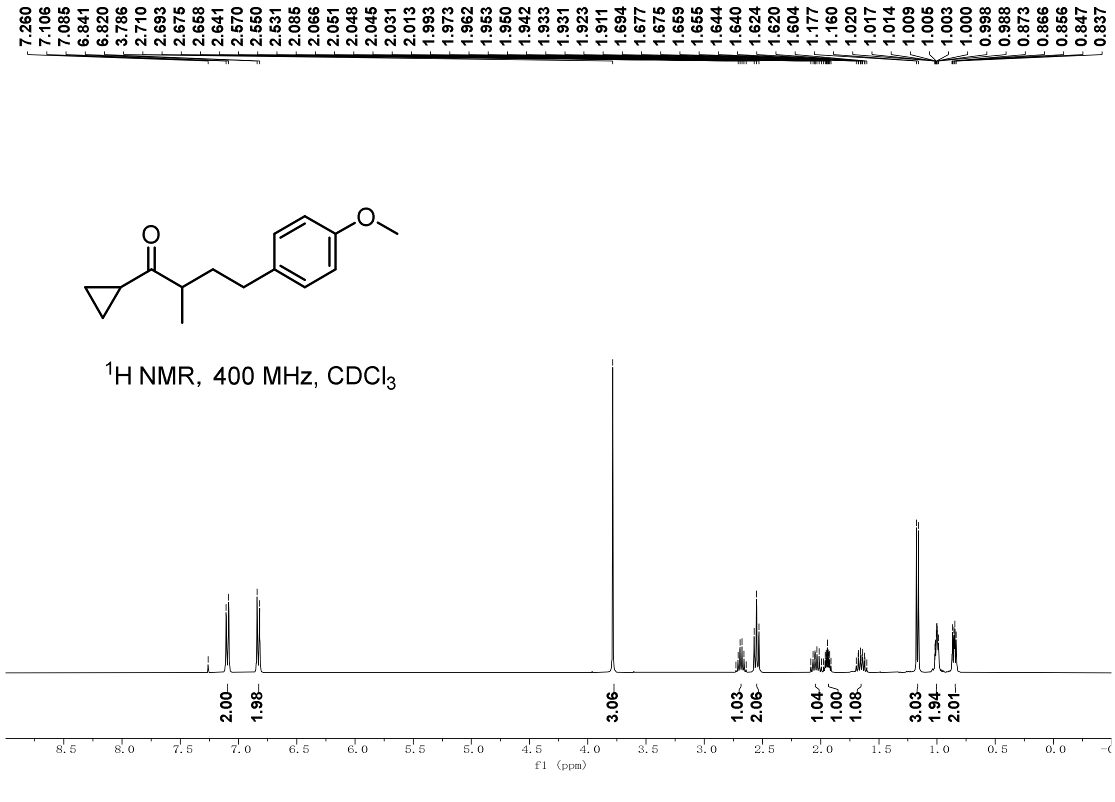
7.260
7.106
7.085
6.841
6.820
3.786
2.710
2.693
2.675
2.658
2.641
2.570
2.550
2.531
2.085
2.066
2.051
2.048
2.045
2.031
2.013
1.993
1.973
1.962
1.953
1.950
1.942
1.933
1.931
1.923
1.911
1.694
1.677
1.675
1.659
1.655
1.644
1.640
1.624
1.620
1.604
1.177
1.160
1.020
1.017
1.014
1.009
1.005
1.003
1.000
0.998
0.988
0.873
0.866
0.856
0.847
0.837
O
O
1H NMR, 400 MHz, CDCl3
2.00
1.98
3.06
1.03
2.06
1.04
1.00
1.08
3.03
1.94
2.01
f1 (ppm)

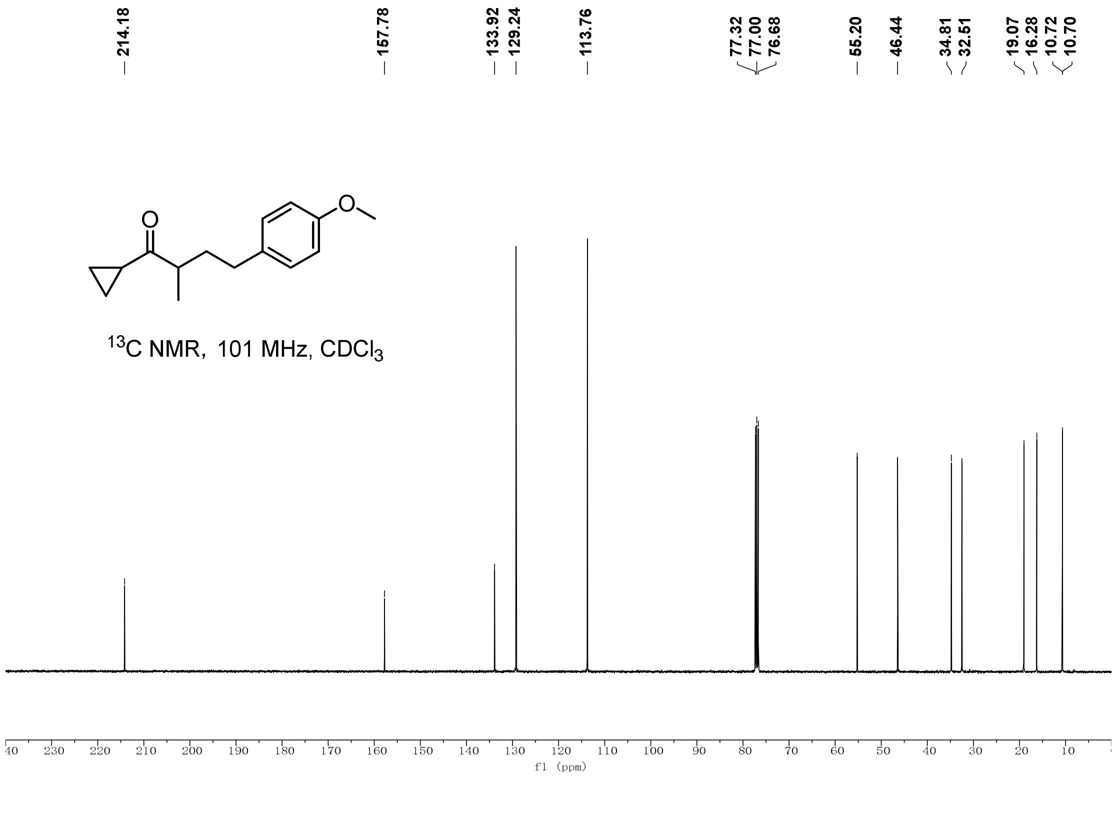
214.18
157.78
133.92
129.24
113.76
77.32
77.00
76.68
55.20
46.44
34.81
32.51
19.07
16.28
10.72
10.70
O
O
13C NMR, 101 MHz, CDCl3
f1 (ppm)

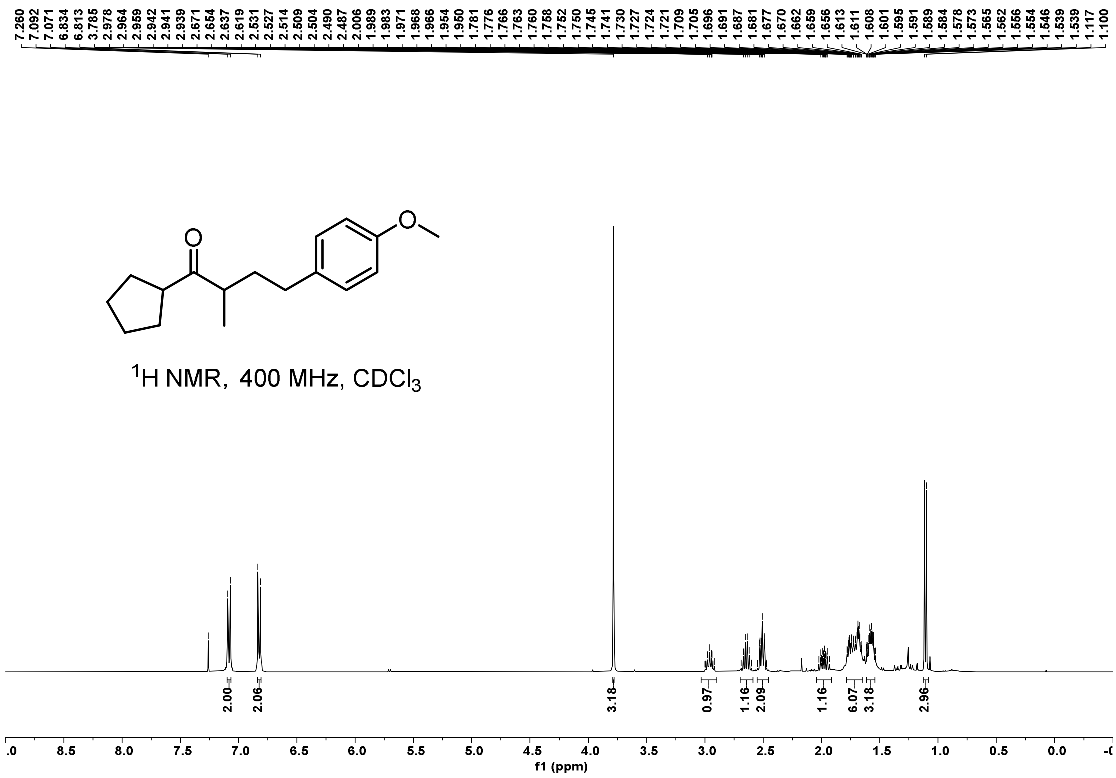
7.260 7.092 7.071 6.834 6.813 3.785 2.978 2.964 2.959 2.942 2.941 2.939 2.671 2.654 2.637 2.619 2.531 2.527 2.514 2.509 2.504 2.490 2.487 2.006 1.989 1.983 1.971 1.968 1.966 1.954 1.950 1.781 1.776 1.766 1.763 1.760 1.758 1.752 1.750 1.745 1.741 1.730 1.727 1.724 1.721 1.709 1.705 1.696 1.691 1.687 1.681 1.677 1.670 1.662 1.659 1.656 1.613 1.611 1.608 1.601 1.595 1.591 1.589 1.584 1.578 1.573 1.565 1.562 1.556 1.554 1.546 1.539 1.117 1.100
O
O
^{1}H NMR, 400 MHz, CDCl$_3$
2.00 2.06 3.18 0.97 1.16 2.09 1.16 6.07 3.18 2.96
f1 (ppm)

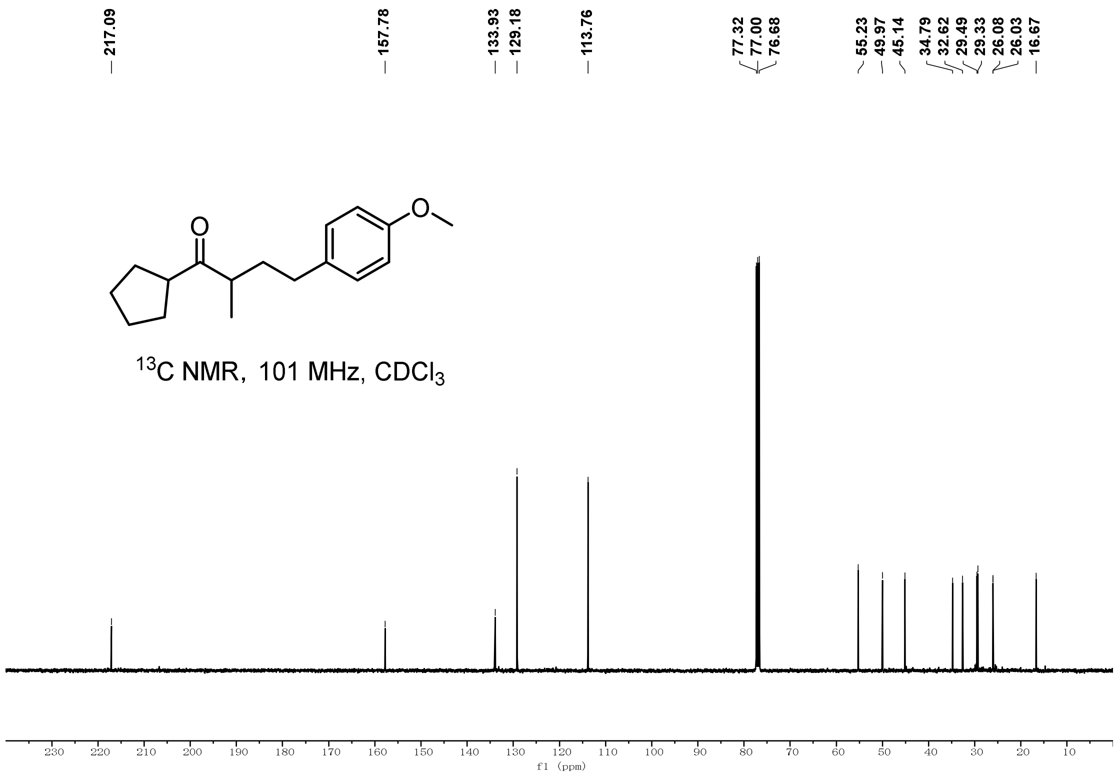
217.09 157.78 133.93 129.18 113.76 77.32 77.00 76.68 55.23 49.97 45.14 34.79 32.62 29.49 29.33 26.08 26.03 16.67
O
O
^{13}C NMR, 101 MHz, CDCl$_3$
f1 (ppm)

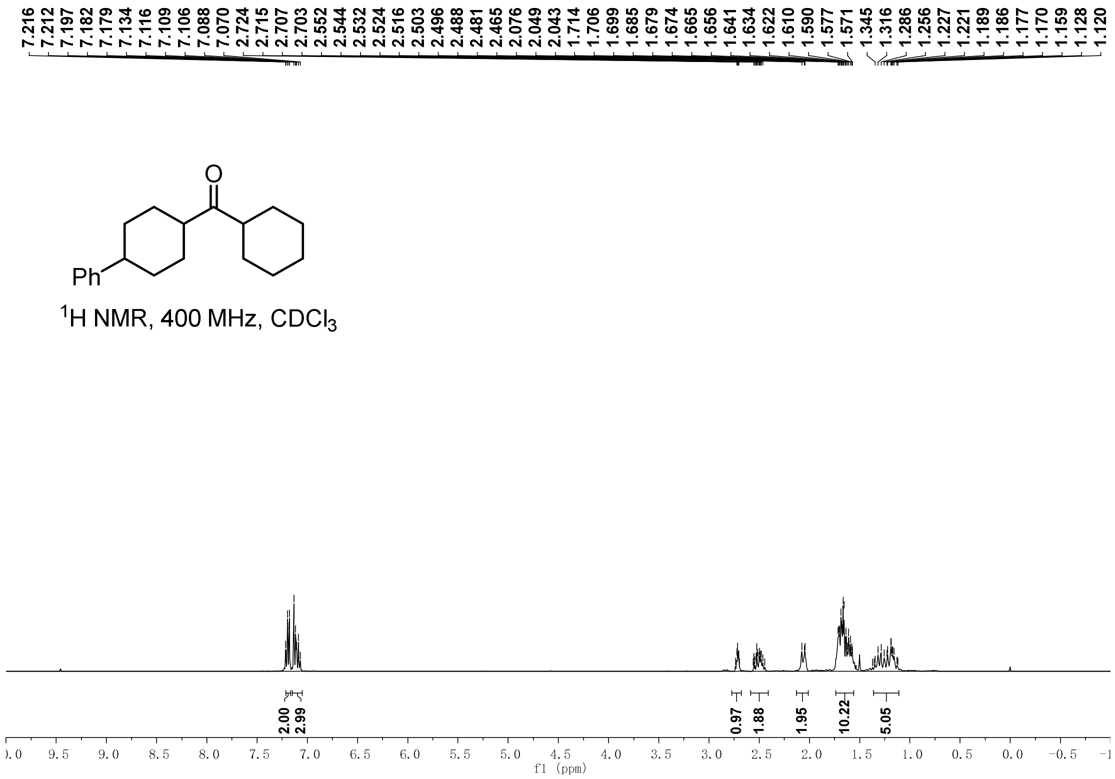

^{1}H NMR, 400 MHz, $CDCl_3$

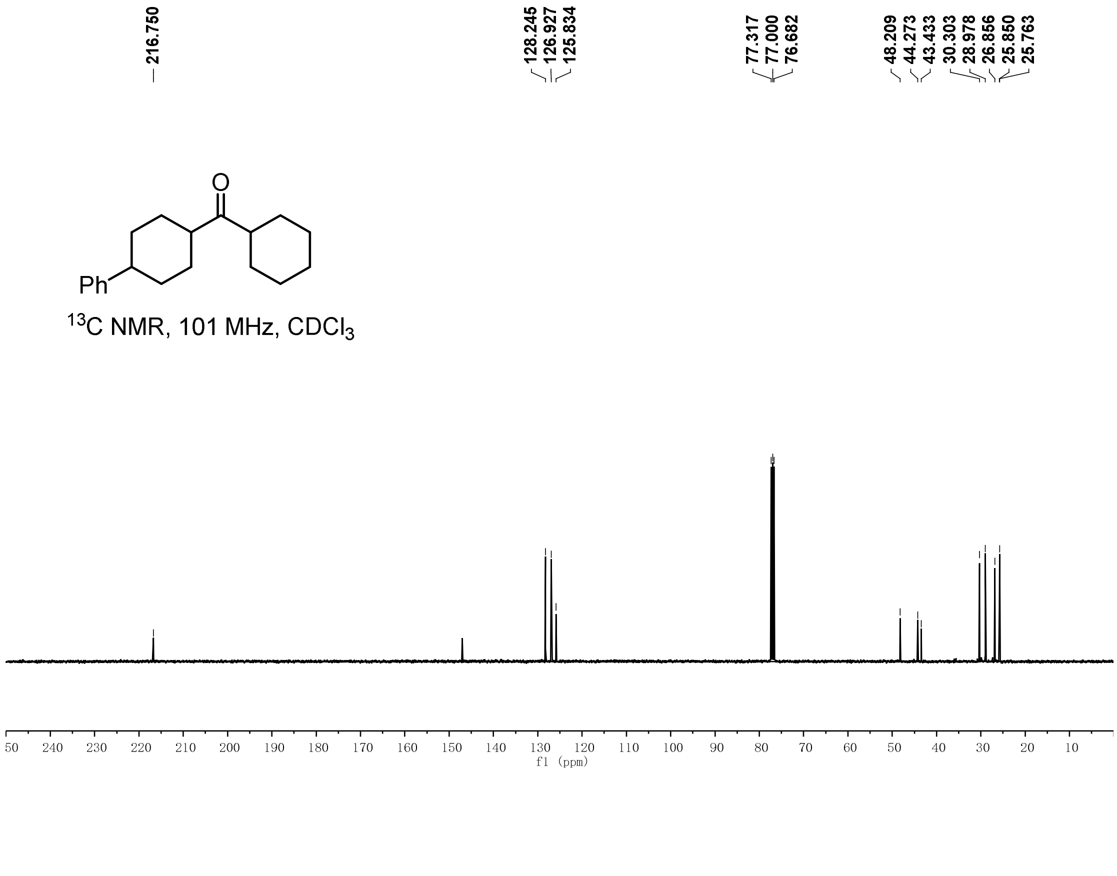

^{13}C NMR, 101 MHz, $CDCl_3$

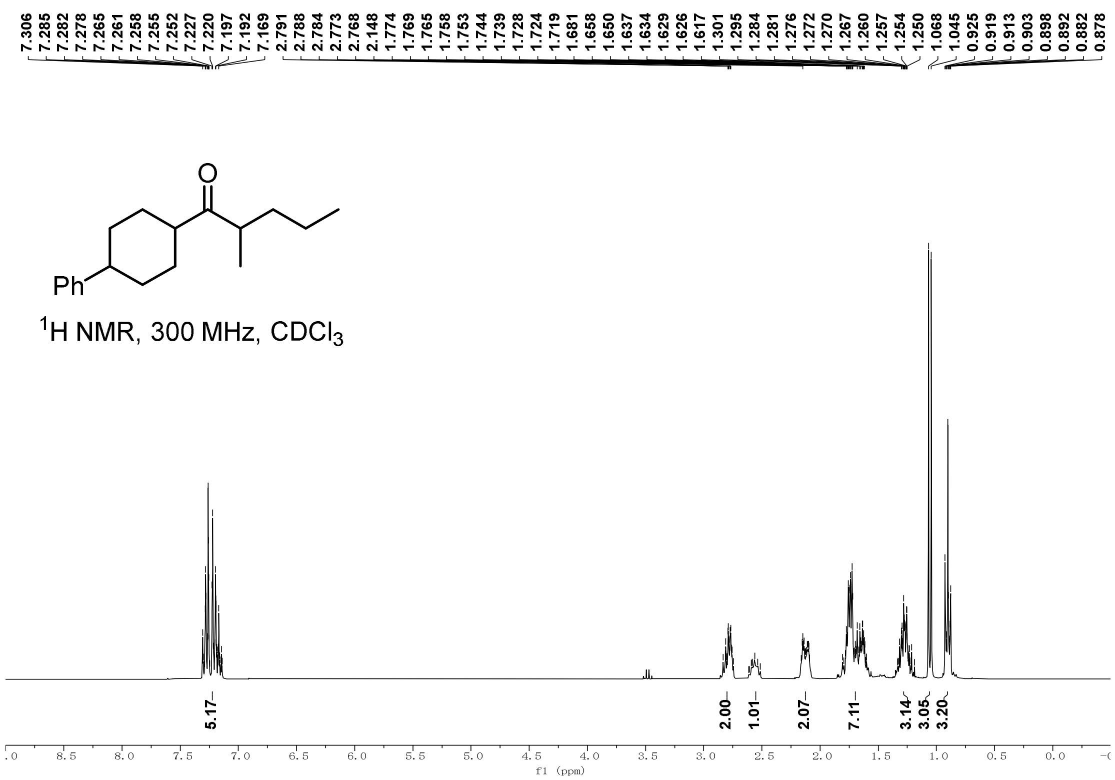
7.306 7.285 7.282 7.278 7.265 7.261 7.258 7.255 7.252 7.227 7.220 7.197 7.192 7.169 2.791 2.788 2.784 2.773 2.768 2.148 1.774 1.769 1.765 1.758 1.753 1.744 1.739 1.728 1.724 1.719 1.681 1.658 1.650 1.637 1.634 1.629 1.626 1.617 1.301 1.295 1.284 1.281 1.276 1.272 1.270 1.267 1.260 1.257 1.254 1.250 1.068 1.045 0.925 0.919 0.913 0.903 0.898 0.892 0.882 0.878
O
Ph
^{1}H NMR, 300 MHz, CDCl$_3$
5.17
2.00
1.01
2.07
7.11
3.14
3.05
3.20
f1 (ppm)

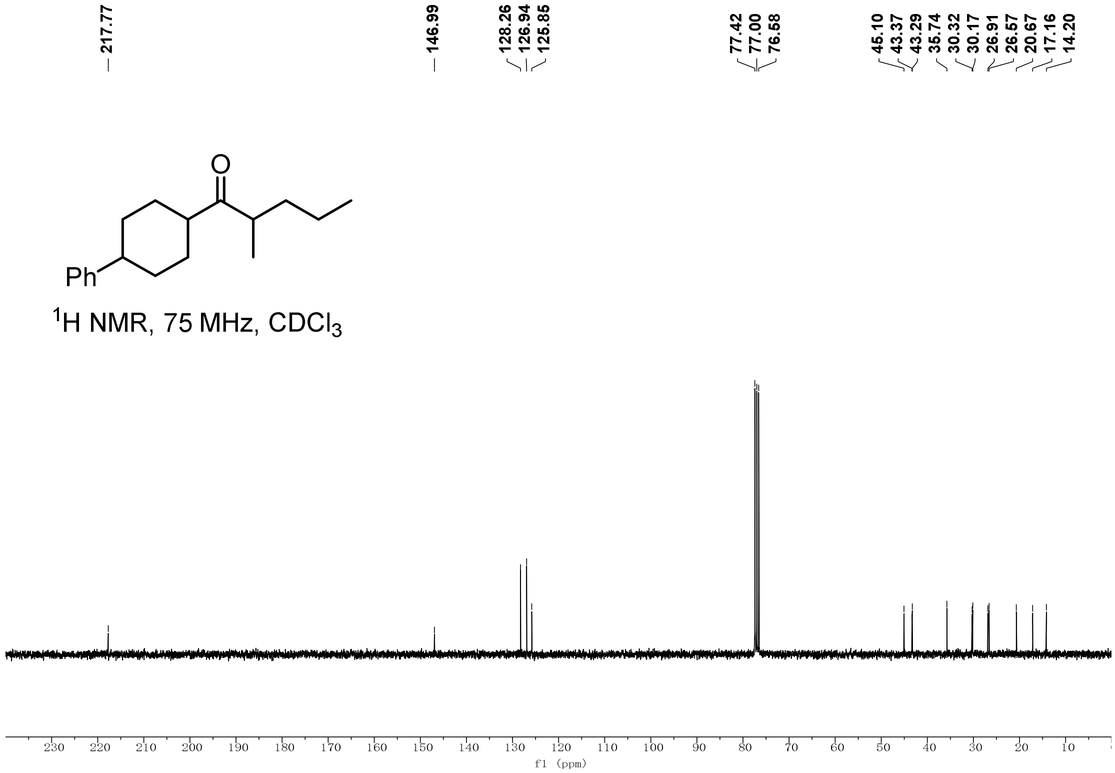
217.77
146.99
128.26 126.94 125.85
77.42 77.00 76.58
45.10 43.37 43.29 35.74 30.32 30.17 26.91 26.57 20.67 17.16 14.20
O
Ph
^{1}H NMR, 75 MHz, CDCl$_3$
f1 (ppm)

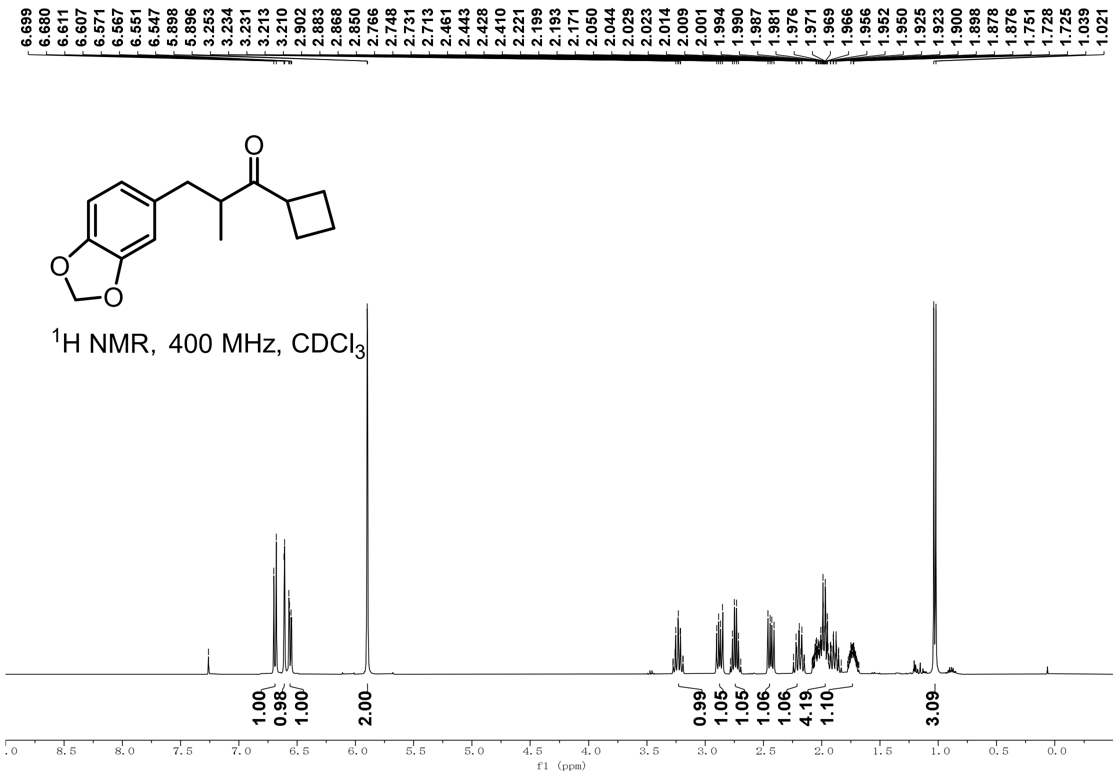
6.699 6.680 6.611 6.607 6.571 6.567 6.551 6.547 5.898 5.896 3.253 3.234 3.231 3.213 3.210 2.902 2.883 2.868 2.850 2.766 2.748 2.731 2.713 2.461 2.443 2.428 2.410 2.221 2.199 2.193 2.171 2.050 2.044 2.029 2.023 2.014 2.009 2.001 1.994 1.990 1.987 1.981 1.976 1.971 1.969 1.966 1.956 1.952 1.950 1.925 1.923 1.900 1.898 1.876 1.751 1.728 1.725 1.039 1.021
^{1}H NMR, 400 MHz, CDCl$_3$
1.00 0.98 1.00 2.00 0.99 1.05 1.05 1.06 1.06 4.19 1.10 3.09
f1 (ppm)

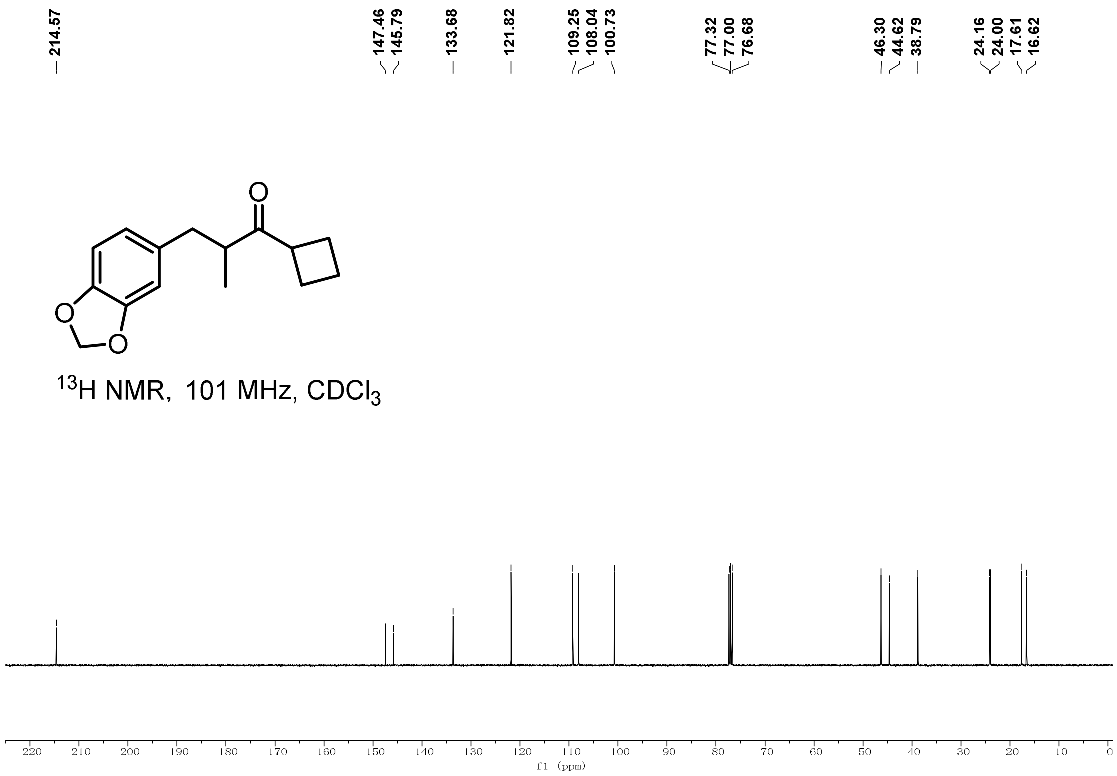
214.57
147.46 145.79 133.68 121.82 109.25 108.04 100.73 77.32 77.00 76.68 46.30 44.62 38.79 24.16 24.00 17.61 16.62
^{13}H NMR, 101 MHz, CDCl$_3$
f1 (ppm)

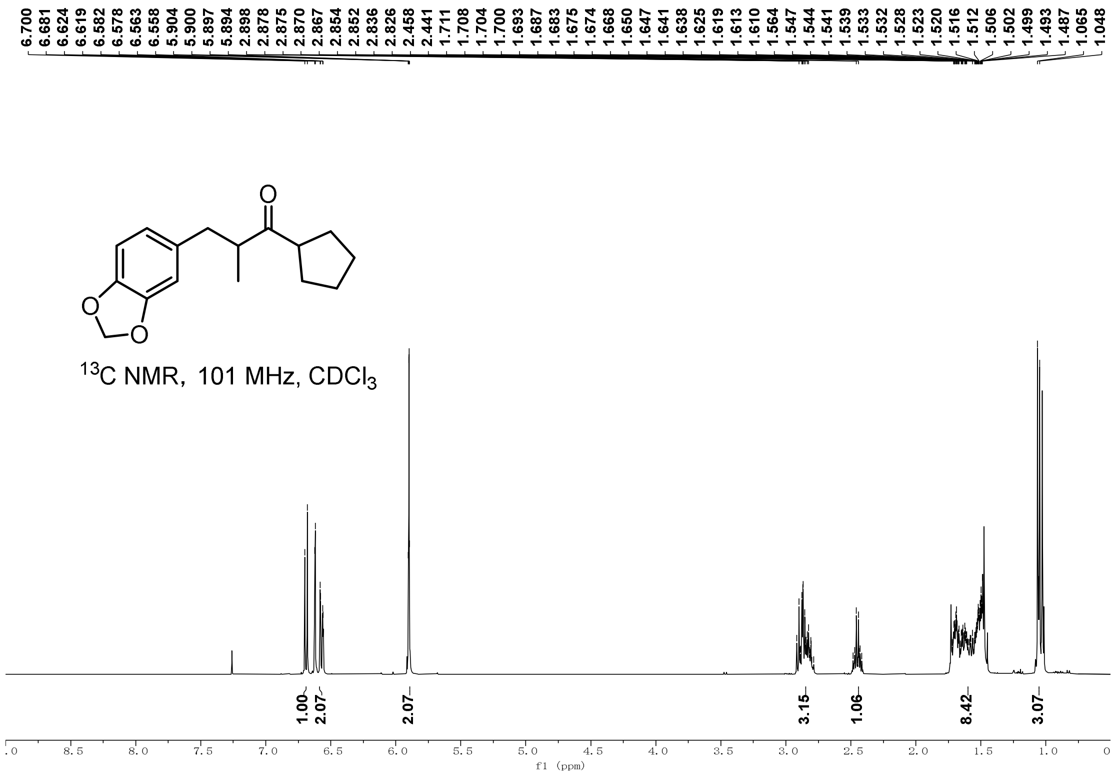
13C NMR, 101 MHz, CDCl3
f1 (ppm)

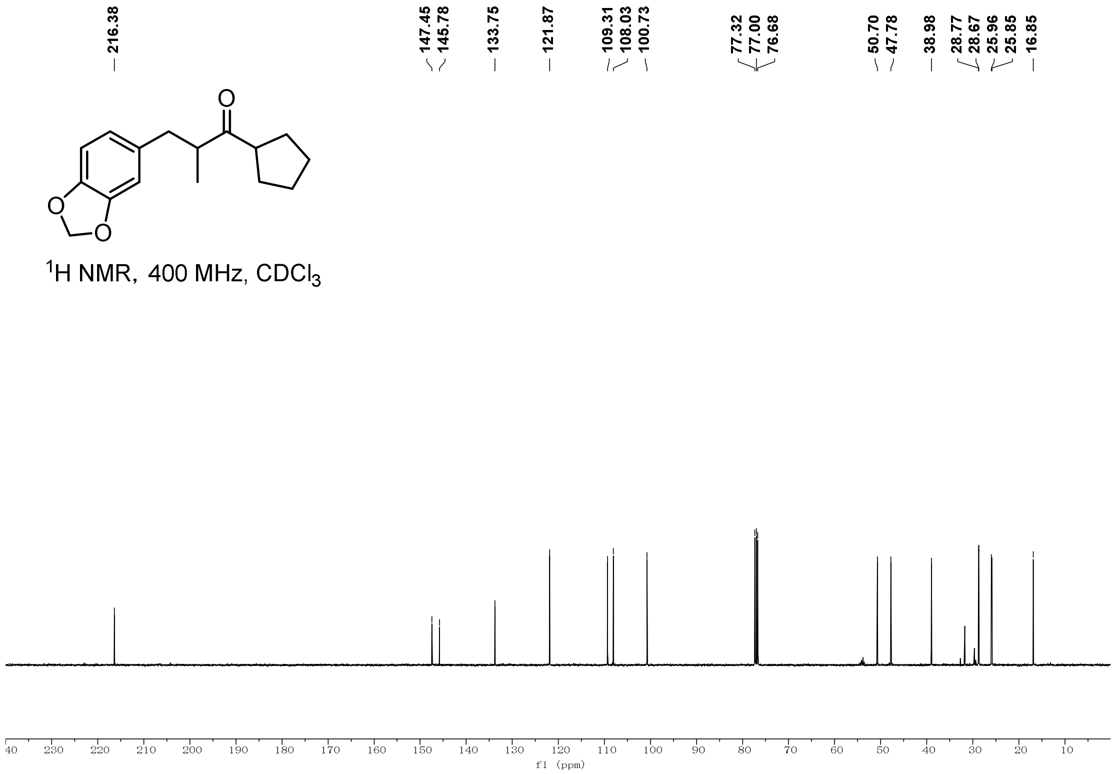
1H NMR, 400 MHz, CDCl3
f1 (ppm)

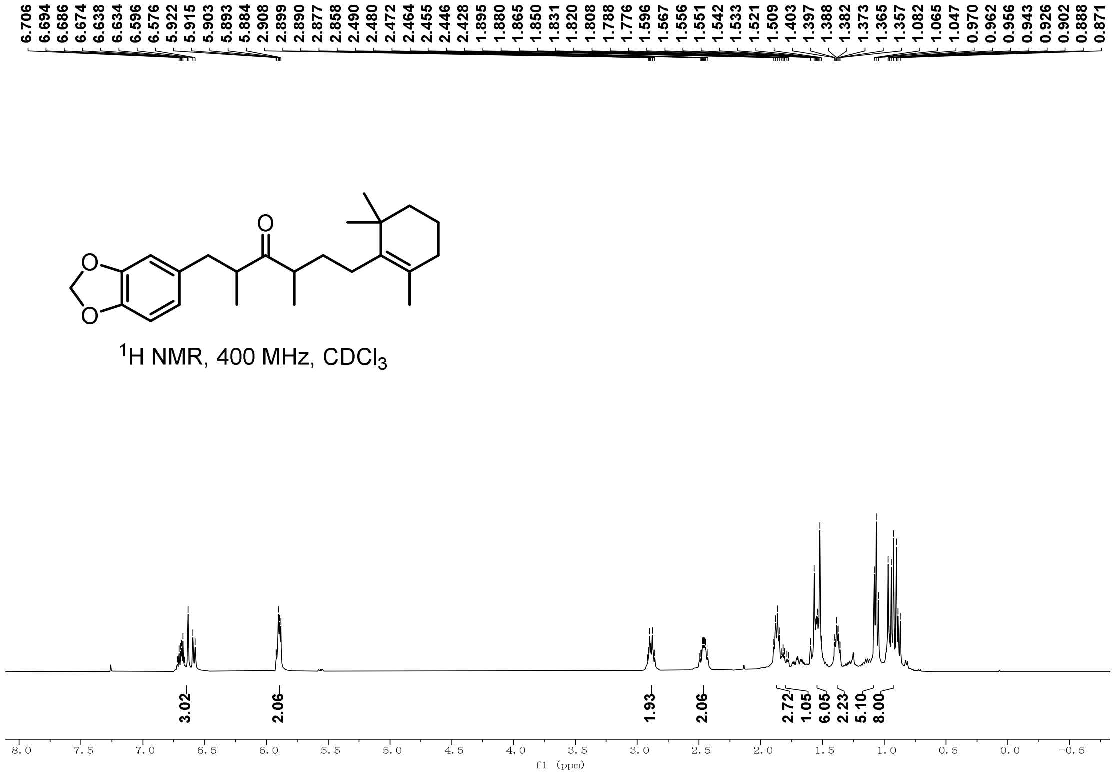

^{1}H NMR, 400 MHz, $CDCl_3$

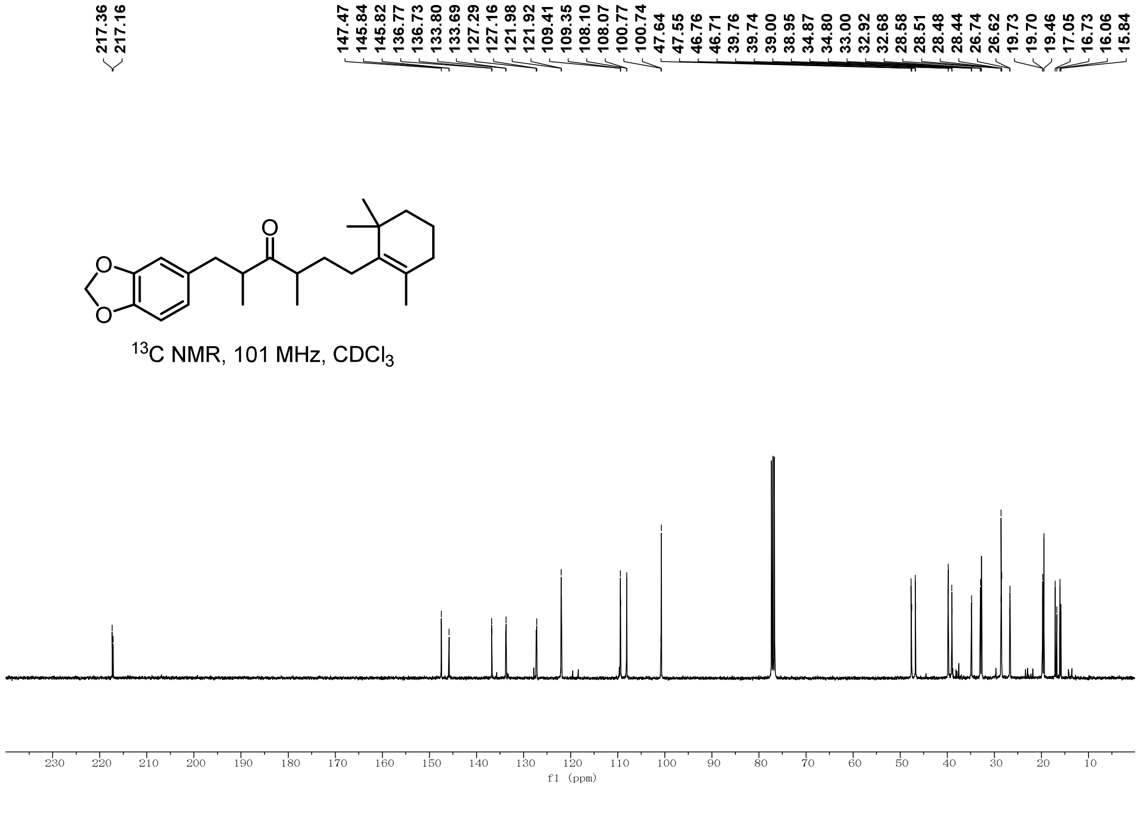

^{13}C NMR, 101 MHz, $CDCl_3$

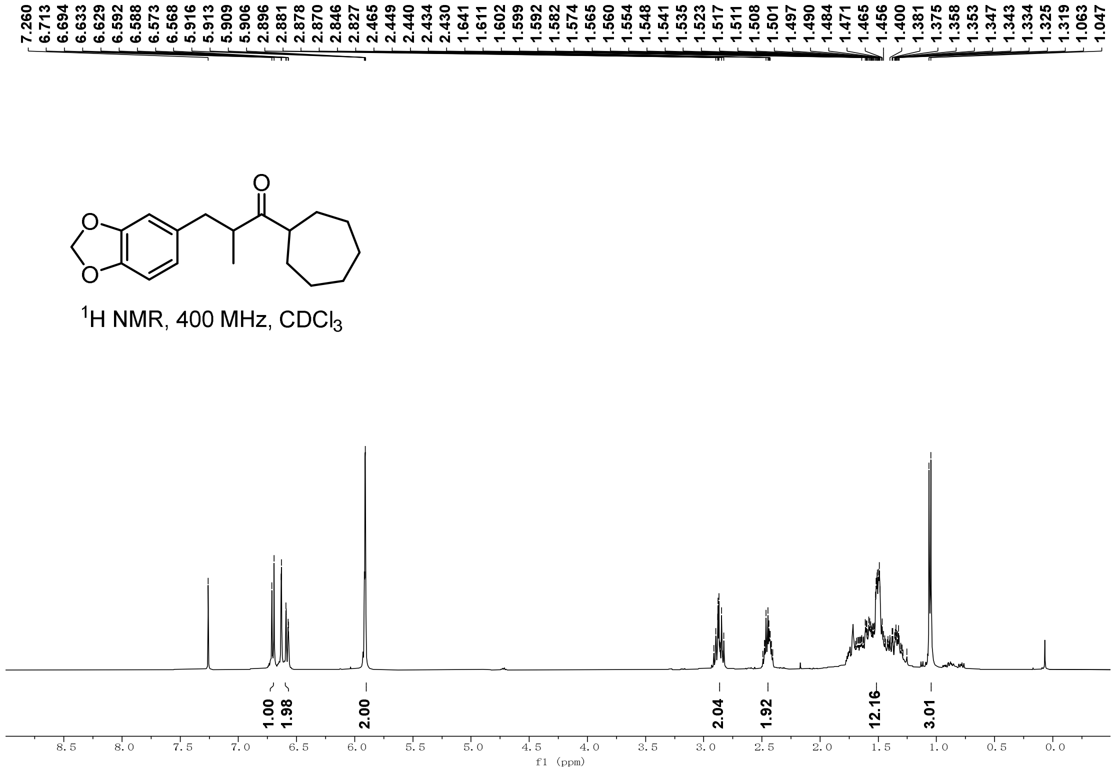
7.260 6.713 6.694 6.633 6.629 6.592 6.588 6.573 6.568 5.916 5.913 5.909 5.906 2.896 2.881 2.878 2.870 2.846 2.827 2.465 2.449 2.440 2.434 2.430 1.641 1.611 1.602 1.599 1.592 1.582 1.574 1.565 1.560 1.554 1.548 1.541 1.535 1.523 1.517 1.511 1.508 1.501 1.497 1.490 1.484 1.471 1.465 1.456 1.400 1.381 1.375 1.358 1.353 1.347 1.343 1.334 1.325 1.319 1.063 1.047
O
O
O
¹H NMR, 400 MHz, CDCl₃
1.00 1.98 2.00 2.04 1.92 12.16 3.01
8.5 8.0 7.5 7.0 6.5 6.0 5.5 5.0 4.5 4.0 3.5 3.0 2.5 2.0 1.5 1.0 0.5 0.0
f1 (ppm)

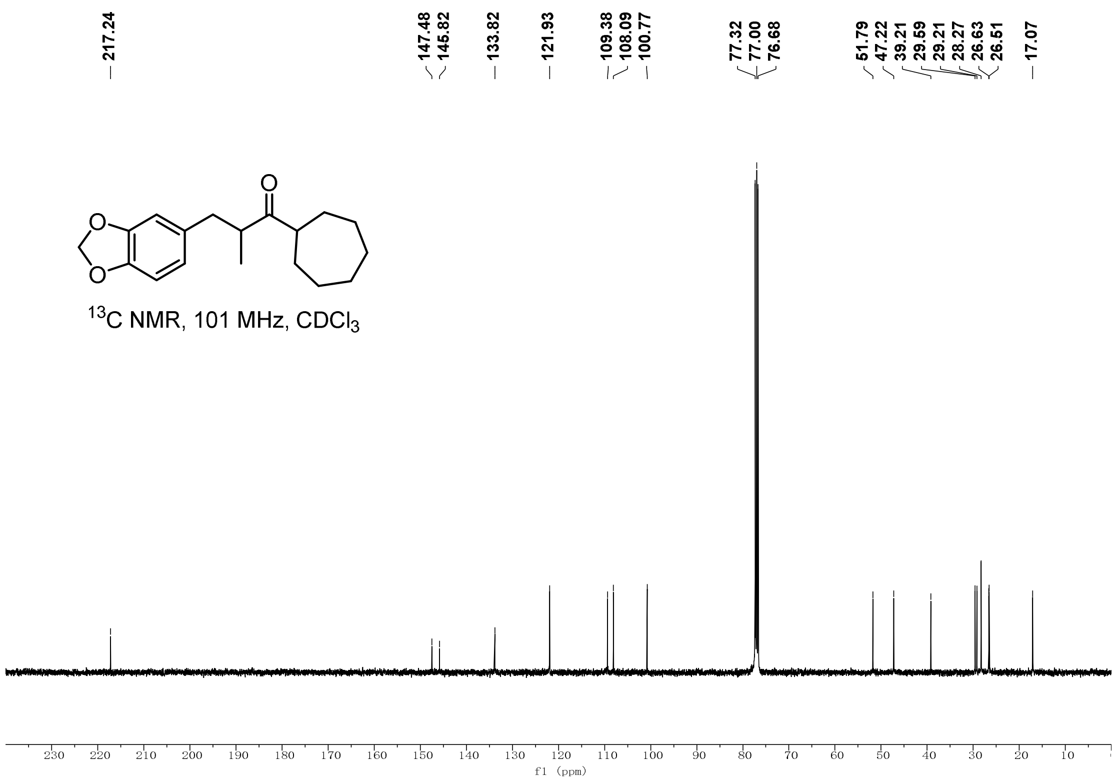
217.24 147.48 145.82 133.82 121.93 109.38 108.09 100.77 77.32 77.00 76.68 51.79 47.22 39.21 29.59 29.21 28.27 26.63 26.51 17.07
O
O
O
¹³C NMR, 101 MHz, CDCl₃
230 220 210 200 190 180 170 160 150 140 130 120 110 100 90 80 70 60 50 40 30 20 10
f1 (ppm)

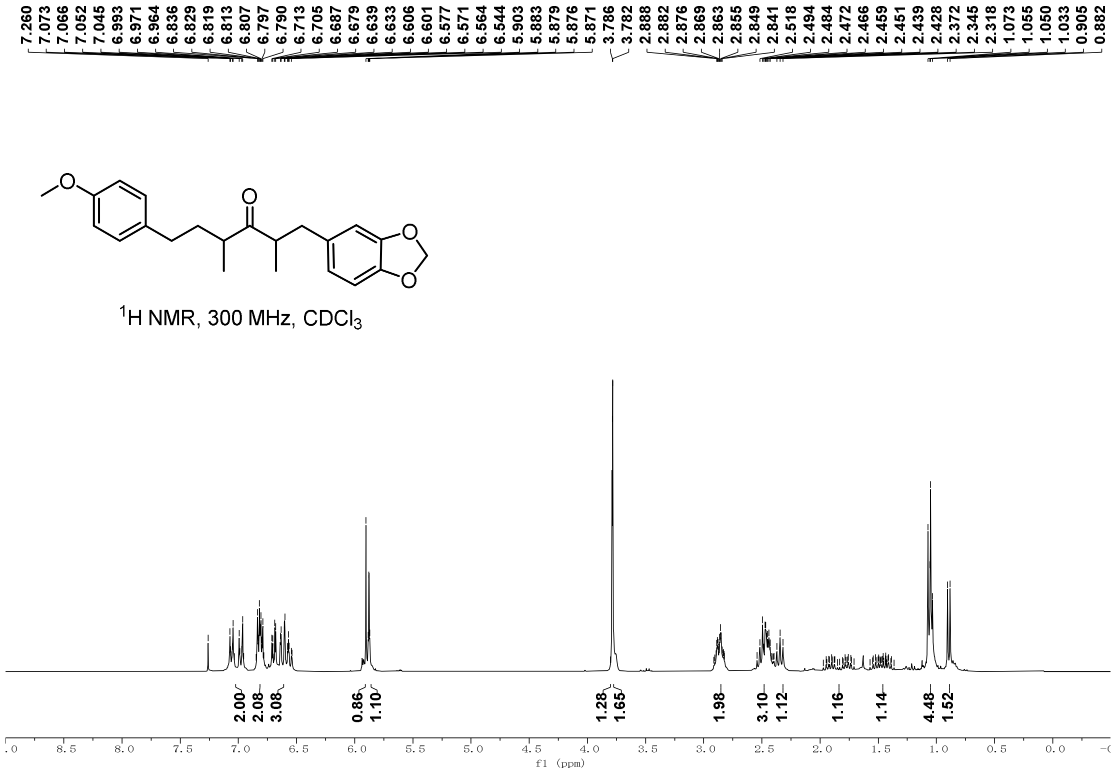
7.260 7.073 7.066 7.052 7.045 6.993 6.971 6.964 6.836 6.829 6.819 6.813 6.807 6.797 6.790 6.713 6.705 6.687 6.679 6.639 6.633 6.606 6.601 6.577 6.571 6.564 6.544 5.903 5.883 5.879 5.876 5.871 3.786 3.782 2.888 2.882 2.876 2.869 2.863 2.855 2.849 2.841 2.518 2.494 2.484 2.472 2.466 2.459 2.451 2.439 2.428 2.372 2.345 2.318 1.073 1.055 1.050 1.033 0.905 0.882
O
O
O
O
^{1}H NMR, 300 MHz, $CDCl_3$
2.00 2.08 3.08 0.86 1.10 1.28 1.65 1.98 3.10 1.12 1.16 1.14 4.48 1.52
f1 (ppm)

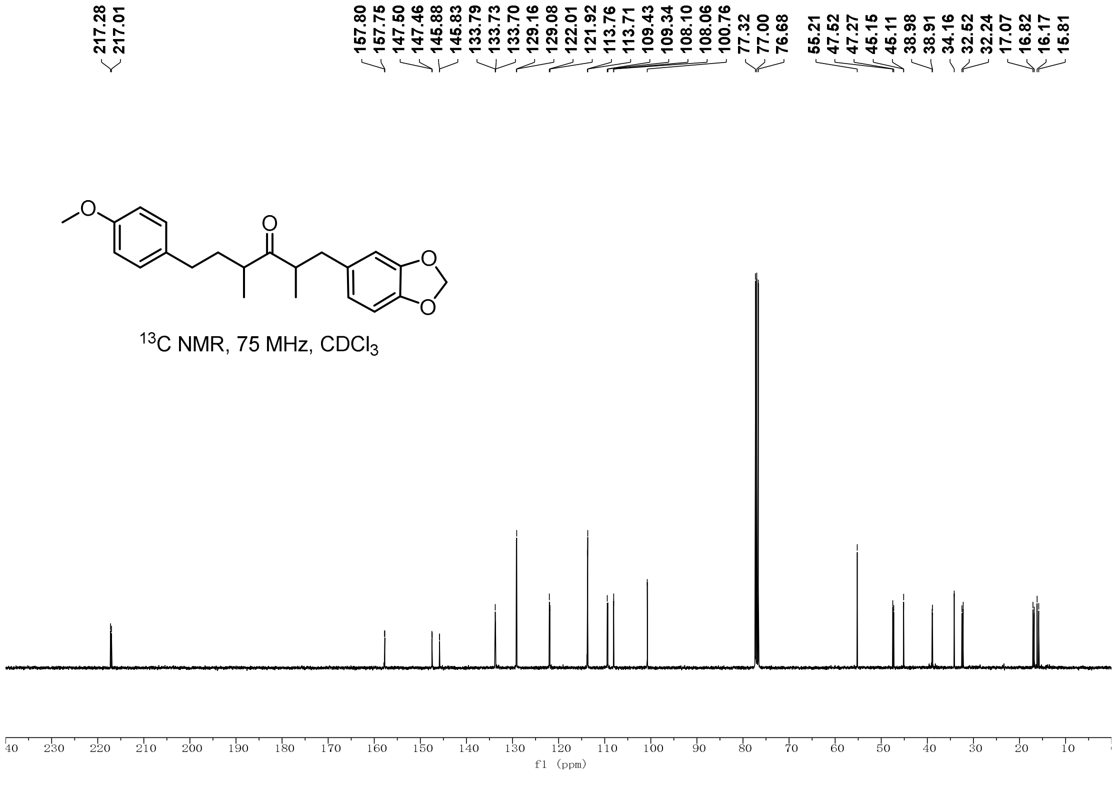
217.28 217.01 157.80 157.75 147.50 147.46 145.88 145.83 133.79 133.73 133.70 129.16 129.08 122.01 121.92 113.76 113.71 109.43 109.34 108.10 108.06 100.76 77.32 77.00 76.68 55.21 47.52 47.27 45.15 45.11 38.98 38.91 34.16 32.52 32.24 17.07 16.82 16.17 15.81
O
O
O
O
^{13}C NMR, 75 MHz, $CDCl_3$
f1 (ppm)

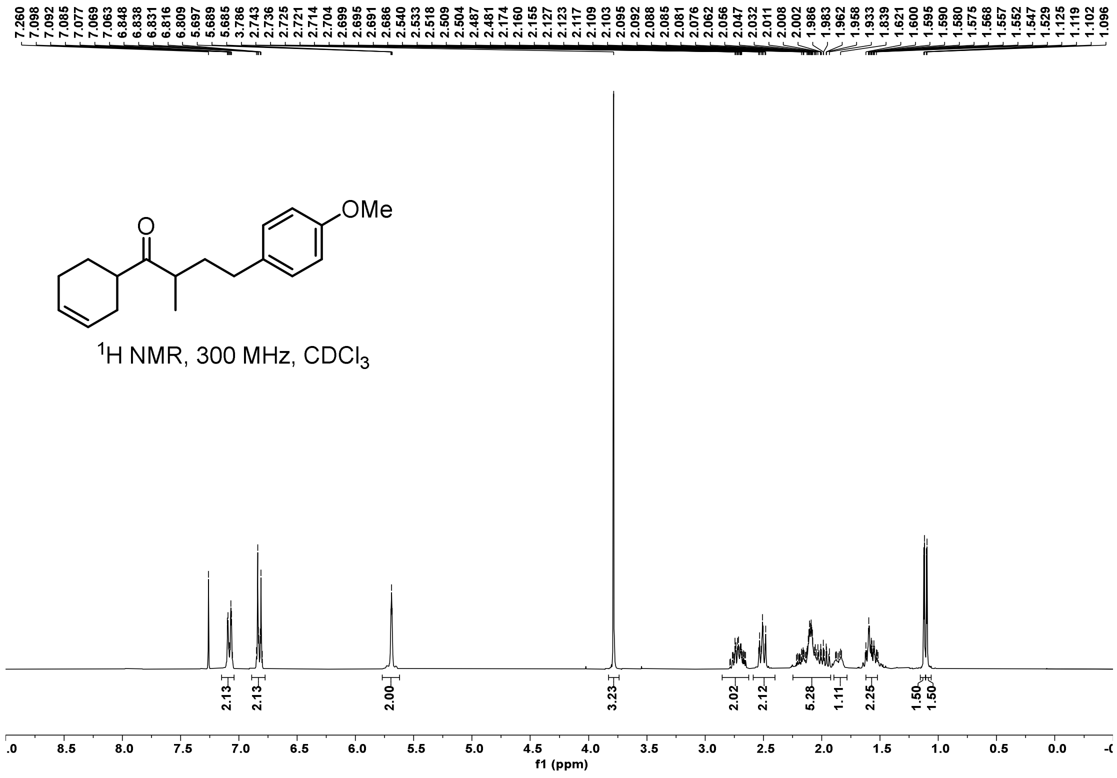
OMe
O
1H NMR, 300 MHz, CDCl3
f1 (ppm)

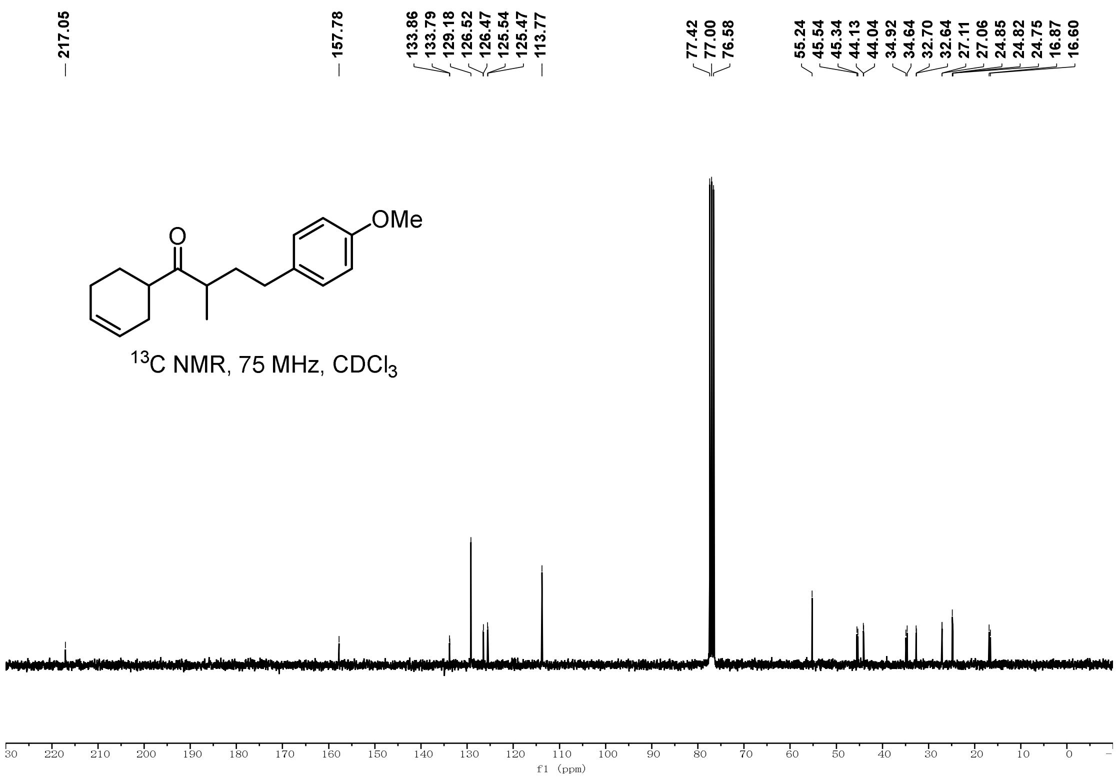
OMe
O
13C NMR, 75 MHz, CDCl3
f1 (ppm)

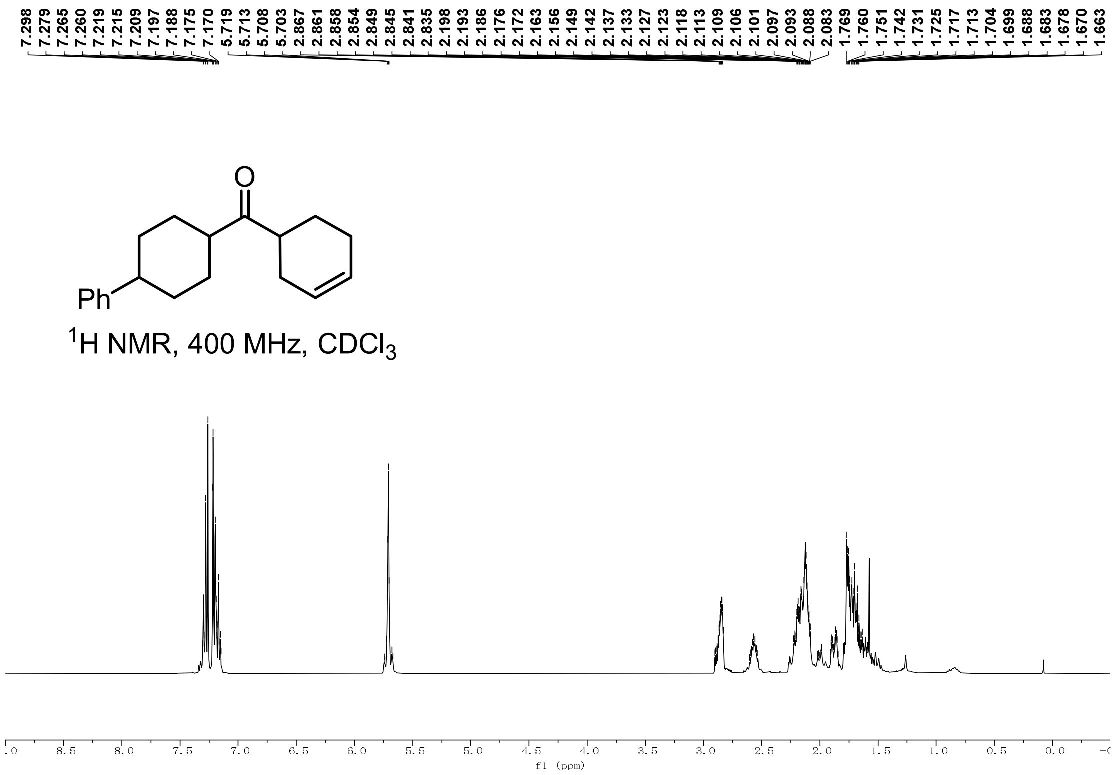
O
Ph
1H NMR, 400 MHz, CDCl3
f1 (ppm)

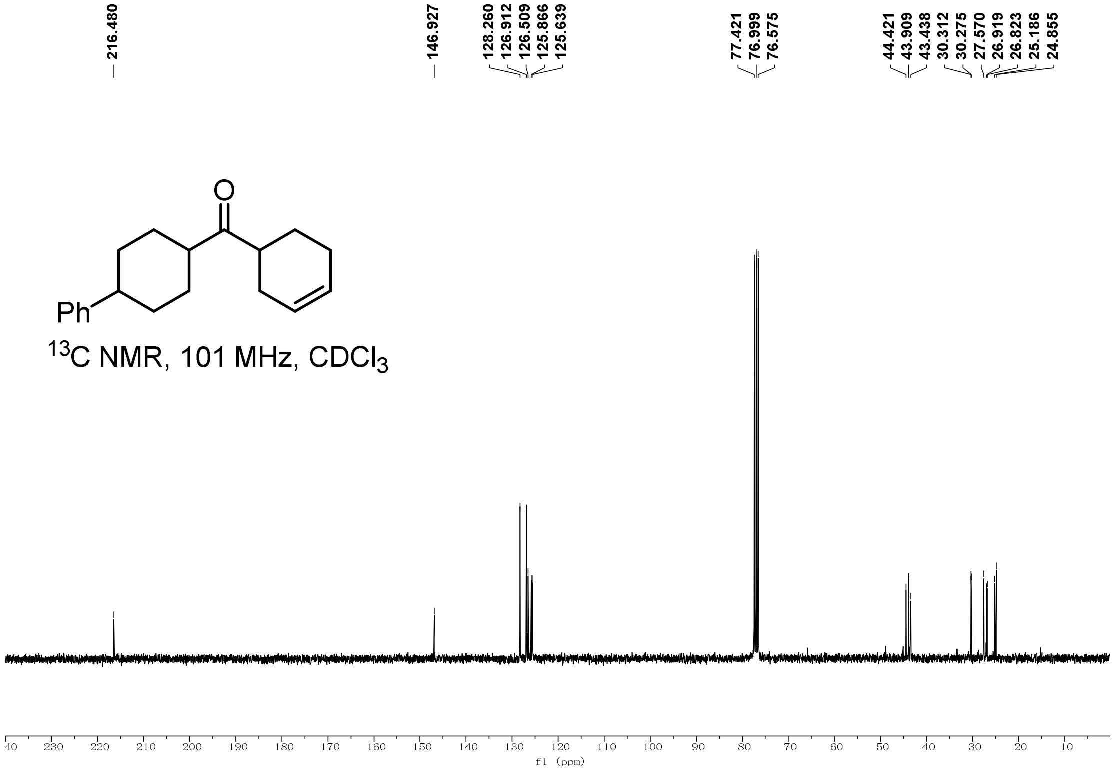
O
Ph
13C NMR, 101 MHz, CDCl3
f1 (ppm)

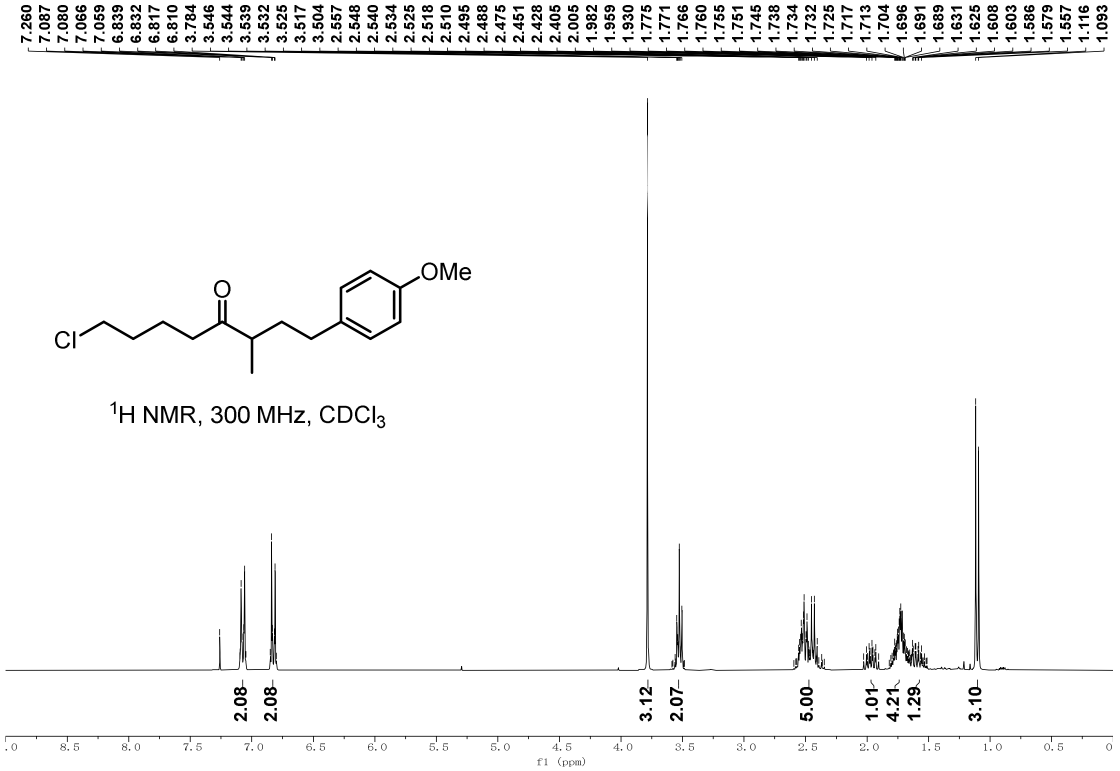
OMe
O
Cl
^{1}H NMR, 300 MHz, $CDCl_3$
7.260 7.087 7.080 7.066 7.059 6.839 6.832 6.817 6.810 3.784 3.546 3.544 3.539 3.532 3.525 3.517 3.504 2.557 2.548 2.540 2.534 2.525 2.518 2.510 2.495 2.488 2.475 2.451 2.428 2.405 2.005 1.982 1.959 1.930 1.775 1.771 1.766 1.760 1.755 1.751 1.745 1.738 1.734 1.732 1.725 1.717 1.713 1.704 1.696 1.691 1.689 1.631 1.625 1.608 1.603 1.586 1.579 1.557 1.116 1.093
2.08 2.08 3.12 2.07 5.00 1.01 4.21 1.29 3.10
f1 (ppm)

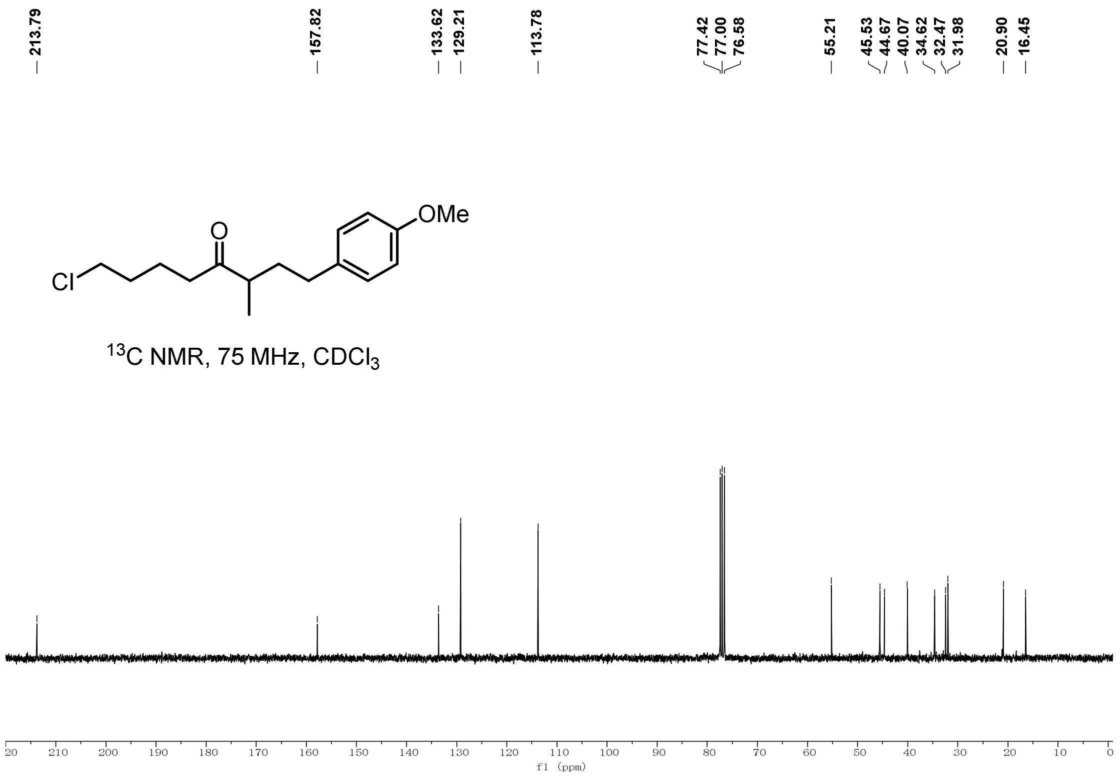
OMe
O
Cl
^{13}C NMR, 75 MHz, $CDCl_3$
213.79 157.82 133.62 129.21 113.78 77.42 77.00 76.58 55.21 45.53 44.67 40.07 34.62 32.47 31.98 20.90 16.45
f1 (ppm)

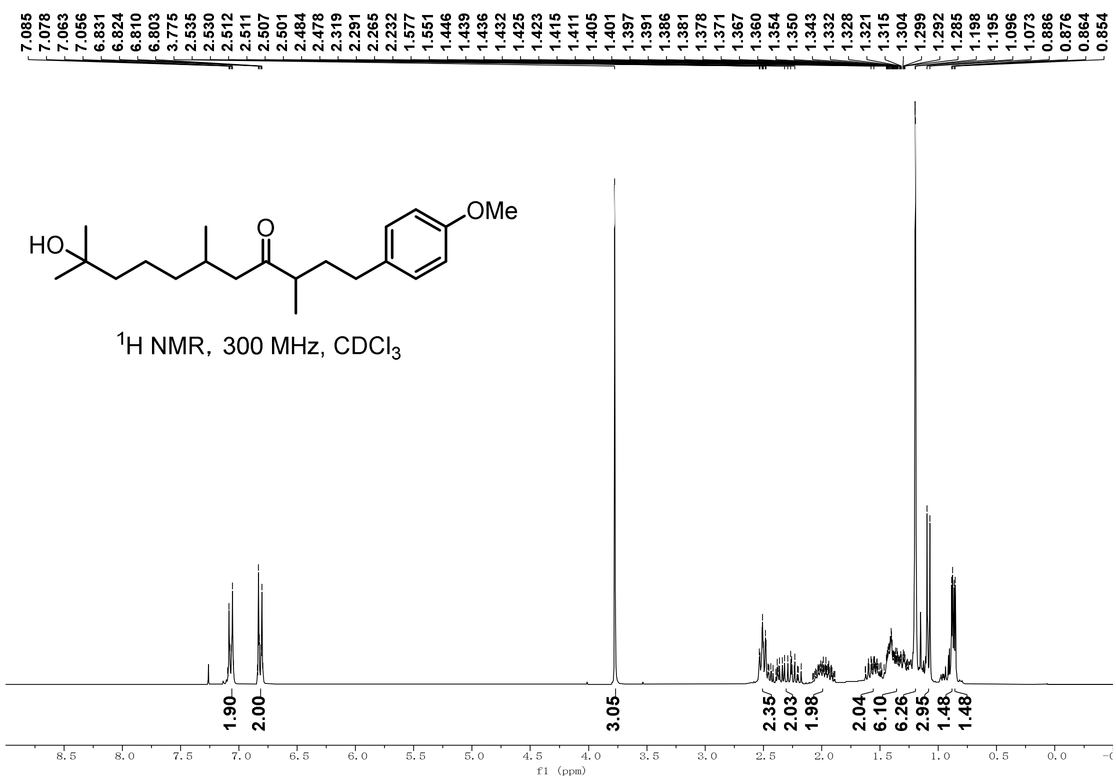
7.085 7.078 7.063 7.056 6.831 6.824 6.810 6.803 3.775 2.535 2.530 2.512 2.511 2.507 2.501 2.484 2.478 2.319 2.291 2.265 2.232 1.577 1.551 1.446 1.439 1.436 1.432 1.425 1.423 1.415 1.411 1.405 1.401 1.397 1.391 1.386 1.381 1.378 1.371 1.367 1.360 1.354 1.350 1.343 1.332 1.328 1.321 1.315 1.304 1.299 1.292 1.285 1.198 1.195 1.096 1.073 0.886 0.876 0.864 0.854
HO
O
OMe
1H NMR, 300 MHz, CDCl3
1.90 2.00 3.05 2.35 2.03 1.98 2.04 6.10 6.26 2.95 1.48 1.48
f1 (ppm)

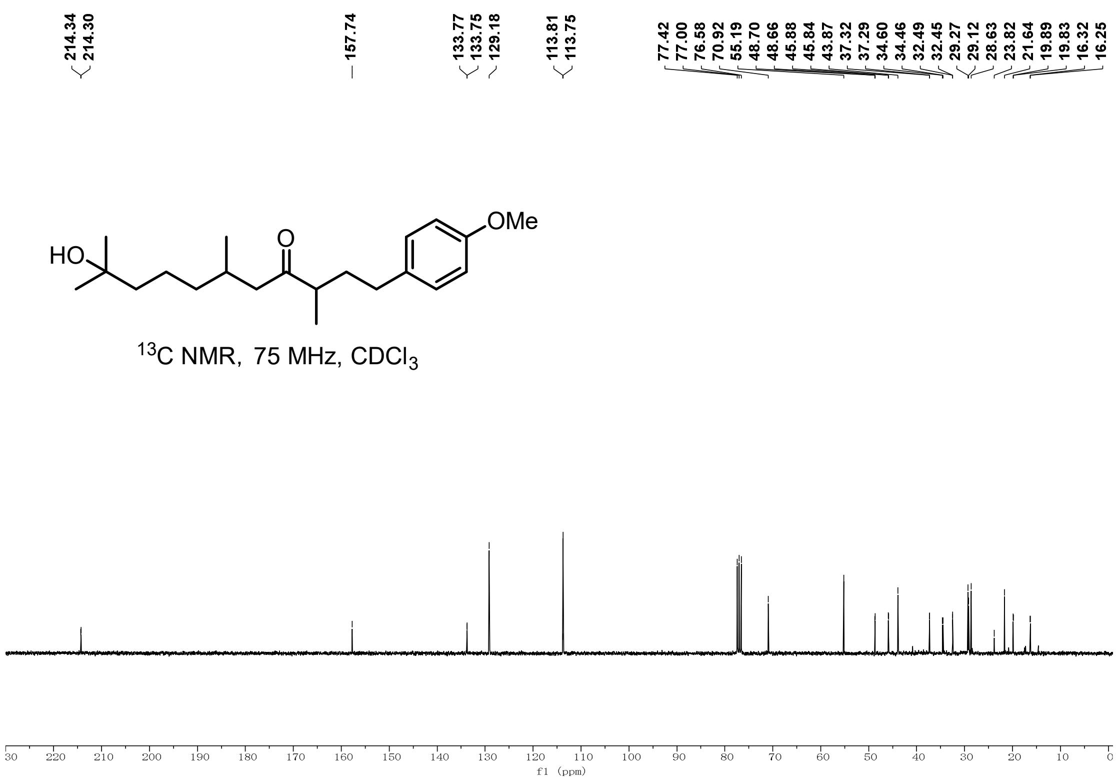
214.34 214.30 157.74 133.77 133.75 129.18 113.81 113.75 77.42 77.00 76.58 70.92 55.19 48.70 48.66 45.88 45.84 43.87 37.32 37.29 34.60 34.46 32.49 32.45 29.27 29.12 28.63 23.82 21.64 19.89 19.83 16.32 16.25
HO
O
OMe
13C NMR, 75 MHz, CDCl3
f1 (ppm)

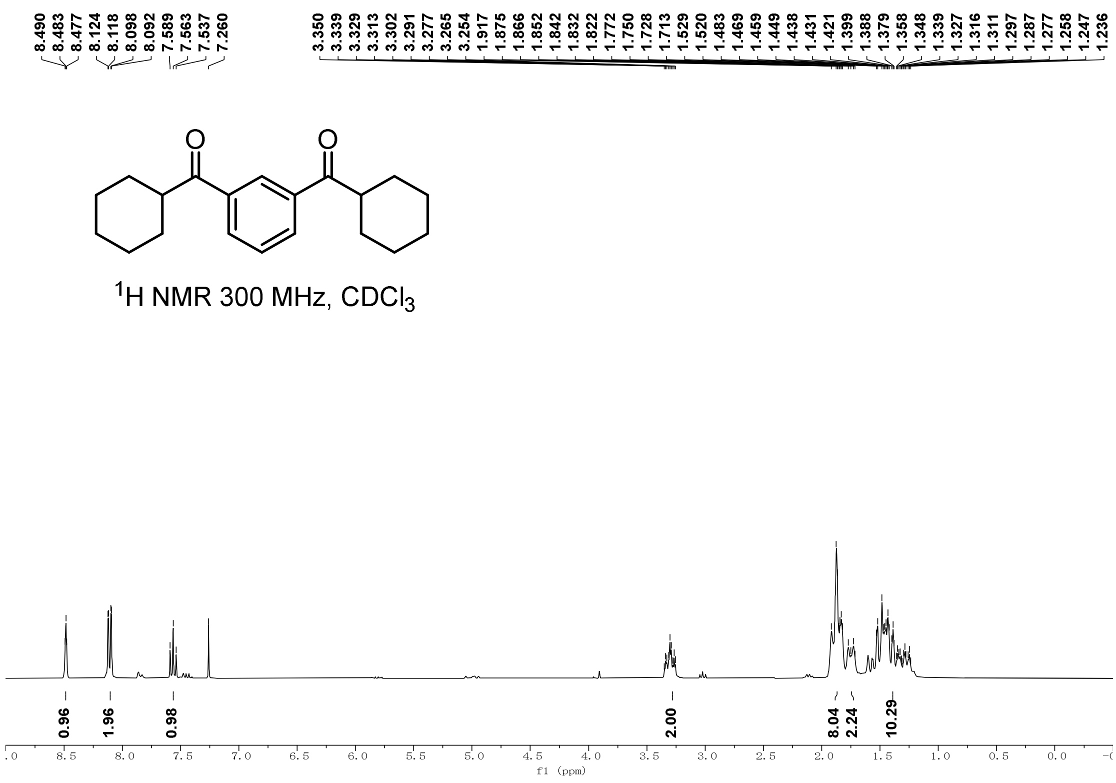
8.490 8.483 8.477 8.124 8.118 8.098 8.092 7.589 7.563 7.537 7.260
3.350 3.339 3.329 3.313 3.302 3.291 3.277 3.265 3.254 1.917 1.875 1.866 1.852 1.842 1.832 1.822 1.772 1.750 1.728 1.713 1.529 1.520 1.483 1.469 1.459 1.449 1.438 1.431 1.421 1.399 1.388 1.379 1.358 1.348 1.339 1.327 1.316 1.311 1.297 1.287 1.277 1.258 1.247 1.236
O
O
^{1}H NMR 300 MHz, CDCl$_3$
0.96
1.96
0.98
2.00
8.04
2.24
10.29
f1 (ppm)

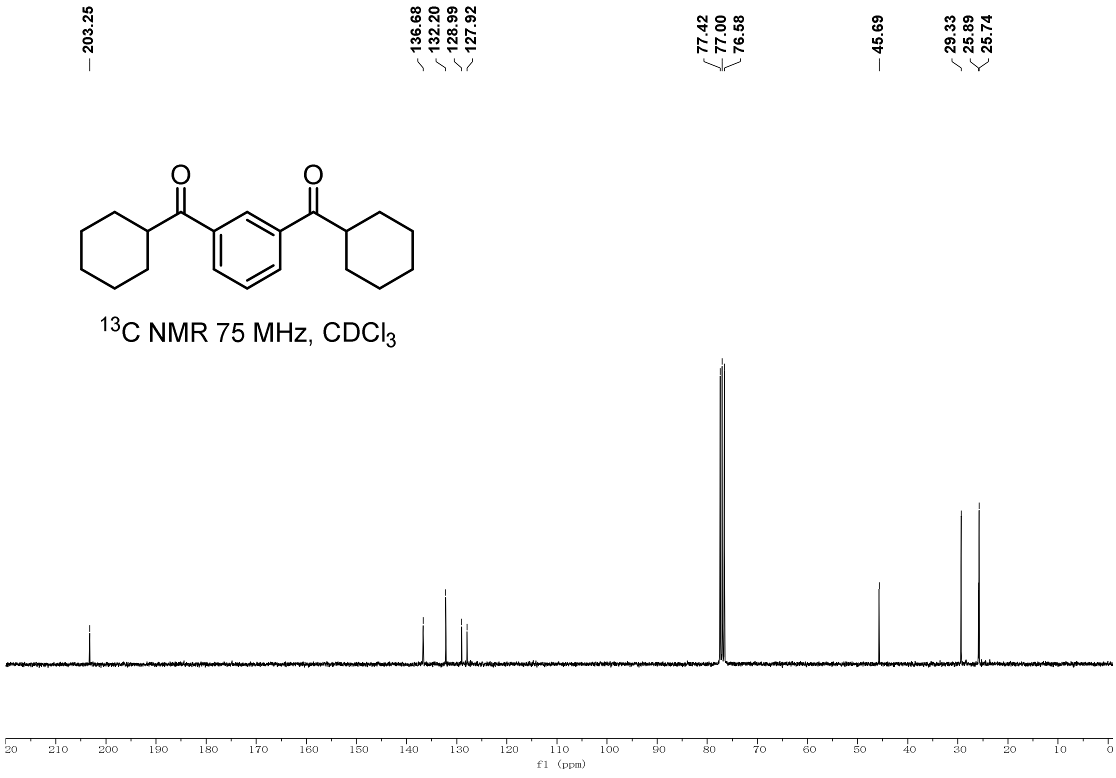
203.25
136.68 132.20 128.99 127.92
77.42 77.00 76.58
45.69
29.33 25.89 25.74
O
O
^{13}C NMR 75 MHz, CDCl$_3$
f1 (ppm)

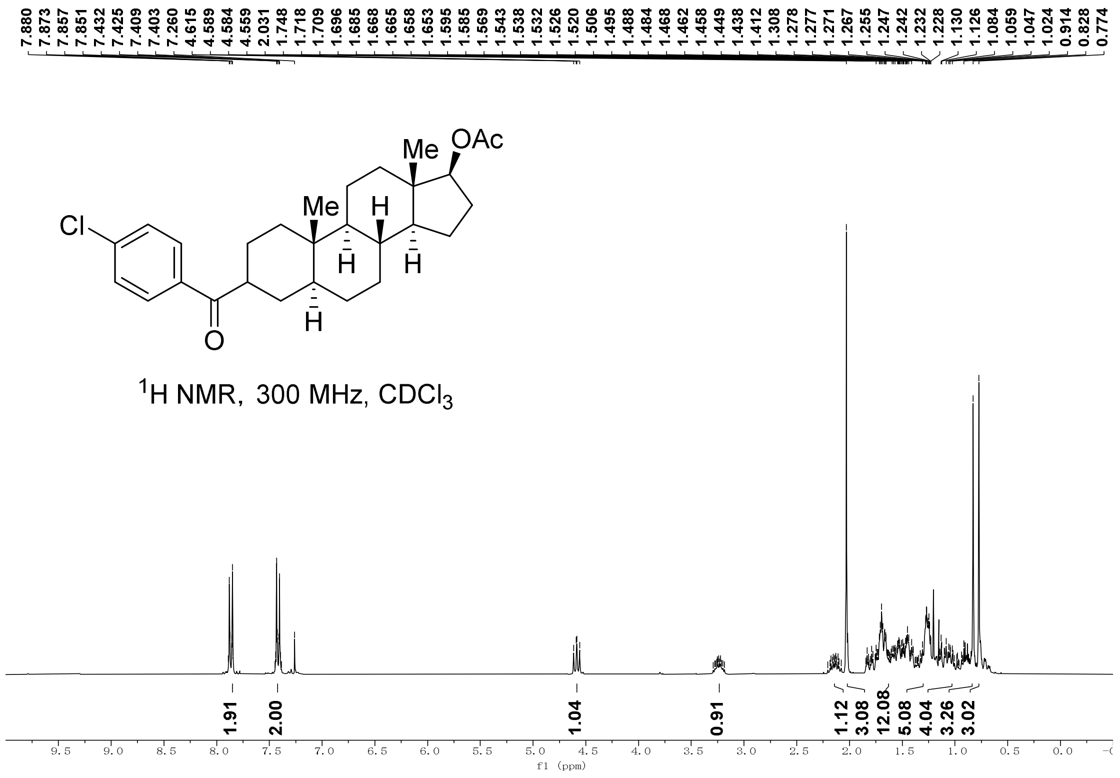

OAc
Me
Me
H
H
Cl
H
O
H
^{1}H NMR, 300 MHz, $CDCl_3$
f1 (ppm)

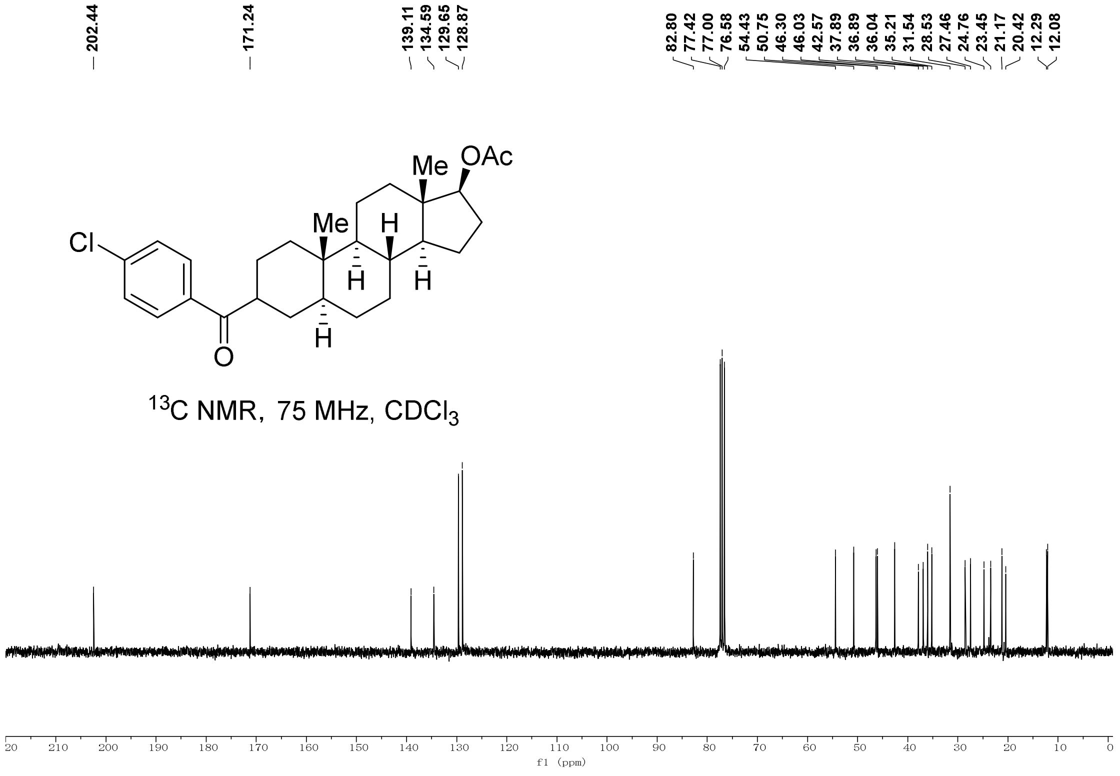

202.44
171.24
139.11
134.59
129.65
128.87
82.80
77.42
77.00
76.58
54.43
50.75
46.30
46.03
42.57
37.89
36.89
36.04
35.21
31.54
28.53
27.46
24.76
23.45
21.17
20.42
12.29
12.08
OAc
Me
Me
H
H
Cl
H
O
H
^{13}C NMR, 75 MHz, $CDCl_3$
f1 (ppm)

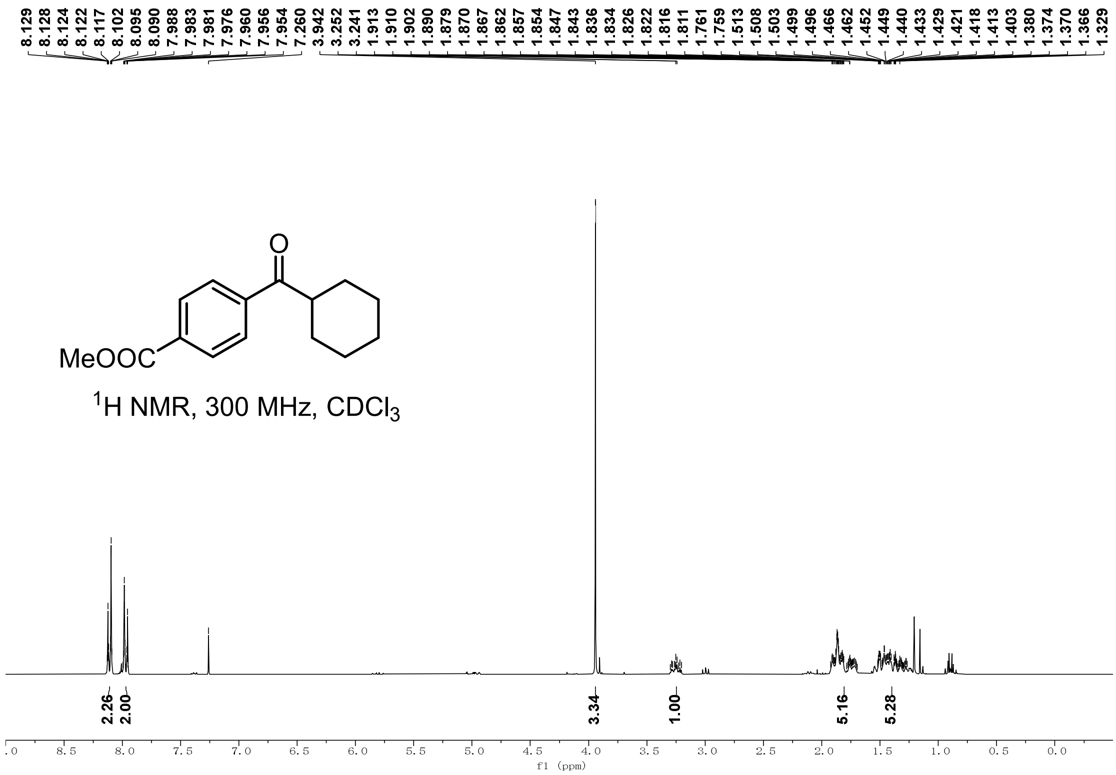
8.129 8.128 8.124 8.122 8.117 8.102 8.095 8.090 7.988 7.983 7.981 7.976 7.960 7.956 7.954 7.260 3.942 3.252 3.241 1.913 1.910 1.902 1.890 1.879 1.870 1.867 1.862 1.857 1.854 1.847 1.843 1.836 1.834 1.826 1.822 1.816 1.811 1.761 1.759 1.513 1.508 1.503 1.499 1.496 1.466 1.462 1.452 1.449 1.440 1.433 1.429 1.421 1.418 1.413 1.403 1.380 1.374 1.370 1.366 1.329
O
MeOOC
1H NMR, 300 MHz, CDCl3
2.26
2.00
3.34
1.00
5.16
5.28
f1 (ppm)

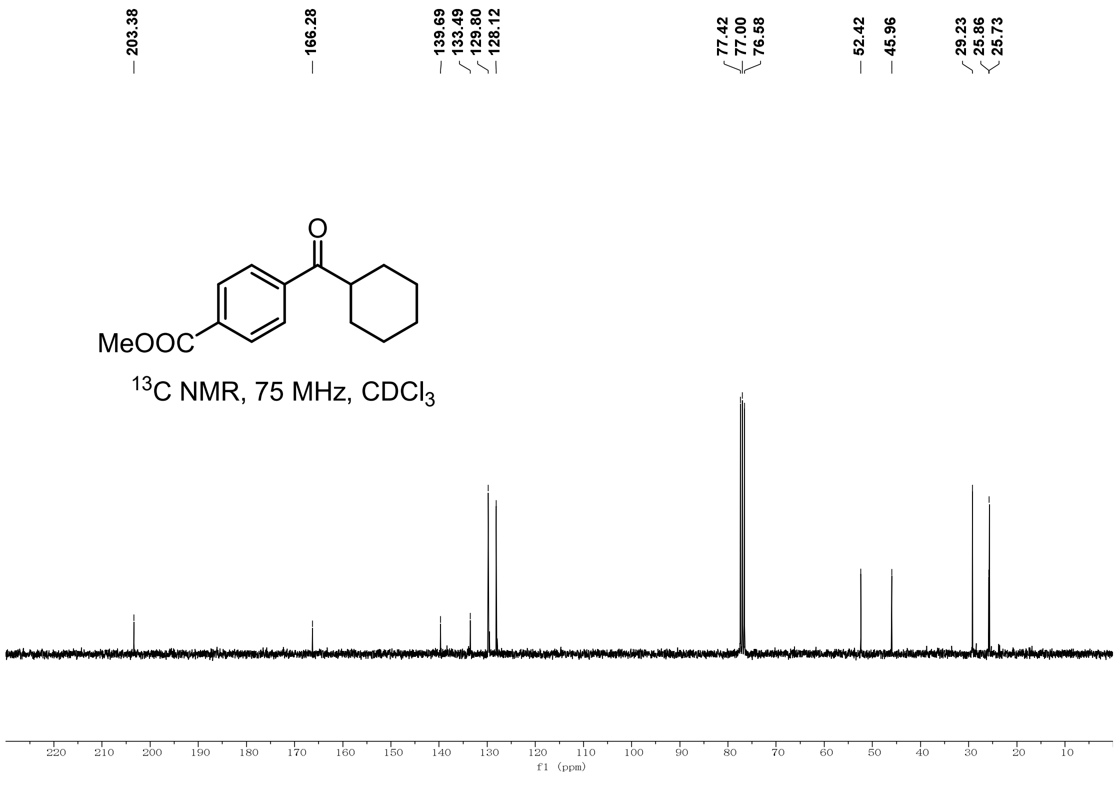
203.38
166.28
139.69
133.49
129.80
128.12
77.42
77.00
76.58
52.42
45.96
29.23
25.86
25.73
O
MeOOC
13C NMR, 75 MHz, CDCl3
f1 (ppm)

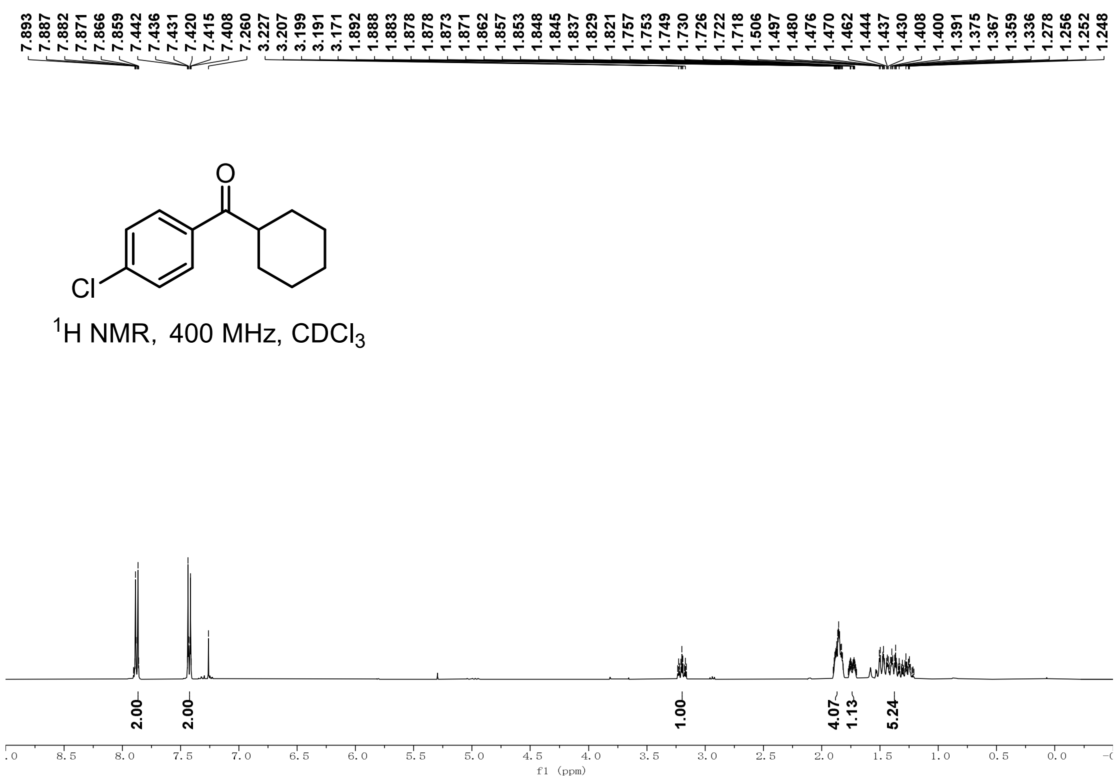

7.893 7.887 7.882 7.871 7.866 7.859 7.442 7.436 7.431 7.420 7.415 7.408 7.260 3.227 3.207 3.199 3.191 3.171 1.892 1.888 1.883 1.878 1.878 1.873 1.871 1.862 1.857 1.853 1.848 1.845 1.837 1.829 1.821 1.757 1.753 1.749 1.730 1.726 1.722 1.718 1.506 1.497 1.480 1.476 1.470 1.462 1.444 1.437 1.430 1.408 1.400 1.391 1.375 1.367 1.359 1.336 1.278 1.256 1.252 1.248
O
Cl
^{1}H NMR, 400 MHz, CDCl$_3$
2.00 2.00 1.00 4.07 1.13 5.24
f1 (ppm)

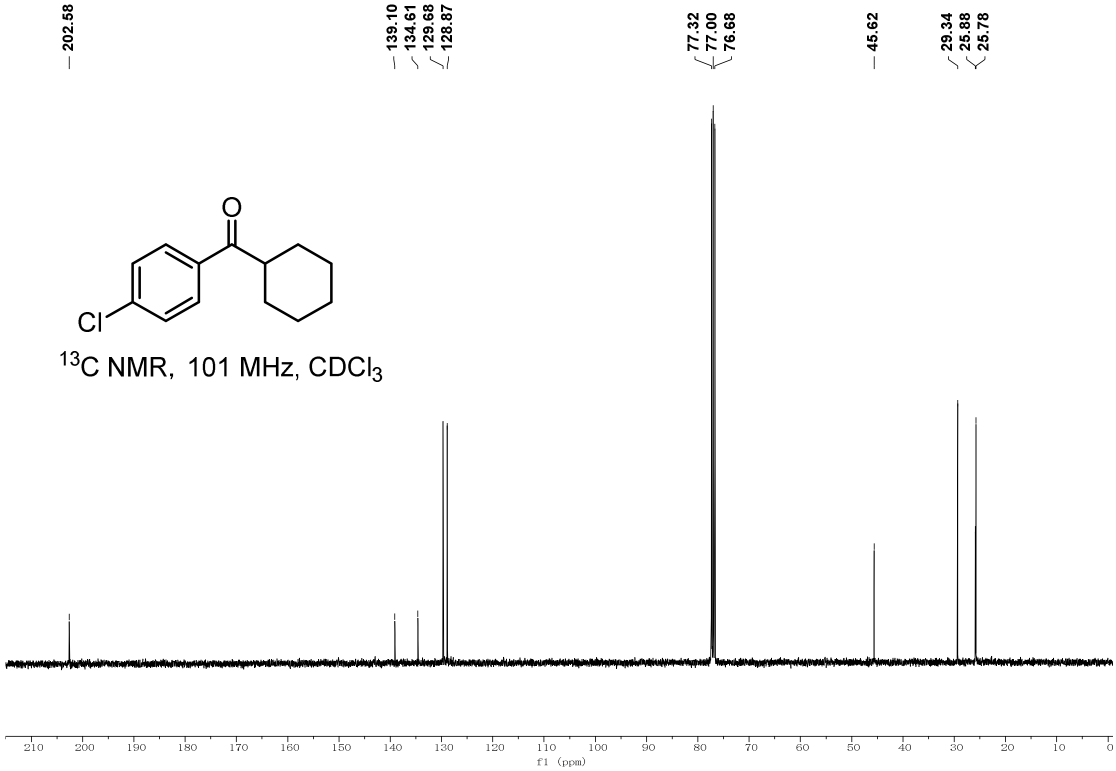

202.58 139.10 134.61 129.68 128.87 77.32 77.00 76.68 45.62 29.34 25.88 25.78
O
Cl
^{13}C NMR, 101 MHz, CDCl$_3$
210 200 190 180 170 160 150 140 130 120 110 100 90 80 70 60 50 40 30 20 10 0
f1 (ppm)

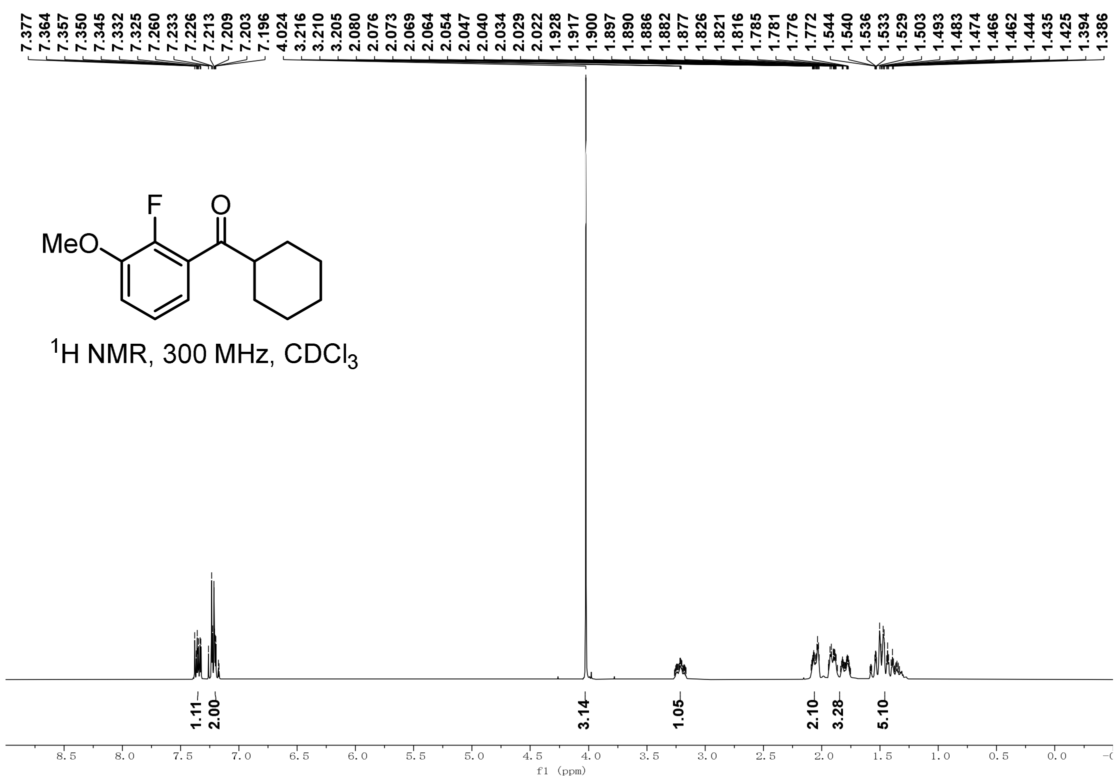
7.377 7.364 7.357 7.350 7.345 7.332 7.325 7.260 7.233 7.226 7.213 7.209 7.203 7.196 4.024 3.216 3.210 3.205 2.080 2.076 2.073 2.069 2.064 2.054 2.047 2.040 2.034 2.029 2.022 1.928 1.917 1.900 1.897 1.890 1.886 1.882 1.877 1.826 1.821 1.816 1.785 1.781 1.776 1.772 1.544 1.540 1.536 1.533 1.529 1.503 1.493 1.483 1.474 1.466 1.462 1.444 1.435 1.425 1.394 1.386
F
O
MeO
1H NMR, 300 MHz, CDCl3
1.11
2.00
3.14
1.05
2.10
3.28
5.10
f1 (ppm)

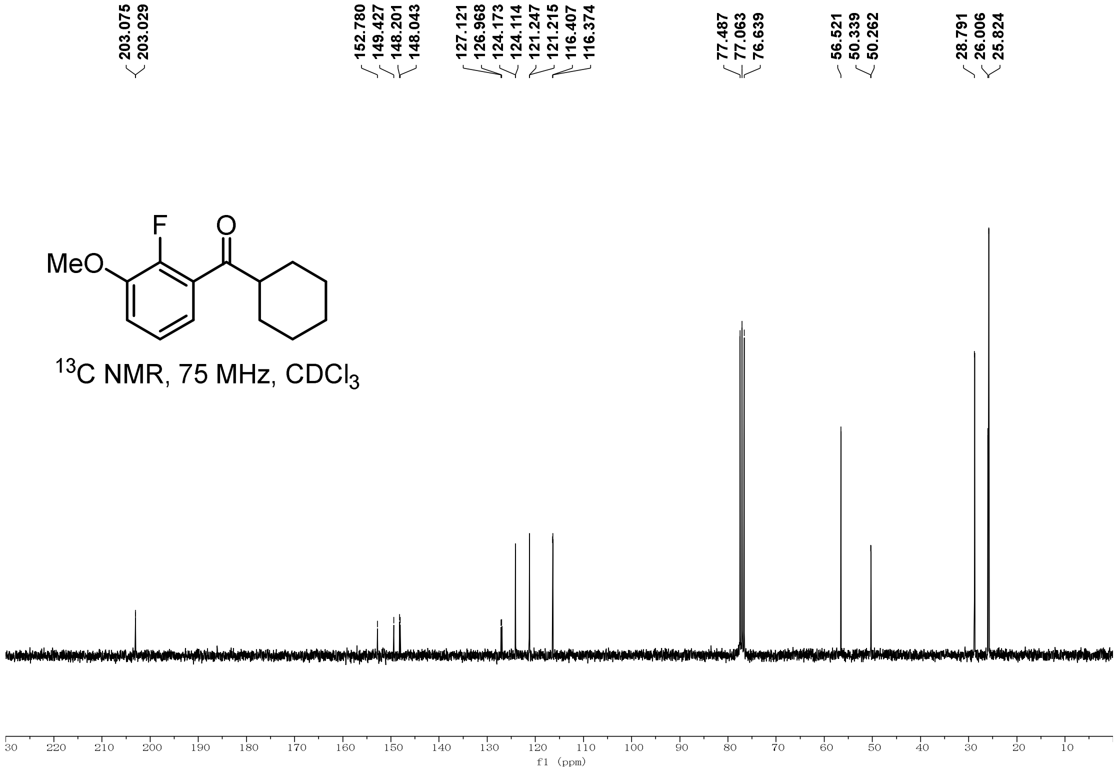
203.075 203.029
152.780 149.427 148.201 148.043
127.121 126.968 124.173 124.114 121.247 121.215 116.407 116.374
77.487 77.063 76.639
56.521 50.339 50.262
28.791 26.006 25.824
F
O
MeO
13C NMR, 75 MHz, CDCl3
f1 (ppm)

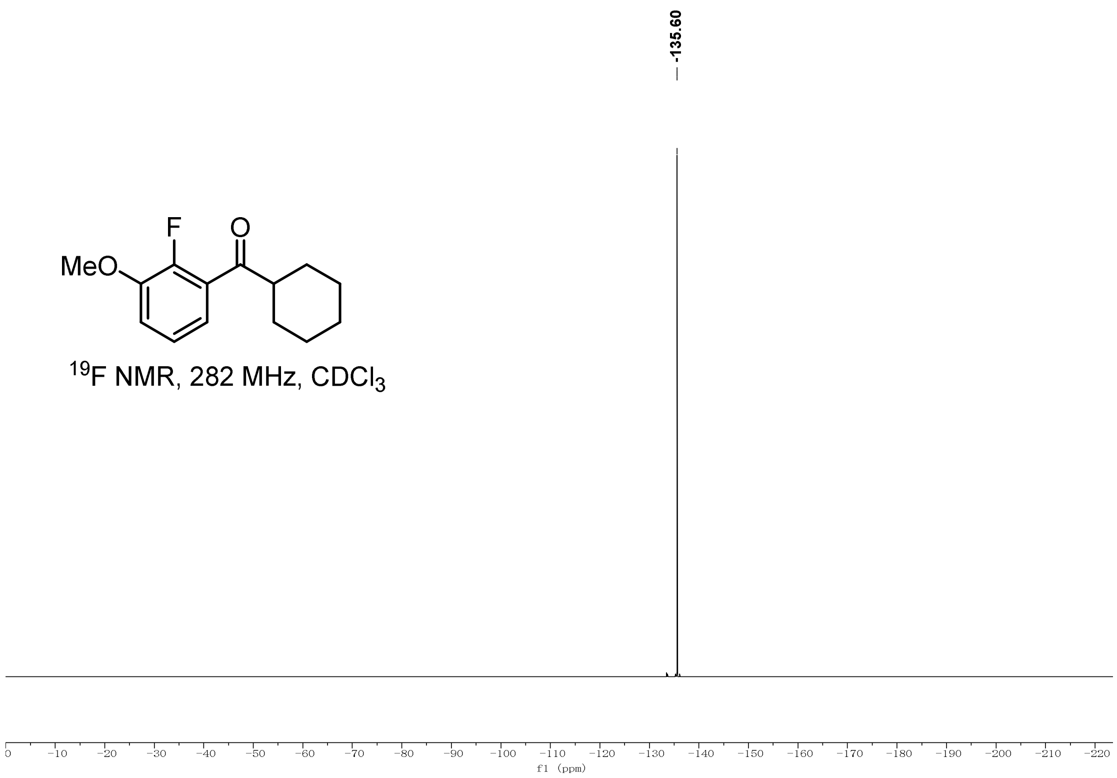
-135.60
MeO
F
O
19F NMR, 282 MHz, CDCl3
0 −10 −20 −30 −40 −50 −60 −70 −80 −90 −100 −110 −120 −130 −140 −150 −160 −170 −180 −190 −200 −210 −220
f1 (ppm)

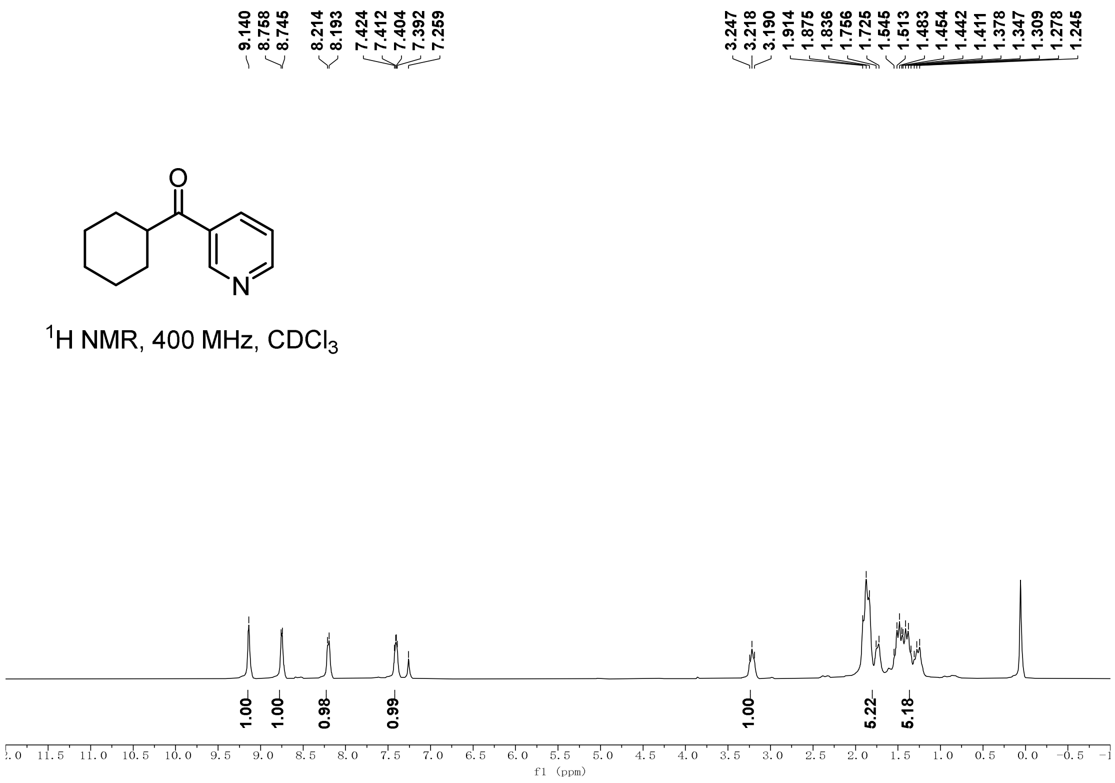
9.140
8.758
8.745
8.214
8.193
7.424
7.412
7.404
7.392
7.259
3.247
3.218
3.190
1.914
1.875
1.836
1.756
1.725
1.545
1.513
1.483
1.454
1.442
1.411
1.378
1.347
1.309
1.278
1.245
O
N
^{1}H NMR, 400 MHz, $CDCl_3$
1.00
1.00
0.98
0.99
1.00
5.22
5.18
f1 (ppm)

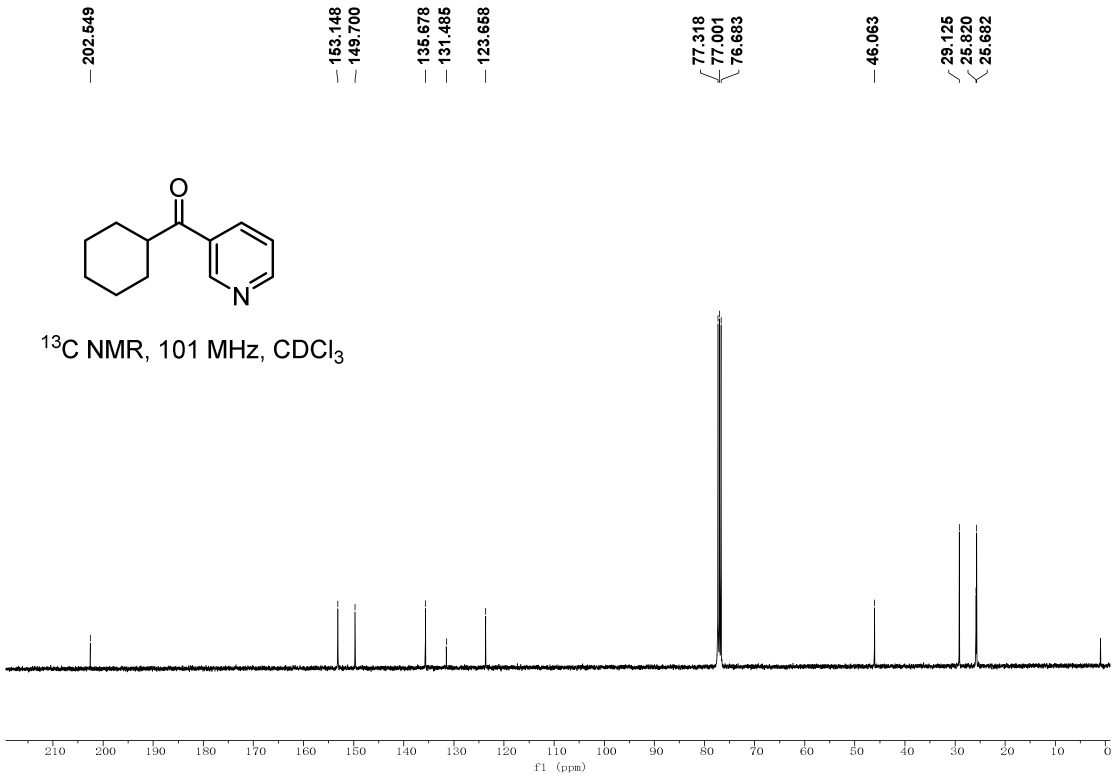
202.549
153.148
149.700
135.678
131.485
123.658
77.318
77.001
76.683
46.063
29.125
25.820
25.682
O
N
^{13}C NMR, 101 MHz, $CDCl_3$
f1 (ppm)

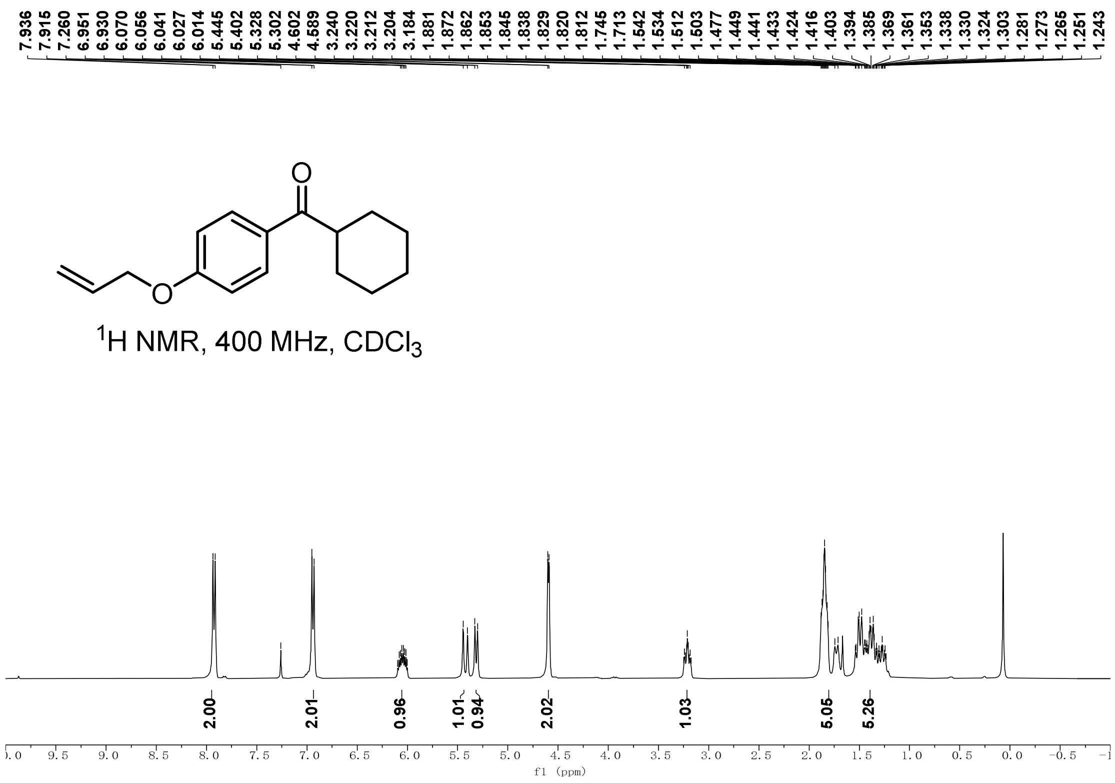
7.936 7.915 7.260 6.951 6.930 6.070 6.056 6.041 6.027 6.014 5.445 5.402 5.328 5.302 4.602 4.589 3.240 3.220 3.212 3.204 3.184 1.881 1.872 1.862 1.853 1.845 1.838 1.829 1.820 1.812 1.745 1.713 1.542 1.534 1.512 1.503 1.477 1.449 1.441 1.433 1.424 1.416 1.403 1.394 1.385 1.369 1.361 1.353 1.338 1.330 1.324 1.303 1.281 1.273 1.265 1.251 1.243
O
O
^{1}H NMR, 400 MHz, $CDCl_3$
2.00 2.01 0.96 1.01 0.94 2.02 1.03 5.05 5.26
f1 (ppm)

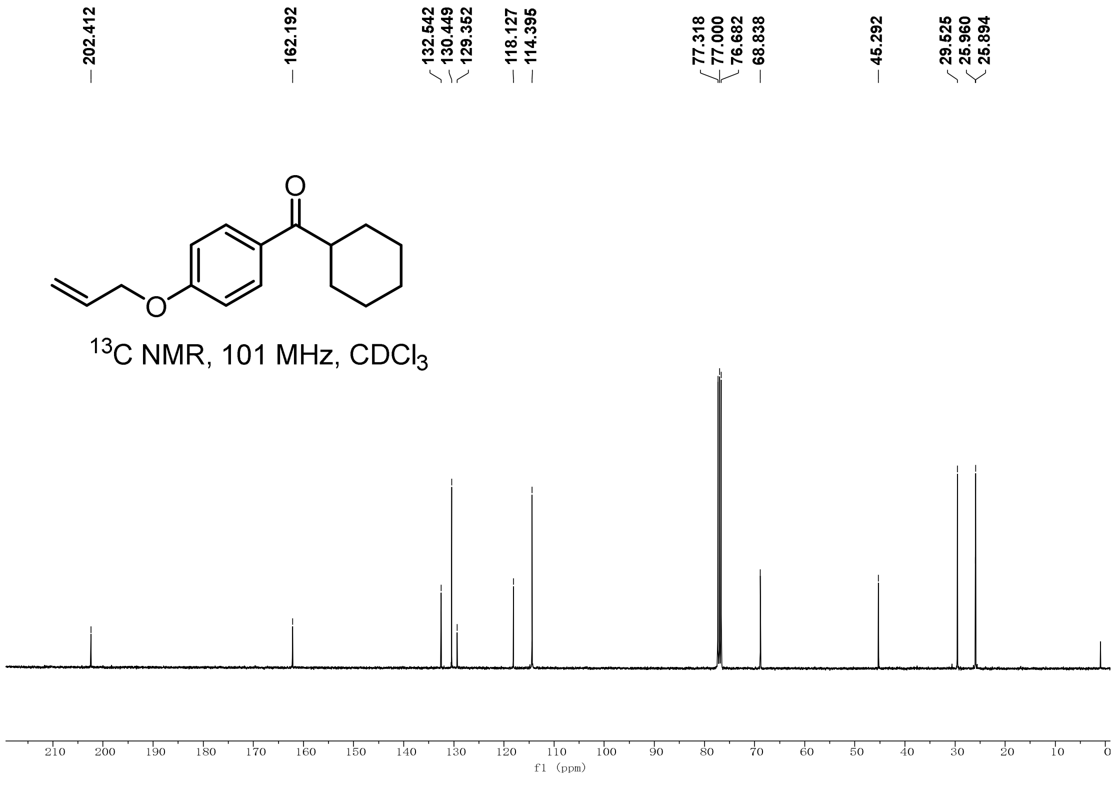
202.412 162.192 132.542 130.449 129.352 118.127 114.395 77.318 77.000 76.682 68.838 45.292 29.525 25.960 25.894
O
O
^{13}C NMR, 101 MHz, $CDCl_3$
210 200 190 180 170 160 150 140 130 120 110 100 90 80 70 60 50 40 30 20 10 0
f1 (ppm)

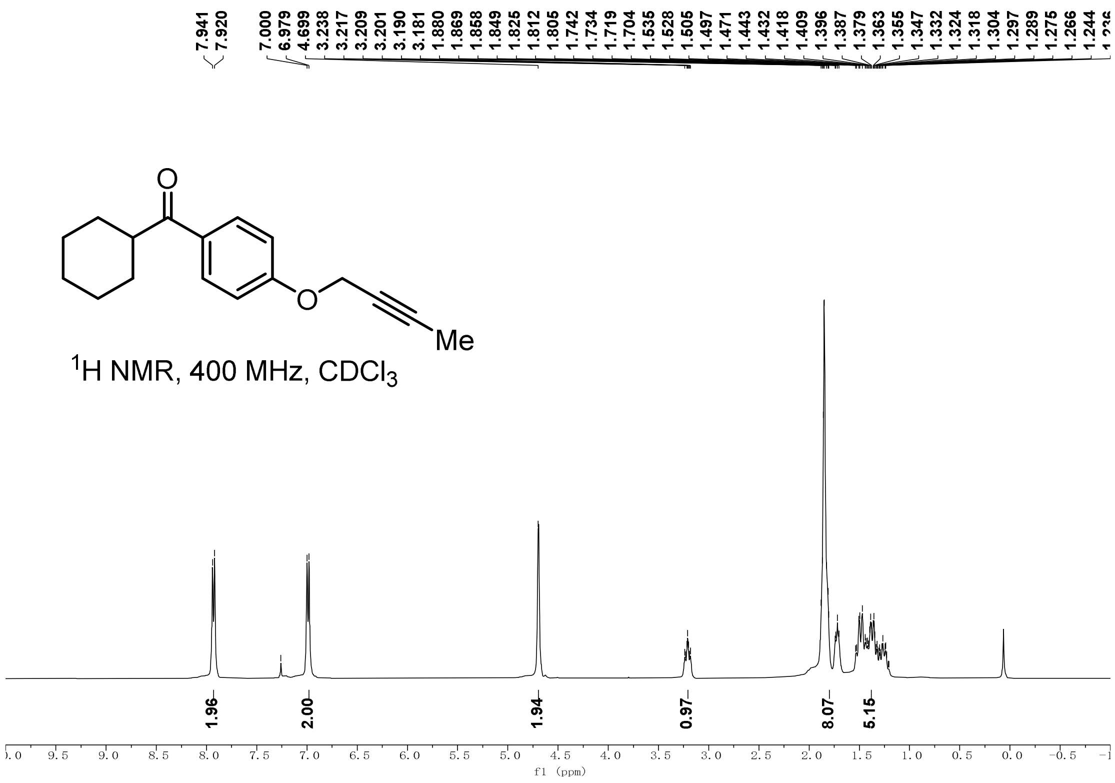

O
Me
^{1}H NMR, 400 MHz, $CDCl_3$
f1 (ppm)

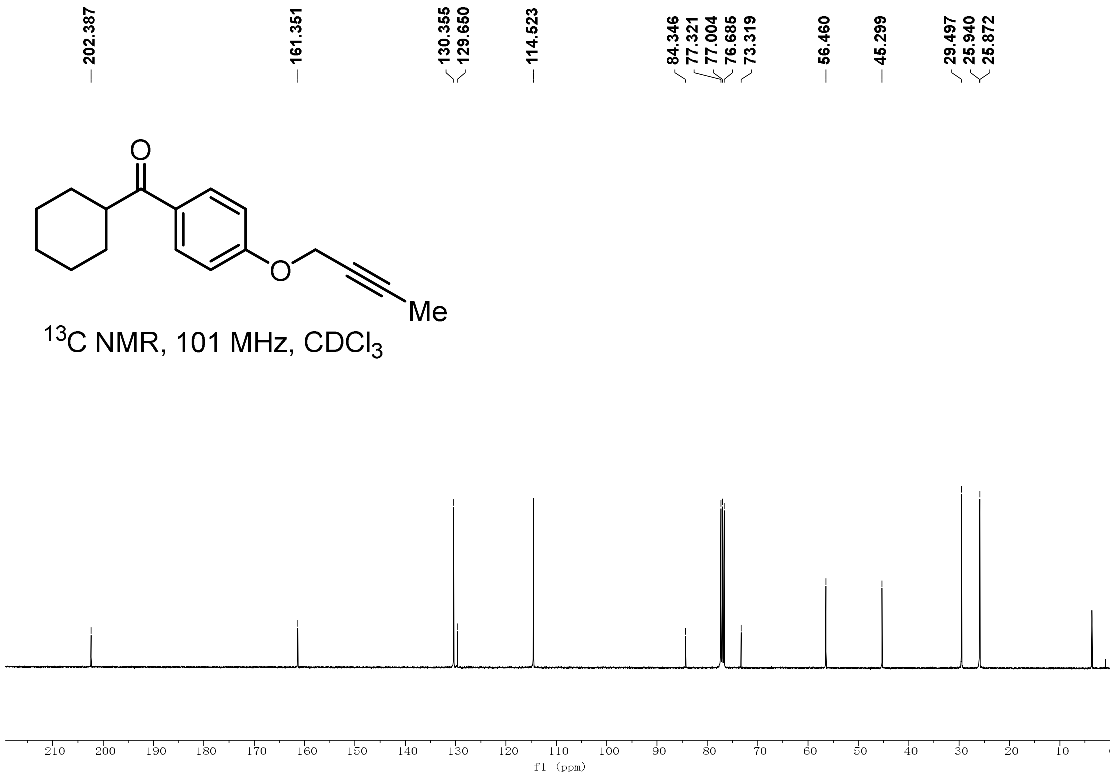

O
Me
^{13}C NMR, 101 MHz, $CDCl_3$
f1 (ppm)

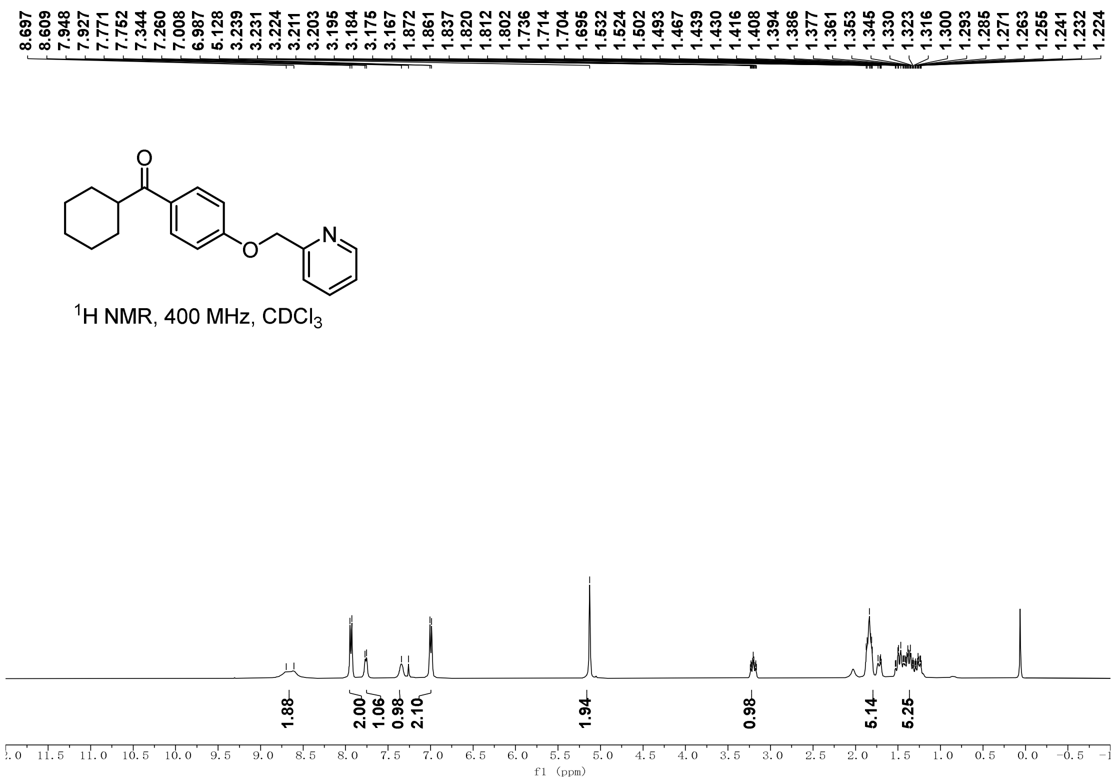
8.697 8.609 7.948 7.927 7.771 7.752 7.344 7.260 7.008 6.987 5.128 3.239 3.231 3.224 3.211 3.203 3.195 3.184 3.175 3.167 1.872 1.861 1.837 1.820 1.812 1.802 1.736 1.714 1.704 1.695 1.532 1.524 1.502 1.493 1.467 1.439 1.430 1.416 1.408 1.394 1.386 1.377 1.361 1.353 1.345 1.330 1.323 1.316 1.300 1.293 1.285 1.271 1.263 1.255 1.241 1.232 1.224
O
O
N
^{1}H NMR, 400 MHz, CDCl$_3$
1.88 2.00 1.06 0.98 2.10 1.94 0.98 5.14 5.25
f1 (ppm)

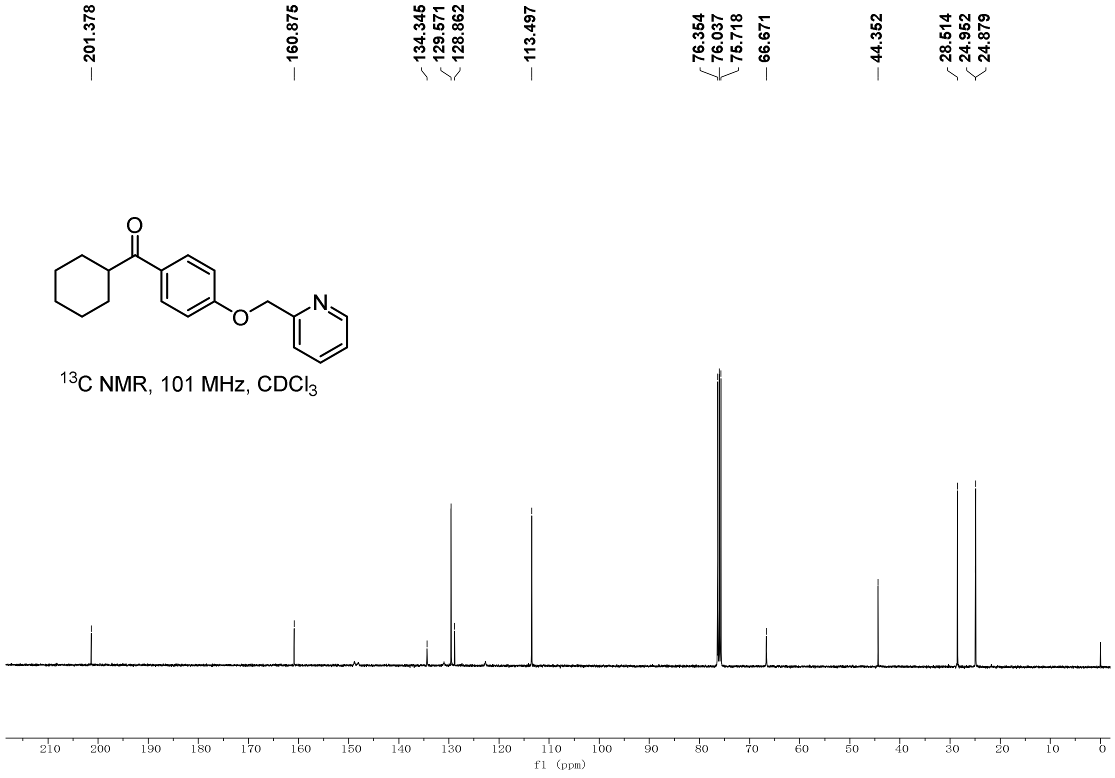
201.378 160.875 134.345 129.571 128.862 113.497 76.354 76.037 75.718 66.671 44.352 28.514 24.952 24.879
O
O
N
^{13}C NMR, 101 MHz, CDCl$_3$
f1 (ppm)

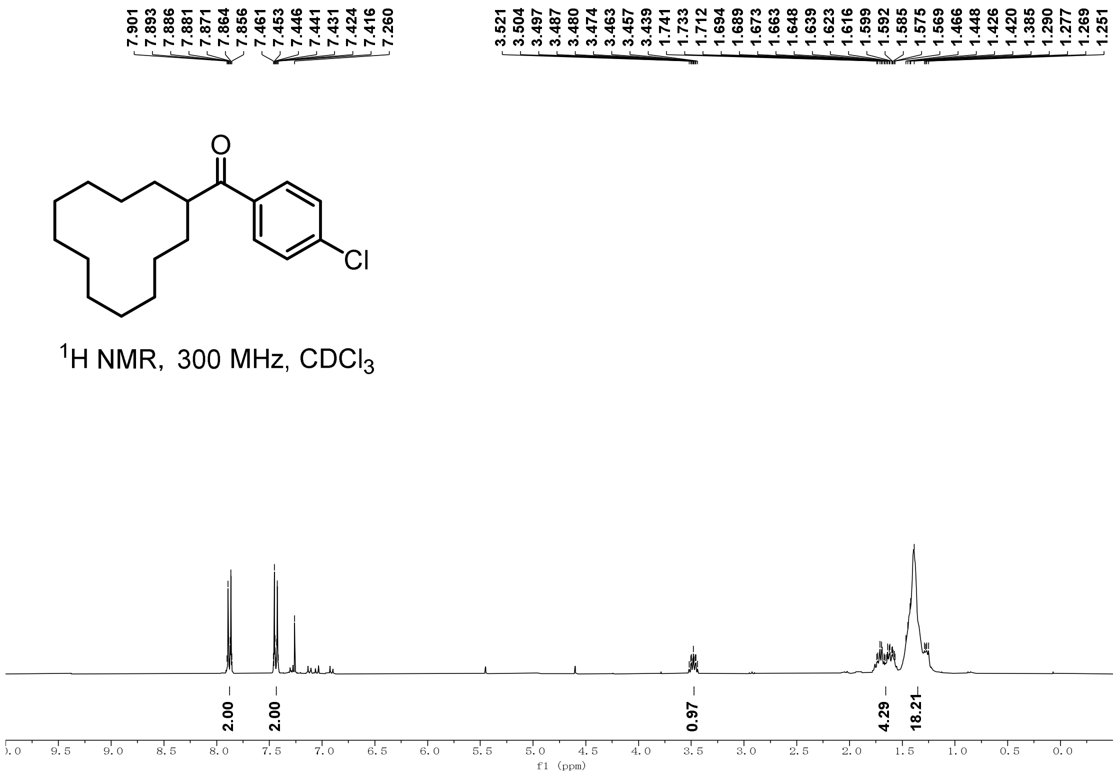
7.901 7.893 7.886 7.881 7.871 7.864 7.856 7.461 7.453 7.446 7.441 7.431 7.424 7.416 7.260
3.521 3.504 3.497 3.487 3.480 3.474 3.463 3.457 3.439 1.741 1.733 1.712 1.694 1.689 1.673 1.663 1.648 1.639 1.623 1.616 1.599 1.592 1.585 1.575 1.569 1.466 1.448 1.426 1.420 1.385 1.290 1.277 1.269 1.251
O
Cl
^{1}H NMR, 300 MHz, CDCl$_3$
2.00
2.00
0.97
4.29
18.21
f1 (ppm)

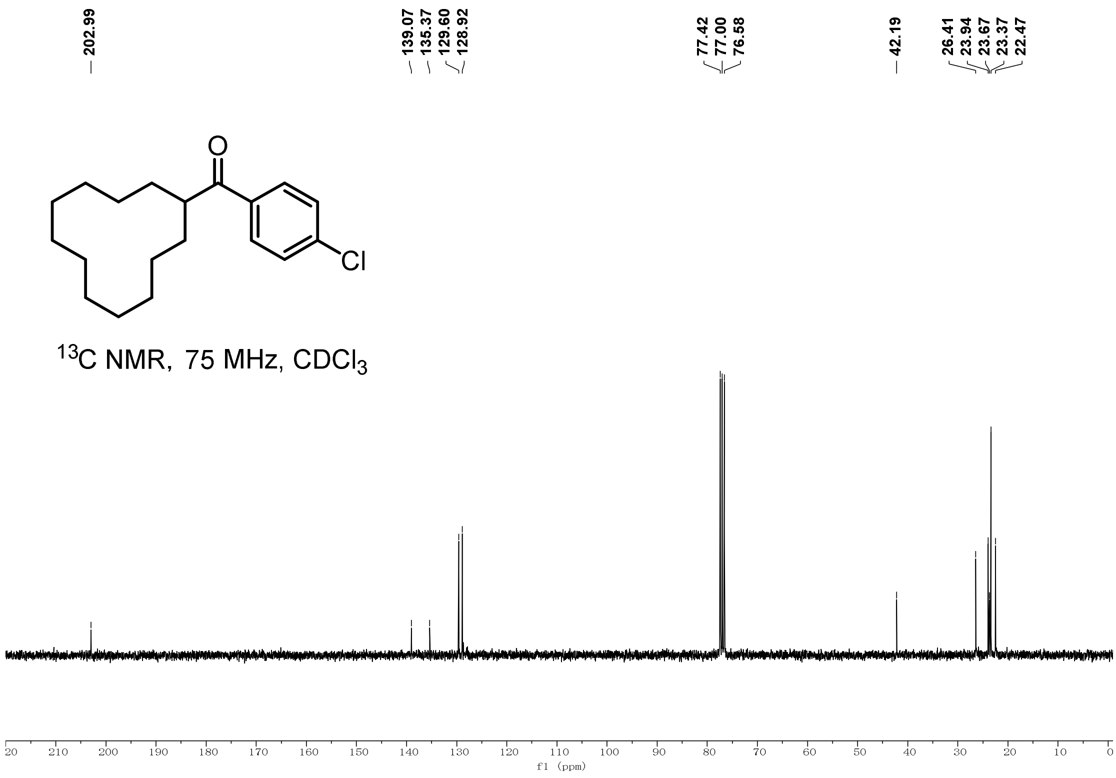
202.99
139.07 135.37 129.60 128.92
77.42 77.00 76.58
42.19
26.41 23.94 23.67 23.37 22.47
O
Cl
^{13}C NMR, 75 MHz, CDCl$_3$
f1 (ppm)

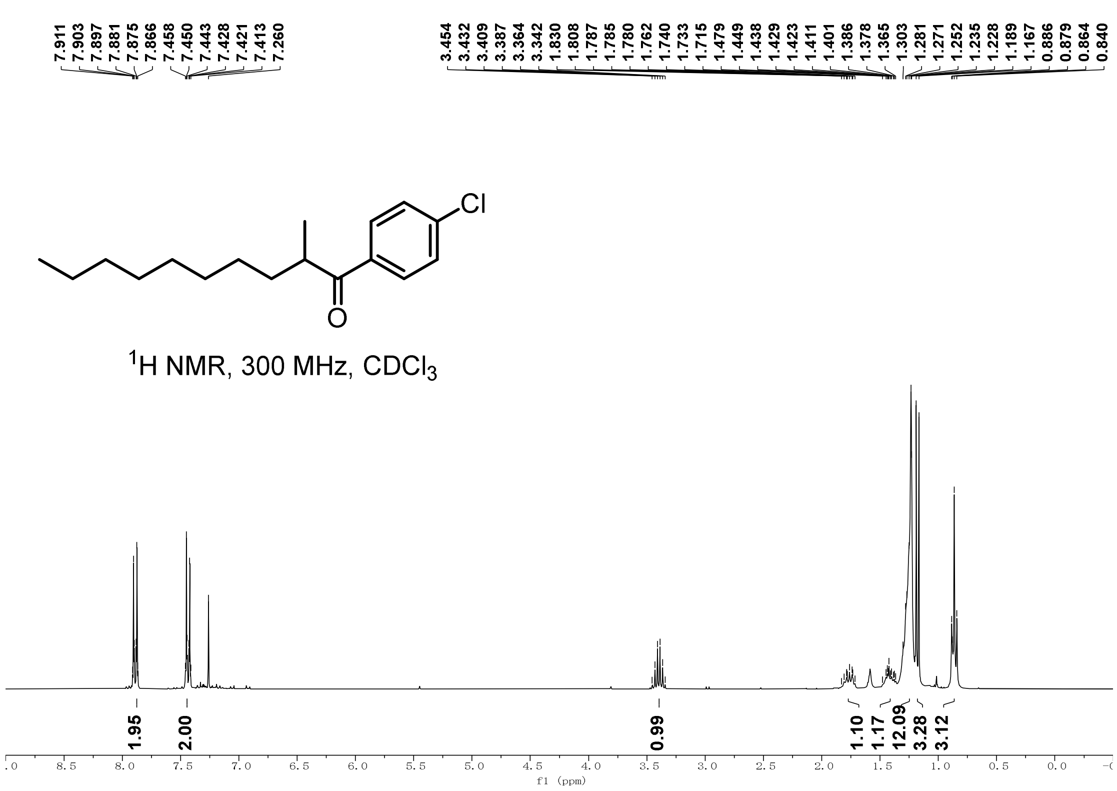
7.911 7.903 7.897 7.881 7.875 7.866 7.458 7.450 7.443 7.428 7.421 7.413 7.260
3.454 3.432 3.409 3.387 3.364 3.342 1.830 1.808 1.787 1.785 1.780 1.762 1.740 1.733 1.715 1.479 1.449 1.438 1.429 1.423 1.411 1.401 1.386 1.378 1.365 1.303 1.281 1.271 1.252 1.235 1.228 1.189 1.167 0.886 0.879 0.864 0.840
Cl
O
1H NMR, 300 MHz, CDCl3
1.95 2.00 0.99 1.10 1.17 12.09 3.28 3.12
f1 (ppm)

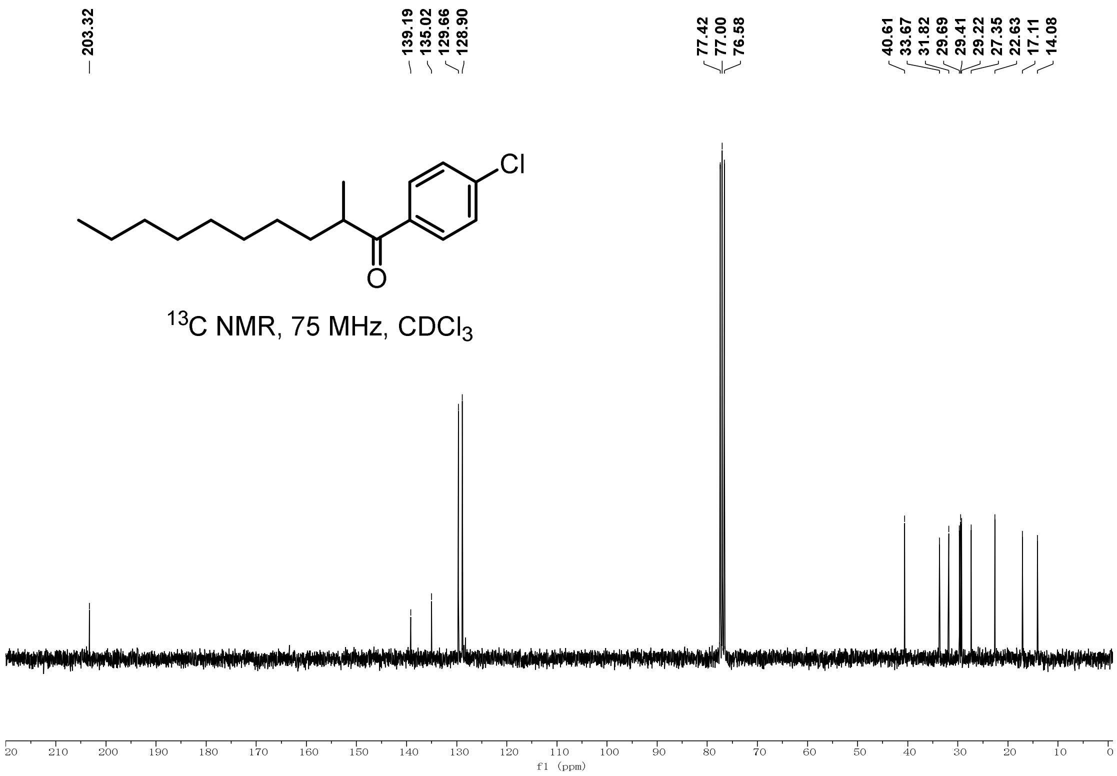
203.32
139.19 135.02 129.66 128.90
77.42 77.00 76.58
40.61 33.67 31.82 29.69 29.41 29.22 27.35 22.63 17.11 14.08
Cl
O
13C NMR, 75 MHz, CDCl3
f1 (ppm)

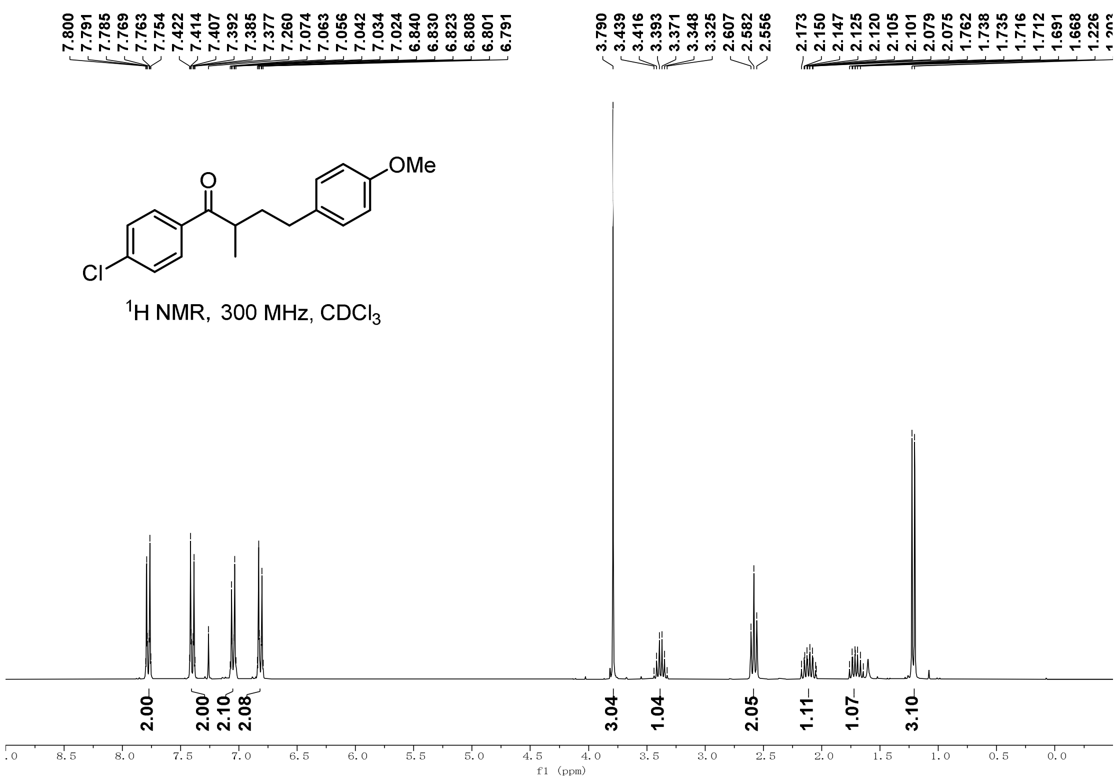

OMe
O
Cl
^{1}H NMR, 300 MHz, CDCl$_3$
f1 (ppm)

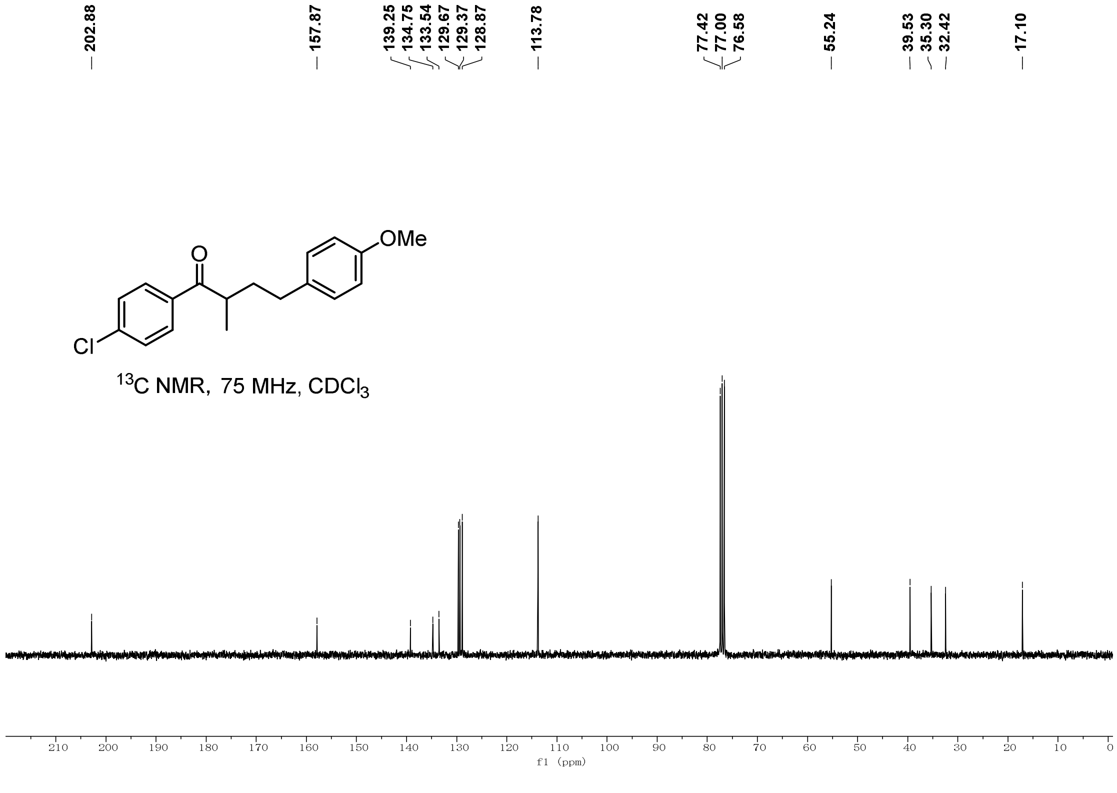

202.88
157.87
139.25
134.75
133.54
129.67
129.37
128.87
113.78
77.42
77.00
76.58
55.24
39.53
35.30
32.42
17.10
OMe
O
Cl
^{13}C NMR, 75 MHz, CDCl$_3$
f1 (ppm)

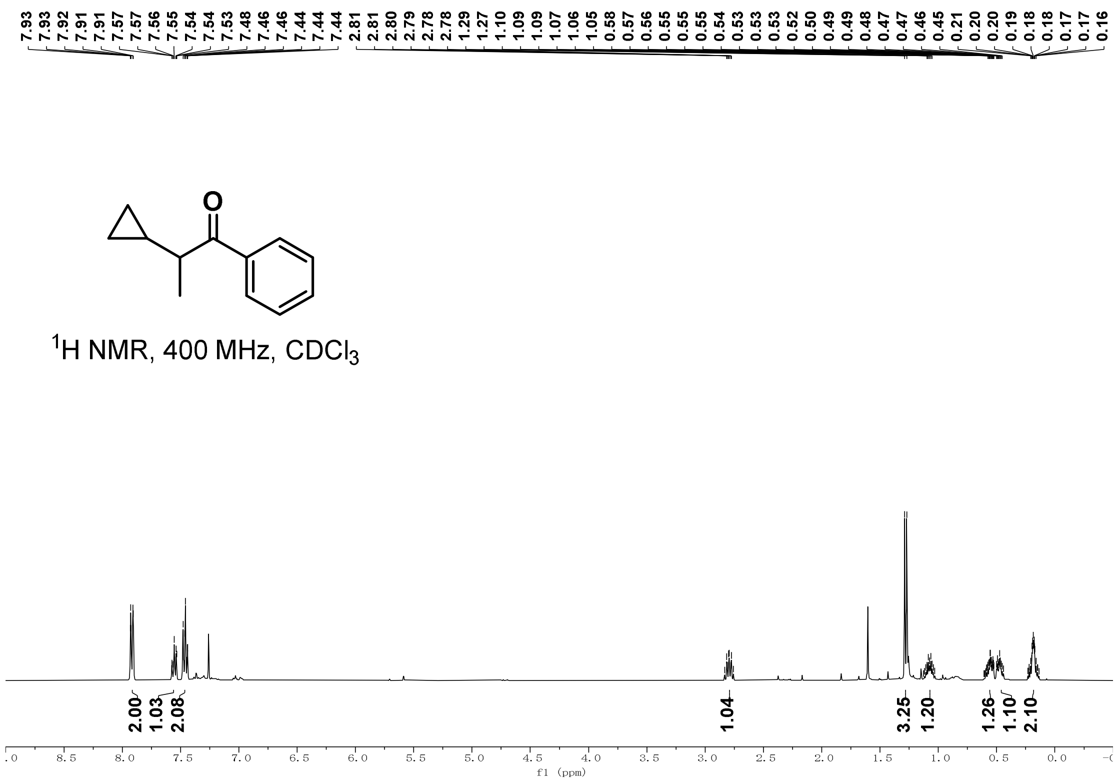

^{1}H NMR, 400 MHz, $CDCl_3$

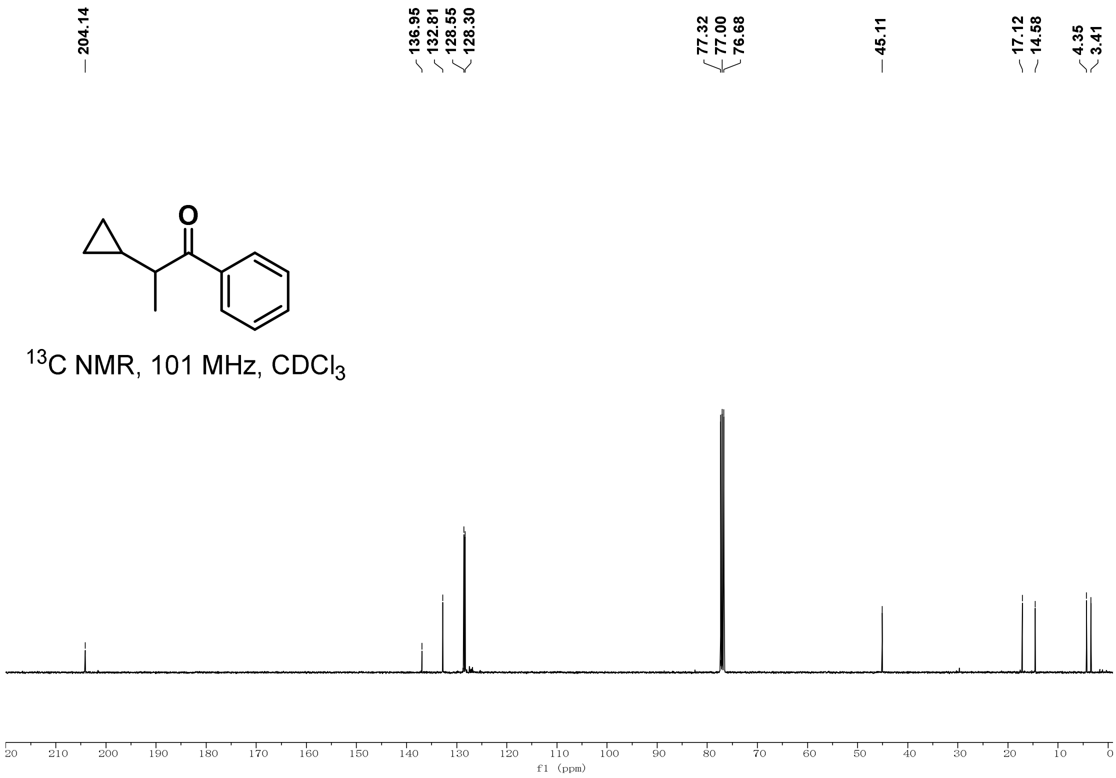

^{13}C NMR, 101 MHz, $CDCl_3$

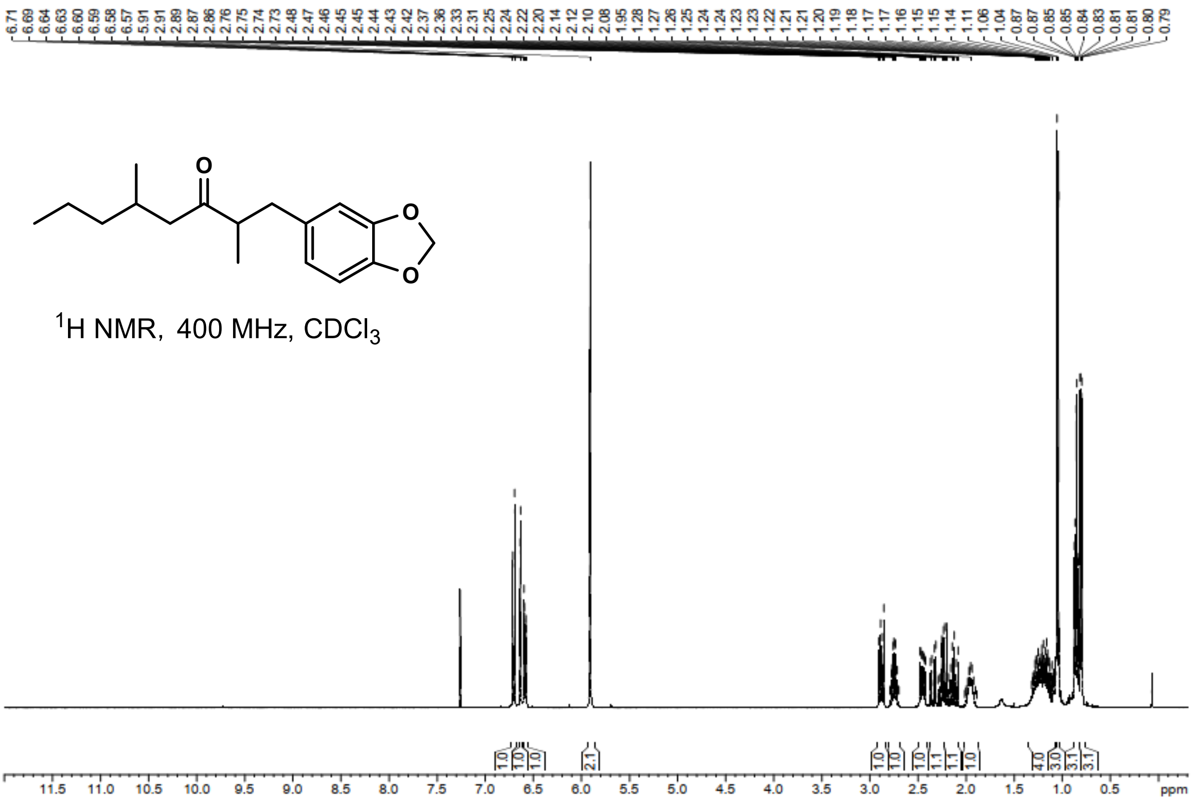
O
O
O
^{1}H NMR, 400 MHz, CDCl$_3$
ppm

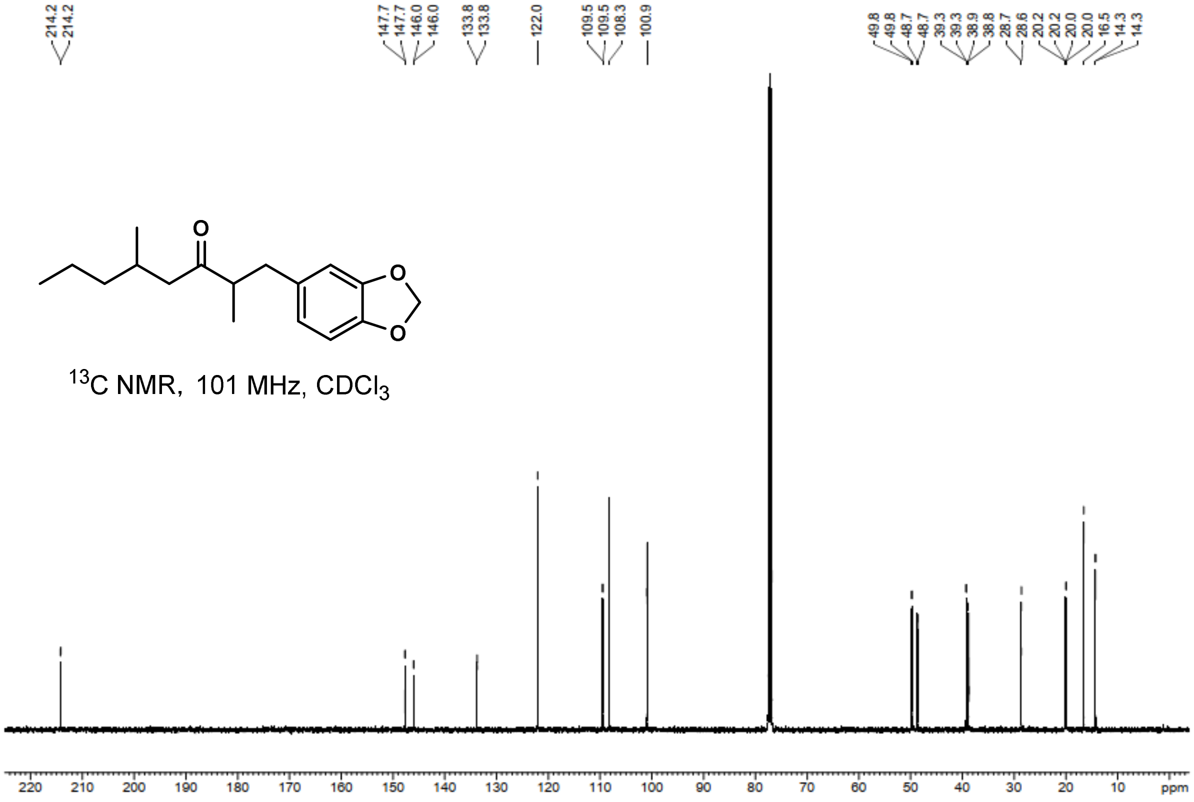
O
O
O
^{13}C NMR, 101 MHz, CDCl$_3$
ppm

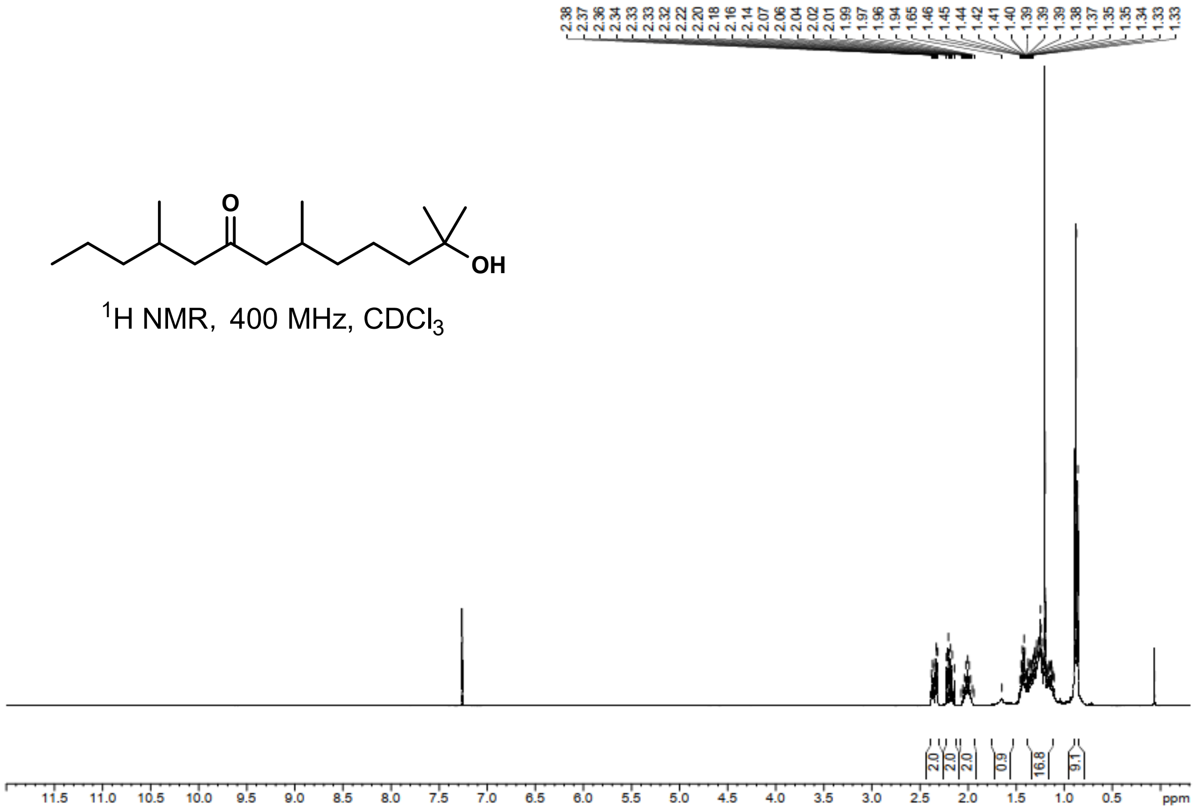
O
OH
1H NMR, 400 MHz, CDCl3
2.38 2.37 2.36 2.34 2.33 2.33 2.32 2.22 2.20 2.18 2.16 2.14 2.07 2.06 2.04 2.02 2.01 1.99 1.97 1.96 1.94 1.65 1.46 1.45 1.44 1.42 1.41 1.40 1.39 1.39 1.39 1.38 1.37 1.35 1.35 1.34 1.33 1.33
2.0 2.0 2.0 0.9 16.8 9.1
11.5 11.0 10.5 10.0 9.5 9.0 8.5 8.0 7.5 7.0 6.5 6.0 5.5 5.0 4.5 4.0 3.5 3.0 2.5 2.0 1.5 1.0 0.5 ppm

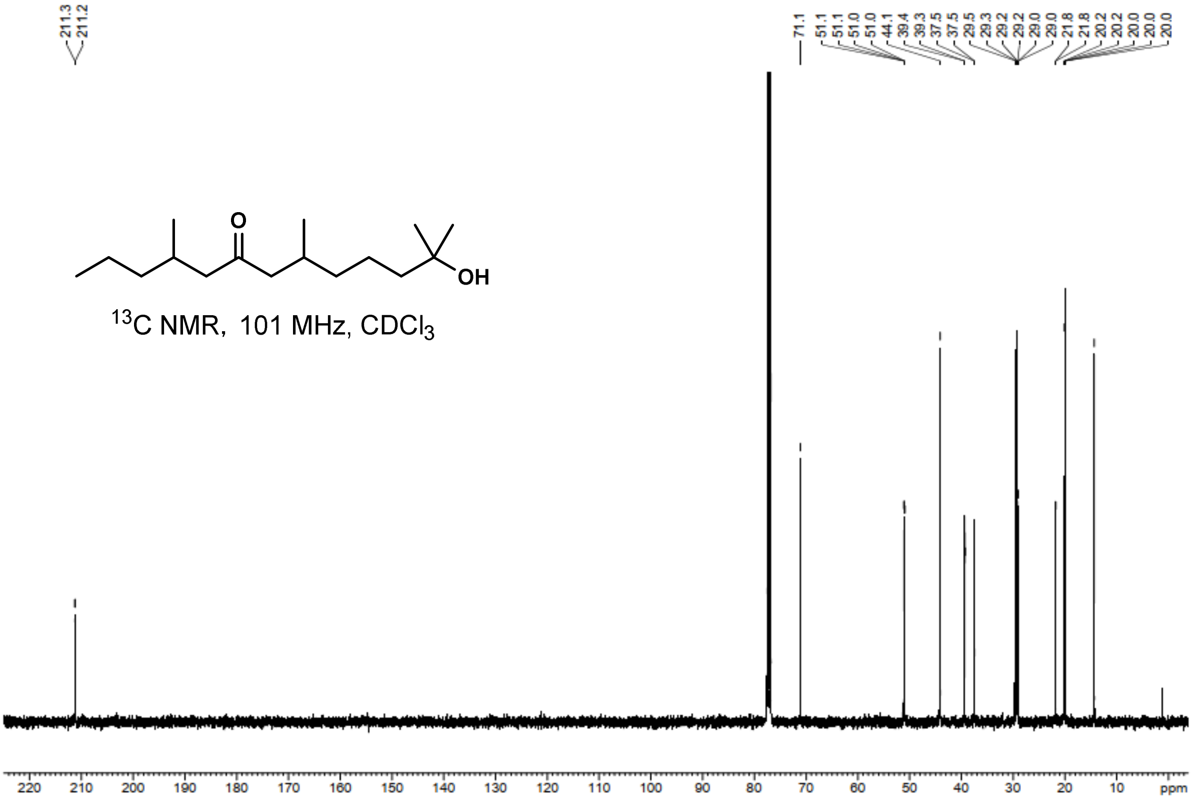
211.3 211.2
71.1 51.1 51.1 51.0 51.0 44.1 39.4 39.3 37.5 37.5 29.5 29.3 29.2 29.2 29.0 29.0 21.8 21.8 20.2 20.2 20.0 20.0 20.0
O
OH
13C NMR, 101 MHz, CDCl3
220 210 200 190 180 170 160 150 140 130 120 110 100 90 80 70 60 50 40 30 20 10 ppm

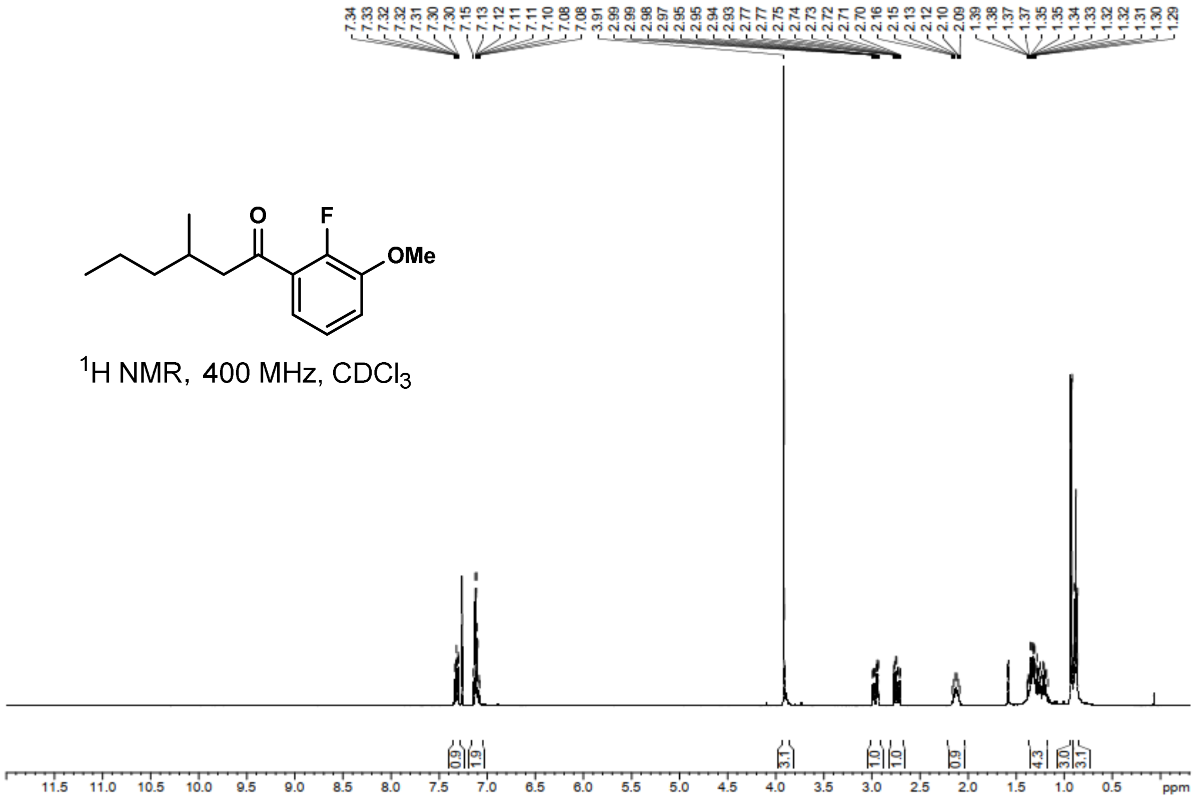
O
F
OMe
1H NMR, 400 MHz, CDCl3

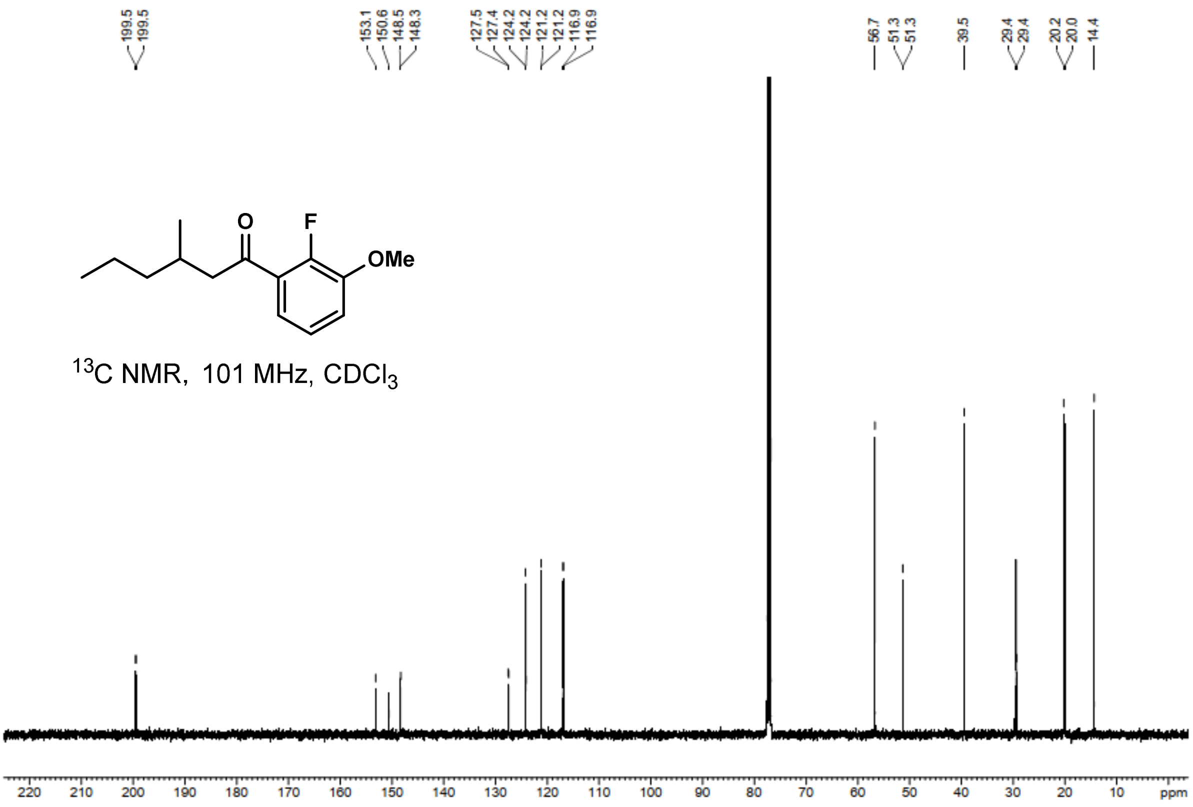
O
F
OMe
13C NMR, 101 MHz, CDCl3

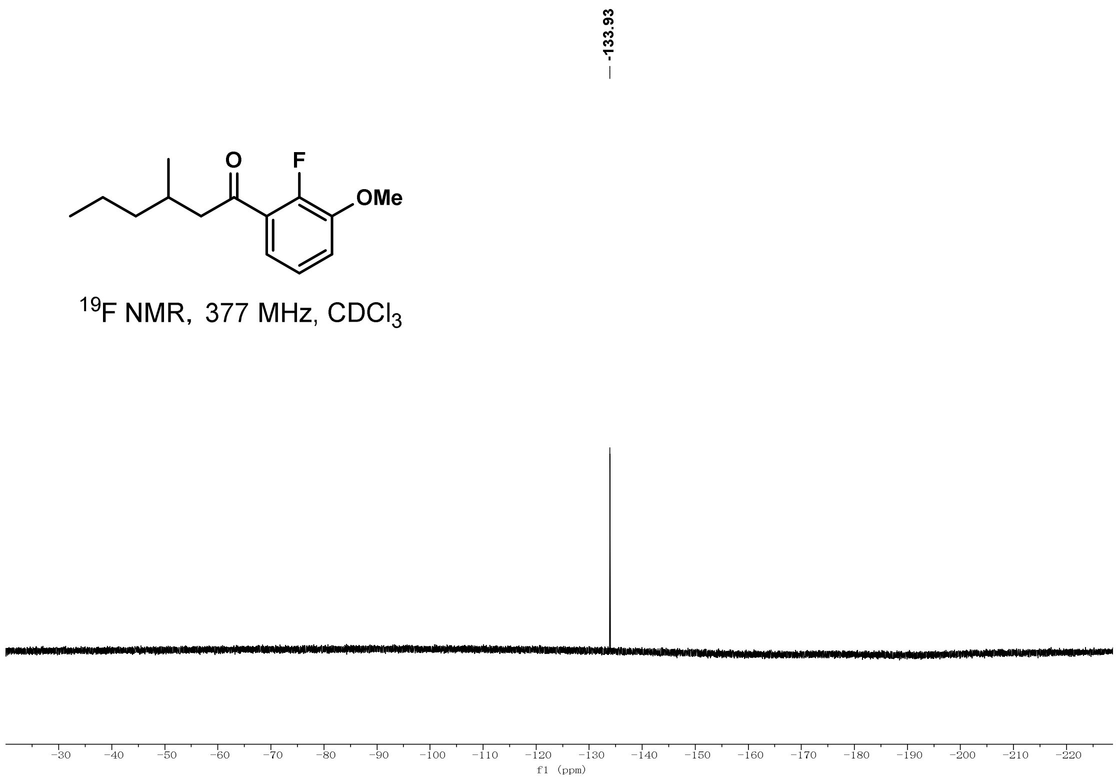
-133.93
O
F
OMe
^{19}F NMR, 377 MHz, $CDCl_3$
−30 −40 −50 −60 −70 −80 −90 −100 −110 −120 −130 −140 −150 −160 −170 −180 −190 −200 −210 −220
f1 (ppm)

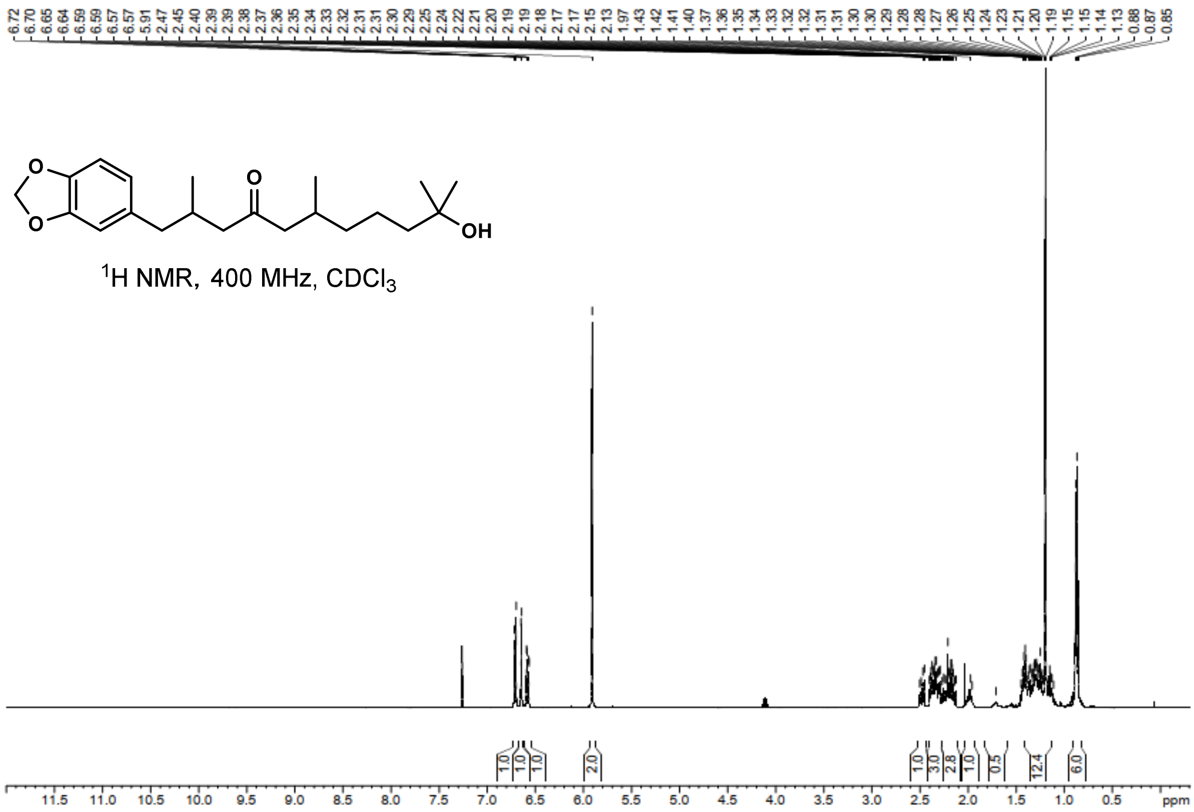
O
O
O
OH
1H NMR, 400 MHz, CDCl3
ppm

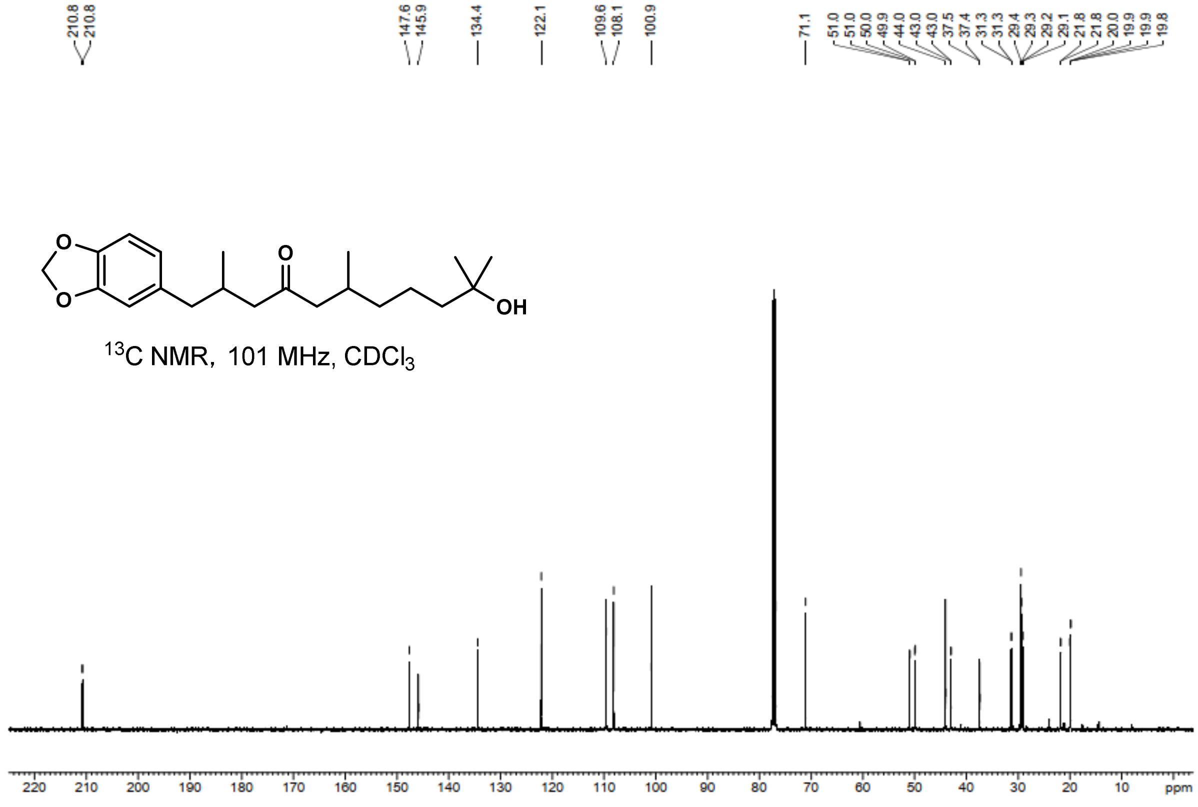
O
O
O
OH
13C NMR, 101 MHz, CDCl3
ppm

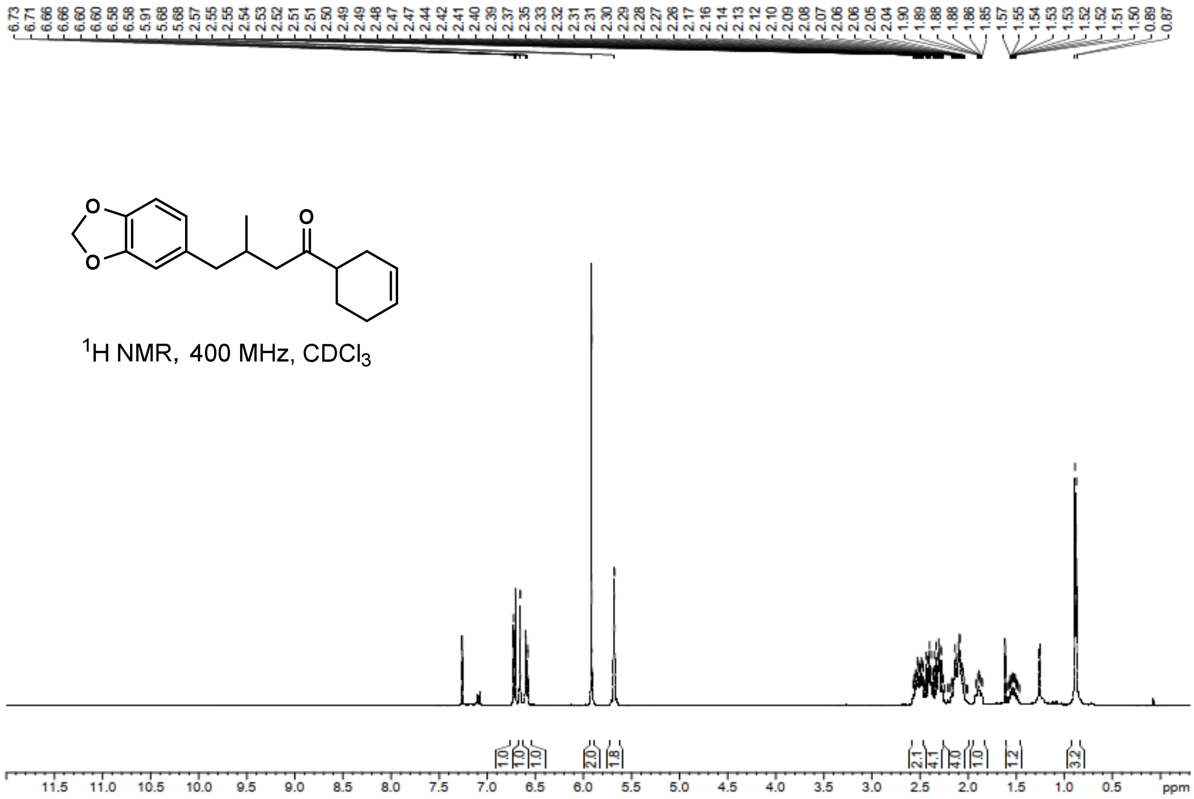
6.73 6.71 6.66 6.66 6.60 6.60 6.58 6.58 5.91 5.68 5.68 2.57 2.55 2.55 2.54 2.53 2.52 2.51 2.51 2.50 2.49 2.49 2.48 2.47 2.47 2.44 2.42 2.41 2.40 2.39 2.37 2.35 2.33 2.32 2.31 2.30 2.29 2.28 2.27 2.26 2.17 2.16 2.14 2.13 2.12 2.10 2.09 2.08 2.07 2.06 2.06 2.05 2.04 1.90 1.89 1.88 1.88 1.86 1.85 1.57 1.55 1.54 1.53 1.53 1.52 1.52 1.51 1.50 0.89 0.87
O
O
O
^{1}H NMR, 400 MHz, CDCl$_3$
1.0 1.0 1.0 2.0 1.8 2.1 4.1 4.0 1.0 1.2 3.2
ppm

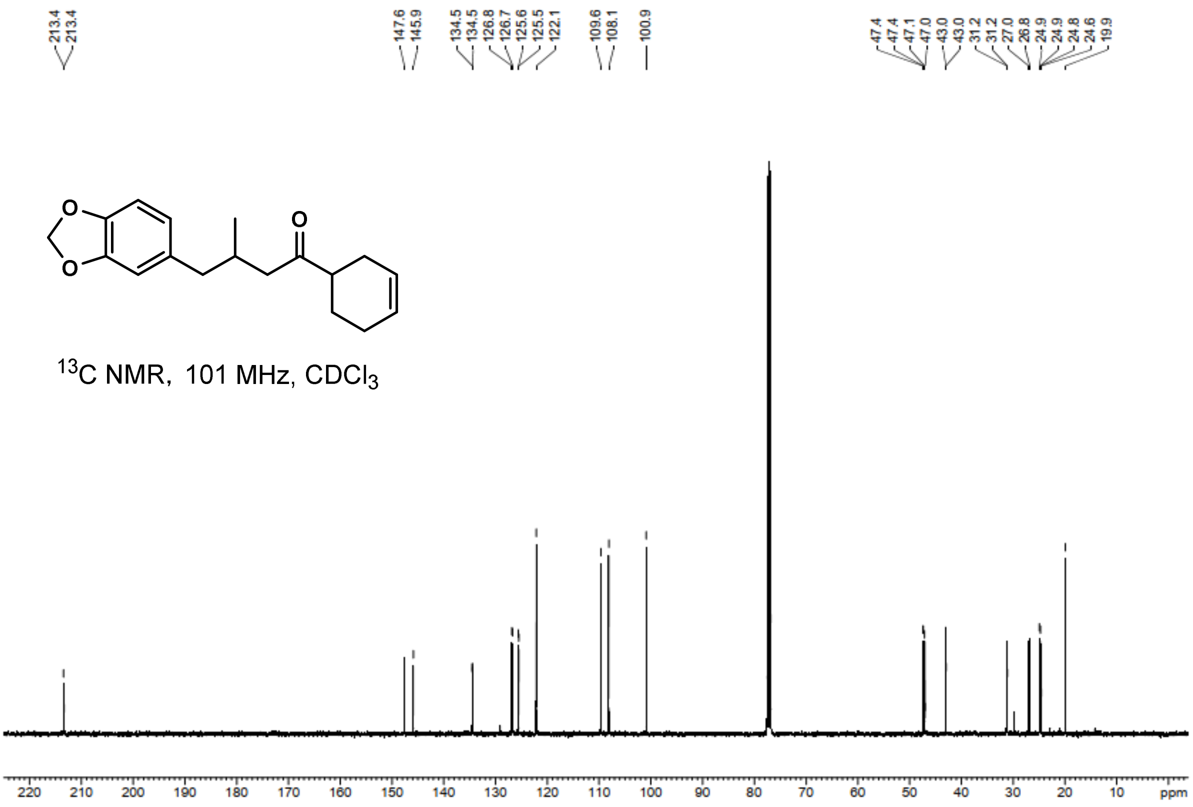
213.4 213.4 147.6 145.9 134.5 134.5 126.8 126.7 125.6 125.5 122.1 109.6 108.1 100.9 47.4 47.4 47.1 47.0 43.0 43.0 31.2 31.2 27.0 26.8 24.9 24.9 24.8 24.6 19.9
O
O
O
^{13}C NMR, 101 MHz, CDCl$_3$
ppm

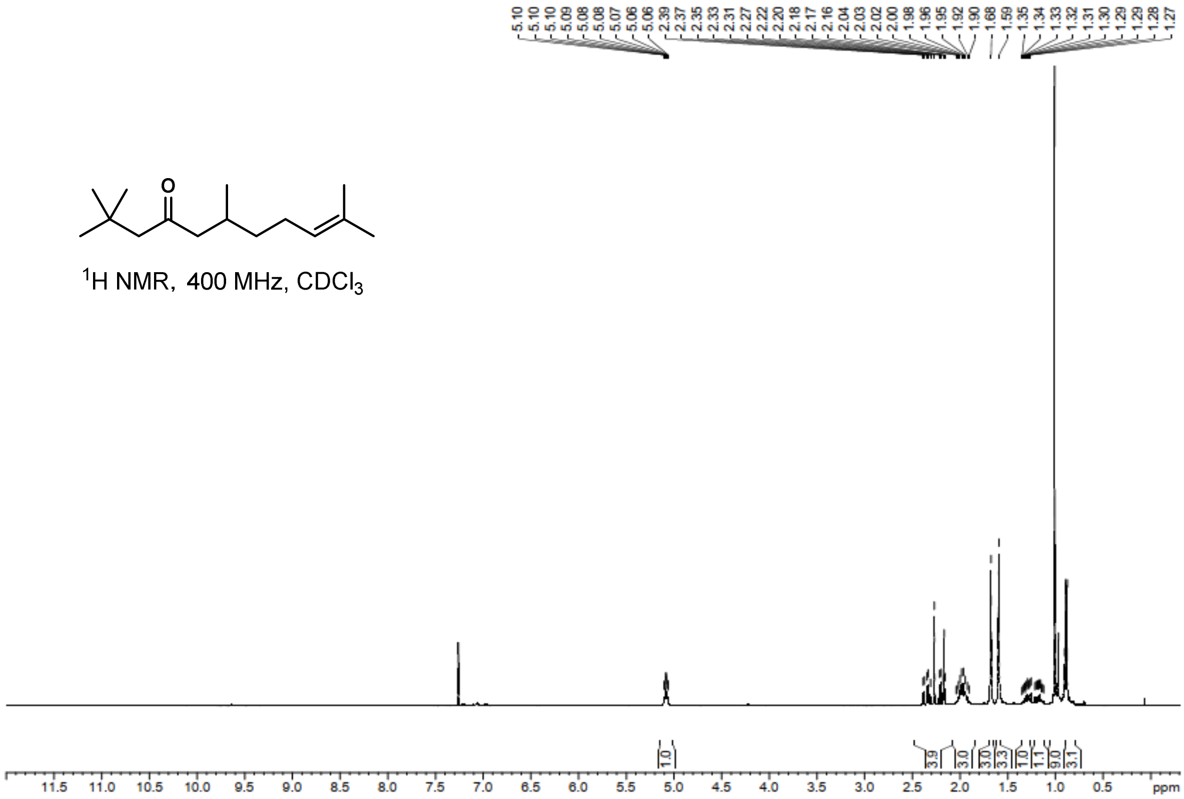
O
1H NMR, 400 MHz, CDCl3
5.10 5.10 5.10 5.09 5.08 5.08 5.07 5.06 5.06 2.39 2.37 2.35 2.33 2.31 2.27 2.22 2.20 2.18 2.17 2.16 2.04 2.03 2.02 2.00 1.98 1.96 1.95 1.92 1.90 1.68 1.59 1.35 1.34 1.33 1.32 1.31 1.30 1.29 1.29 1.28 1.27
1.0 3.9 3.0 3.0 3.3 1.0 1.1 9.0 3.1
11.5 11.0 10.5 10.0 9.5 9.0 8.5 8.0 7.5 7.0 6.5 6.0 5.5 5.0 4.5 4.0 3.5 3.0 2.5 2.0 1.5 1.0 0.5 ppm

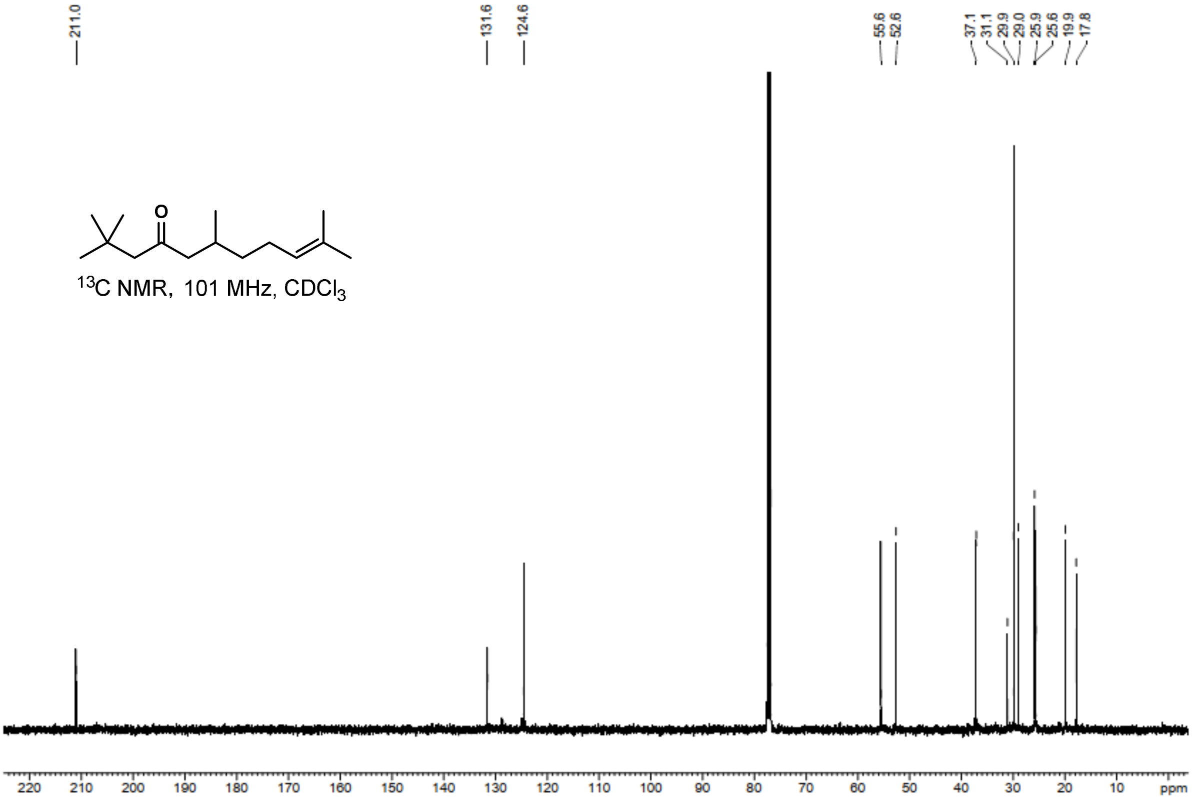
O
13C NMR, 101 MHz, CDCl3
211.0
131.6
124.6
55.6 52.6
37.1 31.1 29.9 29.0 25.9 25.6 19.9 17.8
220 210 200 190 180 170 160 150 140 130 120 110 100 90 80 70 60 50 40 30 20 10 ppm

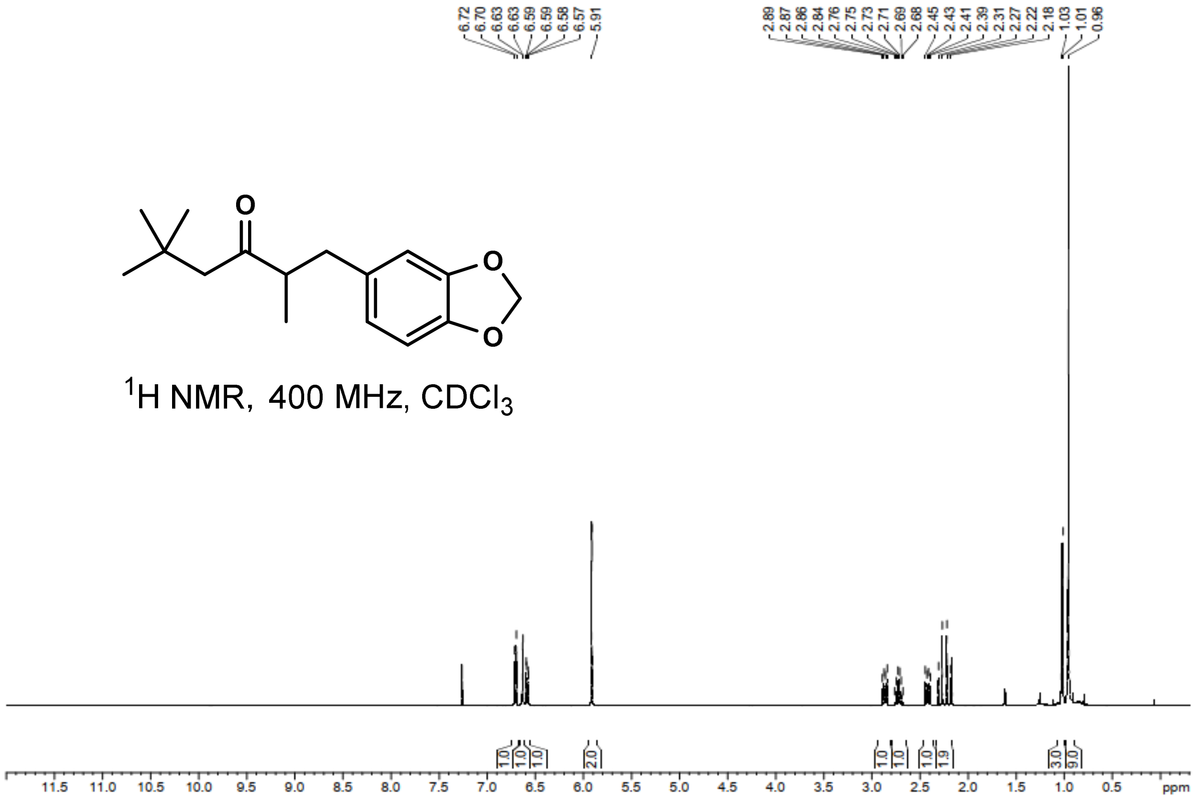

1H NMR, 400 MHz, CDCl3
6.72 6.70 6.63 6.63 6.59 6.59 6.58 6.57 5.91
2.89 2.87 2.86 2.84 2.76 2.75 2.73 2.71 2.69 2.68 2.45 2.43 2.41 2.39 2.31 2.27 2.22 2.18 1.03 1.01 0.96
1.0 1.0 1.0 2.0 1.0 1.0 1.0 1.9 3.0 9.0
ppm

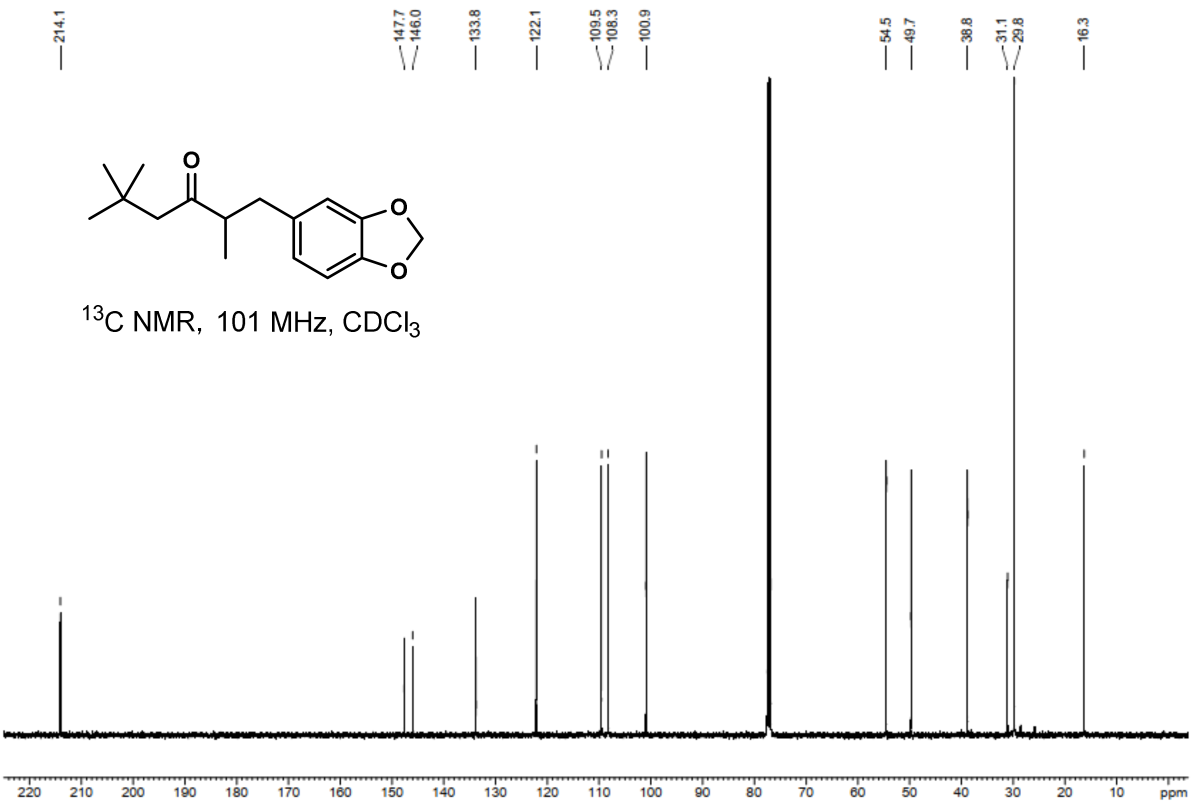

13C NMR, 101 MHz, CDCl3
214.1 147.7 146.0 133.8 122.1 109.5 108.3 100.9 54.5 49.7 38.8 31.1 29.8 16.3
ppm

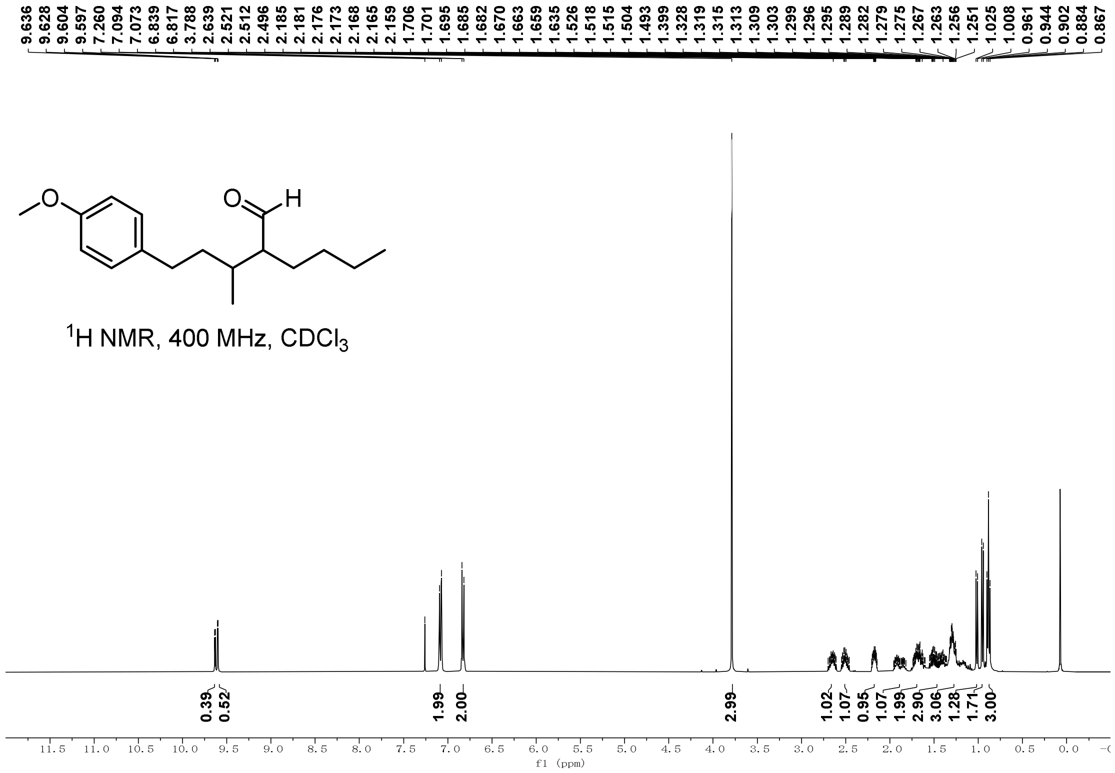
O
O
H
^{1}H NMR, 400 MHz, $CDCl_3$
f1 (ppm)

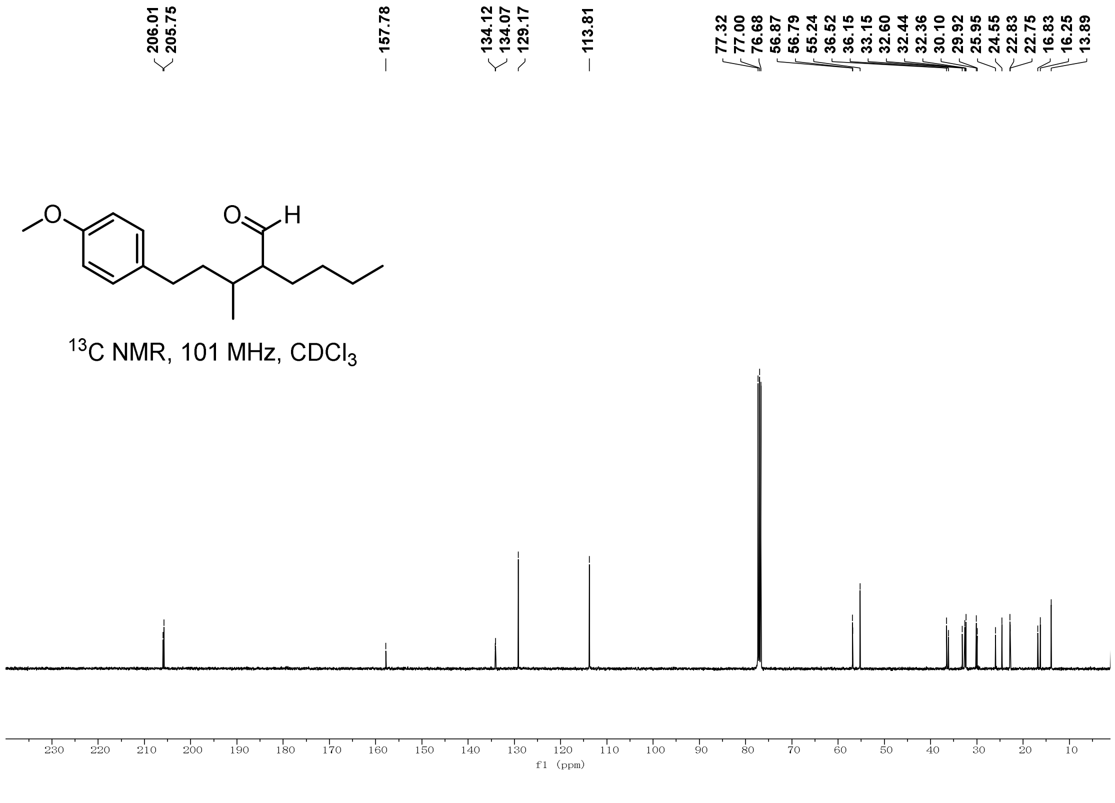
O
O
H
^{13}C NMR, 101 MHz, $CDCl_3$
f1 (ppm)

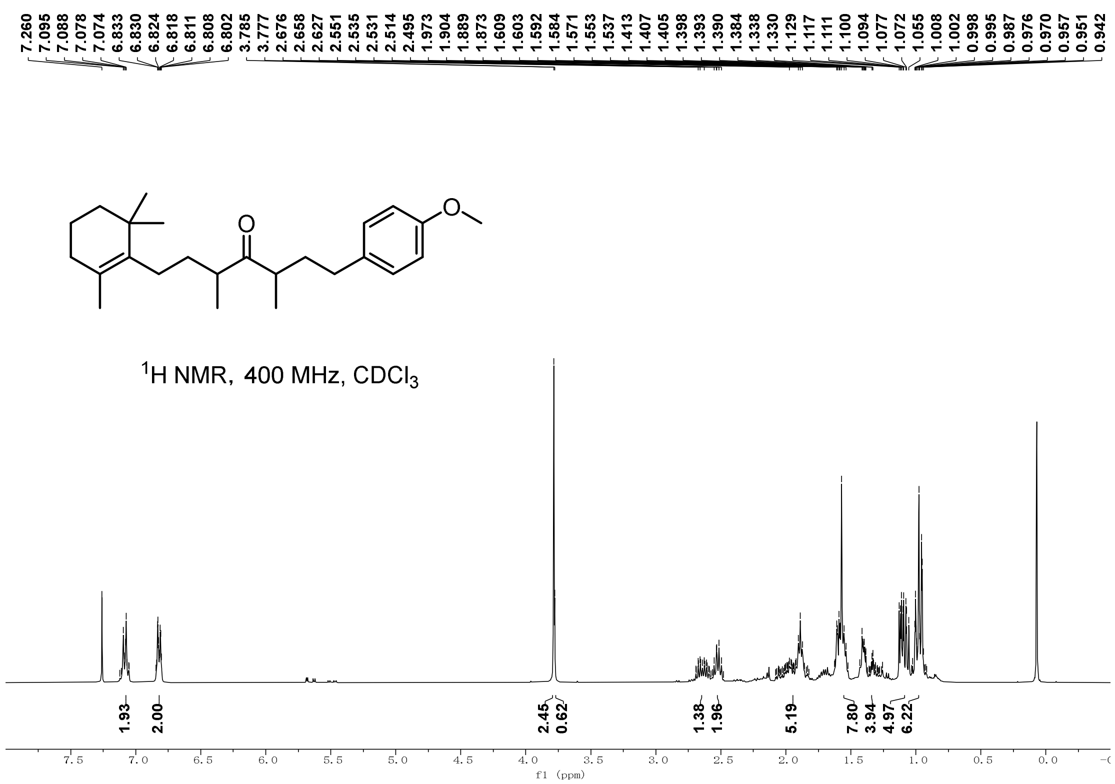
7.260 7.095 7.088 7.078 7.074 6.833 6.830 6.824 6.818 6.811 6.808 6.802 3.785 3.777 2.676 2.658 2.627 2.551 2.535 2.531 2.514 2.495 1.973 1.904 1.889 1.873 1.609 1.603 1.592 1.584 1.571 1.553 1.537 1.413 1.407 1.405 1.398 1.393 1.390 1.384 1.338 1.330 1.129 1.117 1.111 1.100 1.094 1.077 1.072 1.055 1.008 1.002 0.998 0.995 0.987 0.976 0.970 0.957 0.951 0.942
O
1H NMR, 400 MHz, CDCl3
1.93 2.00 2.45 0.62 1.38 1.96 5.19 7.80 3.94 4.97 6.22
f1 (ppm)

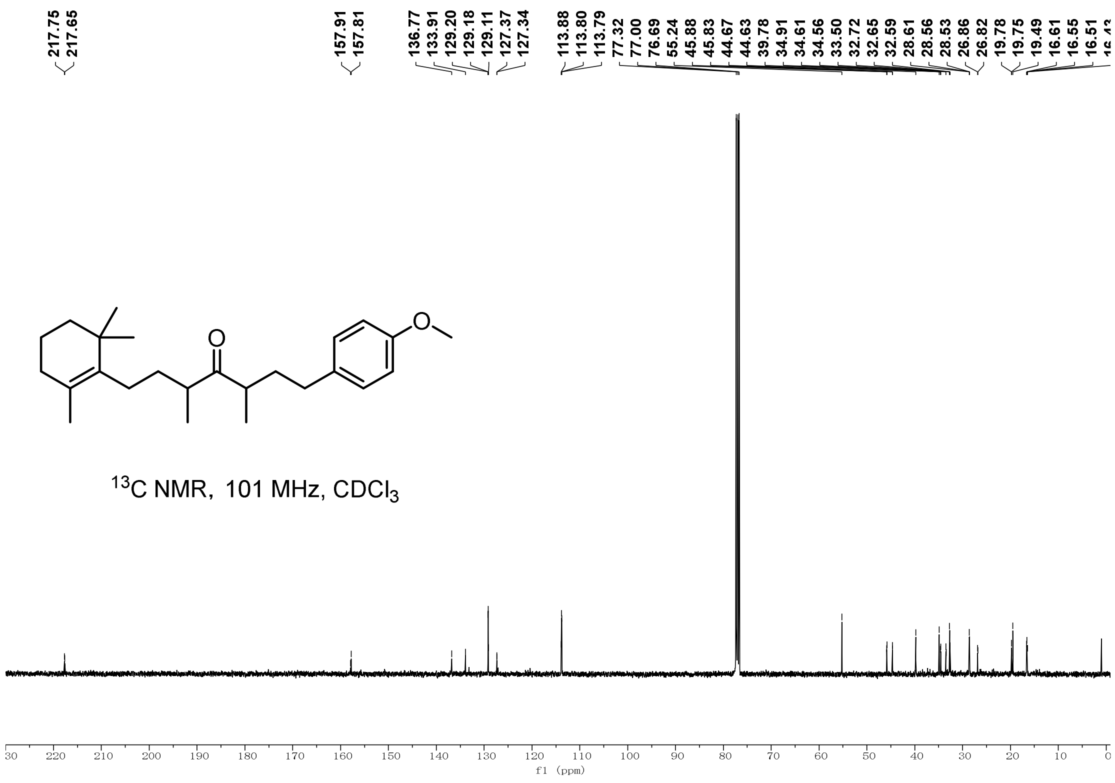
217.75 217.65 157.91 157.81 136.77 133.91 129.20 129.18 129.11 127.37 127.34 113.88 113.80 113.79 77.32 77.00 76.69 55.24 45.88 45.83 44.67 44.63 39.78 34.91 34.61 34.56 33.50 32.72 32.65 32.59 28.61 28.56 28.53 26.86 26.82 19.78 19.75 19.49 16.61 16.55 16.51
O
13C NMR, 101 MHz, CDCl3
f1 (ppm)

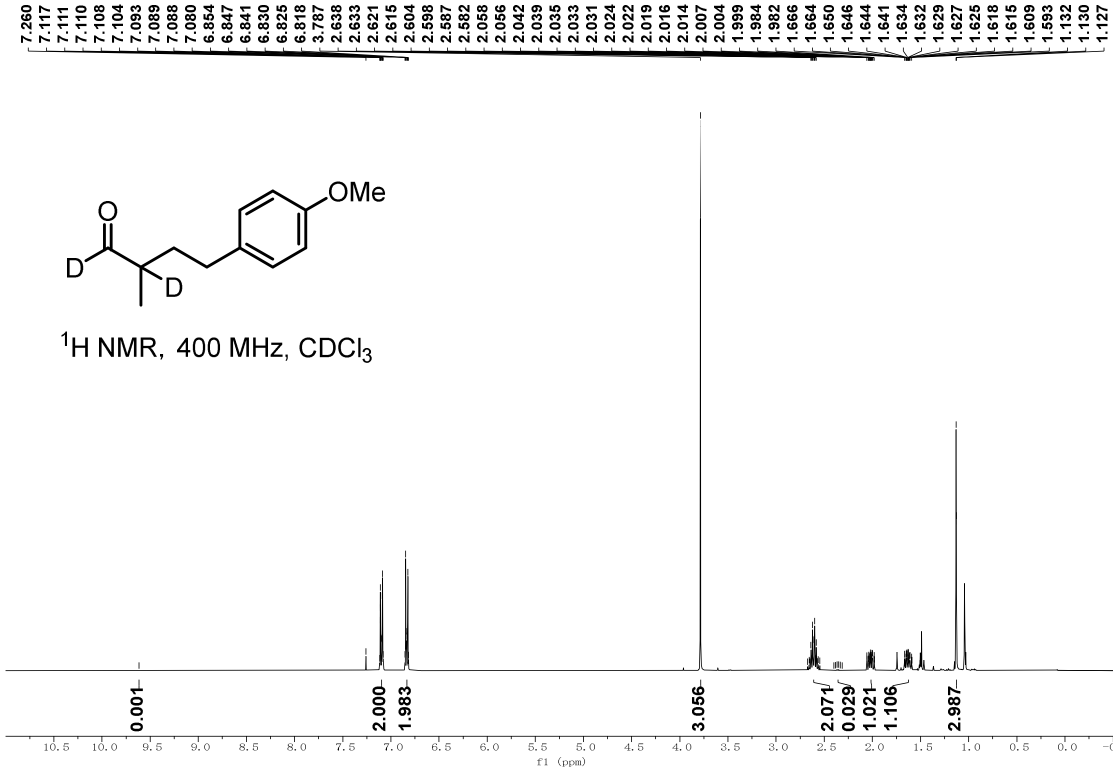
OMe
O
D
D
1H NMR, 400 MHz, CDCl3
0.001
2.000
1.983
3.056
2.071
0.029
1.021
1.106
2.987
f1 (ppm)

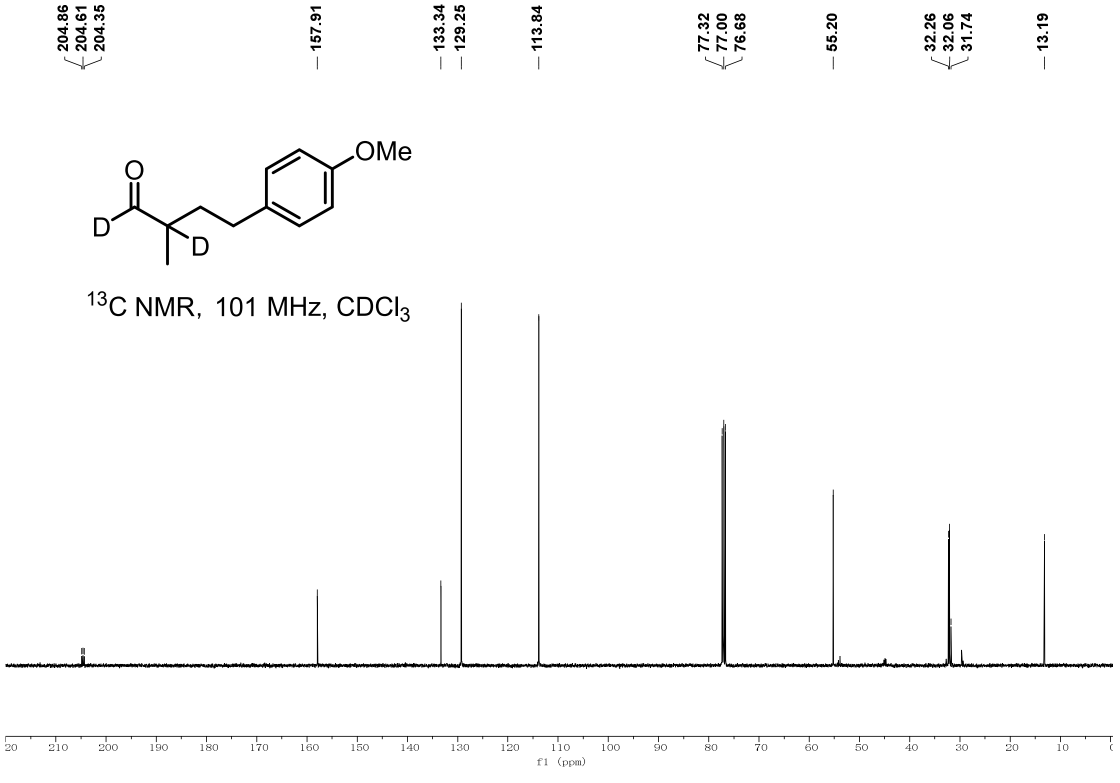
204.86
204.61
204.35
157.91
133.34
129.25
113.84
77.32
77.00
76.68
55.20
32.26
32.06
31.74
13.19
OMe
O
D
D
13C NMR, 101 MHz, CDCl3
f1 (ppm)

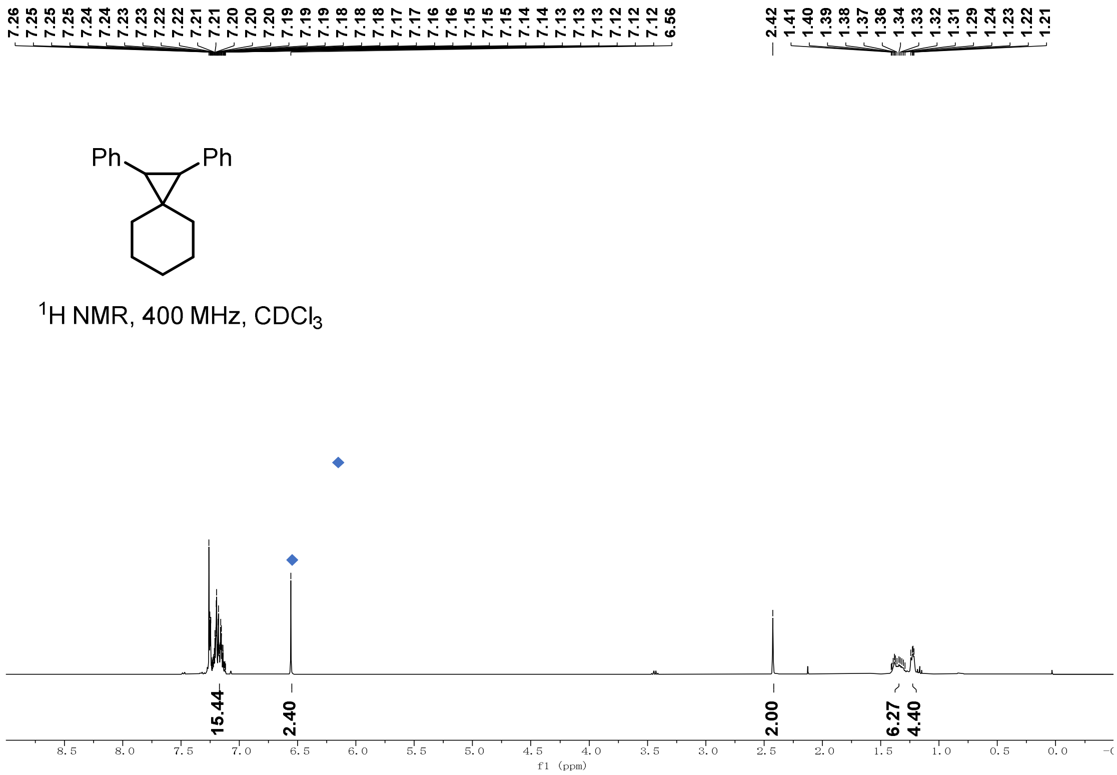

Ph
Ph
^{1}H NMR, 400 MHz, CDCl$_3$
15.44
2.40
2.00
6.27
4.40
f1 (ppm)

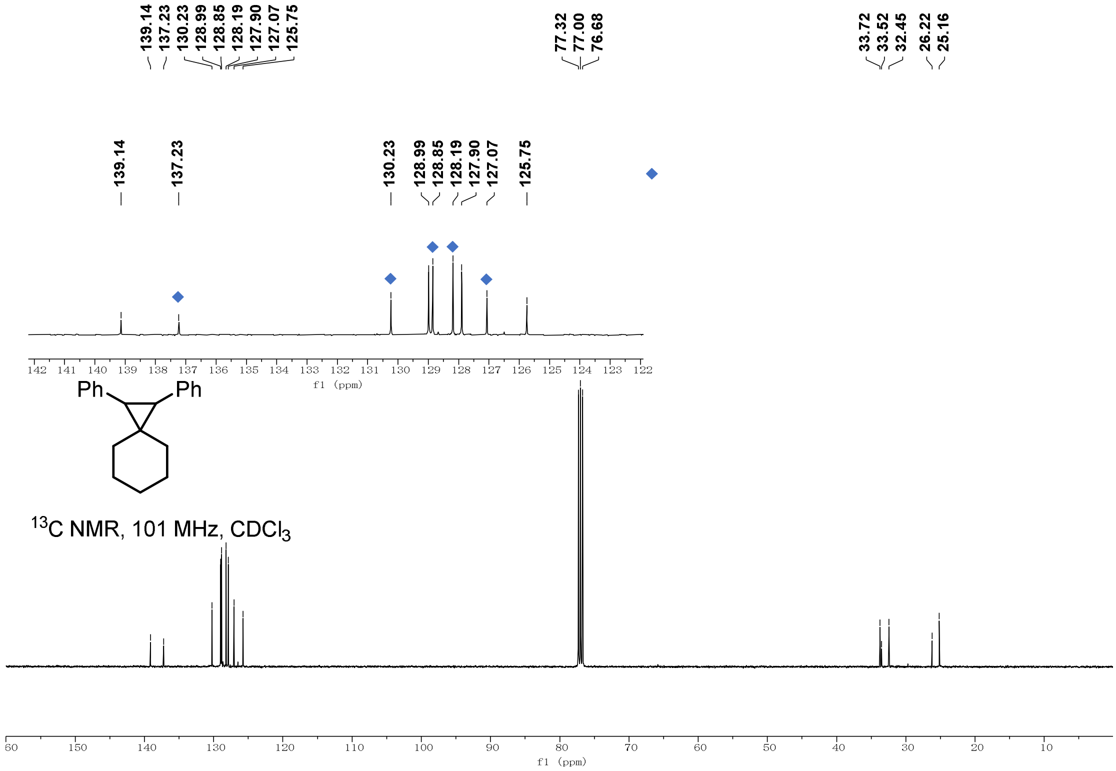

139.14
137.23
130.23
128.99
128.85
128.19
127.90
127.07
125.75
77.32
77.00
76.68
33.72
33.52
32.45
26.22
25.16
Ph
Ph
^{13}C NMR, 101 MHz, CDCl$_3$
f1 (ppm)

9. GC spectra

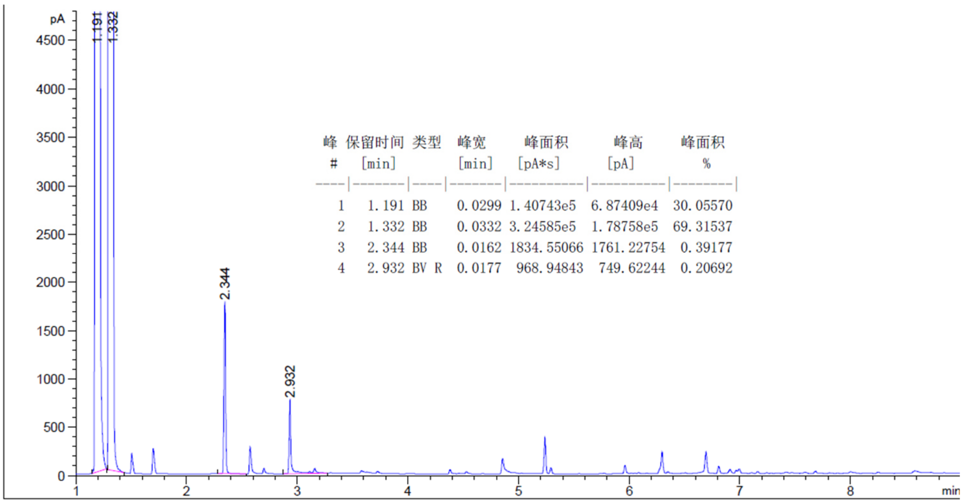

Figure S10. GC Spectrum of **3o**. RT = 2.932 min (area = 968.9); **3o**, 2.344 min (area = 1834.5): anisole (internal std.).

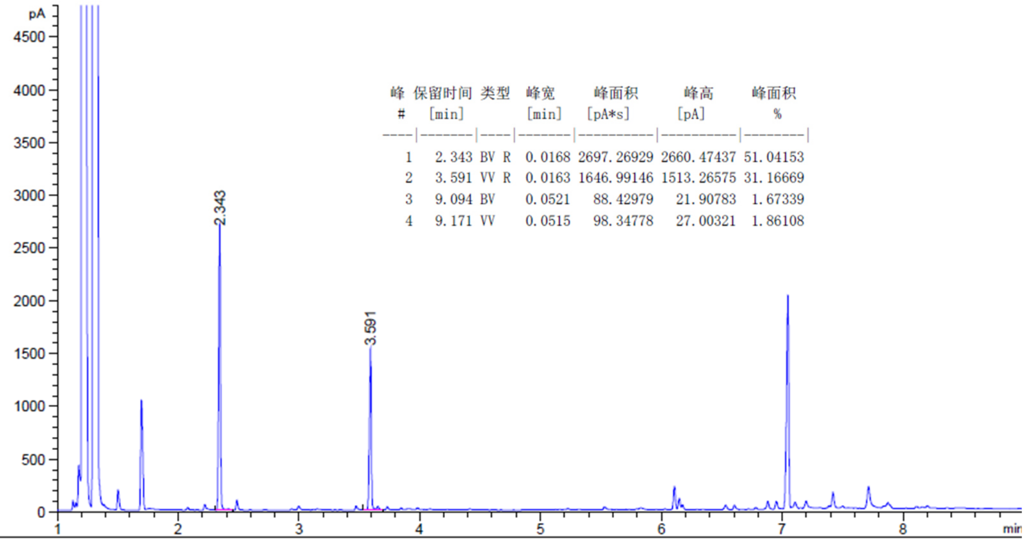

Figure S11. GC Spectrum of **3p**. RT = 3.591 min (area = 1646.99); **3p**, 2.343 min (area = 2697.3): anisole (internal std.).

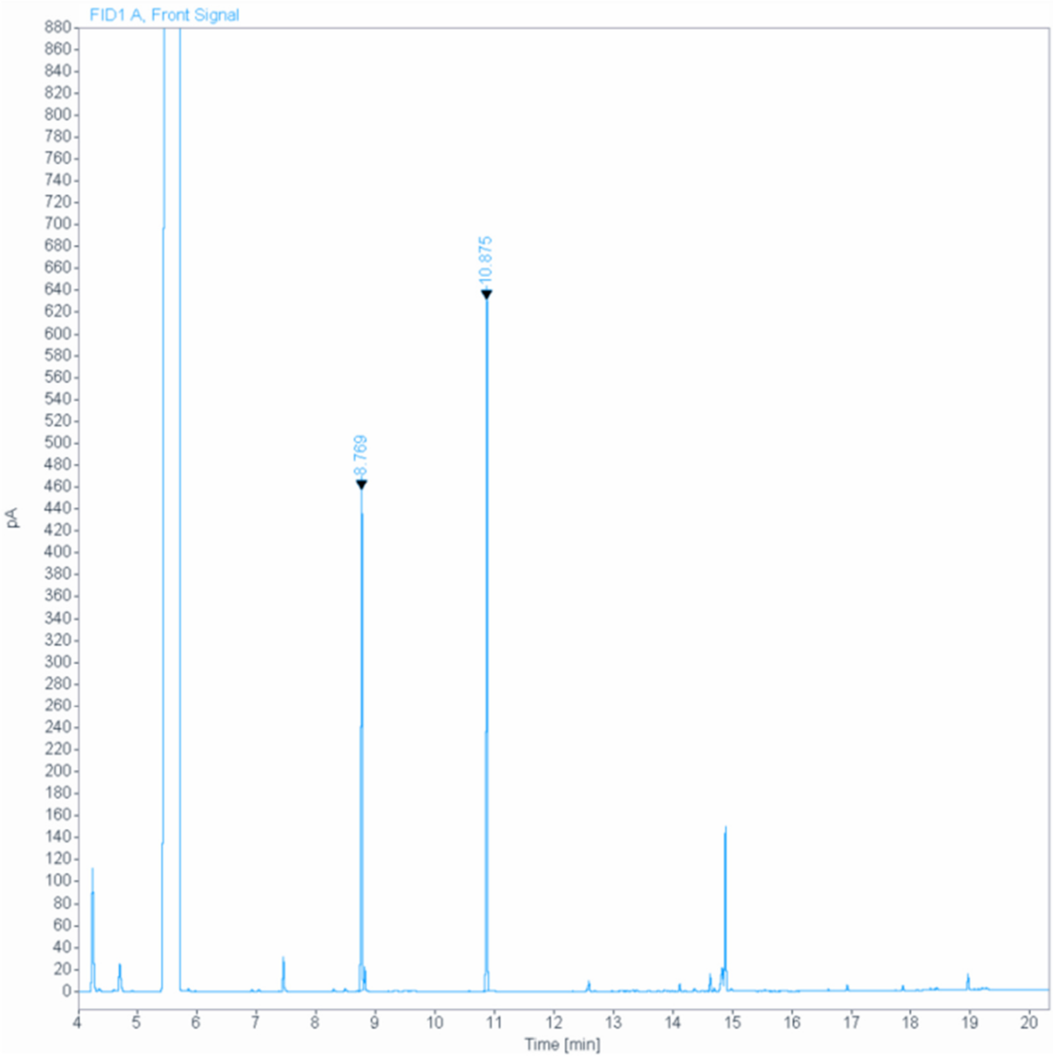

Figure S12. GC Spectrum of **3q**. t = 8.769 min: **3q**; 10.875 min: *n*-dodecane (internal std.).

Signal: FID1 A, Front Signal

RT [min]	Type	Width [min]	Area	Height	Area%	Name
8.769	MM	0.0247	678.8668	457.7612	43.8198	
10.875	BB	0.0209	870.3565	632.1578	56.1802	
		Sum	1549.2233			

Figure S13. Integration of the GC Spectrum of **3q**. RT = 8.769 min; **3q**, 10.875 min: *n*-dodecane (internal std.). n(std) = 87.25 µmol.

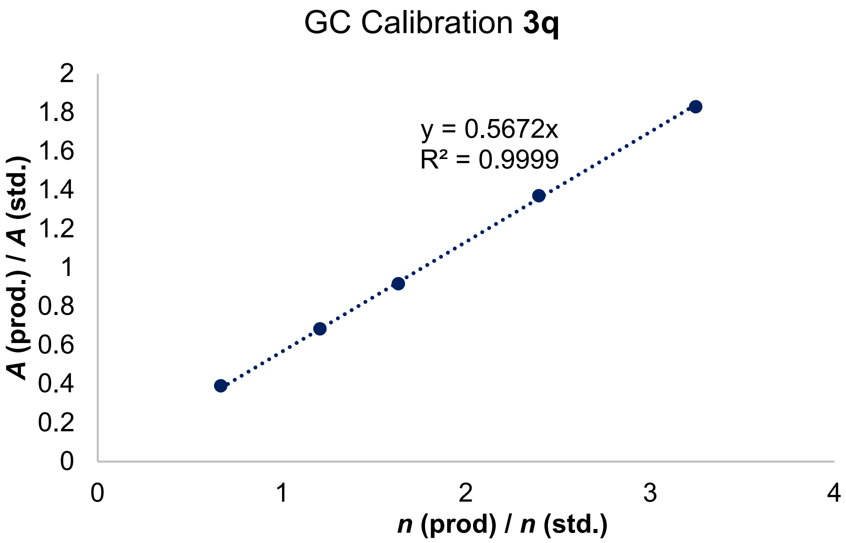

Figure S14. GC Calibration curve of **3q** with dodecane as internal standard.

10. References

[1] S. Wang, B.-Y. Cheng, M. Sršen, B. König, *J. Am. Chem. Soc.* **2020**, *142*, 7524-7531.

[2] a)C. Empel, C. Pei, R. M. Koenigs, *Chem. Commun.* **2022**, *58*, 2788-2798; b) S. Jana, C. Pei, C. Empel, R. M. Koenigs, *Angew. Chem. Int. Ed.* **2021**, *60*, 13271-13279; c) Z. Yang, M. L. Stivanin, I. D. Jurberg, R. M. Koenigs, *Chem. Soc. Rev.* **2020**, *49*, 6833-6847; d) J. Durka, J. Turkowska, D. Gryko, *Eur. J. Org. Chem.* **2021**, *9*, 8895-8918.

[3] K. Yang, Q. Li, Y. Liu, G. Li, H. Ge, *J. Am. Chem. Soc.* **2016**, *138*, 12775-12778.

[4] K. Palakodety Radha, R. Ch. Govardhan, G. V. M. Sharma, *Lett. Org. Chem.* **2004**, *1*, 212-214.

[5] T. Shin, H. Kim, S. Kim, A. Lee, M.-S. Seo, J. Choi, H. Kim, H. Kim, *Org. Lett.* **2019**, *21*, 5789-5792.

[6] D. J. Vyas, E. Larionov, C. Besnard, L. Guénée, C. Mazet, *J. Am. Chem. Soc.* **2013**, *135*, 6177-6183.

[7] M.-A. Schneider, K. Seifert, *Eur. J. Org. Chem.* **2017**, *2017*, 6739-6746.

[8] G. S. Lee, J. Won, S. Choi, M.-H. Baik, S. H. Hong, *Angew. Chem. Int. Ed.* **2020**, *59*, 16933-16942.
